# DriverMP enables improved identification of cancer driver genes

**DOI:** 10.1093/gigascience/giad106

**Published:** 2023-12-13

**Authors:** Yangyang Liu, Jiyun Han, Tongxin Kong, Nannan Xiao, Qinglin Mei, Juntao Liu

**Affiliations:** School of Mathematics and Statistics, Shandong University (Weihai), Weihai 264209, China; School of Mathematics and Statistics, Shandong University (Weihai), Weihai 264209, China; School of Mathematics and Statistics, Shandong University (Weihai), Weihai 264209, China; School of Mathematics and Statistics, Shandong University (Weihai), Weihai 264209, China; MOE Key Laboratory of Bioinformatics, BNRIST Bioinformatics Division, Department of Automation, Tsinghua University, Beijing 100084, China; School of Mathematics and Statistics, Shandong University (Weihai), Weihai 264209, China

**Keywords:** cancer genomics, driver genes, multiomics in cancer, mutated gene pairs

## Abstract

**Background:**

Cancer is widely regarded as a complex disease primarily driven by genetic mutations. A critical concern and significant obstacle lies in discerning driver genes amid an extensive array of passenger genes.

**Findings:**

We present a new method termed DriverMP for effectively prioritizing altered genes on a cancer-type level by considering mutated gene pairs. It is designed to first apply nonsilent somatic mutation data, protein‒protein interaction network data, and differential gene expression data to prioritize mutated gene pairs, and then individual mutated genes are prioritized based on prioritized mutated gene pairs. Application of this method in 10 cancer datasets from The Cancer Genome Atlas demonstrated its great improvements over all the compared state-of-the-art methods in identifying known driver genes. Then, a comprehensive analysis demonstrated the reliability of the novel driver genes that are strongly supported by clinical experiments, disease enrichment, or biological pathway analysis.

**Conclusions:**

The new method, DriverMP, which is able to identify driver genes by effectively integrating the advantages of multiple kinds of cancer data, is available at https://github.com/LiuYangyangSDU/DriverMP. In addition, we have developed a novel driver gene database for 10 cancer types and an online service that can be freely accessed without registration for users. The DriverMP method, the database of novel drivers, and the user-friendly online server are expected to contribute to new diagnostic and therapeutic opportunities for cancers.

## Introduction

Cancer is one of the most complex diseases and accounts for 1 in 6 deaths, making it the second most common cause of death globally [1]. The most widely accepted theory is that cancer is mainly caused by genetic mutations [2]. For this reason, several large-scale cancer sequencing projects, such as The Cancer Genome Atlas (TCGA) [3], the International Cancer Genome Consortium (ICGC) [4] and Therapeutically Applicable Research to Generate Effective Treatments (TARGET) [5], have generated a large amount of multiomics data for various cancer types, and the resulting databases have accelerated the discovery of cancer genes. Related studies have shown that among the large number of somatic mutated genes, only a small number of them (the so-called driver genes) confer a selective advantage to cancers, and most of them (the so-called passenger genes) exhibit little impact on cancer progression [2, 6, 7].

A key issue and major challenge is to distinguish driver genes from a very large number of passenger genes [2]. The most basic and intuitive approach is to prioritize all somatic mutations according to their occurrence frequencies based on the hypothesis that drivers demonstrate higher mutation rates than expected [8]. A great number of methods have been developed to identify driver genes based on mutation frequencies; these include MutSig2.0 [9], MuSigCV [10], MuSiC [7], and WITER [11]. These approaches first attempt to estimate the background mutation rate (BMR) and then compare the mutation frequency of each gene with the BMR to identify driver genes. In addition, other methods, such as Mutation_assessor [12], CHASM [13], transFIC [14], and FATHMM [15], distinguish driver genes from passenger genes by assessing the functional impact of mutations.

Although significant efforts have been made in the accurate identification of driver genes, mutation frequency–based approaches have shown limited power in practical applications because most cancers demonstrate extensive mutational heterogeneity across samples [16]. Studies have shown that only a small number of cancer drivers are frequently mutated, and most of them are mutated in a few samples, which is the so-called long-tail phenomenon [2, 6, 7]. This phenomenon highlights an enormous challenge in the identification of rarely mutated driver genes by methods based on mutation frequency. Fortunately, recent studies revealed that driver genes in a given cancer type usually act together in a limited number of biological pathways or protein complexes, although the cancer genes demonstrate a random distribution across different samples [17, 18]. Therefore, rarely mutated drivers may be identified by applying information on biological pathways or networks. Based on this fact, quite a few approaches have been developed to increase the prediction accuracy. Functional network-based methods, such as HotNet2 [17] and VarWalker [19], predict numerous “cancer modules” containing drivers rather than individual genes by using the “heat diffusion”–like model in physics to determine “mutation scores” in protein‒protein interaction (PPI) networks. Mutation- and network-based methods, such as MUFFINN [16] and MaxMIF [20], were developed by integrating the information of both mutation and functional networks to prioritize genes. MUFFINN defined 2 kinds of mutation scores for ranking genes by considering mutations in the most frequently mutated neighbor (direct neighbor maximum, DNmax) and genes in all direct neighbors with normalization by their degree connectivity (direct neighbor sum, DNsum). MaxMIF involves a maximum mutation impact function that considers the mutation frequencies of 2 genes and the interaction strength between them in the PPI network. These approaches effectively improve the prediction of driver genes involved in the biological networks; however, their false-positive rates are still too high. Therefore, new algorithms considering more meaningful biological information are urgently needed.

Related studies show that driver genes tend to change the expression of their interacting partners or genes that share the same biological pathways, which directly alters the expression of all genes in a biological subnetwork or in pathways associated with driver genes. In contrast, passenger genes do not usually cause significant changes in gene expression [21]. Therefore, making better use of gene expression data will undoubtedly facilitate the identification of driver genes. Several related methods have been developed; these include iPDG [22], DriverNet [21], and DawnRank [23], which prioritize genes by combining multiple types of data, including mutation, PPI network, biological pathway, and differential gene expression data. iPDG evaluates the changes in expression levels of “key genes” to identify potential driver genes by using DNA copy number variation, somatic mutation, and gene expression data. Both DriverNet and DawnRank rank potential driver genes based on their impact on the overall differential expression of downstream genes in a molecular interaction network. The above methods, which combine multiple types of biological information, have made the identification of driver genes more accurate and reliable; however, their prediction effects are still far from satisfactory.

We introduce a new method called DriverMP that prioritizes cancer genes on a cancer-type level by utilizing mutation data (only nonsilent somatic mutations affecting protein-coding genes are considered in this study), PPI networks, and differential expression data. The new approach effectively improves the identification of driver genes by introducing the following innovations. (i) Based on our observation that most cancer driver genes have a driver neighbor in the PPI network, DriverMP first prioritizes the gene pairs, and then the individual mutated genes are prioritized by dividing each gene pair. (ii) Considering that driver genes are expected to exhibit different expression patterns and simultaneously influence the expression of their interacting genes in a biological subnetwork, DriverMP constructs a new network, called the differential expression network, to quantify the differential expression of the subnetwork centered on a gene pair. (iii) According to another observation that driver genes tend to converge to a limited number of biological pathways or protein complexes, DriverMP quantifies the association strength between each gene pair and its mutated neighbors in the PPI network. (iv) Combining the topological properties from both the PPI network and differential expression network, DriverMP generates an impact score for each mutated gene pair and then generates an impact score for each mutated gene based on the contribution of the gene to complete the prioritization of cancer genes.

Based on the known driver genes, the performance of DriverMP was evaluated in 10 common cancer types and compared with the performance of 10 other state-of-the-art approaches, including MutSig2.0 [9], MutSigCV [10], Mutation_Assessor [18], MaxMIF [20], DawnRank [23], MUFFINN [16], DriverNet [21], OncoVar [24], driverMAPS [25], and DriverRWH [26], in terms of the receiver operating characteristic (ROC) curve, area under the ROC curve (AUC), cumulative number curve of the top 500 genes, F1-score curve of the top 500 genes, and area under the F1-score curve (AUFC). DriverMP consistently demonstrated the best performance in all 10 cancer types.

With respect to the novel drivers, DriverMP identified multiple potential driver genes with high impact scores for each cancer type, and these findings are strongly supported by the results of clinical experiments, disease enrichment analysis, or biological pathway analysis. For example, we found 14 and 10 novel candidates that are significantly related to breast cancer and breast cancer–related diseases, respectively, via disease enrichment analysis. In addition, another 6 genes, PRKDC, NCL, CCNA2, AXL, GLI3, and SUPT5H, were identified to be highly related to the growth and development of breast cancer; the overall and postprogression survival in patients with breast cancer; the regulation of the gene expression that controls the proliferation, migration, cell cycle, and apoptosis of breast cancer MDA-MB-231 cells; and so on. We provided a database of those potential driver genes with detailed descriptions of strong biological or clinical evidence for each of the 10 cancer types. Moreover, we observed that the novel candidates enriched in the corresponding cancer type according to disease enrichment analysis formed a significantly dense and highly weighted subnetwork in the PPI network (the *P* value is approximately 0). This observation coincided with previous findings that mutated genes in the cancer genome tend to converge in a few biological pathways [18] and that genes act together in various signaling and regulatory pathways and protein complexes [17]. The new method and the identified novel driver candidates are expected to contribute to a deeper understanding of the architecture of cancers and new diagnostic and therapeutic opportunities for cancers.

## Results

### Overview of DriverMP

**Figure 1:**
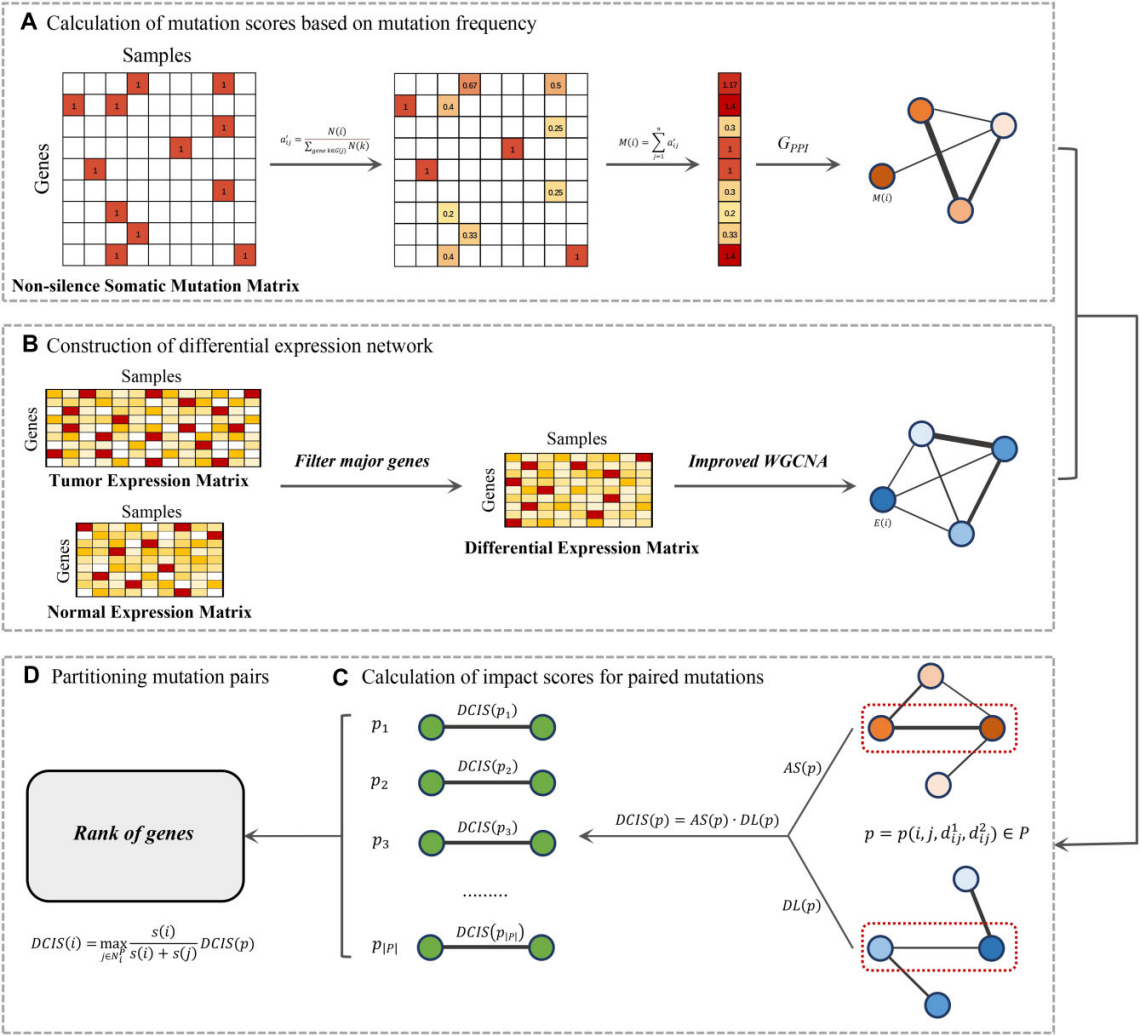
Workflow of DriverMP. (A) Normalization of the mutation matrix and calculation of mutation scores. (B) Preprocessing of the differential expression data and construction of the differential expression network *G_diff_*. (C) Calculation of impact scores (DCIS (driving cancer impact score) by AS (association strength) and DL (differential level) from *sub-G_PPI_* and *sub-G_diff_*, respectively) for mutated gene pairs. (D) Prioritization of individual mutated genes by partitioning mutated gene pairs.

Based on our observation that most driver genes have a driver neighbor in the PPI network, we propose that assessment of gene pairs would capture cancer driver characteristics in a more realistic pattern than direct assessment of individual genes. Therefore, unlike conventional approaches that prioritize individual mutated genes directly, DriverMP is designed to first prioritize gene pairs. According to another observation that driver genes tend to converge to a limited number of biological pathways or protein complexes, those gene pairs will be ranked higher if they are strongly associated with other genes in the PPI network. The new framework will quantify the association strength between each mutated gene pair and its mutated neighbors in the PPI network. Considering that driver genes are expected to exhibit different expression patterns and simultaneously influence the expression of their interacting genes in a biological subnetwork, a mutated gene pair is ranked highly if it and the subnetwork centered on it are highly differentially expressed. The new DriverMP approach quantifies the differential expression of the subnetwork centered on a mutated gene pair. DriverMP is a novel approach that involves an innovative strategy—it considers both biological networks and differential gene expression, which can facilitate the identification of rarely mutated cancer drivers (see Fig. 1).

### DriverMP was developed based on the characteristics of driver genes

To explore the interaction characteristics of known driver genes in the PPI network, we calculated 3 metrics: the percentage of codrivers (a driver gene is defined as a codriver if it has at least 1 driver neighbor in the PPI network), the average connection density (the connection density of a driver gene is defined as the number of its driver neighbors), and the average connection strength (the connection strength of a driver gene is defined as the sum of the edge weights between the gene and its driver neighbors).

1. **Driver genes usually interact with one another**. To investigate the interactions between driver genes, we calculated the percentage of driver genes occurring with other driver genes among all known driver genes, and the results showed that the percentages reached 94.52% and 99.92%, respectively, based on the HumanNet [27] and STRINGv10 [28] PPI networks. In addition, we calculated the percentage of passenger genes (not included in the benchmark reference) that have at least 1 driver neighbor, and it was only 75.92% and 96.49% based on the 2 networks, which demonstrates the obvious phenomenon of codrivers in PPI networks.
2. **The interactions between driver genes are highly dense**. The average connection density of known driver genes reached 18.88 and 130.04 based on the HumanNet and STRINGv10 PPI networks, respectively. However, the average number of edges between passenger and driver genes was only 7.08 and 50.30 in the 2 networks, which also clearly demonstrates the phenomenon of dense interactions between drivers.
3. **The dense interactions between driver genes are extremely strong**. The average strength score for connections between driver genes reached 5.61 and 44.72 in the HumanNet and STRINGv10 PPI networks, respectively. In comparison, the average weight of the edges between passenger and driver genes was only 1.93 and 14.96 based on the 2 PPI networks, demonstrating that the interactions between driver genes are generally stronger than those between passenger genes.

These observations suggest the interaction properties of driver genes in the PPI network, which seems consistent with a previous observation that driver genes tend to be enriched in a few biological pathways, where gene‒gene interactions are more frequent. Based on these interaction characteristics of driver genes, the DriverMP method was developed to focus on the prioritization of mutated gene pairs.

### DriverMP demonstrates great improvement in overall performance

To evaluate the overall performance of DriverMP in the prediction of driver genes, we employed nonsilent somatic mutation data and differential expression data collected for 10 cancer types from the TCGA, including BRCA, PRAD, LUAD, LUSC, KIRC, KIRP, HNSC, COADREAD, UCEC, and BLCA, and 2 independently developed PPI networks (based on STRINGv10 and HumanNet) were applied (see the Methods section for details). To evaluate the improvements of DriverMP over the other state-of-the-art methods, 10 driver gene predictors (MutSig2.0, MutSigCV, Mutation_Assessor, DawnRank, MaxMIF, MUFFINN [DNmax and DNsum], DriverNet, OncoVar, driverMAPS, and DriverRWH) were selected for the overall performance comparison. Additionally, we added a comparison with the frequency-based approach named Freq-based, which ranks genes based solely on frequency. The overall performance of all the methods was compared under the criteria of ROC curves and AUC values, which effectively evaluate the overall sensitivity and specificity of driver candidate prioritization.

**Figure 2:**
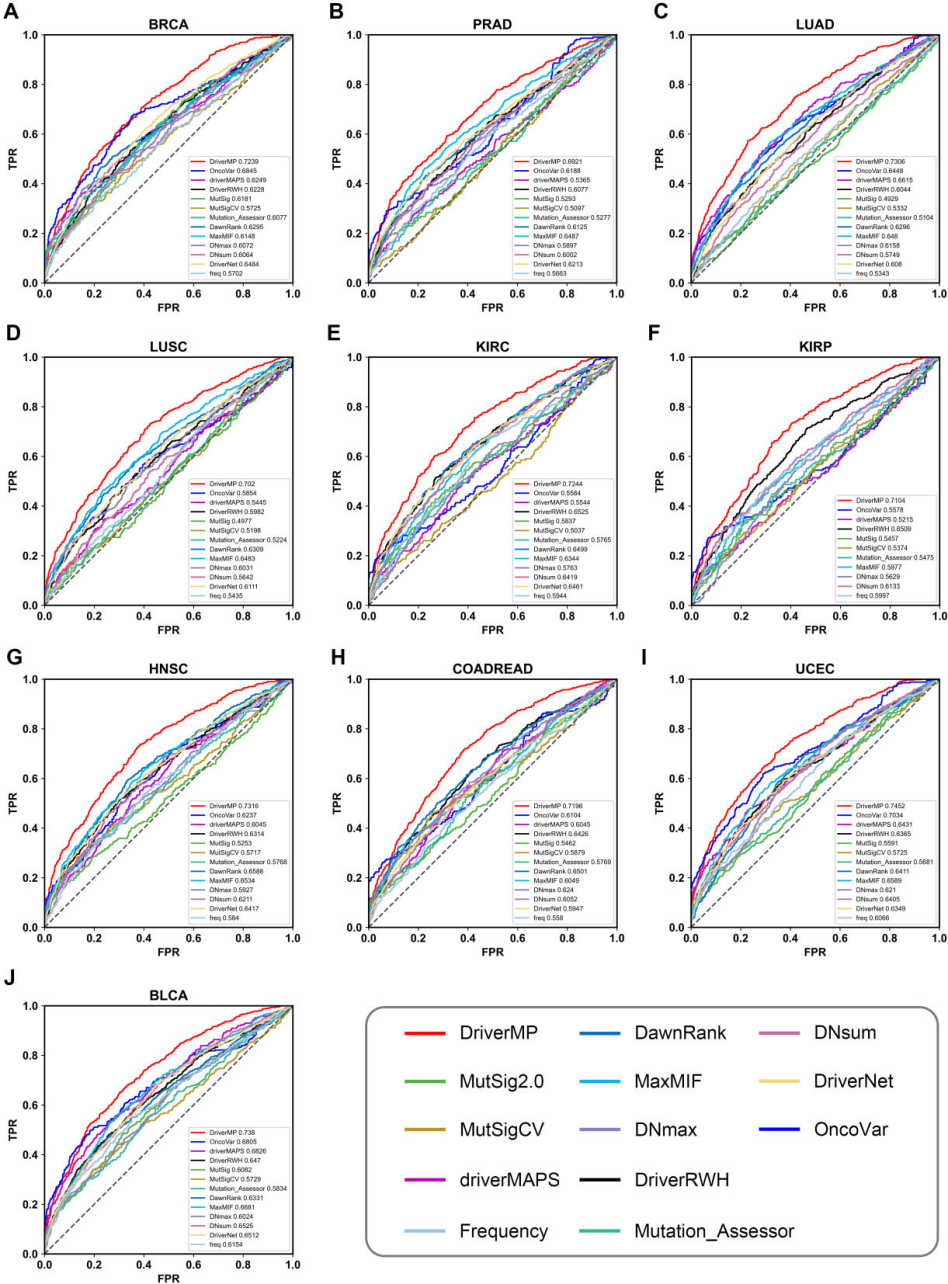
ROC curves of DriverMP and the other compared methods in (A) BRCA, (B) PRAD, (C) LUAD, (D) LUSC, (E) KIRC, (F) KIRP, (G) HNSC, (H) COADREAD, (I) UCEC, and (J) BLCA, using the STRINGv10 network.

After comparison based on ROC curves, DriverMP demonstrated high improvements over all the compared methods on all 10 datasets when using the STRINGv10 PPI network (see Fig. 2). Similar results were obtained with the HumanNet PPI network (see Supplemental Materials for details). In addition, the AUC values of the ROC curves were calculated for the compared methods in the analysis of the STRINGv10 PPI network, and the results showed that the AUC values of DriverMP were much higher than those of all the other methods in all 10 cancer types, and the average improvement rate of DriverMP over the other compared methods in the 10 datasets reached 7.98% to 26.69%. Similar improvements were obtained with DriverMP in the analysis of the HumanNet PPI network (see Supplemental Materials for details). Therefore, DriverMP shows the best overall performance among all the compared methods under the criteria of ROC curves and AUC values.

### DriverMP shows great improvement in identifying top-ranked genes

In practical applications, only the top-ranked genes can be confirmed by subsequent experiments. To compare the performance of DriverMP and other methods based on top-ranked genes, we analyzed the top 500 genes ranked by each method and compared the number of identified known driver genes in the benchmark reference, the F1-score, and the AUFC based on the STRINGv10 network.

**Figure 3:**
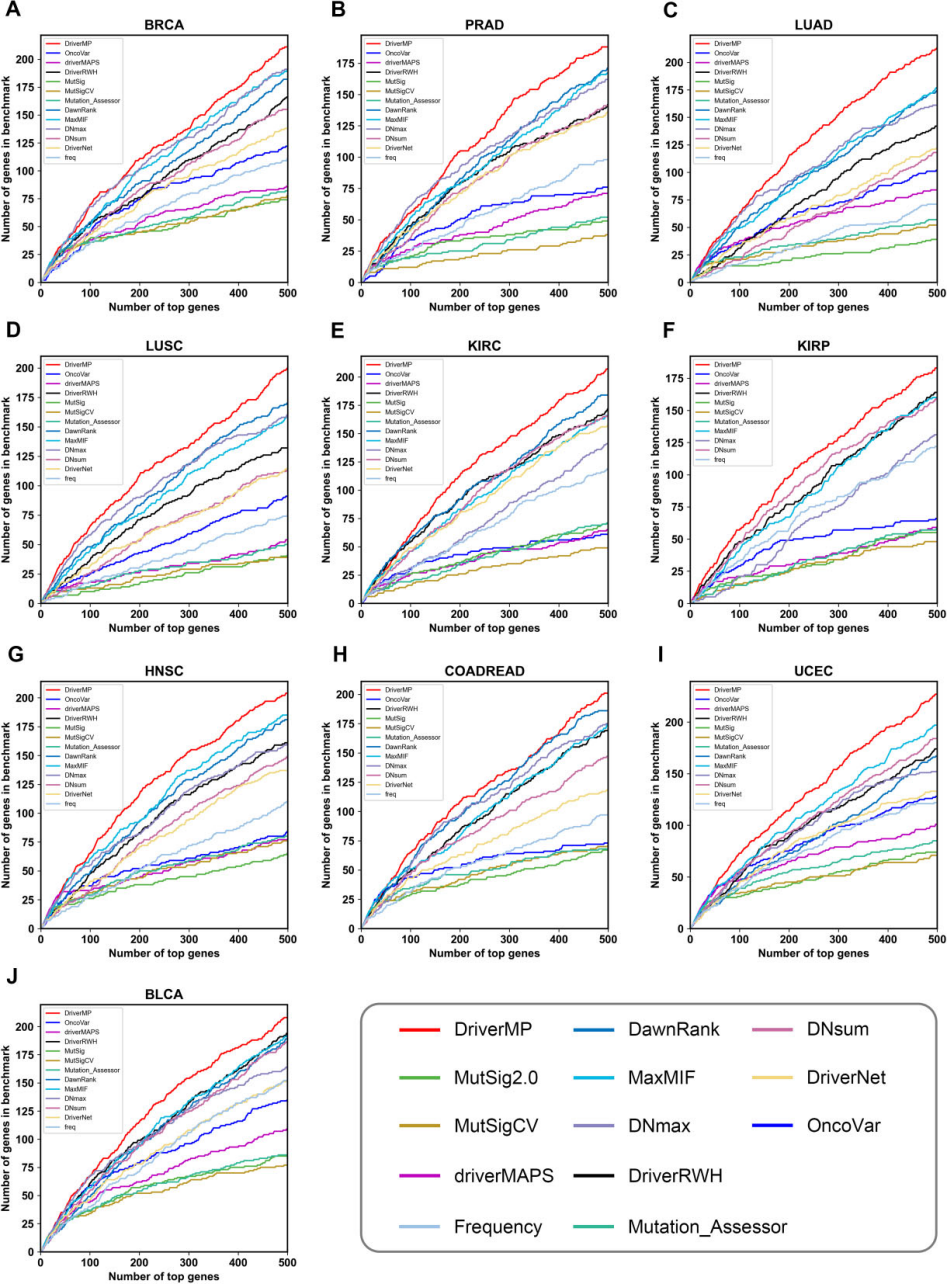
The curves of the numbers of identified known driver genes of the top-ranked 500 genes in (A) BRCA, (B) PRAD, (C) LUAD, (D) LUSC, (E) KIRC, (F) KIRP, (G) HNSC, (H) COADREAD, (I) BLCA, and (J) BLCA, using the STRINGv10 PPI network.

**Figure 4:**
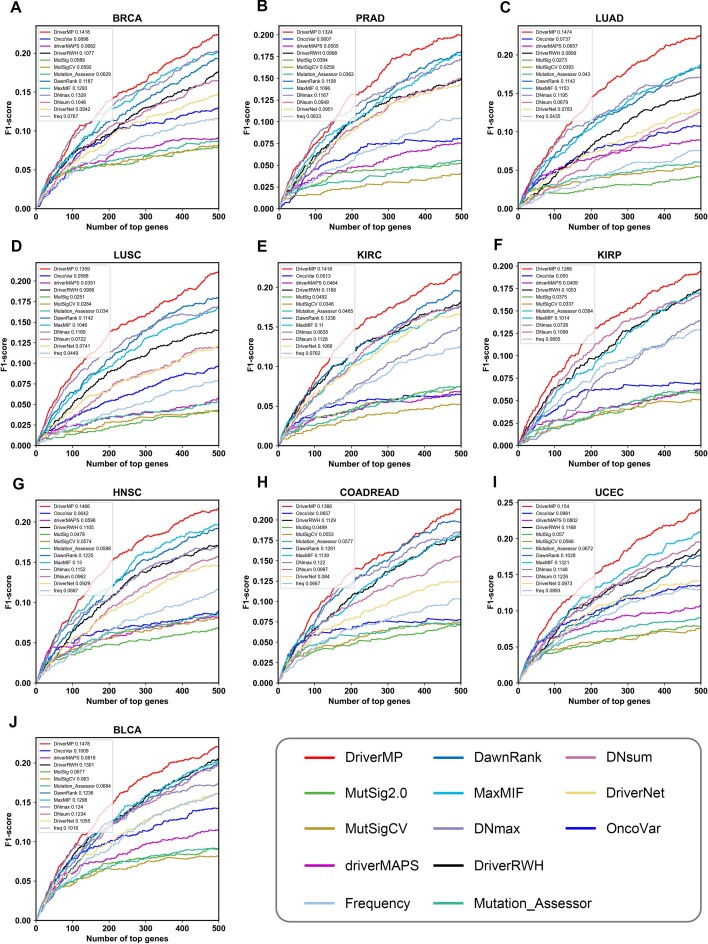
Performance of F1-scores of DriverMP and other compared methods in 10 cancer types—(A) BRCA, (B) PRAD, (C) LUAD, (D) LUSC, (E) KIRC, (F) KIRP, (G) HNSC, (H) COADREAD, (I) BLCA, and (J) BLCA—using the STRINGv10 network.

After comparison, DriverMP showed high improvements over all the others in the 10 cancer types in terms of the number of identified known driver genes (see Fig. 3). To evaluate the overall performance of DriverMP by combining the sensitivity and precision, the F1-score curves and the AUFC values were calculated, and the results showed that DriverMP consistently performed the best among all the compared methods on all 10 datasets (see Fig. 4). Specifically, the average improvements of DriverMP over the others on the 10 datasets reached 2.08% to 65.98% in terms of AUFC. Similar results were obtained by using the HumanNet PPI network under the 3 criteria (see Supplemental Materials for details). In conclusion, DriverMP demonstrated superior predictive power over all the other methods in identifying the top-ranked genes.

### DriverMP demonstrates stable performance with a reduced sample number

Although substantial efforts have been made in DNA sequencing to identify possible cancer genes, many cancer types have only a limited number of sequenced samples available due to the complexity, expense, and time-consuming nature of clinical experiments. Therefore, the performance of a practical driver gene identifier cannot be highly dependent on the number of samples. To evaluate the stability of DriverMP, we tested it under the following 2 conditions: (i) random selection of only 80%, 50%, and 20% of the samples in the constructed differential expression matrix and (2) random selection of only 80%, 50%, and 20% of the samples in the mutation dataset. Each of the above random selections was performed 10 times, and the average AUC and AUFC values were calculated to evaluate the performance of DriverMP.

**Figure 5:**
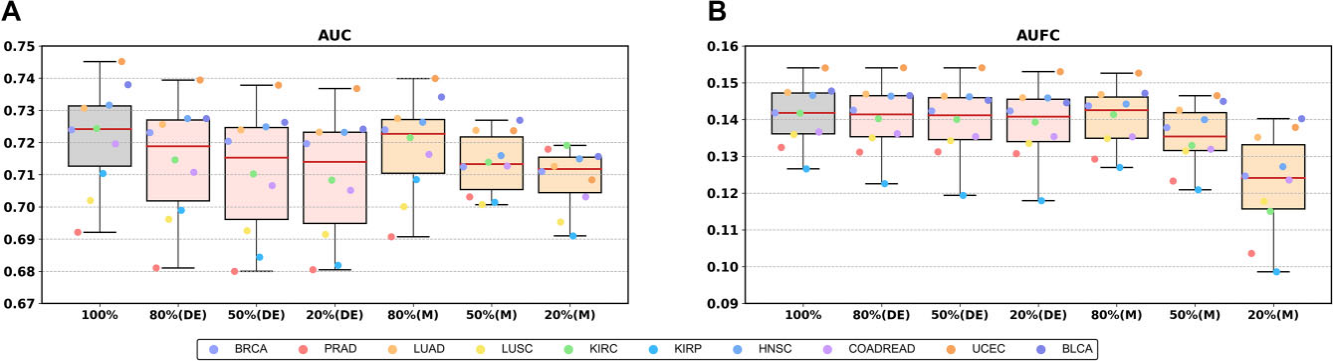
Results of the stability tests. This figure shows the boxplots of the effects of the number of samples on the performance of DriverMP in terms of mutation data (M) and differential expression (DE) data, measured by average AUC (A) and AUFC (B) of 10 times of random selections of different numbers of samples using the STRINGv10 network.

DriverMP exhibited highly stable performance in terms of differential expression analysis (see Fig. 5A, B), and only a slight decrease in prediction power was observed even when 80% of the samples were removed. For the mutation data, although the stability of DriverMP in predicting drivers was not as high as that in predicting differential expression, it still demonstrated stable performance, especially when 20% and 50% of the samples were removed. It seems that the stability of DriverMP is relatively worse when 80% of the samples were removed; however, the average differences in AUC and AUFC before and after sample removal were only 0.013 and 0.019, respectively, for the 10 cancer types. Therefore, DriverMP shows highly stable performance even when only a small number of samples are available.

The framework of DriverMP was designed by deeply mining the relationships among mutated genes and effectively fusing different sources of cancer data, which may be the reason why DriverMP is able to maintain stable performance even when many samples are removed. Moreover, the low dependence of DriverMP on the number of samples directly contributes to the identification of infrequently mutated driver genes buried in the long tail.

### DriverMP reliably predicts novel driver genes

In the absence of a well-defined and systematic approach to identifying driver candidates, we proposed a cancer-specific 6-level assessment by a comprehensive literature survey. We then applied it to assess the quality of the driver candidates predicted by DriverMP in all 10 cancer types. The main text shows the results for DriverMP analysis of 2 popular cancer types, breast cancer and lung adenocarcinoma, and similar results for DriverMP analysis of the other 8 cancer types are shown in the Supplemental Materials. The detailed rules of the 6-level assessment are illustrated as follows.

1. **Cancer-type level**. In this level of analysis, data are collected on driver candidates that are enriched in different cancer types from the well-known tool DAVID [29] against the Genetic Association Database (GAD) [30], which is a developing archive of human genetic association studies of complex diseases and disorders allowing the comprehensive analysis of complex common human genetic diseases.
2. **Literature-supported level**. In this level of analysis, the driver candidates are divided into 3 categories based on literature type: (i) overview studies (O) that summarize previous studies, (ii) experimental studies (E) that verify the mechanism of carcinogenesis via *in vivo* or clinical trials, and (iii) bioinformatics studies (B) that infer cancer mechanisms via reliable statistical or bioinformatics analysis of large multiomics datasets.
3. **Pathway level**. In this level of analysis, candidates that are enriched in biological pathways directly related to specific cancers with a false discovery rate (FDR) <10^–3^ are identified by using the tool STRING [31] against well-recognized biological pathway databases, such as KEGG [32] and Reactome [33].
4. **Noncancer disease level**. In this level of analysis, genes that are enriched in diseases that are associated with specific cancers are identified using DAVID against the GAD database.
5. **Gene level**. For this level of analysis, each novel candidate is collected if it has at least one homologue in CGC, which is achieved by sequence alignments using BLAST [34]. In detail, it is defined that a novel candidate has a homologue in CGC if the sequence similarity between the novel candidate and a CGC gene is higher than 40% with an e-value lower than 10^−4^.
6. **Validation-required level**. In the last level of analysis, the remaining genes that are not included in the above 5 levels are noted; these genes require further validation.

#### DriverMP reliably predicts novel drivers for breast cancer

Breast cancer (BC) is the most commonly diagnosed life-threatening cancer and the leading cause of cancer death in women [35]. In this study, we utilized DriverMP under both the STRINGv10 and HumanNet networks to identify potential driver genes in BC. We identified a total of 51 novel driver candidates that ranked within the top 250 in both networks and were not previously included in CGC. These candidates underwent the 6-level assessment and were subsequently divided into 6 groups (Table 1).

**Table 1:** Six-level assessment of the 51 novel driver candidates of BC

No.	Level of analysis	Driver candidates	Count	Percentage
1	Cancer type level	ABCB1, APEX1, CCNB1, CDC14A, CDK2, ERCC6, IGF1R, INSR, PIK3CB, PIK3CG, PRKDC, RAD51, TOP2A, TP53BP1	14	27.45
2	Literature-supported level	AXL, CCNA2, CCNB1, CDK5, ERCC6, GLI3, IGF1R, NCL, PRKDC, RAD51, SUPT5H, TP53BP1	12	23.53
3	Pathway level	ANK3, APEX1, AXL, CACNA1A, CCNA2, CCNB1, CD4, CDC14A, CDK2, CDK5, CHD3, CTNNA1, DLG1, EEF2, ERCC6, GLI3, HDAC1, HSPA8, HSPA9, IGF1R, INSR, MAPK3, MAPK8, MDC1, NR3C1, PIK3CB, PIK3CG, POLA1, PRCKDC, PRKDC, PTK2, RPS6KA1, SGK1, SIN3A, SLC2A4, SMARCA2, SP3, SUMO1, SUPT5H, TAF1, TOP2A, TP53BP1, TYK2, VAV1	44	86.27
4	Noncancer disease level	CDK5, IGF1R, INSR, MAPK3, NR3C1, PIK3CB, SGK1, SLC2A4, SMARCA2, SUMO1	10	19.61
5	Gene level	AXL, CACNA1A, CDK2, CDK5, CHD3, DLG1, GLI3, IGF1R, INSR, MAPK3, MAPK8, NEB, NR3C1, PIK3CB, PRKDC, PTK2, RPS6KA1, SGK1, SMARCA1, SMARCA2, SP3, TAF1, TYK2	23	45.10
6	Validation-required level	CAD, EEF1A1, TTN	3	5.88

##### Cancer-type level

**Figure 6:**
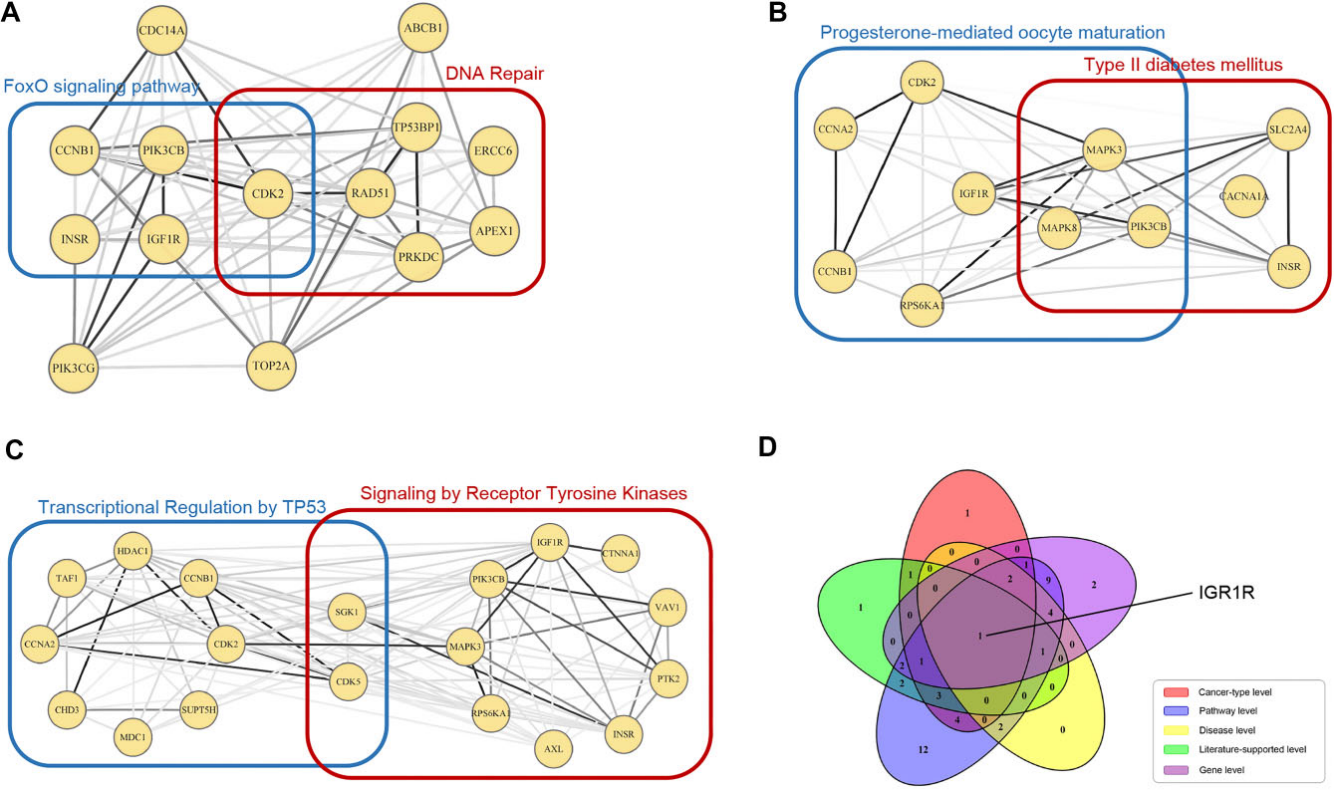
Six-level assessment of 51 driver candidates of breast cancer. (A) Subnetwork of the genes in the analysis at the “cancer-type level” from the STRINGv10 network. The 5 genes in the blue box are enriched in the “FoxO signaling pathway” (KEGG), and the 6 genes in the red box are enriched in “DNA repair” (Reactome). (B) Relationship of genes enriched in 2 biological pathways against KEGG from the STRINGv10 network. (C) Relationship of genes enriched in 2 biological pathways against Reactome from the STRINGv10 network. (D) Venn diagram of the 5 levels of breast cancer.

Based on the wealth of annotated genes associated with complex diseases in GAD, we used DAVID to confirm the relevance of the 51 driver candidates to breast cancer and found that 31 (60.8%) of these candidates were enriched in “cancer” ($P = 1.4\ \times {10}^{ - 9},\ FDR = 2.5\ \times {10}^{ - 8}$) and 14 (45.2%) of them were enriched in “breast cancer” ($P = 4.5\ \times {10}^{ - 8},\ FDR = 3.3\ \times {10}^{ - 5}$). Therefore, analysis at the cancer-type level yielded 14 novel genes that are noted as being related to BC. In addition, we performed pathway enrichment for the 14 genes in the first level of analysis, and the results showed that 5 genes, IGF1R, INSR, CDK2, PIK3CB, and CCNB1, were enriched in the “FoxO signaling pathway” (KEGG, $FDR = 5.2\ \times {10}^{ - 3}$), and 6 genes, CDK2, RAD51, PRKDC, APEX1, ERCC6, and TP53BP1, were enriched in “DNA repair” (Reactome, $FDR = 6.7\ \times {10}^{ - 3}$) (Fig. 6A). Related studies have shown that the lack of functional FOXO proteins such as FOXO3a leads to the development of breast tumors [36, 37] and that DNA repair-related pathways play an important role in the pathogenesis of breast cancer [38].

##### Literature-supported level

Based on the comprehensive survey of reliable literature, 12 novel genes supported by relevant studies were classified into this group (Table 2). The function of each of the 12 genes and the relationship of each gene with breast cancers are briefly described in Table 2. For example, studies have demonstrated that suppressing CDK5 can impede cell motility and tumor development in the mesenchymal breast cancer cell lines MDA-MB-231 and BT549 [39], while elevated expression of PRKDC promotes breast cancer cell proliferation by regulating p38 MAPK signaling [40].

**Table 2:** Twelve driver candidates of “literature-supported level” of breast cancer

Gene	NCBI Entrez ID	Rank (HumanNet)	Rank (STRINGv10)	Function	Type
IGF1R	3480	13	133	IGF1R, as part of insulin-like growth factor signaling, is highly overexpressed in most malignant tissues, where it functions as an antiapoptotic agent by enhancing cell survival [41].	O
RAD51	5888	20	67	Breast cancer driver gene BRCA2 directed the binding of RAD51 recombinase to single-stranded DNA, reduced the binding of RAD51 to duplex DNA, and stimulated RAD51-mediated DNA strand exchange [42].	E
TP53BP1	7158	50	105	TP53BP1 may be associated with breast cancer staging and breast cancer prognosis [43].	B
ERCC6	2074	73	111	Integrative genomics approach suggests that ERCC6 may be a previously unreported low- to moderate-risk breast cancer susceptibility gene, which may also interact with ERCC8 [44].	B
CDK5	1020	119	196	CDK5 is commonly overexpressed and significantly correlated with several poor prognostic parameters of breast cancer. Its overexpression also exhibited a potential synergy in promoting TGF-β1–induced EMT (epithelial-mesenchymal transition) [39].	E
CCNB1	891	213	220	CCNB1 is a biomarker for the prognosis of ER^+^ breast cancer and monitoring of hormone therapy efficacy [45].	B
PRKDC	5591	16	20	PRKDCs are all involved with the growth and development of breast cancer cells [40].	E
NCL	4691	42	222	NCL is commonly overexpressed in human breast tumors, and its expression correlates with that of NCL-dependent microRNAs [46].	E
CCNA2	890	61	127	Kaplan–Meier survival analyses confirmed that elevated CCNA2 and CCNB1 expression levels were associated with overall and postprogression survival and recurrence-free probability rates in patients with BRCA [47].	B
AXL	558	153	70	In breast cancer, AXL expression has been observed in all of the main transcriptional subtypes, and AXL expression in primary breast tumors is strongly predictive of reduced patient survival and poor outcome [48].	E
GLI3	2737	178	45	ERα^+^ BRCA cell growth is dependent on Gli3, which indicates that Gli might be a preferential target for the clinical management of ERα^+^ BRCA [49].	E
SUPT5H	6829	231	206	SUPT5H plays an important role in BRCA tumorigenicity by regulating the expression levels of genes that control the proliferation, migration, cell cycle, and apoptosis of breast cancer MDA-MB-231 cells [50].	E

##### Pathway level

To investigate whether the enriched biological pathways are associated with breast cancers, pathway enrichment was performed using the 51 driver candidates. A total of 15 and 13 breast cancer–related biological pathways were identified using the KEGG and Reactome databases, respectively, covering 44 of the 51 driver candidates (see Table 3 for the functional descriptions of the pathways and their associations with breast cancers). Specifically, the KEGG enrichment results showed that 8 and 6 genes were enriched in 2 pathways, “progesterone-mediated oocyte maturation” ($FDR = 3.6{\mathrm{\ }} \times {10}^{ - 8}$) and “type 2 diabetes mellitus” ($FDR = 4.7{\mathrm{\ }} \times {10}^{ - 7}$), respectively, both of which are directly associated with breast cancers (Fig. 6B). Multiple studies have reported that the pathogenesis and prognosis of breast cancer are highly related to progesterone-mediated oocyte maturation [51, 52]. The correlation between type 2 diabetes and BC is also very strong, which will be discussed in detail in the next section. Reactome analysis showed that 10 (19.6%) and 11 (21.6%) genes were enriched in the “transcriptional regulation by TP53” ($FDR = 2.5{\mathrm{\ }} \times {10}^{ - 5}$) and “signaling by receptor tyrosine kinases” ($FDR = 2.8{\mathrm{\ }} \times {10}^{ - 6}$) pathways, respectively (Fig. 6C). TP53 is a well-known tumor suppressor gene that drives multiple cancers, including BC [53]. Under stress conditions, it regulates the transcription of many genes involved in various cellular processes, such as cellular metabolism, survival, senescence, apoptosis, and the DNA damage response [54]. For the “signaling by receptor tyrosine kinases” pathway, several studies have suggested that high levels of receptor tyrosine kinases (RTKs) may be associated with increased breast cancer aggressiveness and decreased overall and disease-free survival [55–60].

**Table 3:** Biological pathways associated with breast cancer

Database	ID	Pathway	Genes	Count	Function	FDR
**KEGG**	hsa04068	FoxO signaling pathway	CCNB1, CDK2, IGF1R, INSR, MAPK3, MAPK8, PIK3CB, SGK1, SLC2A4	9	Lack of functional FOXO proteins such as FOXO3a leads to the development of breast tumors [36, 37].	2.26E-08
	hsa04914	Progesterone-mediated oocyte maturation	CCNA2, CCNB1, CDK2, IGF1R, MAPK3, MAPK8, PIK3CB, RPS6KA1	8	Several bioinformatic analyses suggest that the pathogenesis and prognosis of breast cancer are associated with progestin-mediated oocyte maturation [51, 52].	3.59E-08
	hsa04930	Type 2 diabetes mellitus	CACNA1A, INSR, MAPK3, MAPK8, PIK3CB, SLC2A4	6	It is reported that up to 16% of patients with BC have diabetes, and type 2 diabetes may be associated with a 10–20% increased relative risk of BC [61, 62].	4.74E-07
	hsa04110	Cell cycle	CCNA2, CCNB1, CDK2, CDC14A, HDAC1, PRKDC	6	Therapeutic targeting of the cell cycle has long been viewed as a promising anticancer strategy [63].	4.31E-05
	hsa04152	AMPK signaling pathway	CCNA2, EEF2, IGF1R, INSR, PIK3CB, SLC2A4	6	Downregulation of AMPK activity or decreased level is involved in the promotion of breast tumorigenesis and thus activation of AMPK found to oppose tumor progression [64].	4.31E-05
	hsa05169	Epstein‒Barr virus infection	CCNA2, CDK2, HDAC1, MAPK8, PIK3CB, SIN3A, TYK2	7	A study confirmed the presence of Epstein–Barr virus (EBV) in one-third of BC and demonstrated that EBV-positive tumors presented with a more aggressive phenotype that could be useful when considering potential therapeutic targets [65].	4.31E-05
	hsa04150	mTOR signaling pathway	IGF1R, INSR, MAPK3, PIK3CB, RPS6KA1, SGK1	6	One of the most commonly altered pathways driving breast cancer cell growth, survival, and motility is the PI3K/AKT/mTOR signaling cascade [66].	8.25E-05
	hsa04151	PI3K-Akt signaling pathway	CDK2, IGF1R, INSR, MAPK3, PIK3CB, PIK3CG, PTK2, SGK1	8		8.77E-05
	hsa01522	Endocrine resistance	IGF1R, MAPK3, MAPK8, PIK3CB, PTK2	5	Endocrine therapies that target estrogen action (antiestrogens and aromatase inhibitors) are widely used and successful breast cancer therapies, but many women treated with these therapies will relapse with endocrine-resistant disease [67].	1.20E-04
	hsa05203	Viral carcinogenesis	CCNA2, CDK2, DLG1, HDAC1, MAPK3, PIK3CB	6	Virus-associated cancer refers to a cancer where viral infection results in the malignant transformation of the host's infected cells. Human papillomaviruses, mouse mammary tumor virus, and EBV are prime candidate viruses as agents of human breast cancer [68].	1.70E-04
	hsa04931	Insulin resistance	INSR, MAPK8, PIK3CB, RPS6KA1, SLC2A4	5	Biological markers of insulin resistance such as the insulin level, the insulin/glucose ratio, HOMA (homeostasis model assessment), adiponectin, leptin/adiponectin, and decreased SHBG (sex hormone binding globulin) have been associated with an increased risk of breast cancer essentially in postmenopausal women [69].	1.80E-04
	hsa04510	Focal adhesion	IGF1R, MAPK3, MAPK8, PIK3CB, PTK2, VAV1	6	The link between FAK and breast cancers is strongly suggested by numerous reports showing that the FAK gene is amplified and overexpressed in a large fraction of breast cancer specimens [70].	2.10E-04
	hsa05205	Proteoglycans in cancer	ANK3, IGF1R, MAPK3, PIK3CB, PTK2, VAV1	6	Proteoglycan biosynthesis is dysregulated in breast cancer, and targeting proteoglycans may provide new therapeutic approaches for breast cancer [71].	2.10E-04
	hsa04910	Insulin signaling pathway	INSR, MAPK3, MAPK8, PIK3CB, SLC2A4	5	Insulin receptor is present in many malignant cells, including breast cancer cells, and insulin may be involved in the growth of these malignancies [72].	3.70E-04
	hsa04012	ErbB signaling pathway	MAPK3, MAPK8, PIK3CB, PTK2	4	Activation of ErbB signaling can regulate EMT-associated invasion and migration in normal and malignant mammary epithelial cells, as well as modulating discrete stages of mammary gland development [73].	7.80E-04
**Reactome**	HSA-3108232	SUMO E3 ligases SUMOylate target proteins	CHD3, HDAC1, MDC1, NR3C1, SIN3A, SP3, SUMO1, TOP2A, TP53BP1	9	Among the proteins involved in SUMOylation, the protein inhibitor of activated STAT (PIAS) E3-ligases was initially described as a transcriptional coregulator. Several components of the SUMO machinery are highly expressed in breast cancer, suggesting that SUMOylation is required to initiate or sustain tumorigenesis [74].	1.39E-06
	HSA-3700989	Transcriptional regulation by tp53	CCNA2, CCNB1, CDK2, CDK5, CHD3, HDAC1, MDC1, SGK1, SUPT5H, TAF1	10	The tumor suppressor TP53 (or P53) is a well-known gene that drives multiple cancers. Under stress conditions, it regulates the transcription of many genes involved in various cellular processes such as cellular metabolism, survival, senescence, apoptosis, and DNA damage response [53, 54].	2.53E-05
	HSA-212436	Generic transcription pathway	CCNA2, CCNB1, CDK2, CDK5, CHD3, GLI3, HDAC1, MAPK3, MDC1, NR3C1, SGK1, SIN3A, SMARCA2, SUMO1, SUPT5H	16	One or more latent cytoplasmic transcription factors have increased activity in most human cancers and in many cases prevent apoptosis of cancer cells. Necessary physical interaction among transcription factors and cofactors in the nucleus affords selective sites of potential drug action [75].	2.53E-05
	HSA-74160	Gene expression (transcription)	CCNA2, CCNB1, CDK2, CDK5, CHD3, ERCC6, GLI3, HDAC1, MAPK3, MDC1, NR3C1, SGK1, SIN3A, SMARCA2, SUMO1, SUPT5H	17		3.17E-05
	HSA-9006934	Signaling by receptor tyrosine kinases	AXL, CDK5, CTNNA1, IGF1R, INSR, MAPK3, PIK3CB, PTK2, RPS6KA1, SGK1, VAV1	11	High levels of RTKs may be associated with increased breast cancer aggressiveness and decreased overall and disease-free survival [55–60].	2.82E-05
	HSA-73894	DNA repair	APEX1, CCNA2, CDK2, ERCC6, MAPK8, MDC1, PRKDC, SUMO1, TP53BP1	9	Defective components in DNA damage and repair machinery are an underlying cause for the development and progression of different types of cancers, and breast cancer is no exception [38].	3.58E-05
	HSA-5693532	DNA double-strand break repair	CCNA2, CDK2, MAPK8, MDC1, PRCKDC, SUMO1,TP53BP1	7	DNA double-strand break repair dysfunction increases the risk of familial and sporadic breast cancer [76].	3.83E-05
	HSA-5633007	Regulation of TP53 activity	CCNA2, CDK2, CDK5, CHD3, HDAC1, SGK1, TAF1	7	Similar to the function of transcriptional regulation by TP53 above.	4.19E-05
	HSA-1640170	Cell cycle	CCNA2, CCNB1, CDC14A, CDK2, HDAC1, MAPK3, MDC1, POLA1, TOP2A, TP53BP1, SUMO1	11	Similar to the function of cell cycle in KEGG.	1.30E-04
	HSA-453279	Mitotic G1 phase and G1/S transition	CCNA2, CCNB1, CDK2, HDAC1, POLA1, TOP2A	6	Estrogen-induced mitochondrial oxidants control the early stages of cell cycle progression, which provides the basis for the discovery of new antioxidant-based drugs or antioxidant gene therapies to prevent and treat estrogen-dependent breast cancer [77].	3.30E-04
	HSA-4420097	VEGFA–VEGFR2 pathway	AXL, CTNNA1, PIK3CB, PTK2, VAV1	5	A study suggests that ACE2, a potential resister of breast cancer, may inhibit breast cancer angiogenesis through the VEGFa/VEGFR2/ERK pathway [78].	6.90E-04
	HSA-1280215	Cytokine signaling in the immune system	CD4, HSPA8, HSPA9, MAPK3, MAPK8, PIK3CB, RPS6KA1, SUMO1, TYK2, VAV1	10	One study found dysregulated cytokine signaling in peripheral blood T cells from patients with BC, even those with localized disease [79].	9.00E-04
	HSA-422475	Axon guidance	ANK3, CDK5, DLG1, HSPA8, MAPK3, MAPK8, PIK3CB, PTK2, RPS6KA1	9	The frequent dysregulation of axon guidance molecule (AGM) expression during tumorigenesis and tumor progression suggests that AGMs also play a crucial role as tumor suppressors and oncogenes in breast cancer [80].	9.70E-04

##### Noncancer disease level

In addition to the “cancer-type level” analysis discussed above, we found in the “noncancer disease level” analysis that 10 of the 51 driver candidates were enriched in 3 BC-related diseases, including type 2 diabetes, obesity, and plasma high-density lipoprotein cholesterol (HDL-C) (see details in Supplementary Table S2). Reportedly, up to 16% of patients with BC have diabetes, and type 2 diabetes may be associated with a 10% to 20% increased relative risk of BC [61]. In addition, a recent study suggested that type 2 diabetes accelerates the paracrine effects of AT-MSCs (adipose tissue-derived mesenchymal stem cells) to induce the migration of breast cancer cells (BCCs) and upregulate migration and EMT-related factors in BCCs [62]. Obesity has long been recognized as an important determinant of the progression of BC and mortality [81]. In fact, obesity increases the risk of cancer recurrence and death and, in particular, accelerates and exacerbates the metastatic progression of breast cancers [82–87]. Moreover, patients with breast cancers and obesity are up to 46% more likely to have distant metastases 10 years after diagnosis [83]. Several studies have shown that HDL-C has anti-inflammatory properties that may be associated with an increased risk of breast cancer [35, 88–93].

##### Gene level

We analyzed the homology between the 51 driver candidates and the genes in CGC, and a total of 23 (45.1%) genes have homologs in CGC, which are shown in Table 4. In particular, homologs AKT1 [94], AKT2 [95], ERBB2 [96], FGFR2 [97], and PIK3CA [98] in CGC play important roles in breast cancer progression, mediating breast cancer risk, corresponding to the driver candidates RPS6KA1, SGK1, PTK2, IGF1R, PIK3CB, and PRKDC predicted by DriverMP.

**Table 4:** Twenty-three driver candidates of “gene level” of breast cancer

Gene	NCBI Entrez ID	CGC [gene (sequence similarity/E_value)]
AXL	558	MET (41.195/9.58E-68), ABL1 (44.086/1.57E-62), FGFR1 (40.122/6.05E-61), ABL2 (42.806/1.52E-60), FGFR3 (40.136/1.35E-59), KDR (40.187/1.30E-41), PDGFRB (41.718/1.17E-35)
CACNA1A	773	CACNA1D (51.124/0)
CDK2	1017	CDK6 (48.495/4.54E-93), CDK4 (45.424/8.14E-82), CDK12 (43.934/7.16E-74)
CDK5	1020	CDK6 (45.638/6.70E-81), CDK4 (44.108/1.67E-74), CDK12 (40.728/1.60E-67)
CHD3	1107	CHD4 (71.161/0), TRIM24 (49.091/8.86E-12), KDM5C (56.522/4.58E-11), TRIM33 (40.678/4.34E-10), KDM5A (52.174/2.22E-10), NSD3 (54.348/3.76E-10), NSD2 (47.17/1.04E-06)
DLG1	1739	PTPN13 (46.875/5.21E-16), GOPC (44.186/1.32E-08)
GLI3	2737	WT1 (40.164/2.43E-21), ZNF331 (40.94/3.47E-19), BCL6 (43.956/5.42E-11), BCL5 (43.956/5.42E-11), KLF6 (40.789/4.53E-14), BCL11B (47.059/7.70E-07), BCL11A (47.059/1.07E-06), SALL4 (41.176/1.19E-05)
IGF1R	3480	ROS1 (40.302/1.30E-73), NTRK1 (41.892/2.27E-71), FGFR3 (40.21/5.94E-65), FGFR2 (40.351/1.35E-64), DDR2 (40.127/1.01E-58), ABL2 (41.606/1.51E-57), PDGFRA (40.462/8.93E-36), PDGFRB (41.176/2.73E-34)
INSR	3643	ROS1 (49.64/2.50E-73), NTRK1 (42.568/7.32E-69), FGFR3 (40.702/1.05E-63), MET (42.105/2.40E-57), ABL1 (40.293/4.29E-57), KIT (42.038/1.84E-36), PDGFRA (41.714/8.88E-36), PDGFRB (40.667/1.35E-32)
MAPK3	5595	MAPK1 (88.15/0)
MAPK8	5599	MAPK1 (42.735/4.93E-86)
NEB	4703	LASP1 (72.464/6.43E-27), ABI1 (43.396/8.24E-06)
NR3C1	2908	AR (51.752/5.44E-125)
PIK3CB	5291	PIK3CA (40.796/0)
PRKDC	5591	PIK3CA (42.424/2.61E-06)
PTK2	5747	ABL1 (40.214/1.47E-63), ABL2 (40.58/1.32E-61), SRC (41.288/7.18E-59), ERBB2 (40.074/1.74E-55), FLT4 (40.331/8.14E-40), KDR (41.714/1.84E-38), FLT3 (40.909/7.65E-35), PDGFRB (40.385/6.40E-31)
RPS6KA1	6195	AKT1 (42.857/3.61E-89), PRKACA (41.16/1.09E-79)
SGK1	6446	AKT2 (48.837/6.11E-114), AKT1 (46.011/2.94E-112)
SMARCA1	6594	SMARCA4 (41.679/7.78E-146)
SMARCA2	6595	SMARCA4 (79.037/0), CHD4 (40.262/6.65E-109)
SP3	6670	KLF6 (53.608/6.60E-31), KLF4 (52.632/2.03E-29), WT1 (44.737/1.71E-27), PRDM1 (43.333/9.13E-17), PATZ1 (41.284/1.31E-16), MECOM (42.623/1.29E-09), ZNF521 (42.5/3.86E-15), ZBTB16 (42.105/4.40E-15), PRDM16 (42.623/1.03E-09), PLAG1 (41.25/3.31E-14), ZNF331 (45/6.61E-13), IKZF1 (43.75/2.43E-12), SALL4 (50/1.31E-08), BCL11B (42.308/7.90E-08), BCL11A (42.105/1.29E-07)
TAF1	6872	TRIM24 (40.625/4.08E-08), TRIM33 (40.678/1.37E-06)
TYK2	7297	JAK1 (46.814/0)

##### Validation-required level

Five genes were identified at this level. The 5 genes were ranked high according to DriverMP, but their direct relationship with BC was unclear based on this study. Future investigations may reveal the associations of these genes with breast cancers.

To visualize the relationship among the 5 levels of candidate drivers, we drew a Venn graph and found that the IGF1R gene appeared in all these groups (Fig. 6D). Based on the specificity of IGF1R, we reviewed the literature and found that examinations of various tumors showed abundant expression of IGF1R, which suggests that upregulation of the IGF1R gene constitutes a common paradigm in different types of cancer [99–101]. Some experimental studies have also suggested that the IGF1R gene is a downstream target of the BRCA1 gene [102–104]. This evidence indicates that IGF1R is highly likely to be a typical driver of BC.

#### DriverMP reliably predicts novel drivers for lung adenocarcinoma

Lung cancer is the top cause of cancer-related death and is histologically classified into small cell lung cancer (SCLC) and non–small cell lung cancer (NSCLC) [105]. NSCLC accounts for approximately 85% of all lung cancer diagnoses, with the majority of patients presenting with lung adenocarcinoma (LUAD) [106]. In this section, we collected 60 novel driver candidates for lung adenocarcinoma and grouped them according to the 6 analysis levels (Table 5).

**Table 5:** Six-level assessment yielded 60 driver candidates of lung adenocarcinoma

No.	Level of analysis	Driver candidates	Count	Percentage
1	Cancer-type level	ABCB1, BARD1, CASP3, CCNA2, CDC25C, CHEK1, ERCC6, IGF1R, IRS1, PLK1, PRKDC, STAT1, SUMO1, TP53BP1	14	23.33
2	Literature-supported level	BARD1, CDC25C, CHEK1, HDAC1, IRS1, KAT2B, LYN, MDC1, PLK1, PRKCB, SP1, SUMO1, TLR4, TOP2A, USP7	15	25.00
3	Pathway level	ABCB1, ACTB, ANK2, BARD1, CASP1, CASP3, CCNA2, CDC14A, CDC25C, CHD3, CHEK1, CSNK1D, CSNK2A1, E2F4, EGR1, ERCC6, FLT1, GAPDH, GLI2, GLI3, GRB2, HDAC1, HSPA8, IGF1R, IRS1, KAT2B, LYN, MAPK9, MDC1, MED1, NR3C1, PIK3CG, PLK1, POLA1, PPP3CA, PRKCA, PRKCB, PRKDC, PTK2B, SIN3A, SMARCA2, SOS1, SP1, STAT1, SUMO1, TAF1, TLR4, TNC, TOP2A, TOP2B, TP53BP1, USP7, VCAN	53	88.33
4	Noncancer disease level	ABCB1, BARD1, CASP3, CCNA2, CDC25C, CHEK1, ERCC6, IGF1R, IRS1, PLK1, STAT1, TLR4	12	20.00
5	Gene level	BARD1, CHD3, EGR1, FLT1, GLI2, GLI3, GRB2, HOXA5, IGF1R, LYN, MAPK9, NEB, NR3C1, PPP3CA, PRKCA, PRKCB, PRKDC, PTK2B, SMARCA2, SP1, STAT1, TAF1, VCAN	23	38.33
6	Validation-required level	CAD, HSPA5, RYR2, RYR1, TTN	5	8.33

##### Cancer-type and noncancer disease levels

**Figure 7:**
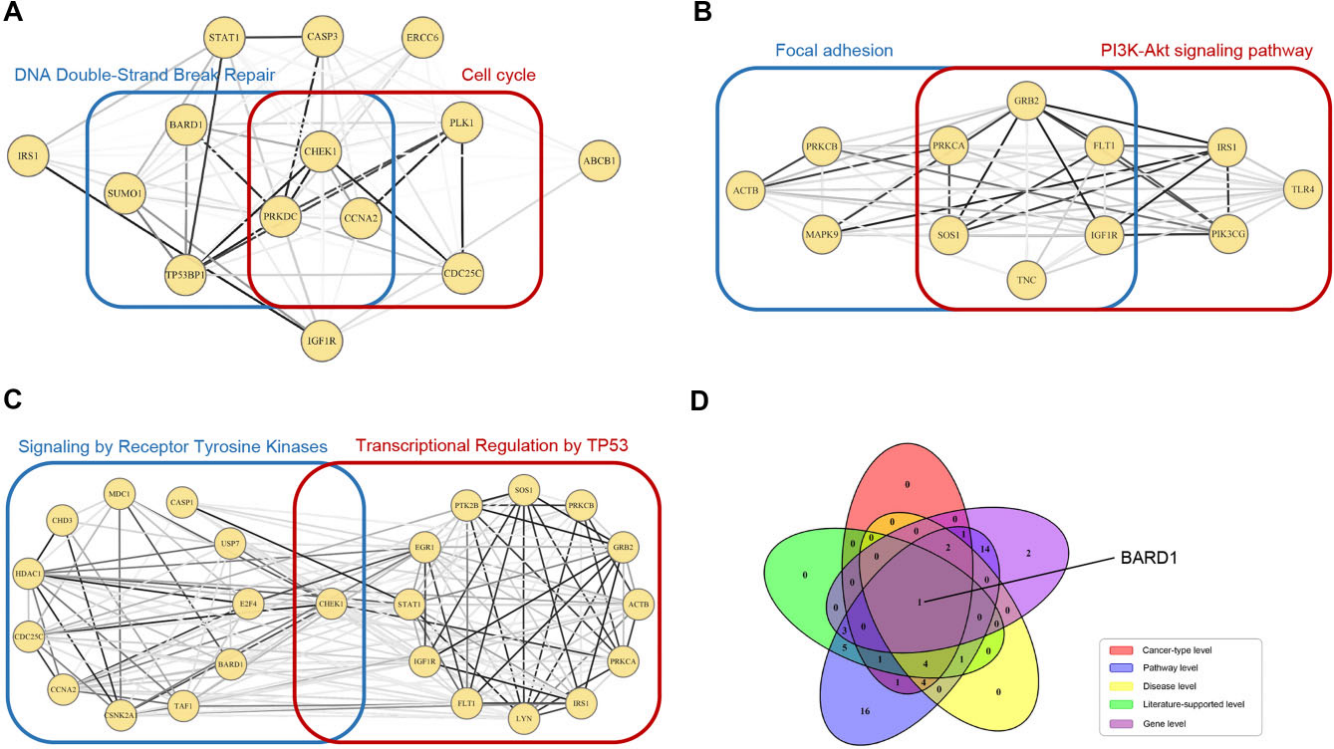
Six-level assessment of 60 driver candidates of lung adenocarcinoma. (A) Subnetwork of the genes of the “cancer-type level” from the STRINGv10 network. The 5 genes in the blue box are enriched in the “cell cycle” pathway (KEGG), and the 6 genes in the red box are enriched in “DNA double-strand break repair” pathway (Reactome). (B) Relationship of the genes enriched in the 2 biological pathways in the KEGG analysis of the STRINGv10 network. (C) Relationship of the genes enriched in the 2 biological pathways in the Reactome analysis of the STRINGv10 network. (D) Venn diagram of driver candidates for the 5 levels of lung adenocarcinoma.

Similar to the process in breast cancer analysis, disease enrichment showed that 47 (78.3%) of these 60 candidates were included in “cancer” ($P = 2.6\ \times {10}^{ - 18},\ FDR = 4.7\ \times {10}^{ - 17}$), and 14 (23.3%) and 12 (20.0%) of them were enriched in “lung cancer” ($P = 3.5\ \times {10}^{ - 7},\ FDR = 6.5\ \times {10}^{ - 5}$) and “chronic obstructive pulmonary disease” ($P = 1.0\ \times {10}^{ - 6},\ FDR = 1.7\ \times {10}^{ - 4}$), respectively (Fig. 7A), which comprise the candidate genes at the “cancer-type level” and “noncancer disease level” (see details in Supplementary Table S3).

For the cancer-type level analysis, we found that 5 of the candidate genes were enriched in the “cell cycle” pathway (KEGG, $FDR = 6.3\ \times {10}^{ - 6}$), and 6 were enriched in the “DNA double-strand break repair” pathway (Reactome, $FDR = 7.0\ \times {10}^{ - 7}$), both of which are associated with cancer progression (Fig. 7A). Regarding the noncancer disease-level analysis, it has been reported that people with chronic obstructive pulmonary disease (COPD) are at higher risk of developing lung cancer than those without COPD and that these patients are more susceptible to poor outcomes after diagnosis and treatment; COPD could be the driving factor for lung cancer, providing a conducive environment for cell propagation and evolution [107, 108].

##### Literature-supported candidate drivers

This level of analysis yielded 15 genes supported by 3 different types of evidence, as shown in Table 6. For example, PLK1 is a key mitotic kinase that is overexpressed in various cancers, including NSCLC, and drives cancer growth [109, 110]. Sp1 levels accumulate strongly in the early stage and then decline in the late stage, which is important for lung cancer cell proliferation and metastasis during tumorigenesis [111].

**Table 6:** Fifteen driver candidates identified in the “literature-supported level” of analysis of lung adenocarcinoma

Gene	NCBI Entrez ID	Rank (HumanNet)	Rank (STRINGv10)	Function	Type
CHEK1	1111	31	179	Recently, integrated bioinformatics analysis showed that CHEK1 may be a critical gene in the development and prognosis of NSCLC. In addition, it has been suggested that CHEK1 expression was increased in NSCLC, compared with adjacent normal tissues [112, 113].	O
PLK1	5347	42	50	Polo-like kinase 1 (PLK1) is a critical mitotic kinase that is overexpressed in various cancers, including NSCLC, and drives cancer growth [109, 110].	E&B
CDC25C	995	80	213	CDC25C may predict poor prognosis and may have important roles in the regulation of S-phase and M/G1 phase of the cell cycle as well as the FAS-mediated apoptosis in LUAD [114].	B
IRS1	3667	113	169	Recent studies have analyzed the biological impact of newly identified mutations within the IRS1 gene and suggested that these mutations may be diagnostic markers for lung cancer [115].	E
SUMO1	7341	139	192	SUMO1 promotes the proliferation and invasion of NSCLC cells by regulating NF-κB[116].	E
BARD1	580	244	191	BARD1 isoforms might be involved in tumor initiation and invasive progression and might represent a novel prognostic marker for NSCLC [117].	E
KAT2B	8850	30	72	Recent study has demonstrated that KAT2B expression positively correlated with the outcomes of patients with lung adenocarcinoma. Additionally, KAT2B was synergistic with multiple immune cell infiltration and immune checkpoints in NSCLC [118].	B
USP7	7874	32	74	Intervention in USP7 to induce the downregulation of Ki-67 protein could inhibit proliferation of NSCLC cells and even increase the sensitivity of the cells to some chemotherapy drugs [119].	E
TOP2A	7153	52	91	Study revealed that TOP2A was highly expressed in lung adenocarcinoma compared with matched adjacent normal tissues, and high expression of TOP2A was associated with poor prognosis for patients with LUAD [120].	E&B
SP1	6667	69	163	Sp1 level accumulated strongly in early stage and then declined in late stage, which is important for lung cancer cell proliferation and metastasis during tumorigenesis [111].	E
TLR4	7099	75	29	TLR4 is highly expressed in NSCLC tumor cells and strongly correlates with malignant tumor phenotypes. TLR4 ligation promotes the secretion of immunosuppressive cytokines TGF-β, VEGF, and proangiogenic chemokine IL-8 from human lung cancer cells [121, 122].	E
LYN	4067	145	98	Lyn regulates activation of epidermal growth factor receptors in lung adenocarcinoma cells. Specifically, Lyn is involved in the EGFR signaling pathway, and inhibition of its expression can reduce EGFR activation and cell viability [123].	E
PRKCB	5579	155	107	Recent study showed that PRKCB is relevant to prognosis of LUAD through methylation and immune infiltration [124].	B
HDAC1	3065	170	176	The results in a meta-analysis suggest that HDAC1 may serve as a good diagnostic and prognostic marker for lung cancer [125].	B
MDC1	9656	208	83	MDC1 plays important roles in tumor formation, progression, and treatment. In addition, MDC1 expression was assessed by immunohistochemistry in lung tumors and was found to be commonly expressed in benign tissues but reduced or lost in 26% of lung cancer samples [126, 127].	O

##### Pathway level

In total, 53 of the 60 driver candidates were identified as pathway-related driver candidates. We performed a pathway enrichment analysis on these 60 driver candidates and identified 16 and 11 pathways associated with lung adenocarcinoma or NSCLC in KEGG and Reactome, respectively (Table 7). For instance, we found that 9 (15.0%) and 9 (15.0%) of the 60 candidates were enriched in “focal adhesion” ($FDR = 6.5\ \times {10}^{ - 7}$) and “PI3K-Akt signaling pathway” ($FDR = 1.7\ \times {10}^{ - 5}$) against KEGG (Fig. 7B), respectively, both of which are highly related to LUAD. Several studies have suggested that focal adhesion kinase (FAK) expression is frequently upregulated in different types of cancer, including NSCLC, and a number of studies have focused on either reducing FAK expression or activity to inhibit the growth and metastatic capacities of tumors [128, 129]. The PI3K/AKT pathway has been reported as an emerging source of lung cancer aggressiveness [130]. We also found that 13 (21.7%) and 12 (20.0%) of the 60 genes were enriched in “signaling by receptor tyrosine kinases” ($FDR = 8.5\ \times {10}^{ - 7}$) and “transcriptional regulation by TP53” ($FDR = 3.8\ \times {10}^{ - 5}$), respectively, according to the Reactome database (Fig. 7C). RTKs such as EGFR, the classical driver of lung cancer, are important components of the cellular signaling apparatus and are frequently mutated or otherwise dysregulated in NSCLC [131]. The function of the “transcriptional regulation by TP53” pathway has already been discussed in the analysis of breast cancer. As described before, TP53 (or P53) is a well-known gene that drives multiple cancers, including lung adenocarcinoma [53].

**Table 7:** Biological pathways associated with lung adenocarcinoma.

Database	Pathway ID	Pathway	Genes	Count	Function	FDR
**KEGG**	hsa05206	MicroRNAs in cancer	ABCB1, CASP3, CDC25C, GRB2, HDAC1, IRS1, PRKCA, PRKCB, SOS1, TNC	10	Dysregulation of microRNA expression often appears in many cancers such as lung cancer, breast cancer, and cervical cancer and is directly associated with tumor initiation, progression, and metastasis [132].	3.00E-08
	hsa04110	Cell cycle	CCNA2, CDC14A, CDC25C, CHEK1, E2F4, HDAC1, PLK1, PRKDC	8	Cell cycle deregulation is a common feature of human cancer. Cancer cells frequently display uncontrolled proliferation, genomic instability (increased DNA mutations and chromosomal aberrations), and chromosomal instability (changes in chromosome number) [133].	5.32E-07
	hsa04510	Focal adhesion	ACTB, FLT1, GRB2, IGF1R, MAPK9, PRKCA, PRKCB, SOS1, TNC	9	Several studies have suggested that FAK is frequently upregulated in different types of cancer, including NSCLC, and a great number of studies have focused on reducing FAK expression or activity to inhibit the growth and metastatic capacities of tumors [128, 129].	6.48E-07
	hsa04912	GnRH signaling pathway	EGR1, GRB2, MAPK9, PRKCA, PRKCB, PTK2B, SOS1	7	GnRH and GnRH-R are expressed in several types of cancer tissues, including NSCLC, indicating that the expression of GnRH may be associated with tumor progression [134].	6.78E-07
	hsa04010	MAPK signaling pathway	CASP3, FLT1, GRB2, HSPA8, IGF1R, MAPK9, PPP3CA, PRKCA, PRKCB	10	MAPK pathway affects decisive roles in the carcinogenesis and treatment resistance of NSCLC cells by promoting proliferation or inhibiting apoptosis of NSCLC cells [135].	7.26E-07
	hsa04935	Growth hormone synthesis, secretion and action	GRB2, IRS1, MAPK9, PRKCA, PRKCB, SOS1, STAT1	7	Both humans and mice lacking functional growth hormone receptors are known to be resistant to cancer. A growth hormone receptor SNP promotes lung cancer by impairment of SOCS2-mediated degradation [136].	3.20E-06
	hsa04650	Natural killer (NK) cell–mediated cytotoxicity	CASP3, CCNA2, CHEK1, GRB2, HDAC1, KAT2B, LYN, USP7	8	CD48-positive NSCLC cells might be susceptible to NK cell–mediated cytotoxicity, which provide information on how to stratify patients with NSCLC potentially responsive to NK-cell therapy [137].	3.20E-06
	hsa04066	HIF-1 signaling pathway	FLT1, GAPDH, IGF1R, PRKCA, PRKCB, TLR4	6	CD39/CD73 upregulation on myeloid-derived suppressor cells via TGF-β–mTOR–HIF-1 signaling in patients with NSCLC [138].	1.58E-05
	hsa04151	PI3K-Akt signaling pathway	FLT1, GRB2, IGF1R, IRS1, PIK3CG, PRKCA, SOS1, TLR4, TNC	9	Currently, PI3K/AKT/mTOR signaling has been reported as an emerging source of lung cancer aggressiveness. The development of therapies targeting PI3K/AKT/mTOR signaling is receiving extensive attention from researchers, and new drugs continue to be discovered [130].	1.74E-05
	hsa04150	mTOR signaling pathway	GRB2, IGF1R, IRS1, PRKCA, PRKCB, SOS1	6		7.58E-05
	hsa04062	Chemokine signaling pathway	GRB2, LYN, PIK3CG, PRKCB, PTK2B, SOS1, STAT1	7	Axl highly expressing lung adenocarcinomas exhibit higher expressions of multiple genes encoding immune checkpoint molecules and chemokines/chemokine receptors [139].	2.38E-05
	hsa01521	Epidermal growth factor receptor (EGFR) tyrosine kinase inhibitor resistance	GRB2, IGF1R, PRKCA, PRKCB, SOS1	5	Patients with NSCLC with activating EGFR mutations typically benefit from EGFR tyrosine kinase inhibitor treatment [140].	5.55E-05
	hsa04014	Ras signaling pathway	FLT1, GRB2, IGF1R, MAPK9, PRKCA, PRKCB, SOS1	7	The Ras proteins are pivotal regulators of cellular proliferation, differentiation, motility, and apoptosis. Mutations on the K-ras gene have been found in 20–30% of NSCLCs and are believed to play a key role in this malignancy [141].	6.60E-05
	hsa04012	ErbB signaling pathway	GRB2, MAPK9, PRKCA, PRKCB, SOS1	5	Evidence is now accruing that EGFR works in concert with other ErbB family members, particularly HER2 and ErbB3, to activate these signaling pathways in lung cancers [142].	6.78E-05
	hsa05223	NSCLC	GRB2, PRKCA, PRKCB, SOS1	4	NSCLC accounts for approximately 85% of all lung cancer diagnoses, with the majority of patients presenting with lung adenocarcinoma [105, 106].	4.60E-04
	hsa04310	Wnt signaling pathway	CSNK2A1, MAPK9, PPP3CA, PRKCA, PRKCB	5	Available data indicate that Wnt signaling substantially impacts NSCLC tumorigenesis, prognosis, and resistance to therapy, with loss of Wnt signaling inhibitors by promoter hypermethylation or other mechanisms appearing to be particularly important [143].	7.60E-04
**Reactome**	HSA-1640170	Cell cycle	BARD1, CCNA2, CDC14A, CDC25C, CHEK1, CSNK1D, CSNK2A1, E2F4, HDAC1, LYN, MDC1, POLA1, PRKCA, PRKCB, PLK1, SUMO1, TOP2A, TP53BP1	18	Similar to the function of the cell cycle KEGG pathway.	6.05E-10
	HSA-69278	Cell cycle, mitotic	CCNA2, CDC14A, CDC25C, CSNK1D, CSNK2A1, E2F4, HDAC1, LYN, PLK1, POLA1, PRKCA, PRKCB, SUMO1, TOP2A	14		1.95E-07
	HSA-212436	Generic transcription pathway	BARD1, CASP1, CCNA2, CDC25C, CHD3, CHEK1, CSNK2A1, E2F4, GLI2, GLI3, HDAC1, KAT2B, MDC1, MED1, NR3C1, PRKCB, SIN3A, SMARCA2, SP1, STAT1, SUMO1, TAF1, USP7	23	Mutated or dysregulated transcription factors represent a unique class of drug targets that mediate aberrant gene expression, including blockade of differentiation and cell death gene expression programs, hallmark properties of cancers [144].	6.05E-10
	HSA-162582	Gene expression (transcription)	ACTB, BARD1, CASP1, CCNA2, CDC25C, CHD3, CHEK1, CSNK2A1, E2F4, ERCC6, GLI2, GLI3, HDAC1, KAT2B, MDC1, MED1, NR3C1, PRKCB, SIN3A, SMARCA2, SP1, STAT1, SUMO1, TAF1, USP7	25		6.05E-10
	HSA-3700989	Transcriptional regulation by TP53	BARD1, CASP1, CCNA2, CDC25C, CHD3, CHEK1, CSNK2A1, E2F4, HDAC1, MDC1, TAF1, USP7	12	The function of this pathway has been mentioned in the section on breast cancer.	3.78E-07
	HSA-597592	Posttranslational protein modification	ACTB, ANK2, BARD1, CCNA2, CHD3, CSNK1D, HDAC1, HSPA8, KAT2B, MDC1, NR3C1, PRKDC, SIN3A, SUMO1, TNC, TOP2A, TOP2B, TP53BP1, USP7, VCAN	20	In the past few years, several therapeutic design options focusing on specific kinases or phosphatases dysregulated in lung cancer progression have been developed with various levels of success [145].	7.87E-07
	HSA-9006934	Signaling by receptor tyrosine kinases	ACTB, CHEK1, EGR1, FLT1, GRB2, IGF1R, IRS1, LYN, PRKCA, PRKCB, PTK2B, SOS1, STAT1	13	RTKs are important components of the cellular signaling apparatus and are frequently mutated or otherwise dysregulated in NSCLC, such as EGFR, the classical driver of lung cancer [131].	8.47E-07
	HSA-73894	DNA repair	ACTB, BARD1, CCNA2, CHEK1, ERCC6, MDC1, PRKDC, SUMO1, TP53BP1, USP7	10	DNA repair pathways can enable tumor cells to survive DNA damage that is induced by chemotherapeutic treatments; therefore, inhibitors of specific DNA repair pathways might prove efficacious when used in combination with DNA-damaging chemotherapeutic drugs [146].	6.82E-06
	HSA-1433559	Regulation of KIT signaling	GRB2, LYN, PRKCA, SOS1	4	miR-1260b, mediated by YY1, activates KIT signaling by targeting SOCS6 to regulate NSCLC cell proliferation and apoptosis and is a potential biomarker and therapeutic target for NSCLC [147].	4.45E-05
	HSA-1280215	Cytokine signaling in the immune system	CASP1, CASP3, EGR1, GRB2, HSPA8, IRS1, LYN, MAPK9, PTK2B, SOS1, STAT1, SUMO1	12	TLR4 expressed on human lung cancer cells is functionally active and may play important roles in promoting immune escape of human lung cancer cells by inducing immunosuppressive cytokines and apoptosis resistance [121].	8.90E-05
	HSA-194138	Signaling by VEGF	ACTB, FLT1, PTK2B, PRKCA, PRKCB	5	In lung cancer, VEGF plays a significant role in establishing a vascular supply within the tumor [148].	9.40E-04

##### Gene level

We also analyzed the homology between the 60 driver candidates and the genes in CGC, and a total of 23 (38.33%) genes have homologs in CGC, which are shown in Table 8. In particular, FLT1 predicted by DriverMP has high sequence similarity to the KDR gene associated with lung cancer in CGC, and related studies showed that tumors expressing both FLT1 and KDR may have a greater malignant potential and a poorer prognosis [149]. In addition, SMARCA2, which has been added to the gene level, shares a sequence similarity of up to 79.04% with SMARCA4, and Tian et al. [150] claimed that SMARCA2 could be a novel therapeutic target as a key synthetic lethal target in SMARCA4-deficient cancers.

**Table 8:** Twenty-three driver candidates of “gene level” of lung adenocarcinoma

Gene	NCBI Entrez ID	CGC [gene (sequence similarity/E_value)]
BARD1	580	BCOR (40.299/8.86E-18)
CHD3	1107	CHD4 (71.161/0), TRIM24 (49.091/8.86E-12), KDM5C (56.522/4.58E-11), TRIM33 (40.678/4.34E-10), KDM5A (52.174/2.22E-10), NSD3 (54.348/3.76E-10), NSD2 (47.17/1.04E-06)
EGR1	1958	WT1 (61.053/1.47E-32), KLF6 (59.016/2.34E-19), BCL6 (41.379/5.24E-09), BCL5 (41.379/5.24E-09), ZNF331 (44.048/3.82E-17), PRDM16 (45.122/8.63E-18), ZNF521 (40.777/1.02E-17), MECOM (45.122/1.23E-17), CTCF (42.857/3.31E-13), SALL4 (49.057/7.86E-11)
FLT1	2321	KDR (44.933/0), FLT4 (40.603/0), FLT3 (40.812/2.65E-104), FGFR4 (44.857/7.29E-88), RET (42.135/3.70E-78), MET (45.604/3.68E-45), PTK6 (47.647/1.77E-40), ABL2 (44.654/3.47E-39), ROS1 (43.506/1.65E-37), ALK (42.683/1.92E-37), ABL1 (40.719/8.67E-37), EGFR (41.975/2.94E-36), FES (41.718/4.07E-36), ERBB2 (42.405/7.47E-35), SYK (40.881/3.99E-31), JAK3 (42.529/1.62E-30)
GLI2	2736	WT1 (40.164/6.23E-22), ZNF331 (40.741/1.86E-14), BCL6 (43.956/4.04E-11), BCL5 (43.956/4.04E-11), BCL11B (45.283/2.68E-07), BCL11A (45.283/3.92E-07), SALL4 (40.741/3.17E-06)
GLI3	2737	WT1 (40.164/2.43E-21), ZNF331 (40.94/3.47E-19), BCL6 (43.956/5.42E-11), BCL5 (43.956/5.42E-11), KLF6 (40.789/4.53E-14), BCL11B (47.059/7.70E-07), BCL11A (47.059/1.07E-06), SALL4 (41.176/1.19E-05)
GRB2	2885	SH3GL1 (50/2.47E-13), ABI1 (42/2.30E-09), SRGAP3 (43.396/1.43E-07), ARHGAP26 (41.509/3.79E-07)
HOXA5	3202	HOXA9 (68.333/2.48E-23), MNX1 (52.778/1.36E-21), CDX2 (53.012/5.89E-21), HOXA11 (47.945/4.71E-19), HOXD11 (51.667/1.58E-18), HOXC11 (47.541/7.85E-18), HOXA13 (49.254/2.89E-15), HOXD13 (48.438/1.35E-13)
IGF1R	3480	ROS1 (40.302/1.30E-73), NTRK1 (41.892/2.27E-71), FGFR3 (40.21/5.94E-65), FGFR2 (40.351/1.35E-64), DDR2 (40.127/1.01E-58), ABL2 (41.606/1.51E-57), PDGFRA (40.462/8.93E-36), PDGFRB (41.176/2.73E-34)
LYN	4067	LCK (67.292/0), SRC (60.271/0), ABL1 (41.648/4.59E-113), ABL2 (41.203/3.66E-112), PDGFRA (41.071/9.31E-13), PDGFRB (48.322/1.99E-39), KIT (45.161/1.99E-38), FLT3 (42.391/2.54E-38), KDR (42.012/6.19E-35)
MAPK9	5601	MAPK1 (41.274/1.00E-84)
NEB	4703	LASP1 (72.464/6.43E-27), ABI1 (43.396/8.24E-06)
NR3C1	2908	AR (51.752/5.44E-125)
PPP3CA	5530	PPP6C (41.219/1.27E-67)
PRKCA	5578	PRKACA (100/0), AKT2 (47.432/1.54E-104), AKT1 (47.436/1.49E-100)
PRKCB	5579	PRKACA (79.552/0), AKT2 (45.584/3.35E-110), AKT1 (42.785/3.16E-105)
PRKDC	5591	PIK3CA (42.424/2.61E-06)
PTK2B	2185	ROS1 (42.697/2.24E-52), FLT4 (45.342/2.22E-38), KDR (43.195/2.11E-37), KIT (40.667/3.18E-32)
SMARCA2	6595	SMARCA4 (79.037/0), CHD4 (40.262/6.65E-109)
SP1	6667	KLF6 (54.639/1.45E-31), KLF4 (52.525/1.64E-30), WT1 (47.423/2.61E-26), PRDM1 (42.222/2.96E-16), MECOM (46.296/3.81E-09), PRDM16 (46.296/2.43E-09), PATZ1 (43.333/3.33E-11), ZNF331 (44.737/1.28E-10), IKZF1 (46.97/6.42E-12), SALL4 (50/1.10E-10), CTCF (42.466/1.40E-10), BCL11B (42/3.03E-07), BCL11A (42/6.09E-07)
STAT1	6772	STAT3 (52.901/0)
TAF1	6872	TRIM24 (40.625/4.08E-08), TRIM33 (40.678/1.37E-06)
VCAN	1462	NOTCH2 (52.381/3.46E-15), NOTCH1 (49.315/1.16E-15), FAT1 (48.571/3.81E-05)

##### Validation-required level

A total of 7 genes were identified in this level of analysis, and their associations with LUAD may be revealed in future investigations.

Notably, the Venn diagram of candidate drivers at the 5 levels showed that BARD1 is present in the results for the 6-level assessment (Fig. 7D), which indicated its potential importance, while Zhang et al. [117] showed that isoforms of BARD1 might be involved in tumor initiation and invasive progression and represent a novel prognostic marker for NSCLC.

### Genes identified at the cancer-type level exhibit high densities in PPI networks

Genes identified at the cancer-type level demonstrated direct relationships with specific cancer types and had great potential to drive the development of corresponding cancers. To deeply investigate the relationships among these genes, we analyzed the density of the subnetwork of these genes from the PPI network.

By analysis, we found that the subnetwork of the genes at the cancer-type level demonstrated extremely high densities in all 10 cancer types (see Figs. 6A and 7A for breast cancer and lung adenocarcinoma and Supplemental Materials for the other 8 cancer types). Specifically, the subnetwork of the genes in the cancer-type level of breast cancer from the STRINGv10 network had 74 edges, and the sum of edge weights was 35.35. For lung adenocarcinoma, the subnetwork of the genes at the cancer-type level had 72 edges, and the sum of edge weights was 31.77. However, when the subnetwork was constructed from 14 genes of 1,000,000 random selections, the average number of edges and the average sum of edge weights were only 2.19 and 0.65, respectively, and none of the 1,000,000 selections constructed a subnetwork with a higher density. This phenomenon may be consistent with the view that “mutations in the cancer genome tend to converge in a few biological pathways” [18] and “genes acting together in various signaling and regulatory pathways and protein complexes is a prominent explanation for the heterogeneity of cancer mutations” [17].

### DriverMP: A user-friendly online service and a database of novel drivers

**Figure 8:**
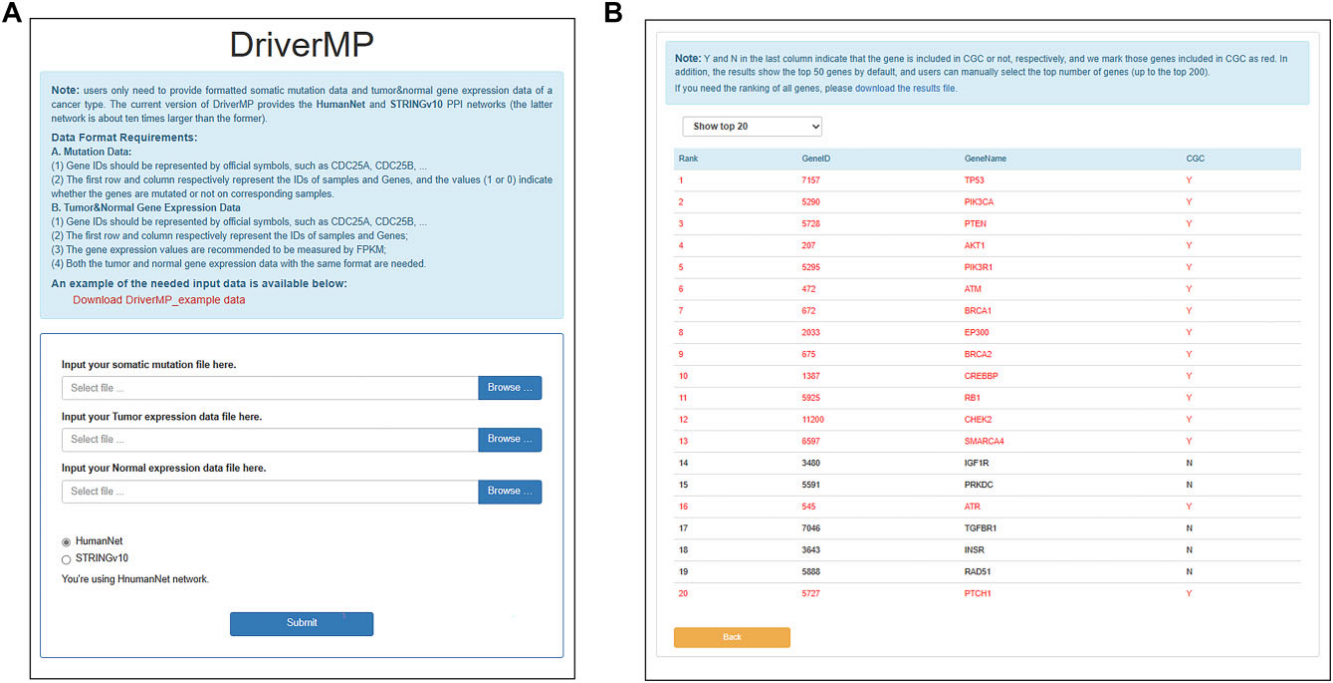
The online web server of DriverMP. (A) The interface of the online service. Users only need to submit formatted somatic mutation data and tumor and normal gene expression data, choose 1 of the 2 PPI networks (HumanNet or STRINGv10), and press the “submit” button to start DriverMP. (B) Results page. The top 50 gene candidates ranked by DriverMP are displayed by default, and users can manually choose to show the top 10, 20, 50, 100, and 200 genes. To view the full output, users can download the result file.

To make the DriverMP tool more convenient for users, we developed a user-friendly web server [151] (Fig. 8A). After entering the web server, users only need to upload 3 datasets (a mutation dataset and 2 gene expression datasets [tumor and normal]) for a cancer type and press the “submit” button to start the running of DriverMP. The current version of DriverMP provides the HumanNet and STRINGv10 PPI networks, and more PPI networks will be added in the future. After running DriverMP, the results will be directly displayed on the webpage and can also be downloaded from our page (Fig. 8B).

**Figure 9:**
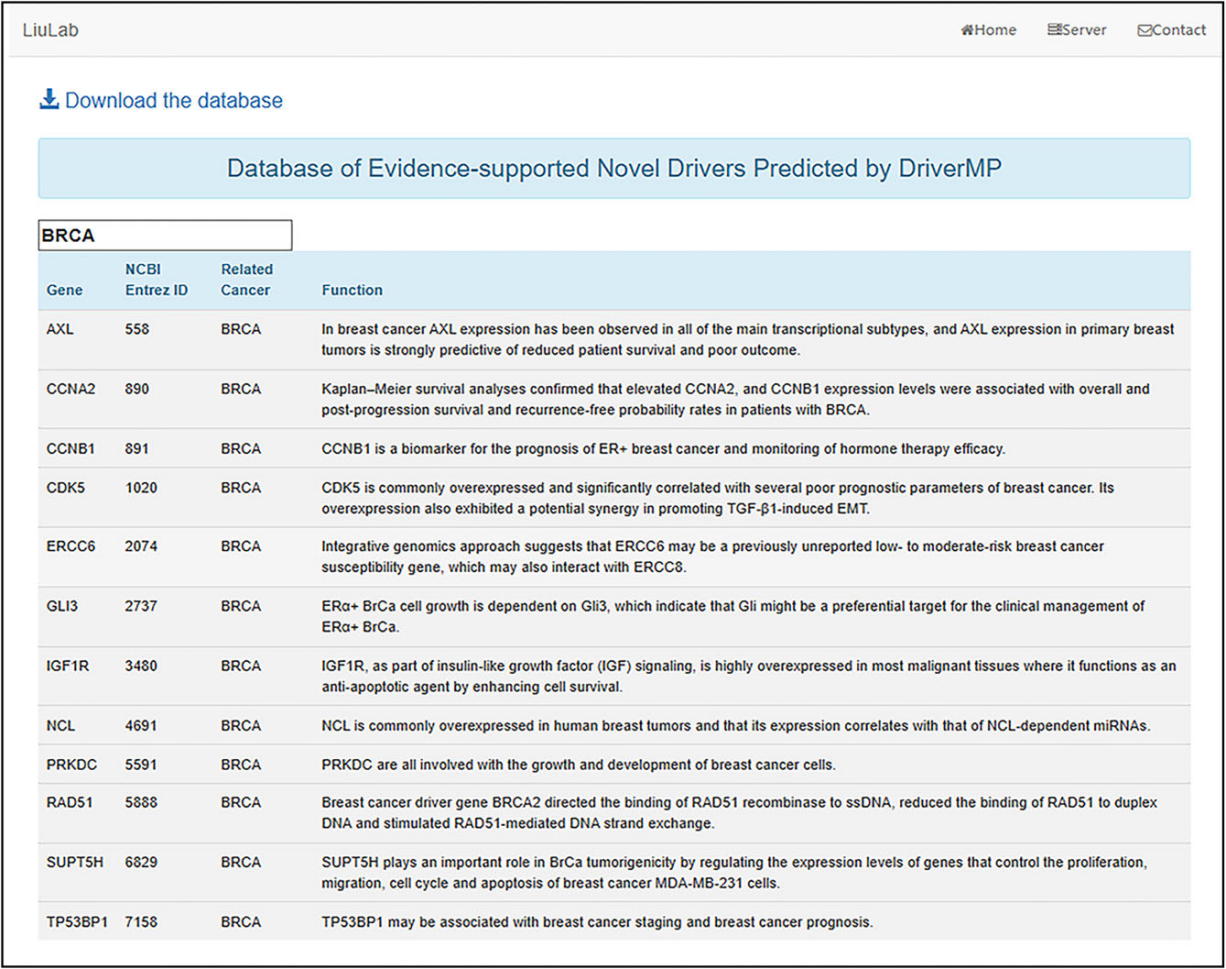
Searchable database page. We applied DriverMP to 10 different cancer types and obtained 85 driver gene candidates not included in CGC but strongly supported by related literature, based on which we built a database of reliable novel drivers.

In addition to the online web server, we developed a database [152] of those novel driver genes that are strongly supported by clinical experiments, disease enrichment analysis, or biological pathway analysis. After entering the database, users can not only search for the genes of interest to discover their related cancer types but also explore cancer types of interest to obtain novel drivers. The relationships between the genes and the corresponding cancer types are detailed in the database (Fig. 9). More cancer types will be added, and the novel drivers in the database will also be continuously updated in the future.

## Discussion

Over the past few years, it has been a major issue in cancer genetics to identify drivers from the large number of passengers. Some frequency-based computational methods have made great attempts but failed to discover the real drivers that are buried in the long tail due to the preference for highly mutated genes and the reliance on sample size. With the discovery that driver genes tend to be enriched in signaling and regulatory pathways and affect alterations in gene expression in the biological subnetworks or pathways associated with them, many algorithmic methods using PPI networks, biological pathways, or gene expression data have provided diverse solutions for the prediction of drivers, but their predictive effects, in terms of both sensitivity and specificity, are far from satisfactory.

In this study, we developed a new method, DriverMP, for the identification of driver genes by effectively integrating the advantages of multiple kinds of data analysis methods. After evaluating the performance of DriverMP and comparing it with other leading predictors, it consistently demonstrated much higher prediction accuracy based on the benchmark reference. Moreover, DriverMP demonstrated strong robustness in identifying drivers present at low frequencies even when many samples were removed, a finding that greatly contributes to overcoming the challenge of the long-tail dilemma. For the top prioritized candidates (termed novel driver candidates) identified by DriverMP that are not included in the reference, we proposed a cancer-specific 6-level assessment method to comprehensively evaluate the reliability of the novel driver candidates by applying various analytical approaches, such as disease enrichment analysis, literature investigation, and biological pathway enrichment analysis. We also provide a catalog of potentially novel driver genes with high ranks according to DriverMP and strong literature evidence for 10 common cancer types on our webpage, which provide a reference for future studies on cancer mechanisms. Moreover, we offer a user-friendly online web service for researchers to analyze their own data using DriverMP. The superiority of DriverMP may be attributed to the following innovations.

First, based on our observation that most driver genes have codriver neighbors in the PPI network, we pioneered a method for the prediction of drivers based on gene pairs, which is one of the key innovations and theoretically captures the cancer-driven characteristics in a more realistic pattern. Second, we redefined a mutation score for each gene by normalizing the mutation matrix according to the mutation frequency of each gene, based on which the contributions of different samples with different numbers of mutated genes can be effectively balanced. Third, according to the theory that genes affected by driver genes and their neighbors are simultaneously differentially expressed, we developed a differential expression network to quantify the differential expression level of each gene and the associations between 2 neighbors. Based on the assumption that driver genes tend to converge into meaningful biological subnetworks, we extracted a subnetwork from the PPI network centered on each mutated gene pair. Then, based on the differential expression network and PPI subnetwork, an impact score for each mutated gene pair was generated by combining the network topology relationships of the mutated gene pair.

Despite the obvious advantages of DriverMP, there is still much room for further improvement. For example, the current version of DriverMP only accepts 3 data types, including nonsilent somatic mutation data, PPI network, and gene expression data. However, other types of data (e.g., DNA methylation and proteomics data) can also be effectively applied to further improve the prediction performance. In addition, the current version of DriverMP can only predict driver genes for a specific cancer type and not for a specific sample. In the future version of DriverMP, we will attempt to solve these problems, add relative functions, and make further improvements.

To our knowledge, DriverMP is the first driver gene predictor that prioritizes gene pairs by constructing 2 differential expression networks and PPI subnetworks centered on each mutated gene pair and then combining the topology relationship of the 2 networks. The tool has been developed to be user-friendly and is expected to play a crucial role in new discoveries of developments, mechanisms, diagnosis, and treatments of cancers.

## Methods

### Datasets and gold-standard set

We analyzed 10 popular cancer types from the TCGA, including breast invasive carcinoma (BRCA), prostate adenocarcinoma (PRAD), lung adenocarcinoma (LUAD), lung squamous cell carcinoma (LUSC), kidney clear cell carcinoma (KIRC), kidney renal papillary cell carcinoma (KIRP), head and neck squamous cell carcinoma (HNSC), colon adenocarcinoma/rectum adenocarcinoma esophageal carcinoma (COADREAD), uterine corpus endometrial carcinoma (UCEC), and bladder urothelial carcinoma (BLCA). The somatic nonsilent mutation data and the gene expression data were downloaded from the UCSC Browser database [153, 154] and TCGA website [155], respectively, and PPI networks generated via 2 commonly used tools, STRING (RRID:SCR_005223) [31] and HumanNet (RRID:SCR_016146) [156], were downloaded from their official websites, respectively. The edge weights (representing the interaction strength between 2 proteins) of both PPI networks were normalized to values between 0 and 1 by dividing by the largest weight, and self-loops of nodes were removed. Then, a PPI network is represented as a weighted graph *G_PPI_*.

Since the real cancer genes are unavailable for the analyzed cancer types, we collected all the known cancer driver genes from the following 5 widely used databases, the Cancer Genome Census (CGC, containing 579 genes) [157], CGCpointMut (containing 245 genes), HCD (containing 291 genes) [158], MouseMut (containing 797 genes) [159], and Rule2020 (containing 124 genes) [7], to evaluate the performance of DriverMP and all the compared approaches. We combined all the driver genes in the above databases into a “benchmark reference” containing a total of 1,391 driver genes, and then a gene is defined as a driver if it is included in the benchmark reference and a passenger otherwise.

### Evaluation criteria

The performance of a cancer gene identifier was evaluated by the following criteria. (i) The ROC analysis and AUC values for uncovering known driver genes. The ROC and AUC criteria were used to evaluate the overall sensitivity and specificity of gene prioritization. As different methods output different numbers of genes and the number of genes largely affects the ROC curves and AUC values, we normalize the number of genes by selecting top *N* genes for each method, where *N* is the smallest number of genes output by all the compared methods. When plotting an ROC curve for a method, the TPR is defined as the fraction of correctly predicted driver genes in the intersection of the top *N* genes and 1,391 benchmark reference genes. (ii) The curve of the numbers of identified known driver genes in the top-ranked 1, 2, …, and *N* candidate genes. (iii) The curve of the F1-scores in the top-ranked 1, 2, …, and *N* candidate genes and the corresponding AUFC values. In practical applications, only the top-ranked genes have a chance to be validated by the follow-up experiments. For this reason, only the top-ranked 500 candidate genes for each compared approach were selected for comparing the numbers of identified known driver genes, the F1-score, and AUFC. The definition of F1-score in the top-ranked *K* genes is displayed as follows.


\begin{eqnarray*}
F1\_score = \frac{{2\ \times Presicion \times Recall}}{{Presicion + Recall}}
\end{eqnarray*}

where the precision and recall are defined as follows:


\begin{eqnarray*}
{Precision} = \frac{{\# \left\{ {genes\,\, ranked\,\, before\,\, Kth} \right\} \cap \left\{ {known\,\, driver\,\, genes} \right\}}}{{\# \left\{ {genes\,\, ranked\,\, before\,\, Kth} \right\}}}
\end{eqnarray*}

\begin{eqnarray*}
{Recall} = \frac{{\# \left\{ {genes\,\, ranked\,\, before\,\, Kth} \right\} \cap \left\{ {known\,\, driver\,\,genes} \right\}}}{{\# \left\{ {known\,\, driver\,\,genes} \right\}}}
\end{eqnarray*}

### Calculation of mutation scores based on mutation frequency

Given a nonsilent somatic mutation matrix *A* = (*a_ij_*)*_m_*_×_*_n_* of a cancer type with *m* genes and *n* samples, the value *a_ij_* = 1 if gene *i* has at least 1 nonsilent somatic mutation in sample *j*, and *a_ij_* = 0 otherwise. According to mutation matrix *A*, the mutation frequency *N*(*i*) for each gene *i* can be directly calculated as the number of samples that have a mutation in gene *i*. However, different samples usually have different numbers of mutated genes and therefore contribute unequally to the calculation of mutation frequency. To resolve the biases of different samples, we first normalized the values of the mutation matrix *A*, based on which a mutation score *M*(*i*) was defined for each gene *i*.

(1) **Normalization of the mutation matrix**. To balance the contribution of different samples, the mutation matrix *A* was first normalized to a new matrix *A′* = (*a′_ij_*)*_m_*_×_*_n_* as follows.
  \begin{eqnarray*}
a_{ij}^{\prime} = \frac{{N\left( i \right)}}{{\mathop \sum \nolimits_{gene\ k \in G\left( j \right)} N\left( k \right)}}
\end{eqnarray*}

where *G*(*j*) represents the set of mutated genes in sample *j*. Based on the above definition, the sum of the normalized mutations for each sample is 1.

(2) **Calculation of mutation scores**. Based on the normalized mutation matrix *A′*, a mutation score *M*(*i*) for each gene *i* is defined by the following formula.
  \begin{eqnarray*}
M\left( i \right) = \mathop \sum \limits_{j = 1}^n a_{ij}^{\prime}
\end{eqnarray*}

### Data preparation and the selection of major mutated genes

(1) **Gene filtering**. A gene was removed if it satisfied at least 1 of the following conditions: (i) the gene was not expressed in at least 1 sample of the tumor or normal expression matrix, (ii) the gene was not mutated in any sample, and (iii) the gene was not covered in the PPI network. The remaining genes form a gene set *S_remain_*.
(2) **Preprocess of gene expression data**. The 2 tumor and normal expression matrices with rows and columns represent genes and samples, respectively. To quantify the differential expression between tumor and normal samples, 2 matrices are processed as follows: (i) the genes are removed if they are not contained in the set *S_remain_*, and (ii) the samples are removed if they are included in only 1 of the 2 matrices. The processed tumor and normal expression matrices *M_tumor_* and *M_normal_* have the same remaining genes and samples. Then, for each gene *i*, the differential level $\delta ( i )$ between tumor and normal expression values is calculated as follows.
  \begin{eqnarray*}
\delta \left( i \right) = \left \| {M}_{tumor}(i) - {M}_{normal}(i)\right \|_2
\end{eqnarray*}

where ${M}_{tumor}( i )$ and ${M}_{normal}( i )$ represent the *i*th rows of the 2 matrices *M_tumor_* and *M_normal_*, respectively.

(3) **Selection of major mutated genes**. Based on the observations that driver genes tend to be strongly associated with other mutated genes in the PPI network and exhibit differential tumor/normal expression, we selected the major mutated genes for further analysis by the following 2 steps.

**Step 1**. A maximum neighbor weight *W_max_*(*i*) in the PPI network was defined for each gene *i* in *S_remain_* as the largest interaction weight between gene *i* and its interacting neighbors. The top 30% of genes in *S_remain_* with the largest maximum neighbor weights were selected and formed a gene set *G_PPI_*.

**Step 2**. According to gene differential expression, the top 4% of genes in *S_remain_* with the highest differential levels between tumor and normal expression values were selected and formed another gene set, *S_diff_*.

The set of mutated genes *S_major_* was then defined as *S_major_ = S_PPI_*∪*S_diff_*. Accordingly, the genes that were not included in *S_major_* were removed from the 2 expression matrices *M_tumor_* and *M_normal_* with *S* rows and *L* columns after removal.

### Construction of the differential expression network

In this section, a new network named the differential expression network *G_diff_* was constructed to quantify the correlation between each pair of mutated genes in terms of differential expression. The nodes of the network represent the genes in *S_major_*, while the edges between 2 nodes and the node and edge weights were defined as follows.

1. **Generation of the differential expression matrix and calculation of node weights**. Based on the 2 expression matrices *M_tumor_* and *M_normal_*, the expression values were first log transformed, and then the differential expression value *M_diff_*(*i,j*) of gene *i* on sample *j* between tumor and normal was calculated by the following formula.
  \begin{eqnarray*}
{M}_{diff}\left( {i,j} \right) = lo{g}_2\left[ {1 + {M}_{tumor}\left( {i,j} \right)} \right]-{log}_2\left[ {1 + {M}_{normal}\left( {i,j} \right)} \right]
\end{eqnarray*}

Then, *z-score* normalization was performed on *M_diff_* for each gene followed by an absolute value operation.


\begin{eqnarray*}
{\tilde{M}}_{diff}\left( {i,j} \right) = \left| {\frac{{{M}_{diff}\left( {i,j} \right) - mean\left[ {{M}_{diff}\left( i \right)} \right]}}{{{\sigma }_i}}} \right|
\end{eqnarray*}

where $mean[ {{M}_{diff}( i )} ]$ and ${\sigma }_i$ represent the average value and standard deviation of the *i*th row of *M_diff_*, respectively. The matrix ${\tilde{M}}_{diff}$ is defined as the differential expression matrix between the tumor and normal expression matrices *M_tumor_* and *M_normal_*. Then, the differential expression score *W_diff_*(*v_i_*) of gene *i* is defined as the average differential expression values of gene *i* on all *L* samples, which is also assigned as the node weight of gene *i*.


\begin{eqnarray*}
{W}_{diff}\left( {{v}_i} \right) = \frac{1}{l}\mathop \sum \limits_{j = 1}^l {\tilde{M}}_{diff}\left( {i,j} \right),\ i = 1,2, \cdots ,S
\end{eqnarray*}

(2) **Generation of network edges and calculation of edge weights**. Motivated by WGCNA17, which is an expression clustering analysis method that aims to find coexpressed gene modules, we improved it to better adapt to the construction of the differential expression network by the following 2 steps.

**Step 1**. Similar to WGCNA, a Pearson correlation coefficient *cij* was calculated for a pair of genes *i* and *j* in the differential expression matrix ${\tilde{M}}_{diff}$ to measure the correlation of the 2 genes in terms of differential expression. A positive correlation between 2 genes represents a similar pattern of differential expression, and therefore, an edge was added between the 2 genes in the network. However, unlike WGCNA, a negative correlation between 2 genes was considered to indicate that the 2 genes demonstrated different patterns of differential expression, and no edge was added between the 2 genes.

**Step 2**. Similar to WGCNA, it is supposed that the degrees of nodes in the differential expression network should obey a power-law distribution (or long-tailed distribution). To achieve this, an appropriate power ${\alpha }_0$ was calculated and added to the Pearson correlation coefficients. Specifically, the degree ${D}_i( {{\alpha }_0} )$ of node *i* under power ${\alpha }_0$ is defined as follows.


\begin{eqnarray*}
{D}_i\left( \alpha \right) = \mathop \sum \limits_{j \in N\left( i \right)} {\left( {{c}_{ij}} \right)}^{{\alpha }_0}
\end{eqnarray*}

where *N*(*i*) represents the neighbor set of node *i*.

To search for an optimal power ${\alpha }_0$ that makes the degrees of nodes under ${\alpha }_0$ obey a power-law distribution, we exhausted the selections of $\alpha = 1,\ 2,\ \cdots ,6$. For each $\alpha $, the degree values of nodes were ordered and then equally divided into 10 intervals, and the median degrees were selected as the degrees of the intervals, which were denoted as $\{ {{k}^1,\ {k}^2,\ \cdots ,\ {k}^{10}} \}$. Meanwhile, the frequencies of genes in the 10 intervals were represented as $\{ f({k}^1),\ f( {{k}^2} ),\ \cdots ,\ f( {{k}^{10}} )\} $. Then, a linear regression was fitted between $\{ {lo{g}_{10}{k}^1,\ lo{g}_{10}{k}^2,\ \cdots ,\ lo{g}_{10}{k}^{10}} \}$ and $\{ lo{g}_{10}f({k}^1),\ lo{g}_{10}f( {{k}^2} ),\ \cdots ,lo{g}_{10}f( {{k}^{10}} )\} $ with a coefficient of determination ${R}^2$. The $\alpha $ generating the largest ${R}^2$ was selected as the optimal power ${\alpha }_0$. Based on the updated Pearson correlation coefficients, an ${\alpha }_0$-topological correlation coefficient *W_diff_*(*e_ij_*) for each edge *e_ij_* was calculated by applying the topological relationship of the genes in the network using the following formula, which is also assigned as the edge weight of edge *e_ij_*.


\begin{eqnarray*}
{W}_{diff}\left( {{e}_{ij}} \right) = \frac{{\mathop \sum \nolimits_{u \in N\left( i \right) \cap N\left( j \right)} {{\left( {{c}_{iu}} \right)}}^{{\alpha }_0}{{\left( {{c}_{uj}} \right)}}^{{\alpha }_0} + {{\left( {{c}_{ij}} \right)}}^{{\alpha }_0}}}{{\min \left( {{D}_i\left( {{\alpha }_0} \right),\ \ {D}_j\left( {{\alpha }_0} \right)} \right) + 1 - {{\left( {{c}_{ij}} \right)}}^{{\alpha }_0}}}
\end{eqnarray*}

### Calculation of impact scores for mutated gene pairs

In this section, an impact score for each mutated gene pair will be calculated to determine the rank of the pair in driving cancers, which was achieved by applying the mutation scores and topological properties from both the PPI and differential expression networks.

Given a mutation score *M*(*i*) for each gene *i*, a PPI network *G_PPI_*, and a differential expression network *G_diff_*, 2 mutated genes *i* and *j* that are connected by an edge in both the PPI and differential expression networks are defined as a mutated gene pair *p* = *p*(*i, j*). A gene *k* is defined as a neighbor of mutated gene pair *p* in *G_PPI_* (or *G_diff_*) if at least 1 of the 2 edges *e_ki_* and *e_kj_* exists. If only 1 of the 2 edges exists, the effect score *E*(*k, p*) of gene *k* on mutated gene pair *p* is defined as the corresponding edge weight, while the maximum score is defined if both edges exist. Based on the above definitions, a subnetwork *sub*-*G_PPI_* (or *sub-G_diff_*) centered on mutated gene pair *p* was constructed with nodes and edges denoting the mutated gene pair *p* and its neighbors and their connections, and the edge weights representing the effect scores of the neighbors on the mutated gene pair *p*. The association strength of the network *sub-G_PPI_* and the differential level of the network *sub-G_diff_* were then calculated and combined to generate an impact score of the mutated gene pair *p*.

(1) **Calculation of the association strength of the network *sub-G_PPI_***. Given the network *sub-G_PPI_* centered on a mutated gene pair *p* = *p*(*i, j*), the association strength *AS*(*p,k*) between *p* and its neighbor *k* was calculated by the following formula.
  \begin{eqnarray*}
AS\left( {p,k} \right) = \left\{ {\begin{array}{@{}*{1}{c}@{}} {\frac{{M\left( i \right)M\left( j \right)M\left( k \right)}}{{{{\left( {{d}_{ij} \cdot {d}_{pk}} \right)}}^2}}, \quad if\ N_p^{PPI} \ne \emptyset \ }\\ {\frac{{M\left( i \right)M\left( j \right){M}_{min}}}{{{{\left( {{d}_{ij} \cdot {d}_{max}} \right)}}^2}},\ \quad if\ N_p^{PPI} = \emptyset } \end{array}} \right. \end{eqnarray*}

where *M*(*i*) represents the mutation score of gene *i, N_p_^PPI^* represents the set of neighbors of *p* in *G_PPI_, d_ij_* and *d_pk_* are the reciprocals of the edge weights *W_PPI_*(*e_ij_*) and *E*(*k, p*), *M_min_* is the minimum mutation score among all the mutated genes, and *d_max_* denotes the reciprocal of the minimum edge weight in *G_PPI_*.

Based on the above calculations, the association strength *AS*(*p*) of the subnetwork *sub-G_PPI_* centered on *p* was defined as the maximum association strength between the mutated gene pair *p* and its neighbors as follows.


\begin{eqnarray*}
AS\left( p \right) = \left\{ {\begin{array}{@{}*{1}{c}@{}} {\mathop {\max }\limits_{\ k \in N_p^{PPI}} AS\left( {p,k} \right),\quad if\ N_p^{PPI} \ne \emptyset \ }\\ {\frac{{M\left( i \right)M\left( j \right){M}_{min}}}{{{{\left( {{d}_{ij} \cdot {d}_{max}} \right)}}^2}},\quad if\ N_p^{PPI} = \emptyset } \end{array}} \right. \end{eqnarray*}

(2) **Calculation of the differential level of the network *sub-G_diff_***. Based on the network *sub-G_diff_* centered on a mutated gene pair *p* = *p*(*i, j*), the differential level *DL*(*p,k*) between *p* and its neighbor *k* was calculated as follows.
  \begin{eqnarray*}
DL\left( {p,k} \right) = \left\{ {\begin{array}{@{}*{1}{c}@{}} {\frac{{max\left[ {{W}_{diff}\left( {{v}_i} \right),{W}_{diff}\left( {{v}_j} \right)} \right] \cdot {W}_{diff}\left( {{v}_k} \right)}}{{{{\left( {{q}_{pk}} \right)}}^2}},\quad if\ N_p^{diff} \ne \emptyset \ }\\ {\frac{{max\left[ {{W}_{diff}\left( {{v}_i} \right),{W}_{diff}\left( {{v}_j} \right)} \right] \cdot {W}_{diff}\left( {{v}_{min}} \right)}}{{{{\left( {{q}_{max}} \right)}}^2}}, \quad if\ N_p^{diff} = \emptyset } \end{array}} \right. \end{eqnarray*}

where *N_p_^diff^* is the set of neighbors of *p* in *G_diff_*, ${q}_{pk} = 1 - E( {k,{\mathrm{\ }}p} )$, ${q}_{max} = \mathop {\max }\limits_{{e}_{ij} \in {G}_{diff}} [ {1 - {W}_{diff}( {{e}_{ij}} )} ]$, and *W_diff_*(*v_min_*) represents the minimum node weight in *G_diff_*.

Then, the differential level *DL*(*p*) of the subnetwork *sub*-*G_diff_* centered on *p* was defined as the maximum differential level between the mutated gene pair *p* and its neighbors as follows.


\begin{eqnarray*}
DL\left( p \right) = \left\{ {\begin{array}{@{}*{1}{c}@{}} {\mathop {\max }\limits_{\ k \in N_p^{diff}} DL\left( {p,k} \right),\quad if\ N_p^{diff} \ne \emptyset \ }\\ {\frac{{max\left[ {{W}_{diff}\left( {{v}_i} \right),{W}_{diff}\left( {{v}_j} \right)} \right] \cdot {W}_{diff}\left( {{v}_{min}} \right)}}{{{{\left( {{q}_{max}} \right)}}^2}},\quad if\ N_p^{diff} = \emptyset } \end{array}} \right. \end{eqnarray*}

Based on the association strength *AS*(*p*) and *DL*(*p*) of the 2 subnetworks *sub*-*G_PPI_* and *sub*-*Gdiff* centered on *p*, the impact score *DCIS*(*p*) of the mutated gene pair *p* in driving cancers was calculated by multiplying *AS*(*p*) and *DL*(*p*).


\begin{eqnarray*}
DCIS\left( p \right) = AS\left( p \right) \cdot DL\left( p \right) \end{eqnarray*}

### Prioritization of individual mutated genes by partitioning mutated gene pairs

To effectively prioritize individual mutated genes, the impact score of a mutated gene pair *p* = *p*(*i, j*) needs to be partitioned into 2 impact scores, *DCIS*(*i, p*) and *DCIS*(*j, p*), corresponding to the 2 individual mutated genes. The partitioning ratio was determined by the different influences of the 2 mutated genes on the PPI network. In this study, the influence *s*(*i*) of gene *i* on the PPI network is defined as follows.


\begin{eqnarray*}
s\left( i \right) = \mathop \sum \limits_{j \in N_i^{PPI}} {W}_{PPI}\left( {{e}_{ij}} \right) \end{eqnarray*}

where *N_i_^PPI^* represents the neighbors of gene *i* on the PPI network. Therefore, the impact score *DCIS*(*i, p*) of gene *i* based on the mutated gene pair *p* = *p*(*i, j*) was calculated as follows.


\begin{eqnarray*}
DCIS\left( {i,p} \right) = \frac{{s\left( i \right)}}{{s\left( i \right) + s\left( j \right)}}DCIS\left( p \right) \end{eqnarray*}

In practice, a gene *i* may be included in multiple mutated gene pairs, and the maximum one was regarded as the final impact score of the mutated gene.


\begin{eqnarray*}
DCIS\left( i \right) = \mathop {\max }\limits_{j \in N_i^P} \frac{{s\left( i \right)}}{{s\left( i \right) + s\left( j \right)}}DCIS\left( p \right) \end{eqnarray*}

where *N_i_^P^*denotes the set of mutated gene pairs that include gene *i*. Based on the impact scores, individual mutated genes were prioritized accordingly.

## Availability of Supporting Source Code and Requirements

Project name: DriverMP

Project homepage: https://github.com/LiuYangyangSDU/DriverMP

Operating system(s): Linux/Unix

Programming language: C++

Other requirements: g++ version 7.5.0

License: GNU GPL v3.0

RRID: SCR_023796

## Abbreviations

AGM: axon guidance molecule; AUC: area under the receiver operating characteristic curve; AUFC: area under the F1-score curve; BC: breast cancer; BCC: breast cancer cell; BLAST: Basic Local Alignment Search Tool; BMR: background mutation rate; COPD: chronic obstructive pulmonary disease; DNmax: direct neighbor maximum; DNsum: direct neighbor sum; EBV: Epstein–Barr virus; EGFR: epidermal growth factor receptor; FAK: focal adhesion kinase; FDR: false discovery rate; GAD: Genetic Association Database; HDL-C: high-density lipoprotein cholesterol; ICGC: International Cancer Genome Consortium; KEGG: Kyoto Encyclopedia of Genes and Genomes; LUAD: lung adenocarcinoma; NSCLC: non–small cell lung cancer; PPI: protein‒protein interaction; ROC: receiver operating characteristic; RTK: receptor tyrosine kinase; SCLC: small cell lung cancer; TARGET: Therapeutically Applicable Research to Generate Effective Treatments; TCGA: The Cancer Genome Atlas.

## Supplementary Material

giad106_GIGA-D-23-00209_Original_Submission

giad106_GIGA-D-23-00209_Revision_1

giad106_Response_to_Reviewer_Comments_Original_Submission

giad106_Reviewer_1_Report_Original_Submission Chunhou Zheng -- 9/1/2023 Reviewed

giad106_Reviewer_1_Report_Revision_1 Chunhou Zheng -- 11/4/2023 Reviewed

giad106_Reviewer_2_Report_Original_Submission Xionghui Zhou -- 9/17/2023 Reviewed

giad106_Reviewer_2_Report_Revision_1 Xionghui Zhou -- 10/30/2023 Reviewed

giad106_Reviewer_3_Report_Original_Submission Junfeng Xia -- 10/5/2023 Reviewed

giad106_Supplemental_File

## Data Availability

All supporting data and materials are available in the *GigaScience* database, GigaDB [160].

## Additional Files

**Supplementary Figure S1**. ROC curves of DriverMP and the other compared methods using HumanNet PPI network.

**Supplementary Figure S2**. The curves of the numbers of identified known driver genes of the top ranked 500 genes using HumanNet PPI network.

**Supplementary Figure S3**. Performance of F1-scores of DriverMP and other compared methods in ten cancer types using HumanNet PPI network.

**Supplementary Figure S4**. Six-level assessment of PRAD.

**Supplementary Figure S5**. Six-level assessment of LUSC.

**Supplementary Figure S6**. Six-level assessment of HNSC.

**Supplementary Figure S7**. Six-level assessment of COADREAD.

**Supplementary Figure S8**. Six-level assessment of BLCA.

**Supplementary Figure S9**. Six-level assessment of UCEC.

**Supplementary Figure S10**. Six-level assessment of KIRC.

**Supplementary Figure S11**. Six-level assessment of KIRP.

**Supplementary Table S1**. Summary of the ten somatic non-silent mutation and tumor/normal expression datasets of each cancer type from TCGA.

**Supplementary Table S2**. List of genes for the four BC-related diseases against GAD.

**Supplementary Table S3**. List of genes for the four LUAD-related diseases against GAD.

**Supplementary Table S4**. Six levels of the 71 driver candidates of PRAD.

**Supplementary Table S5**. 18 driver candidates of “Literature-supported level” of prostate cancer.

**Supplementary Table S6**. Biological pathways associated with prostate cancer.

**Supplementary Table S7**. 23 driver candidates of “Gene level” of PRAD.

**Supplementary Table S8**. Six levels of the 68 driver candidates of LUSC.

**Supplementary Table S9**. 14 driver candidates of “Literature-supported level” of LUSC.

**Supplementary Table S10**. Biological pathways associated with LUSC.

**Supplementary Table S11**. 23 driver candidates of “Gene level” of LUSC.

**Supplementary Table S12**. Six levels of the 65 driver candidates of HNSC.

**Supplementary Table S13**. 12 driver candidates of “Literature-supported level” of HNSC.

**Supplementary Table S14**. Biological pathways associated with HNSC.

**Supplementary Table S15**. 26 driver candidates of “Gene level” of HNSC.

**Supplementary Table S16**. Six levels of the 60 driver candidates of COADREAD.

**Supplementary Table S17**. 16 driver candidates of “Literature-supported level” of COADREAD.

**Supplementary Table S18**. Biological pathways associated with COADREAD.

**Supplementary Table S19**. 20 driver candidates of “Gene level” of COADREAD.

**Supplementary Table S20**. Six levels of the 70 driver candidates of BLCA.

**Supplementary Table S21**. 16 driver candidates of “Literature-supported level” of BLCA.

**Supplementary Table S22**. Biological pathways associated with BLCA.

**Supplementary Table S23**. 25 driver candidates of “Gene level” of BLCA.

**Supplementary Table S24**. Six levels of the 52 driver candidates of UCEC.

**Supplementary Table S25**. 8 driver candidates of “Literature-supported level” of UCEC.

**Supplementary Table S26**. Biological pathways associated with UCEC.

**Supplementary Table S27**. 25 driver candidates of “Gene level” of UCEC.

**Supplementary Table S28**. Six levels of the 45 driver candidates of KIRC.

**Supplementary Table S29**. 12 driver candidates of “Literature-supported level” of KIRC.

**Supplementary Table S30**. Biological pathways associated with KIRC.

**Supplementary Table S31**. 13 driver candidates of “Gene level” of KIRC.

**Supplementary Table S32**. Six levels of the 56 driver candidates of KIRP.

**Supplementary Table S33**. 11 driver candidates of “Gene level” of KIRP.
