# Supplementary material for: DriverMP enables improved identification of cancer driver genes
Source: Gigascience. 2023 Dec 13;12:giad106. doi: 10.1093/gigascience/giad106 (PMC10716827; doi:10.1093/gigascience/giad106)
Supplement: giad106_GIGA-D-23-00209_Original_Submission [file giad106_giga-d-23-00209_original_submission.pdf]

GigaScience

DriverMP enables improved identification of cancer driver mutations

--Manuscript Draft--

**Manuscript Number:**	GIGA-D-23-00209
**Full Title:**	DriverMP enables improved identification of cancer driver mutations
**Article Type:**	Technical Note
**Funding Information:**	National Key R&D Program of China (2020YFA0712400) — Dr. Juntao Liu<br>National Natural Science Foundation of China (62272268) — Dr. Juntao Liu
**Abstract:**	Background<br><br>Cancer is widely regarded as a complex disease primarily driven by genetic mutations. A critical concern and significant obstacle lies in discerning driver mutations amidst an extensive array of passenger mutations.<br><br>Findings<br><br>We present a new method termed DriverMP for effectively prioritizing altered genes on a cancer-type level by considering mutated gene pairs. It is designed to first apply somatic mutation data, protein–protein interaction network data, and differential gene expression data to prioritize mutation pairs, and then individual mutated genes are prioritized based on prioritized mutation pairs. Application of this method in ten TCGA cancer datasets demonstrated its great improvements over all the compared state-of-the-art methods in identifying known driver mutations. Then, a comprehensive analysis demonstrated the reliability of the novel driver mutations that are strongly supported by clinical experiments, disease enrichment, or biological pathway analysis.<br><br>Conclusions<br><br>The new method, DriverMP, is able to identify driver mutations by effectively integrating the advantages of multiple kinds of cancer data, which is available at https://github.com/LiuYangyangSDU/DriverMP). In addition, we have developed a novel driver gene database (available at http://www.liulab.top/DriverMP/table) for ten cancer types and an online service that can be freely accessed without registration for users (available at http://liulab.top/DriverMP/server). The DriverMP method, the database of novel drivers, and the user-friendly online server are expected to contribute to new diagnostic and therapeutic opportunities for cancers.
**Corresponding Author:**	Juntao Liu<br>Shandong University<br>Weihai, CHINA
**Corresponding Author Secondary Information:**	
**Corresponding Author's Institution:**	Shandong University
**Corresponding Author's Secondary Institution:**	
**First Author:**	Yangyang Liu
**First Author Secondary Information:**	
**Order of Authors:**	Yangyang Liu<br>Jiyun Han<br>Nannan Xiao<br>Tongxin Kong

Powered by Editorial Manager® and ProduXion Manager® from Aries Systems Corporation

	Qinglin Mei
	Juntao Liu
**Order of Authors Secondary Information:**	
**Additional Information:**	
**Question**	**Response**
Are you submitting this manuscript to a special series or article collection?	No
**Experimental design and statistics**<br><br>Full details of the experimental design and statistical methods used should be given in the Methods section, as detailed in our Minimum Standards Reporting Checklist. Information essential to interpreting the data presented should be made available in the figure legends.<br><br>Have you included all the information requested in your manuscript?	Yes
**Resources**<br><br>A description of all resources used, including antibodies, cell lines, animals and software tools, with enough information to allow them to be uniquely identified, should be included in the Methods section. Authors are strongly encouraged to cite Research Resource Identifiers (RRIDs) for antibodies, model organisms and tools, where possible.<br><br>Have you included the information requested as detailed in our Minimum Standards Reporting Checklist?	Yes
**Availability of data and materials**<br><br>All datasets and code on which the conclusions of the paper rely must be either included in your submission or deposited in publicly available repositories (where available and ethically appropriate), referencing such data using	Yes

a unique identifier in the references and in the “Availability of Data and Materials” section of your manuscript.<br><br>Have you have met the above requirement as detailed in our Minimum Standards Reporting Checklist?	

Manuscript

Click here to access/download;Manuscript;Manuscript of DriverMP.docx

DriverMP enables improved identification of cancer driver mutations

Yangyang Liu[1], Jiyun Han[1], Nannan Xiao[1], Tongxin Kong[1], Qinglin Mei[2], Juntao Liu[1,*]

[1]School of Mathematics and Statistics, Shandong University (Weihai), Weihai, 264209, China

[2]MOE Key Laboratory of Bioinformatics, BNRIST Bioinformatics Division, Department of Automation, Tsinghua University, Beijing, China

[*]To whom correspondence should be addressed

Abstract

Background: Cancer is widely regarded as a complex disease primarily driven by genetic mutations. A critical concern and significant obstacle lies in discerning driver mutations amidst an extensive array of passenger mutations.

Findings: We present a new method termed DriverMP for effectively prioritizing altered genes on a cancer-type level by considering mutated gene pairs. It is designed to first apply somatic mutation data, protein–protein interaction network data, and differential gene expression data to prioritize mutation pairs, and then individual mutated genes are prioritized based on prioritized mutation pairs. Application of this method in ten TCGA cancer datasets demonstrated its great improvements over all the compared state-of-the-art methods in identifying known driver mutations. Then, a comprehensive analysis demonstrated the reliability of the novel driver mutations that are strongly supported by clinical experiments, disease enrichment, or biological pathway analysis.

Conclusions: The new method, DriverMP, is able to identify driver mutations by effectively integrating the advantages of multiple kinds of cancer data, which is available at https://github.com/LiuYangyangSDU/DriverMP). In addition, we have developed a novel driver gene database (available at http://www.liulab.top/DriverMP/table) for ten cancer types and an online service that can be freely accessed without registration for users (available at http://liulab.top/DriverMP/server). The DriverMP method, the database of novel drivers, and the user-friendly online server are expected to contribute to new diagnostic and therapeutic opportunities for cancers.

Keywords: Cancer genomics, Driver mutations, Multi-omics in Cancer, Mutated gene pairs

Introduction

Cancer is one of the most complex diseases, and there are currently more than 100 known types of cancers in humans [1]. The most widely accepted theory is that cancer is mainly caused by genetic mutations [2]. For this reason, several large-scale cancer sequencing projects, such as The Cancer Genome Atlas (TCGA) [3], the International Cancer Genome Consortium (ICGC) [4] and Therapeutically Applicable Research to Generate Effective Treatments (TARGET) [5], have generated a large amount of multiomics data for various cancer types, and the resulting databases have accelerated the discovery of cancer mutations. Related studies have shown that among the large number of somatic mutated genes, only a small number of them (the so-called driver mutations) confer a selective advantage to cancers, and most of them (the so-called passenger mutations) exhibit little impact on cancer progression [2, 6, 7].

A key issue and major challenge is to distinguish driver mutations from a very large number of passenger mutations [2]. The most basic and intuitive approach is to prioritize all somatic mutations according to their occurrence frequencies based on the hypothesis that drivers demonstrate higher mutation rates than expected [8]. A great number of methods have been developed to identify driver mutations based on mutation frequencies; these include MutSig2.0 [9], MuSigCV [10], MuSiC [7] and WITER [11]. These approaches first attempt to estimate the background mutation rate (BMR) and then compare the mutation frequency of each gene with the BMR to identify driver mutations. In addition, other methods, such as Mutation_assessor [12], CHASM [13], transFIC [14], and FATHMM [15], distinguish driver mutations from passenger mutations by assessing the functional impact of mutations.

Although significant efforts have been made in the accurate identification of driver mutations, mutation frequency-based approaches have shown limited power in practical applications because most cancers demonstrate extensive mutational heterogeneity across samples [16]. Studies have shown that only a small number of cancer drivers are frequently mutated, and most of them are mutated in a few samples, which is the so-called ‘long-tail’ phenomenon [2, 6, 7]. This phenomenon highlights an enormous challenge in the identification of rarely mutated driver genes by methods based on mutation frequency. Fortunately, recent studies revealed that driver mutations in a given cancer type usually act together in a limited number of biological pathways or protein complexes, although the cancer genes demonstrate a random distribution

across different samples [17, 18]. Therefore, rarely mutated drivers may be identified by applying information on biological pathways or networks. Based on this fact, quite a few approaches have been developed to increase the prediction accuracy. Functional network-based methods, such as HotNet2 [17] and VarWalker [19], predict numerous “cancer modules” containing drivers rather than individual genes by using the “heat diffusion”-like model in physics to determine “mutation scores” in protein–protein interaction (PPI) networks. Mutation- and network-based methods, such as MUFFINN [16] and MaxMIF [20], were developed by integrating the information of both mutation and functional networks to prioritize mutations. MUFFINN defined two kinds of mutation scores for ranking genes by considering mutations in the most frequently mutated neighbour (direct neighbour maximum, DNmax) and mutations in all direct neighbours with normalization by their degree connectivity (direct neighbour sum, DNsum). MaxMIF involves a maximum mutation impact function that considers the mutation frequencies of two genes and the interaction strength between them in the PPI network. These approaches effectively improve the prediction of driver mutations involved in the biological networks; however, their false positive rates are still too high. Therefore, new algorithms considering more meaningful biological information are urgently needed.

Related studies show that driver mutations tend to change the expression of their interacting partners or genes that share the same biological pathways, which directly alters the expression of all genes in a biological subnetwork or in pathways associated with driver mutations. In contrast, passenger mutations do not usually cause significant changes in gene expression [21]. Therefore, making better use of gene expression data will undoubtedly facilitate the identification of driver mutations. Several related methods have been developed; these include iPDG [22], DriverNet [21], and DawnRank [23] which prioritize mutations by combining multiple types of data, including mutation, PPI network, biological pathway, and differential gene expression data. iPDG evaluates the changes in expression levels of “key genes” to identify potential driver mutations by using DNA copy number variation, somatic mutation and gene expression data. Both DriverNet and DawnRank rank potential driver genes based on their impact on the overall differential expression of downstream genes in a molecular interaction network. The above methods, which combine multiple types of biological information, have

made the identification of driver mutations more accurate and reliable; however, their prediction effects are still far from satisfactory.

We introduce a new method called DriverMP that prioritizes cancer genes on a cancer-type level by utilizing somatic mutation data, protein–protein interaction networks, and differential expression data. The new approach effectively improves the identification of driver mutations mainly in the following ways. (i) Based on our observation that most cancer driver genes have a driver neighbour in the PPI network, DriverMP first prioritizes the mutation pairs, and then the individual mutations are prioritized by dividing each mutation pair. (ii) DriverMP redefines a mutation score for each mutated gene based on the mutation frequency by balancing the contribution of each sample with different numbers of mutations. (iii) Based on the gene differential expression data, DriverMP constructs a new network, called the differential expression network, to quantify the differential expression of each gene and the relationship between two differentially expressed genes. (iv) Combining the topological properties from both the PPI network and differential expression network, DriverMP generates an impact score for each mutation pair and then generates an impact score for each mutation based on the contribution of each individual gene to complete the prioritization of cancer mutations.

Based on the known driver mutations, the performance of DriverMP was evaluated in ten common cancer types and compared with the performance of eight other state-of-the-art approaches, including MutSig2.0 [9], MutSigCV [10], Mutation_Assessor [18], MaxMIF [20], DawnRank [23], MUFFINN [16] and DriverNet [21], in terms of the receiver operating characteristic (ROC) curve, area under the ROC curve (AUC), cumulative number curve of the top 500 genes, F1-score curve of the top 500 genes and area under the F1-score curve (AUFC). DriverMP consistently demonstrated the best performance in all ten cancer types.

With respect to the novel drivers, DriverMP identified multiple potential driver mutations with high impact scores for each cancer type, and these findings are strongly supported by the results of clinical experiments, disease enrichment analysis, or biological pathway analysis. For example, we found 14 and 10 novel candidates that are significantly related to breast cancer and breast cancer-related diseases, respectively, via disease enrichment analysis. In addition, another 6 genes, PRKDC, NCL, CCNA2, AXL, GLI3 and SUPT5H, were identified to be highly related to the growth and development of breast cancer, the overall and postprogression

survival in breast cancer patients, the regulation of the gene expression that controls the proliferation, migration, cell cycle and apoptosis of breast cancer MDA-MB-231 cells, and so on. We provided a database of those potential driver mutations with detailed descriptions of strong biological or clinical evidence for each of the ten cancer types. Moreover, we observed that the novel candidates enriched in the corresponding cancer type according to disease enrichment analysis formed a significantly dense and highly weighted subnetwork in the PPI network (the *p value* is approximately 0). This observation coincided with previous findings that mutations in the cancer genome tend to converge in a few biological pathways [18] and that genes act together in various signalling and regulatory pathways and protein complexes [17]. The new method and the identified novel driver candidates are expected to contribute to a deeper understanding of the architecture of cancers and new diagnostic and therapeutic opportunities for cancers.

Results

Overview of DriverMP

Based on our observation that most driver mutations have a driver neighbour in the PPI network, we propose that assessment of mutation pairs would capture cancer driver characteristics in a more realistic pattern than direct assessment of individual mutations. Therefore, unlike conventional approaches that prioritize individual mutations directly, DriverMP is designed to first prioritize mutation pairs. According to another observation that driver mutations tend to converge to a limited number of biological pathways or protein complexes, those mutation pairs will be ranked higher if they are strongly associated with other mutations in the PPI network. The new framework will quantify the association strength between each mutation pair and its mutated neighbours in the PPI network. Considering that driver mutations are expected to exhibit different expression patterns and simultaneously influence the expression of their interacting genes in a biological subnetwork, a mutation pair is ranked highly if it, as well as the subnetwork centred on it, are highly differentially expressed. The new DriverMP approach quantifies the differential expression of the subnetwork centred on a mutation pair. DriverMP is a novel approach that involves an innovative strategy—it considers both biological networks and differential gene expression, which can facilitate the identification of rarely mutated cancer

drivers.

DriverMP was developed based on the characteristics of driver mutations

To explore the interaction characteristics of known driver genes in the PPI network, we calculated three metrics: the percentage of codrivers (a driver gene is defined as a codriver if it has at least one driver neighbour in the PPI network), the average connection density (the connection density of a driver gene is defined as the number of its driver neighbours), and the average connection strength (the connection strength of a driver gene is defined as the sum of the edge weights between the gene and its driver neighbours).

(1) Driver genes usually interact with one another. To investigate the interactions between driver genes, we calculated the percentage of driver mutations occurring with other driver mutations among all known driver genes, and the results showed that the percentages reached 93.9% and 99.8%, respectively, based on the HumanNet [24] and STRINGv10 [25] PPI networks. In addition, we calculated the percentage of passenger genes (not included in the CGC) that have at least one driver neighbour, and it was only 64.5% and 92.1% based on the two networks, which demonstrates the obvious phenomenon of codrivers in PPI networks.

(2) The interactions between driver genes are highly dense. The average connection density of known driver genes reached 12.4 and 106.5 based on the HumanNet and STRINGv10 PPI networks, respectively. However, the average number of edges between passenger and driver genes was only 3.6 and 27.2 in the two networks, which also clearly demonstrates the phenomenon of dense interactions between drivers.

(3) The dense interactions between driver genes are extremely strong. The average strength score for connections between driver genes reached 4.0 and 38.5 in the HumanNet and STRINGv10 PPI networks, respectively. In comparison, the average weight of the edges between passenger and driver genes was only 1.0 and 8.2 based on the two PPI networks, demonstrating that the interactions between driver genes are generally stronger than those between passenger genes.

These observations suggest the interaction properties of driver genes in the PPI network, which seems consistent with a previous observation that driver genes tend to be enriched in a few biological pathways, where gene–gene interactions are more frequent. Based on these

interaction characteristics of driver genes, the DriverMP method was developed to focus on the prioritization of mutation pairs.

DriverMP demonstrates great improvement in overall performance

To evaluate the overall performance of DriverMP in the prediction of driver mutations, we employed nonsilent somatic mutation data and differential expression data collected for ten cancer types from the TCGA, including BRCA, PRAD, LUAD, LUSC, KIRC, KIRP, HNSC, COADREAD, UCEC and BLCA, and two independently developed PPI networks (based on STRING v10 and HumanNet) were applied (see the Methods section for details). To evaluate the improvements of DriverMP over the other state-of-the-art methods, seven driver mutation predictors [MutSig2.0, MutSigCV, Mutation_Assessor, DawnRank, MaxMIF, MUFFINN (DNmax and DNsum) and DriverNet] were selected for the overall performance comparison. Additionally, we added a comparison with the frequency-based approach named Freq-based, which ranks mutations based solely on frequency. The overall performance of all the methods was compared under the criteria of ROC curves and AUC values, which effectively evaluate the overall sensitivity and specificity of mutation prioritization.

After comparison based on ROC curves, DriverMP demonstrated high improvements over all the compared methods on all ten datasets when using the STRINGv10 PPI network (see Fig. 2). Similar results were obtained with the HumanNet PPI network (see Supplemental Materials for details). In addition, the AUC values of the ROC curves were calculated for the compared methods in the analysis of the STRINGv10 PPI network, and the results showed that the AUC values of DriverMP were much higher than those of all the other methods in all ten cancer types, and the average improvements rate of DriverMP over the other compared methods in the ten datasets reached 13.7%-33.5% (see Fig. 5a). Similar improvements were obtained with DriverMP in the analysis of the HumanNet PPI network (see Supplemental Materials for details). Therefore, DriverMP shows the best overall performance among all the compared methods under the criteria of ROC curves and AUC values.

DriverMP shows great improvement in identifying top-ranked mutations

In practical applications, only the top-ranked mutations can be confirmed by subsequent

experiments. To compared the performance of DriverMP and other methods based on top-ranked mutations, we analysed the top 500 mutations ranked by each method and compared the number of identified known driver mutations, the F1-score, and the AUFC based on the STRINGv10 network.

After comparison, DriverMP showed high improvements over all the others in the ten cancer types in terms of the number of identified known driver mutations (see Fig. 3). To evaluate the overall performance of DriverMP by combining the sensitivity and precision, the F1-score curves and the AUFC values were calculated, and the results showed that DriverMP consistently performed the best among all the compared methods on all ten datasets (see Fig. 4 and Fig. 5b). Specifically, the average improvements of DriverMP over the others on the ten datasets reach 19.4%-267.5% in terms of AUFC. Similar results were obtained by using the HumanNet PPI network under the three criteria (see Supplemental Materials for details). In conclusion, DriverMP demonstrated superior predictive power over all the other methods in identifying the top-ranked mutations.

DriverMP demonstrates stable performance with a reduced sample number

Although substantial efforts have been made in DNA sequencing to identify possible cancer mutations, many cancer types have only a limited number of sequenced samples available due to the complexity, expense and time-consuming nature of clinical experiments. Therefore, the performance of a practical driver mutation identifier cannot be highly dependent on the number of samples. To evaluate the stability of DriverMP, we tested it under the following two conditions: (1) random selection of only 80%, 50%, and 20% of the samples in the constructed differential expression matrix; (2) random selection of only 80%, 50%, and 20% of the samples in the mutation dataset. Each of the above random selections was performed ten times, and the average AUC and AUFC values were calculated to evaluate the performance of DriverMP.

DriverMP exhibited highly stable performance in terms of differential expression analysis (see Fig. 6a and Fig. 6b), and only a slight decrease in prediction power was observed even when 80% of the samples were removed. For the mutation data, although the stability of DriverMP in predicting mutations was not as high as that in predicting differential expression, it still demonstrated stable performance, especially when 20% and 50% of the samples were

removed. It seems that the stability of DriverMP is relatively worse when 80% of the samples were removed; however, the average differences in AUC and AUFC before and after sample removal were only 0.015 and 0.029, respectively, for the ten cancer types. Therefore, DriverMP shows highly stable performance even when only a small number of samples are available.

The framework of DriverMP was designed by deeply mining the relationships among mutated genes and effectively fusing different sources of cancer data, which may be the reason why DriverMP is able to maintain stable performance even when many samples are removed. Moreover, the low dependence of DriverMP on the number of samples directly contributes to the identification of infrequently mutated driver genes buried in the long tail.

DriverMP reliably predicts novel driver genes

In the absence of a well-defined and systematic approach to identifying driver candidates, we proposed a cancer-specific five-level assessment by a comprehensive literature survey. We then applied it to assess the quality of the driver candidates predicted by DriverMP in all ten cancer types. The main text shows the results for DriverMP analysis of two popular cancer types, breast cancer and lung adenocarcinoma, and similar results for DriverMP analysis of the other eight cancer types are shown in the supplemental materials. The detailed rules of the five-level assessment are illustrated as follows.

(1) Cancer-type level. In this level of analysis, data is collected on driver candidates that are enriched in different cancer types from the well-known tool DAVID [26] against the Genetic Association Database (GAD) [27], which is a developing archive of human genetic association studies of complex diseases and disorders allowing the comprehensive analysis of complex common human genetic diseases.

(2) Literature-supported level. In this level of analysis, the driver candidates are divided into three categories based on literature type: (i) overview studies (O) that summarize previous studies; (ii) experimental studies (E) that verify the mechanism of carcinogenesis via in vivo or clinical trials; (iii) bioinformatics studies (B) that infer cancer mechanisms via reliable statistical or bioinformatics analysis of large multiomics datasets.

(3) Pathway level. In this level of analysis, candidates that are enriched in biological pathways directly related to specific cancers with FDR < 10-3 are identified by using the tool

STRING (https://cn.string-db.org/) against well-recognized biological pathway databases, such as Kyoto Encyclopedia of Genes and Genomes (KEGG) [28] and Reactome [29].

(4) Disease level. In this level of analysis, genes that are enriched in diseases that are associated with specific cancers are identified using DAVID against the GAD database.

(5) Validation-required level. In the fifth level of analysis, the remaining genes that are not included in the above four levels are noted; these genes require further validation.

DriverMP reliably predicts novel drivers for breast cancer

Breast cancer (BC) is the most commonly diagnosed life-threatening cancer and the leading cause of cancer death in women [30]. In this study, we utilized DriverMP under both the STRINGv10 and HumanNet networks to identify potential driver genes in BC. We identified a total of 51 novel driver candidates that ranked within the top 250 in both networks and were not previously included in CGC. These candidates underwent the five-level assessment and were subsequently divided into five groups (Table 1).

Table 1. Five-level assessment of the 51 novel driver candidates of BC.

No.	Level of analysis	Driver candidates	Count	Percentage
1	Cancer type level	ABCB1, APEX1, CCNB1, CDC14A, CDK2, ERCC6, IGF1R, INSR, PIK3CB, PIK3CG, PRKDC, RAD51, TOP2A, TP53BP1	14	27.45%
2	Literature-supported level	AXL, CCNA2, CCNB1, CDK5, ERCC6, GLI3, IGF1R, NCL, PRKDC, RAD51, SUPT5H, TP53BP1	12	23.53%
3	Pathway level	ANK3, APEX1, AXL, CACNA1A, CCNA2, CCNB1, CD4, CDC14A, CDK2, CDK5, CHD3, CTNNA1, DLG1, EEF2, ERCC6, GLI3, HDAC1, HSPA8, HSPA9, IGF1R, INSR, MAPK3, MAPK8, MDC1, NR3C1, PIK3CB, PIK3CG, POLA1, PRCKDC, PRKDC, PTK2, RPS6KA1, SGK1, SIN3A, SLC2A4, SMARCA2, SP3, SUMO1, SUPT5H, TAF1, TOP2A, TP53BP1, TYK2, VAV1	44	86.27%
4	Disease level	CDK5, IGF1R, INSR, MAPK3, NR3C1, PIK3CB, SGK1, SLC2A4, SMARCA2, SUMO1	10	19.61%
5	Validation-required level	CAD, EEF1A1, NEB, SMARCA1, TTN	5	9.80%

Cancer-type level. Based on the wealth of annotated genes associated with complex diseases in GAD, we used DAVID to confirm the relevance of the 51 driver candidates to breast cancer and found that 31 (60.8%) of these candidates were enriched in "cancer" ($p = 1.4 \times 10^{-9}, FDR = 2.5 \times 10^{-8}$) and 14 (45.2%) of them were enriched in "breast cancer" ($p = 4.5 \times 10^{-8}, FDR = 3.3 \times 10^{-5}$). Therefore, analysis at the cancer-type level yielded of 14 novel genes that are noted as being related to BC. In addition, we performed pathway enrichment for

the 14 genes in the first level of analysis, and the results showed that five genes, IGF1R, INSR, CDK2, PIK3CB, and CCNB1, were enriched in the "FoxO signalling pathway" (KEGG, $FDR = 5.2 \times 10^{-3}$) and six genes, CDK2, RAD51, PRKDC, APEX1, ERCC6, and TP53BP1, were enriched in "DNA repair" (Reactome, $FDR = 6.7 \times 10^{-3}$) (Fig. 7a). Related studies have shown that the lack of functional FOXO proteins such as FOXO3a leads to the development of breast tumours [31, 32] and that DNA repair-related pathways play an important role in the pathogenesis of breast cancer [33].

Literature-supported level. Based on the comprehensive survey of reliable literature, 12 novel genes supported by relevant studies were classified into this group (Table 2). The function of each of the 12 genes and the relationship of each gene with breast cancers are briefly described in Table 2. For example, studies have demonstrated that suppressing CDK5 can impede cell motility and tumour development in the mesenchymal breast cancer cell lines MDA-MB-231 and BT549 [34], while elevated expression of PRKDC promotes breast cancer cell proliferation by regulating p38 MAPK signalling [35].

Table 2. 12 driver candidates of "Literature supported level" of breast cancer.

Gene	NCBI Entrez ID	Rank (HumanNet)	Rank (STRINGv10)	Function	Type
IGF1R	3480	13	133	IGF1R, as part of insulin-like growth factor (IGF) signalling, is highly overexpressed in most malignant tissues where it functions as an anti-apoptotic agent by enhancing cell survival [36].	O
RAD51	5888	20	67	Breast cancer driver gene BRCA2 directed the binding of RAD51 recombinase to ssDNA, reduced the binding of RAD51 to duplex DNA and stimulated RAD51-mediated DNA strand exchange [37].	E
TP53BP1	7158	50	105	TP53BP1 may be associated with breast cancer staging and breast cancer prognosis [38].	B
ERCC6	2074	73	111	Integrative genomics approach suggests that ERCC6 may be a previously unreported low- to moderate-risk breast cancer susceptibility gene, which may also interact with ERCC8 [39].	B
CDK5	1020	119	196	CDK5 is commonly overexpressed and significantly correlated with several poor prognostic parameters of breast cancer. Its overexpression also exhibited a potential synergy in promoting TGF-β1-induced EMT [34].	E
CCNB1	891	213	220	CCNB1 is a biomarker for the prognosis of ER+ breast cancer and monitoring of hormone therapy efficacy [40].	B
PRKDC	5591	16	20	PRKDC are all involved with the growth and development of breast cancer cells [35].	E
NCL	4691	42	222	NCL is commonly overexpressed in human breast tumours and that its expression correlates with that of NCL-dependent miRNAs [41].	E
CCNA2	890	61	127	Kaplan–Meier survival analyses confirmed that elevated CCNA2, and CCNB1 expression levels were associated with overall and postprogression survival and recurrence-free probability rates in patients with BRCA [42].	B
AXL	558	153	70	In breast cancer AXL expression has been observed in all of the main transcriptional subtypes, and AXL expression in primary breast tumours is strongly predictive of reduced patient survival and poor outcome [43].	E

GLI3	2737	178	45	ERα+ BRCA cell growth is dependent on Gli3, which indicate that Gli might be a preferential target for the clinical management of ERα+ BRCA [44].	E
SUPT5H	6829	231	206	SUPT5H plays an important role in BRCA tumorigenicity by regulating the expression levels of genes that control the proliferation, migration, cell cycle and apoptosis of breast cancer MDA-MB-231 cells [45].	E

Pathway level. To investigate whether the enriched biological pathways are associated with breast cancers, pathway enrichment was performed using the 51 driver candidates. A total of 15 and 13 breast cancer-related biological pathways were identified using the KEGG and Reactome databases, respectively, covering 44 of the 51 driver candidates (see Table 3 for the functional descriptions of the pathways and their associations with breast cancers). Specifically, the KEGG enrichment results showed that 8 and 6 genes were enriched in two pathways, "progesterone-mediated oocyte maturation" ($FDR = 3.6 \times 10^{-8}$) and "type II diabetes mellitus" ($FDR = 4.7 \times 10^{-7}$), respectively, both of which are directly associated with breast cancers (Fig. 7b). Multiple studies have reported that the pathogenesis and prognosis of breast cancer are highly related to progesterone-mediated oocyte maturation [46, 47]. The correlation between type 2 diabetes and BC is also very strong, which will be discussed in detail in the next section. Reactome analysis showed that 10 (19.6%) and 11 (21.6%) genes were enriched in the "transcriptional regulation by TP53" ($FDR = 2.5 \times 10^{-5}$) and "signalling by receptor tyrosine kinases" ($FDR = 2.8 \times 10^{-6}$) pathways, respectively (Fig. 7c). TP53 is a well-known tumour suppressor gene that drives multiple cancers, including BC [48]. Under stress conditions, it regulates the transcription of many genes involved in various cellular processes, such as cellular metabolism, survival, senescence, apoptosis and the DNA damage response [49]. For the "signalling by receptor tyrosine kinases" pathway, several studies have suggested that high levels of RTKs may be associated with increased breast cancer aggressiveness and decreased overall and disease-free survival [50-55].

Table 3. Biological pathways associated with breast cancer.

Database	ID	Pathway	Genes	Count	Function	FDR
KEGG	hsa04068	FoxO signalling pathway	CCNB1, CDK2, IGF1R, INSR, MAPK3, MAPK8, PIK3CB, SGK1, SLC2A4	9	Lack of functional FOXO proteins such as FOXO3a leads to the development of breast tumours [31, 32].	2.26E-08
	hsa04914	Progesterone-mediated oocyte maturation	CCNA2, CCNB1, CDK2, IGF1R, MAPK3, MAPK8, PIK3CB, RPS6KA1	8	Several bioinformatic analyses suggest that the pathogenesis and prognosis of breast cancer are associated with progestin-mediated oocyte maturation [46, 47].	3.59E-08
	hsa04930	Type II diabetes mellitus	CACNA1A, INSR, MAPK3, MAPK8, PIK3CB, SLC2A4	6	It is reported that up to 16% of BC patients have diabetes, and type 2 diabetes may be associated with a 10-20% increased relative risk of BC [56, 57].	4.74E-07
	hsa04110	Cell cycle	CCNA2, CCNB1, CDK2, CDC14A, HDAC1, PRKDC	6	Therapeutic targeting of the cell cycle has long been viewed as a promising anticancer strategy [58].	4.31E-05
	hsa04152	AMPK signalling pathway	CCNA2, EEF2, IGF1R, INSR, PIK3CB, SLC2A4	6	Downregulation of AMPK activity or decreased level involved in the promotion of breast tumorigenesis, and thus activation of AMPK found to oppose tumour progression [59].	4.31E-05

	hsa05169	Epstein–Barr virus infection	CCNA2, CDK2, HDAC1, MAPK8, PIK3CB, SIN3A, TYK2	7	A study confirmed the presence of EBV in one third of BC and demonstrated that EBV-positive tumours presented with a more aggressive phenotype that could be useful when considering potential therapeutic targets [60].	4.31E-05
	hsa04150	mTOR signalling pathway	IGF1R, INSR, MAPK3, PIK3CB, RPS6KA1, SGK1	6	One of the most commonly altered pathways driving breast cancer cell growth, survival, and motility is the PI3K/AKT/mTOR signalling cascade [61].	8.25E-05
	hsa04151	PI3K-Akt signalling pathway	CDK2, IGF1R, INSR, MAPK3, PIK3CB, PIK3CG, PTK2, SGK1	8		8.77E-05
	hsa01522	Endocrine resistance	IGF1R, MAPK3, MAPK8, PIK3CB, PTK2	5	Endocrine therapies that target oestrogen action (anti-oestrogens and aromatase inhibitors) are widely used and successful breast cancer therapies, but many women treated with these therapies will relapse with endocrine-resistant disease [62].	1.20E-04
	hsa05203	Viral carcinogenesis	CCNA2, CDK2, DLG1, HDAC1, MAPK3, PIK3CB	6	Virus-associated cancer refers to a cancer where viral infection results in the malignant transformation of the host's infected cells. Human papillomaviruses (HPV), mouse mammary tumour virus (MMTV) and Epstein–Barr (EBV) virus are prime candidate viruses as agents of human breast cancer [63].	1.70E-04
	hsa04931	Insulin resistance	INSR, MAPK8, PIK3CB, RPS6KA1, SLC2A4	5	Biological markers of insulin resistance such as the insulin level, the insulin/glucose ratio, HOMA, adiponectin, leptin/adiponectin, decreased SHBG, have been associated with an increased risk of breast cancer essentially in postmenopausal breast cancer women [64].	1.80E-04
	hsa04510	Focal adhesion	IGF1R, MAPK3, MAPK8, PIK3CB, PTK2, VAV1	6	The link between FAK and breast cancers is strongly suggested by numerous reports showing that FAK gene is amplified and overexpressed in a large fraction of breast cancer specimens [65].	2.10E-04
	hsa05205	Proteoglycans in cancer	ANK3, IGF1R, MAPK3, PIK3CB, PTK2, VAV1	6	Proteoglycan biosynthesis is dysregulated in breast cancer and targeting proteoglycans may provide new therapeutic approaches for breast cancer [66].	2.10E-04
	hsa04910	Insulin signalling pathway	INSR, MAPK3, MAPK8, PIK3CB, SLC2A4	5	Insulin receptor (IR) is present in many malignant cells, including breast cancer cells, and that insulin may be involved in the growth of these malignancies [67].	3.70E-04
	hsa04012	ErbB signalling pathway	MAPK3, MAPK8, PIK3CB, PTK2	4	Activation of ErbB signalling can regulate EMT-associated invasion and migration in normal and malignant mammary epithelial cells, as well as modulating discrete stages of mammary gland development [68].	7.80E-04
**Reactome**	HSA-3108232	SUMO E3 ligases SUMOylate target proteins	CHD3, HDAC1, MDC1,NR3C1, SIN3A, SP3, SUMO1, TOP2A, TP53BP1	9	Among the proteins involved in SUMOylation, the protein inhibitor of activated STAT (PIAS) E3-ligases were initially described as transcriptional coregulators. Several components of the SUMO machinery are highly expressed in breast cancer, suggesting that SUMOylation is required to initiate or sustain tumorigenesis [69].	1.39E-06
	HSA-3700989	Transcriptional regulation by tp53	CCNA2, CCNB1, CDK2, CDK5, CHD3, HDAC1, MDC1, SGK1, SUPT5H, TAF1	10	The tumour suppressor TP53 (or P53) is a well-known gene that drives multiple cancers. Under stress conditions, it regulates the transcription of many genes involved in various cellular processes such as cellular metabolism, survival, senescence, apoptosis and DNA damage response [48, 49].	2.53E-05
	HSA-212436	Generic transcription pathway	CCNA2, CCNB1, CDK2, CDK5, CHD3, GLI3, HDAC1, MAPK3, MDC1, NR3C1, SGK1, SIN3A, SMARCA2, SUMO1, SUPT5H	16	One or more latent cytoplasmic transcription factors have increased activity in most human cancers, and in many cases prevent apoptosis of cancer cells. Necessary physical interaction among transcription factors and cofactors in the nucleus affords selective sites of potential drug action [70].	2.53E-05
	HSA-74160	Gene expression (transcription)	CCNA2, CCNB1, CDK2, CDK5, CHD3, ERCC6, GLI3, HDAC1, MAPK3, MDC1, NR3C1, SGK1, SIN3A, SMARCA2, SUMO1, SUPT5H	17		3.17E-05
	HSA-9006934	Signalling by receptor tyrosine kinases	AXL, CDK5, CTNNA1, IGF1R, INSR, MAPK3, PIK3CB, PTK2, RPS6KA1, SGK1, VAV1	11	High levels of RTKs may be associated with increased breast cancer aggressiveness and decreased overall and disease-free survival [50-55].	2.82E-05
	HSA-73894	DNA repair	APEX1, CCNA2, CDK2, ERCC6, MAPK8, MDC1, PRKDC, SUMO1, TP53BP1	9	Defective components in DNA damage and repair machinery are an underlying cause for the development and progression of different types of cancers, and breast cancer is no exception [33].	3.58E-05
	HSA-5693532	DNA double-strand break Repair	CCNA2, CDK2, MAPK8, MDC1, PRCKDC, SUMO1 ,TP53BP1	7	DNA double strand break (DSB) repair dysfunction increases the risk of familial and sporadic breast cancer [71].	3.83E-05
	HSA-5633007	Regulation of TP53 activity	CCNA2, CDK2, CDK5, CHD3, HDAC1, SGK1, TAF1	7	Similar to the function of Transcriptional Regulation by TP53 above.	4.19E-05
	HSA-1640170	Cell cycle	CCNA2, CCNB1, CDC14A, CDK2, HDAC1, MAPK3, MDC1, POLA1, TOP2A, TP53BP1, SUMO1	11	Similar to the function of Cell Cycle in KEGG.	1.30E-04
	HSA-453279	Mitotic G1 phase and G1/S transition	CCNA2, CCNB1, CDK2, HDAC1, POLA1, TOP2A	6	Oestrogen-induced mitochondrial oxidants control the early stages of cell cycle progression, which provides the basis for the discovery of new antioxidant-based drugs or antioxidant gene therapies to prevent and treat oestrogen-dependent breast cancer [72].	3.30E-04
	HSA-4420097	VEGFA-VEGFR2 pathway	AXL, CTNNA1, PIK3CB, PTK2, VAV1	5	A study suggests that ACE2, a potential resister of breast cancer, may inhibit breast cancer angiogenesis through the VEGFa/VEGFR2/ERK pathway [73].	6.90E-04
	HSA-1280215	Cytokine signalling in the immune system	CD4, HSPA8, HSPA9, MAPK3, MAPK8, PIK3CB, RPS6KA1, SUMO1, TYK2, VAV1	10	One study found dysregulated cytokine signalling in peripheral blood T cells from patients with breast cancer (BC), even those with localized disease [74].	9.00E-04
	HSA-422475	Axon guidance	ANK3, CDK5, DLG1, HSPA8, MAPK3, MAPK8, PIK3CB, PTK2, RPS6KA1	9	The frequent dysregulation of axon guidance molecules (AGMs) expression during tumorigenesis and tumour progression suggests that AGMs also play a crucial role as tumour suppressors and oncogenes in breast cancer [75].	9.70E-04

Disease level. In addition to the "cancer-type level" analysis discussed above, we found in the "disease level" analysis that ten of the 51 driver candidates were enriched in three BC-related diseases, including type 2 diabetes, obesity, and plasma HDL cholesterol (HDL-C) (see details in Table S2). Reportedly, up to 16% of BC patients have diabetes, and type 2 diabetes may be associated with a 10-20% increased relative risk of BC [56]. In addition, a recent study suggested that type 2 diabetes accelerates the paracrine effects of AT-MSCs to induce the

migration of breast cancer cells (BCCs) and upregulate migration and EMT-related factors in BCCs [57]. Obesity has long been recognized as an important determinant of the progression of BC and mortality [76]. In fact, obesity increases the risk of cancer recurrence and death and, in particular, accelerates and exacerbates the metastatic progression of breast cancers [77-82]. Moreover, patients with breast cancers and obesity are up to 46% more likely to have distant metastases 10 years after diagnosis [78]. Several studies have shown that HDL cholesterol has anti-inflammatory properties that may be associated with an increased risk of breast cancer [30, 83-88].

Validation-required level. Five genes were identified at this level. The five genes were ranked high according to DriverMP, but their direct relationship with BC was unclear based on this study. Future investigations may reveal the associations of these genes with breast cancers.

To visualize the relationship among the top four groups of candidate drivers, we drew a Venn graph and found that the IGF1R gene appeared in all four groups (Fig. 7d). Based on the specificity of IGF1R, we reviewed the literature and found that examinations of various tumours showed abundant expression of IGF1R, which suggests that upregulation of the IGF1R gene constitutes a common paradigm in different types of cancer [89-91]. Some experimental studies have also suggested that the IGF1R gene is a downstream target of the BRCA1 gene [92-94]. This evidence indicates that IGF1R is highly likely to be a typical driver of BC.

DriverMP reliably predicts novel drivers for lung adenocarcinoma

Lung cancer is the top cause of cancer-related death and is histologically classified into small cell lung cancer (SCLC) and non-small cell lung cancer (NSCLC) [95]. NSCLC accounts for approximately 85% of all lung cancer diagnoses, with the majority of patients presenting with lung adenocarcinoma (LAC or LUAD) [96]. In this section, we collected 60 novel driver candidates for lung adenocarcinoma and grouped them according to the five analysis levels (**Table 4**).

Table 4. Five-level assessment yielded 60 driver candidates of lung adenocarcinoma.

No.	Level of analysis	Driver candidates	Count	Percentage
1	Cancer-type level	ABCB1, BARD1, CASP3, CCNA2, CDC25C, CHEK1, ERCC6, IGF1R, IRS1, PLK1, PRKDC, STAT1, SUMO1, TP53BP1	14	23.33%

2	Literature-supported level	BARD1, CDC25C, CHEK1, HDAC1, IRS1, KAT2B, LYN, MDC1, PLK1, PRKCB, SP1, SUMO1, TLR4, TOP2A, USP7	15	25.00%
3	Pathway level	ABCB1, ACTB, ANK2, BARD1, CASP1, CASP3, CCNA2, CDC14A, CDC25C, CHD3, CHEK1, CSNK1D, CSNK2A1, E2F4, EGR1, ERCC6, FLT1, GAPDH, GLI2, GLI3, GRB2, HDAC1, HSPA8, IGF1R, IRS1, KAT2B, LYN, MAPK9, MDC1, MED1, NR3C1, PIK3CG, PLK1, POLA1, PPP3CA, PRKCA, PRKCB, PRKDC, PTK2B, SIN3A, SMARCA2, SOS1, SP1, STAT1, SUMO1, TAF1, TLR4, TNC, TOP2A, TOP2B, TP53BP1, USP7, VCAN	53	88.33%
4	Disease level	ABCB1, BARD1, CASP3, CCNA2, CDC25C, CHEK1, ERCC6, IGF1R, IRS1, PLK1, STAT1, TLR4	12	20.00%
5	Validation-required level	CAD, HOXA5, HSPA5, NEB, RYR1, RYR2, TTN	7	11.67%

Cancer-type and disease levels. Similar to the process in breast cancer analysis, disease enrichment showed that 47 (78.3%) of these 60 candidates were included in “cancer” ($p = 2.6 \times 10^{-18}, FDR = 4.7 \times 10^{-17}$), and 14 (23.3%) and 12 (20.0%) of them were enriched in “lung cancer” ($p = 3.5 \times 10^{-7}, FDR = 6.5 \times 10^{-5}$) and “chronic obstructive pulmonary disease” ($p = 1.0 \times 10^{-6}, FDR = 1.7 \times 10^{-4}$), respectively (Fig. 8a), which comprise the candidate genes at the “cancer-type level” and “disease level” (see details in Table S3).

For the specific cancer-type level analysis, we found that five of the candidate genes were enriched in the “cell cycle” pathway (KEGG, $FDR = 6.3 \times 10^{-6}$), and six were enriched in the “DNA double-strand break repair” pathway (Reactome, $FDR = 7.0 \times 10^{-7}$), both of which are associated with cancer progression (Fig. 7a). Regarding the disease level analysis, it has been reported that people suffering from chronic obstructive pulmonary disease (COPD) are at higher risk of developing lung cancer than those without COPD and that these patients are more susceptible to poor outcomes after diagnosis and treatment; COPD could be the driving factor for lung cancer, providing a conducive environment for cell propagation and evolution [97, 98].

Literature-supported candidate drivers. This level of analysis yielded 15 genes supported by three different types of evidence, as shown in Table 5. For example, PLK1 is a key mitotic kinase that is overexpressed in various cancers, including NSCLC, and drives cancer growth [99, 100]. Sp1 levels accumulate strongly in the early stage and then decline in the late stage, which is important for lung cancer cell proliferation and metastasis during tumorigenesis [101].

Table 5. Fifteen driver candidates identified in the “literature-supported level” of analysis of lung adenocarcinoma.

Gene	NCBI Entrez ID	Rank (HumanNet)	Rank (STRINGv10)	Function	Type
CHEK1	1111	31	179	Recently, integrated bioinformatics analysis showed that CHEK1 may be a critical gene in the development and prognosis of NSCLC. In addition, it has been suggested that CHEK1 expression was increased in NSCLC, compared with adjacent normal tissues [102, 103].	O
PLK1	5347	42	50	Polo-like kinase 1 (PLK1) is a critical mitotic kinase that is overexpressed in various cancers including NSCLC and drives cancer growth [99, 100].	E&B
CDC25C	995	80	213	CDC25C may predict poor prognosis and may have important roles in the regulation of S-phase and M/G1 phase of the cell cycle as well as the FAS-mediated apoptosis in LUAD [104].	B
IRS1	3667	113	169	Recent studies have analysed the biological impact of newly identified mutations within the IRS1 gene and suggested that these mutations may be diagnostic markers for lung cancer [105].	E
SUMO1	7341	139	192	SUMO1 promotes the proliferation and invasion of NSCLC cells by regulating NF-Kb [106].	E
BARD1	580	244	191	BARD1 isoforms might be involved in tumour initiation and invasive progression and might represent a novel prognostic marker for NSCLC [107].	E
KAT2B	8850	30	72	Recent study has demonstrated that KAT2B expression positively correlated with the outcomes of patients with lung adenocarcinoma. Additionally, KAT2B was synergistic with multiple immune cells infiltration and immune checkpoints in NSCLC [108].	B
USP7	7874	32	74	Intervention in USP7 to induce the downregulation of Ki-67 protein could inhibit proliferation of NSCLC cells and even increase the sensitivity of the cells to some chemotherapy drugs [109].	E
TOP2A	7153	52	91	Study revealed that TOP2A was highly expressed in lung adenocarcinoma compared with matched adjacent normal tissues and high expression of TOP2A was associated with poor prognosis for LUAD patients [110].	E&B
SP1	6667	69	163	Sp1 level accumulated strongly in early stage and then declined in late stage, which is important for lung cancer cell proliferation and metastasis during tumorigenesis [101].	E
TLR4	7099	75	29	TLR4 is highly expressed in NSCLC tumour cells and strongly correlate with malignant tumour phenotypes. TLR4 ligation promotes the secretion of immunosuppressive cytokines TGF-β, VEGF and proangiogenic chemokine IL-8 from human lung cancer cells [111, 112].	E
LYN	4067	145	98	Lyn regulates activation of epidermal growth factor receptors in lung adenocarcinoma cells. Specifically, Lyn is involved in the EGFR signalling pathway, and inhibition of its expression can reduce EGFR activation and cell viability [113].	E
PRKCB	5579	155	107	Recent study showed that PRKCB is relevant to prognosis of LUAD through methylation and immune infiltration [114].	B
HDAC1	3065	170	176	The results in a meta-analysis suggest that HDAC1 may serve as a good diagnostic and prognostic marker for lung cancer [115].	B
MDC1	9656	208	83	MDC1 plays important roles in tumour formation, progression, and treatment. In addition, MDC1 expression was assessed by immunohistochemistry (IHC) in lung tumours and was found to be commonly expressed in benign tissues, but reduced or lost in 26% of lung cancer samples [116, 117].	O

Pathway level. In total, 53 of the 60 driver candidates were identified as pathway-related driver candidates. We performed a pathway enrichment analysis on these 60 driver candidates and identified 16 and 11 pathways associated with lung adenocarcinoma or non-small cell lung cancer in KEGG and Reactome, respectively (Table 6). For instance, we found that 9 (15.0%) and 9 (15.0%) of the 60 candidates were enriched in "Focal adhesion" ($FDR = 6.5 \times 10^{-7}$) and "PI3K-Akt signalling pathway" ($FDR = 1.7 \times 10^{-5}$) against KEGG (Fig. 8b), respectively, both of which are highly related to LAC. Several studies have suggested that focal adhesion kinase (FAK) expression is frequently upregulated in different types of cancer, including NSCLC, and a great number of studies have focused on either reducing FAK expression or activity to inhibit the growth and metastatic capacities of tumours [118, 119]. The PI3K/AKT

pathway has been reported as an emerging source of lung cancer aggressiveness [120]. We also found that 13 (21.7%) and 12 (20.0%) of the 60 genes were enriched in “signalling by receptor tyrosine kinases” ($FDR = 8.5 \times 10^{-7}$) and “transcriptional regulation by TP53” ($FDR = 3.8 \times 10^{-5}$), respectively, according to the Reactome database (Fig. 8c). Receptor tyrosine kinases (RTKs), such as EGFR, the classical driver of lung cancer, are important components of the cellular signalling apparatus and are frequently mutated or otherwise dysregulated in NSCLC [121]. The function of the “transcriptional regulation by TP53” pathway has already been discussed in the analysis of breast cancer. As described before, TP53 (or P53) is a well-known gene that drives multiple cancers, including lung adenocarcinoma [48].

Validation-required level. A total of seven genes were identified in this level of analysis, and their associations with LAC may be revealed in future investigations.

Table 6. Biological pathways associated with lung adenocarcinoma.

Database	Pathway ID	Pathway	Genes	Count	Function	FDR
KEGG	hsa05206	MicroRNAs in cancer	ABCB1, CASP3, CDC25C, GRB2, HDAC1, IRS1, PRKCA, PRKCB, SOS1, TNC	10	Dysregulation of miRNA expression often appears in many cancers such as lung cancer, breast cancer and cervical cancer, and is directly associated with tumour initiation, progression, and metastasis [122].	3.00E-08
	hsa04110	Cell cycle	CCNA2, CDC14A, CDC25C, CHEK1, E2F4, HDAC1, PLK1, PRKDC	8	Cell cycle deregulation is a common feature of human cancer. Cancer cells frequently display uncontrolled proliferation, genomic instability (increased DNA mutations and chromosomal aberrations) and chromosomal instability (changes in chromosome number) [123].	5.32E-07
	hsa04510	Focal adhesion	ACTB, FLT1, GRB2, IGF1R, MAPK9, PRKCA, PRKCB, SOS1, TNC	9	Several studies have suggested that focal adhesion kinase (FAK) is frequently upregulated in different types of cancer, including NSCLC, and a great number of studies have focused on either reducing FAK expression or activity to inhibit the growth and metastatic capacities of tumors [118, 119].	6.48E-07
	hsa04912	GnRH signalling pathway	EGR1, GRB2, MAPK9, PRKCA, PRKCB, PTK2B, SOS1	7	GnRH and GnRH-R are expressed in several types of cancer tissues, including NSCLC, indicating that the expression of GnRH may be associated with tumour progression [124].	6.78E-07
	hsa04010	MAPK signalling pathway	CASP3, FLT1, GRB2, HSPA8, IGF1R, MAPK9, PPP3CA, PRKCA, PRKCB	10	MAPK pathway affects decisive roles in the carcinogenesis and treatment resistance of NSCLC cells by promoting proliferation or inhibiting apoptosis of NSCLC cells [125].	7.26E-07
	hsa04935	Growth hormone synthesis, secretion and [illegible]	GRB2, IRS1, MAPK9, PRKCA, PRKCB, SOS1, STAT1	7	Both humans and mice lacking functional growth hormone (GH) receptors are known to be resistant to cancer. A growth hormone receptor SNP promotes lung cancer by impairment of SOCS2-mediated degradation [126].	3.20E-06
	hsa04650	Natural killer cell mediated cytotoxicity	CASP3, CCNA2, CHEK1, GRB2, HDAC1, KAT2B, LYN, USP7	8	CD48-positive NSCLC cells might be susceptible to NK cell–mediated cytotoxicity, which provide information on how to stratify NSCLC patients potentially responsive to NK-cell therapy [127].	3.20E-06
	hsa04066	HIF-1 signalling pathway	FLT1, GAPDH, IGF1R, PRKCA, PRKCB, TLR4	6	CD39/CD73 upregulation on myeloid-derived suppressor cells via TGF-β-mTOR-HIF-1 signalling in patients with non-small cell lung cancer [128].	1.58E-05
	hsa04151	PI3K-Akt signalling pathway	FLT1, GRB2, IGF1R, IRS1, PIK3CG, PRKCA, SOS1, TLR4, TNC	9	Currently, PI3K/AKT/mTOR signalling has been reported as an emerging source of lung cancer aggressiveness. The development of therapies targeting PI3K/AKT/mTOR signalling is receiving extensive attention from researchers and new drugs continue to be discovered [120].	1.74E-05
	hsa04150	mTOR signalling pathway	GRB2, IGF1R, IRS1, PRKCA, PRKCB, SOS1	6		7.58E-05
	hsa04062	Chemokine signalling pathway	GRB2, LYN, PIK3CG, PRKCB, PTK2B, SOS1, STAT1	7	Axl-highly expressing lung adenocarcinomas exhibit higher expressions of multiple genes encoding immune checkpoint molecules and chemokines/chemokine receptors [129].	2.38E-05
	hsa01521	EGFR tyrosine kinase inhibitor resistance	GRB2, IGF1R, PRKCA, PRKCB, SOS1	5	Non-small cell lung cancer patients with activating epidermal growth factor receptor (EGFR) mutations typically benefit from EGFR tyrosine kinase inhibitor treatment [130].	5.55E-05
	hsa04014	Ras signalling pathway	FLT1, GRB2, IGF1R, MAPK9, PRKCA, PRKCB, SOS1	7	The Ras proteins are pivotal regulators of cellular proliferation, differentiation, motility, and apoptosis. Mutations on the K-ras gene have been found in 20%–30% of non–small-cell lung cancers and are believed to play a key role in this malignancy [131].	6.60E-05
	hsa04012	ErbB signalling pathway	GRB2, MAPK9, PRKCA, PRKCB, SOS1	5	Evidence is now accruing that EGFR works in concert with other ErbB family members, particularly HER2 and ErbB3, to activate these signalling pathways in lung cancers [132].	6.78E-05
	hsa05223	Non-small cell lung cancer	GRB2, PRKCA, PRKCB, SOS1	4	NSCLC accounts for approximately 85% of all lung cancer diagnoses, with the majority of patients presenting with lung adenocarcinoma [95, 96].	4.60E-04
	hsa04310	Wnt signalling pathway	CSNK2A1, MAPK9, PPP3CA, PRKCA, PRKCB	5	Available data indicate that Wnt signalling substantially impacts NSCLC tumorigenesis, prognosis, and resistance to therapy, with loss of Wnt signalling inhibitors by promoter hypermethylation or other mechanisms appearing to be particularly important [133].	7.60E-04
Reactome	HSA-1640170	Cell cycle	BARD1, CCNA2, CDC14A, CDC25C, CHEK1, CSNK1D, CSNK2A1, E2F4, HDAC1, LYN, MDC1, POLA1, PRKCA, PRKCB, PLK1, SUMO1, TOP2A, TP53BP1	18	Similar to the function of in the cell cycle KEGG pathway.	6.05E-10
	HSA-69278	Cell cycle, mitotic	CCNA2, CDC14A, CDC25C, CSNK1D, CSNK2A1, E2F4, HDAC1, LYN, PLK1, POLA1, PRKCA, PRKCB, SUMO1, TOP2A	14		1.95E-07
	HSA-212436	Generic transcription pathway	BARD1, CASP1, CCNA2, CDC25C, CHD3, CHEK1, CSNK2A1, E2F4, GLI2, GLI3, HDAC1, KAT2B, MDC1, MED1, NR3C1, PRKCB, SIN3A, SMARCA2, SP1, STAT1, SUMO1, TAF1, USP7	23	Mutated or dysregulated transcription factors represent a unique class of drug targets that mediate aberrant gene expression, including blockade of differentiation and cell death gene expression programmes, hallmark properties of cancers [134].	6.05E-10
	HSA-162582	Gene expression (transcription)	ACTB, BARD1, CASP1, CCNA2, CDC25C, CHD3, CHEK1, CSNK2A1, E2F4, ERCC6, GLI2, GLI3, HDAC1, KAT2B, MDC1, MED1, NR3C1, PRKCB, SIN3A, SMARCA2, SP1, STAT1, SUMO1, TAF1, USP7	25		6.05E-10
	HSA-3700989	Transcriptional regulation by TP53	BARD1, CASP1, CCNA2, CDC25C, CHD3, CHEK1, CSNK2A1, E2F4, HDAC1, MDC1, TAF1, USP7	12	The function of this pathway has been mentioned in the section on breast cancer.	3.78E-07
	HSA-597592	Post-translational protein modification	ACTB, ANK2, BARD1, CCNA2, CHD3, CSNK1D, HDAC1, HSPA8, KAT2B, MDC1, NR3C1, PRKDC, SIN3A, SUMO1, TNC, TOP2A, TOP2B, TP53BP1, USP7, VCAN	20	In the last few years, several therapeutic design options focusing on specific kinases or phosphatases dysregulated in lung cancer progression have been developed with various levels of success [135].	7.87E-07
	HSA-9006934	Signalling by receptor tyrosine kinases	ACTB, CHEK1, EGR1, FLT1, GRB2, IGF1R, IRS1, LYN, PRKCA, PRKCB, PTK2B, SOS1, STAT1	13	Receptor tyrosine kinases (RTKs) are important components of the cellular signalling apparatus and are frequently mutated or otherwise dysregulated in NSCLC, such as EGFR, the classical driver of lung cancer [121].	8.47E-07
	HSA-73894	DNA repair	ACTB, BARD1, CCNA2, CHEK1, ERCC6, MDC1, PRKDC, SUMO1, TP53BP1, USP7	10	DNA repair pathways can enable tumour cells to survive DNA damage that is induced by chemotherapeutic treatments; therefore, inhibitors of specific DNA repair pathways might prove efficacious when used in combination with DNA-damaging chemotherapeutic drugs [136].	6.82E-06
	HSA-1433559	Regulation of KIT signalling	GRB2, LYN, PRKCA, SOS1	4	miR-1260b, mediated by YY1, activates KIT signalling by targeting SOCS6 to regulate NSCLC cell proliferation and apoptosis, and is a potential biomarker and therapeutic target for NSCLC [137].	4.45E-05
	HSA-1280215	Cytokine signalling in the immune system	CASP1, CASP3, EGR1, GRB2, HSPA8, IRS1, LYN, MAPK9, PTK2B, SOS1, STAT1, SUMO1	12	TLR4 expressed on human lung cancer cells is functionally active and may play important roles in promoting immune escape of human lung cancer cells by inducing immunosuppressive cytokines and apoptosis resistance [111].	8.90E-05
	HSA-194138	Signalling by VEGF	ACTB, FLT1, PTK2B, PRKCA, PRKCB	5	In lung cancer, VEGF plays a significant role in establishing a vascular supply within the tumor [138].	9.40E-04

Notably, the Venn diagram of candidate drivers at the four levels showed that the IRS1, BARD1, CDC25C, CHEK1 and PLK1 genes were present in the results for the top four levels of analysis (Fig. 8d). In fact, they are closely related to LAC or NSCLC. Novel mutations in IRS1 in non-small cell lung cancer tumour samples (p.S668T and p.D674H) play an important role in the phenotype of lung cancer [105]. Isoforms of BARD1 might be involved in tumour initiation and invasive progression and represent a novel prognostic marker for NSCLC [107]. CDC25C predicts a poor prognosis in LUAD and may function in cell cycle regulation and FAS-mediated apoptosis [104]. CHEK1 may be a critical gene related to NSCLC development and prognosis [102, 103]. The relationship between PLK1 and NSCLC was mentioned above.

Genes identified at the cancer-type level exhibit high densities in PPI networks

Genes identified at the cancer-type level demonstrated direct relationships with specific cancer types and had great potential to drive the development of corresponding cancers. To deeply investigate the relationships among these genes, we analysed the density of the subnetwork of these genes from the PPI network.

By analysis, we found that the subnetwork of the genes at the cancer-type level demonstrated extremely high densities in all ten cancer types (see Fig. 6a and Fig. 7a for breast cancer and lung adenocarcinoma and supplemental materials for the other eight cancer types). Specifically, the subnetwork of the genes in the cancer-type level of breast cancer from the STRINGv10 network had 74 edges, and the sum of edge weights was 35.35. For lung adenocarcinoma, the subnetwork of the genes at the cancer-type level had 72 edges, and the sum of edge weights was 31.77. However, when the subnetwork was constructed from 14 genes of 1,000,000 random selections, the average number of edges and the average sum of edge weights were only 2.19 and 0.65, respectively, and none of the 1,000,000 selections constructed a subnetwork with a higher density. This phenomenon may be consistent with the view that “mutations in the cancer genome tend to converge in a few biological pathways” [18] and “genes acting together in various signalling and regulatory pathways and protein complexes is a prominent explanation for the heterogeneity of cancer mutations” [17].

DriverMP: A user-friendly online service and a database of novel drivers

To make the DriverMP tool more convenient for users, we developed a user-friendly web server available at http://liulab.top/DriverMP/server (Fig. 9a). After entering the web server, users only need to upload three datasets [a mutation dataset and two gene expression datasets (tumour and normal)] for a cancer type and press the “submit” button to start the running of DriverMP. The current version of DriverMP provides the HumanNet and STRINGv10 PPI networks, and more PPI networks will be added in the future. After running DriverMP, the results will be directly displayed on the web page and can also be downloaded from our page (Fig. 9b).

In addition to the online web server, we developed a database (available at http://www.liulab.top/DriverMP/table) of those novel driver genes that are strongly supported by clinical experiments, disease enrichment analysis, or biological pathway analysis. After entering the database, users can not only search for the genes of interest to discover their related cancer types but also explore cancer types of interest to obtain novel drivers. The relationships between the genes and the corresponding cancer types are detailed in the database (Fig. 10). More cancer types will be added, and the novel drivers in the database will also be continuously updated in the future.

Discussion

Over the past few years, it has been a major issue in cancer genetics to identify drivers from the large number of passenger mutations. Some frequency-based computational methods have made great attempts but failed to discover the real drivers that are buried in the long tail due to the preference for highly mutated genes and the reliance on sample size. With the discovery that driver mutations tend to be enriched in signalling and regulatory pathways and affect alterations in gene expression in the biological subnetworks or pathways associated with them, many algorithmic methods using PPI networks, biological pathways, or gene expression data have provided diverse solutions for the prediction of drivers, but their predictive effects, both in terms of sensitivity and specificity, are far from satisfactory.

In this study, we developed a new method, DriverMP, for the identification of driver mutations by effectively integrating the advantages of multiple kinds of data analysis methods. After evaluating the performance of DriverMP and comparing it with other leading predictors, it consistently demonstrated much higher prediction accuracy based on known driver genes in

CGC. Moreover, DriverMP demonstrated strong robustness in identifying drivers present at low frequencies even when many samples were removed, a finding which greatly contributes to overcoming the challenge of the long-tail dilemma. For the top prioritized candidates (termed novel driver candidates) identified by DriverMP that are not included in CGC, we proposed a cancer-specific five-level assessment method to comprehensively evaluate the reliability of the novel driver candidates by applying various analytical approaches, such as disease enrichment analysis, literature investigation, and biological pathway enrichment analysis. We also provide a catalogue of potentially novel driver mutations with high ranks according to DriverMP and strong literature evidence for ten common cancer types, which provide a reference for future studies on cancer mechanisms. Moreover, we offer a user-friendly online web service for researchers to analyse their own data using DriverMP. The superiority of DriverMP may be attributed to the following innovations.

First, based on our observation that most driver genes have codriver neighbours in the PPI network, we pioneered a method for the prediction of drivers based on mutation pairs, which is one of the key innovations and theoretically captures the cancer-driven characteristics in a more realistic pattern. Second, we redefined a mutation score for each gene by normalizing the mutation matrix according to the mutation frequency of each gene, based on which the contributions of different samples with different numbers of mutated genes can be effectively balanced. Third, according to the theory that genes affected by driver mutations and their neighbours are simultaneously differentially expressed, we developed a differential expression network to quantify the differential expression level of each gene and the associations between two neighbours. Based on the assumption that driver mutations tend to converge into meaningful biological subnetworks, we extracted a subnetwork from the PPI network centred on each pairwise mutation. Then, based on the differential expression network and PPI subnetwork, an impact score for each pairwise mutation was generated by combining the network topology relationships of the mutation pair.

Despite the obvious advantages of DriverMP, there is still much room for further improvement. For example, the current version of DriverMP only accepts three data types, including mutation data, PPI network, and gene expression data. However, other types of data, e.g., DNA methylation and proteomics data, can also be effectively applied to further improve

the prediction performance. In addition, the current version of DriverMP can only predict driver mutations for a specific cancer type and not for a specific sample. In the future version of DriverMP, we will attempt to solve these problems, add relative functions, and make further improvements.

To our knowledge, DriverMP is the first driver gene predictor that prioritizes mutation pairs by constructing two differential expression networks and PPI subnetworks centred on each pairwise mutation and then combining the topology relationship of the two networks. The tool has been developed to be user-friendly and is expected to play a crucial role in new discoveries of developments, mechanisms, diagnosis, and treatments of cancers.

Methods

Datasets and gold-standard set

We analysed ten popular cancer types from the TCGA, including breast invasive carcinoma (BRCA), prostate adenocarcinoma (PRAD), lung adenocarcinoma (LUAD), lung squamous cell carcinoma (LUSC), kidney clear cell carcinoma (KIRC), kidney renal papillary cell carcinoma (KIRP), head and neck squamous cell carcinoma (HNSC), colon adenocarcinoma/rectum adenocarcinoma oesophageal carcinoma (COADREAD), uterine corpus endometrial carcinoma (UCEC) and bladder urothelial carcinoma (BLCA). The somatic nonsilent mutation data and the gene expression data were downloaded from the UCSC Browser database [139] (https://xenabrowser.net/datapages/) and TCGA website (https://portal.gdc.cancer.gov/), respectively, and PPI networks generated via two commonly used tools, STRING and HumanNet, were downloaded from their official websites https://cn.string-db.org/ and https://staging2.inetbio.org/humannetv3/, respectively. The edge weights (representing the interaction strength between two proteins) of both PPI networks were normalized to values between 0 and 1 by dividing by the largest weight, and self-loops of nodes were removed. Then, a PPI network is represented as a weighted graph G_{PPI}.

Since the real cancer genes is unavailable for the analysed cancer types, we collected all 616 known cancer driver genes from the most widely used database, the Cancer Genome Census (CGC), to evaluate the performance of DriverMP and all the compared approaches.

Evaluation criteria

The performance of a cancer gene identifier was evaluated by the following criteria. (i) The receiver operation characteristics (ROC) analysis and area under the ROC curve (AUC) values for uncovering known driver mutations. The ROC and AUC criteria were used to evaluate the overall sensitivity and specificity of mutation prioritization. (ii) The curve of the numbers of identified known driver mutations in the top ranked 1, 2, ..., and *N* candidate genes. (iii) The curve of the F1-scores in the top ranked 1, 2, ..., and *N* candidate genes and the corresponding AUFC (Area Under F1-score Curve) values. In practical applications, only the top-ranked mutations have a chance to be validated by the follow-up experiments. For this reason, only the top ranked 500 candidate mutations for each compared approach were selected for comparing the numbers of identified known driver mutations, the F1-score, and AUFC. The definition of F1-score in the top ranked *K* genes is displayed as follows.

$$F1\_score = \frac{2 \times Presicion \times Recall}{Presicion + Recall}$$

where the precision and recall are defined as follows:

$$Precision = \frac{\#\{genes\ ranked\ before\ Kth\} \cap \{known\ driver\ genes\}}{\#\{genes\ ranked\ before\ Kth\}}$$

$$Recall = \frac{\#\{genes\ ranked\ before\ Kth\} \cap \{known\ driver\ genes\}}{\#\{known\ driver\ genes\}}$$

Calculation of mutation scores based on mutation frequency

Given a somatic mutation matrix $A = (a_{ij})_{m\times n}$ of a cancer type with *m* genes and *n* samples, the value $a_{ij} = 1$ if gene *i* has at least one non-silent somatic mutation in sample *j*, and $a_{ij} = 0$ otherwise. According to mutation matrix *A*, the mutation frequency *N*(*i*) for each gene *i* can be directly calculated as the number of samples that have a mutation in gene *i*. However, different samples usually have different numbers of mutated genes and therefore contribute unequally to the calculation of mutation frequency. To resolve the biases of different samples, we first normalized the values of the mutation matrix *A*, based on which a mutation score *M*(*i*) was defined for each gene *i*.

(1) Normalization of the mutation matrix. To balance the contribution of different samples, the mutation matrix *A* was first normalized to a new matrix $A' = (a'_{ij})_{m\times n}$ as follows.

$$a'_{ij} = \frac{N(i)}{\sum_{gene\ k \in G(j)} N(k)}$$

where $G(j)$ represents the set of mutated genes in sample j. Based on the above definition, the sum of the normalized mutations for each sample is 1.

(2) Calculation of mutation scores. Based on the normalized mutation matrix A', a mutation score $M(i)$ for each gene i is defined by the following formula.

$$M(i) = \sum_{j=1}^{n} a'_{ij}$$

Data preparation and the selection of major mutations

(1) Gene filtering. A gene was removed if it satisfied at least one of the following conditions: (i) the gene was not expressed in at least one sample of the tumour or normal expression matrix; (ii) the gene was not mutated in any sample; and (iii) the gene was not covered in the PPI network. The remaining genes form a gene set S_{remain}.

(2) Preprocess of gene expression data. The two tumour and normal expression matrices with rows and columns represent genes and samples, respectively. To quantify the differential expression between tumour and normal samples, two matrices are processed as follows: (i) the genes are removed if they are not contained in the set S_{remain}; (ii) the samples are removed if they are only included in one of the two matrices. The processed tumour and normal expression matrices M_{tumor} and M_{normal} have the same remaining genes and samples. Then, for each gene i, the differential level $\delta(i)$ between tumour and normal expression values is calculated as follows.

$$\delta(i) = \|M_{tumor}(i) - M_{normal}(i)\|_2$$

where $M_{tumor}(i)$ and $M_{normal}(i)$ represent the i-th rows of the two matrices M_{tumor} and M_{normal}, *respectively*.

(3) Selection of major mutations. Based on the observations that driver mutations tend to be strongly associated with other mutations in the PPI network and exhibit differential tumour/normal expression, we selected the major mutations for further analysis by the following two steps.

Step 1. A maximum neighbour weight $W_{max}(i)$ in the PPI network was defined for each gene

i in S_{remain} as the largest interaction weight between gene i and its interacting neighbors. The top 30% of genes in S_{remain} with the largest maximum neighbour weights were selected and formed a gene set G_{PPI}.

Step 2. According to gene differential expression, the top 4% of genes in S_{remain} with the highest differential levels between tumour and normal expression values were selected and formed another gene set, S_{diff}.

The set of major mutations S_{major} was then defined as $S_{major} = S_{PPI} \cup S_{diff}$. Accordingly, the genes that were not included in S_{major} were removed from the two expression matrices M_{tumor} and M_{normal} with S rows and L columns after removal.

Construction of the differential expression network

In this section, a new network named the differential expression network G_{diff} was constructed to quantify the correlation between each pair of mutations in terms of differential expression. The nodes of the network represent the genes in S_{major}, while the edges between two nodes and the node and edge weights were defined as follows.

(1) Generation of the differential expression matrix and calculation of node weights. Based on the two expression matrices M_{tumor} and M_{normal}, the expression values were first log transformed, and then the differential expression value $M_{diff}(i,j)$ of gene i on sample j between tumour and normal was calculated by the following formula.

$$M_{diff}(i,j) = log_2[1 + M_{tumor}(i,j)]\text{-}log_2[1 + M_{normal}(i,j)]$$

Then, *z-score* normalization was performed on M_{diff} for each gene followed by an absolute value operation.

$$\widetilde{M}_{diff}(i,j) = \left|\frac{M_{diff}(i,j) - mean[M_{diff}(i)]}{\sigma_i}\right|$$

where $mean[M_{diff}(i)]$ and σ_i represent the average value and standard deviation of the i-th row of M_{diff}, respectively. The matrix $\widetilde{M}_{diff}$ is defined as the differential expression matrix between the tumour and normal expression matrices M_{tumor} and M_{normal}. Then, the differential expression score $W_{diff}(v_i)$ of gene i is defined as the average differential expression values of gene i on all L samples, which is also assigned as the node weight of gene i.

$$W_{diff}(v_i) = \frac{1}{l}\sum_{j=1}^{l} \widetilde{M}_{diff}(i,j)\, , i = 1,2,\cdots,S$$

(2) **Generation of network edges and calculation of edge weights.** Motivated by WGCNA17, which is an expression clustering analysis method that aims to find co-expressed gene modules, we improved it to better adapt to the construction of the differential expression network by the following two steps.

Step 1. Similar to WGCNA, a Pearson correlation coefficient c_{ij} was calculated for a pair of genes *i* and *j* in the differential expression matrix $\widetilde{M}_{diff}$ to measure the correlation of the two genes in terms of differential expression. A positive correlation between two genes represents a similar pattern of differential expression, and therefore, an edge was added between the two genes in the network. However, unlike WGCNA, a negative correlation between two genes was considered to indicate that the two genes demonstrated different patterns of differential expression, and no edge was added between the two genes.

Step 2. Similar to WGCNA, it is supposed that the degrees of nodes in the differential expression network should obey a power-law distribution (or long-tailed distribution). To achieve this, an appropriate power α_0 was calculated and added to the Pearson correlation coefficients. Specifically, the degree $D_i(\alpha_0)$ of node *i* under power α_0 is defined as follows.

$$D_i(\alpha) = \sum_{j \in N(i)} (c_{ij})^{\alpha_0}$$

where *N*(*i*) represents the neighbour set of node *i*.

To search for an optimal power α_0 that makes the degrees of nodes under α_0 obey a power-law distribution, we exhausted the selections of $\alpha = 1, 2, \cdots, 6$. For each α, the degree values of nodes were ordered and then equally divided into 10 intervals, and the median degrees were selected as the degrees of the intervals, which were denoted as $\{k^1, k^2, \cdots, k^{10}\}$. Meanwhile, the frequencies of genes in the 10 intervals were represented as $\{f(k^1), f(k^2), \cdots, f(k^{10})\}$. Then, a linear regression was fitted between $\{log_{10}k^1, log_{10}k^2, \cdots, log_{10}k^{10}\}$ and $\{log_{10}f(k^1),\ log_{10}f(k^2), \cdots, log_{10}f(k^{10})\}$ with a coefficient of determination R^2. The α generating the largest R^2 was selected as the optimal power α_0. Based on the updated Pearson correlation coefficients, an α_0-topological correlation coefficient $W_{diff}(e_{ij})$ for each edge e_{ij} was calculated by applying the topological

relationship of the genes in the network using the following formula, which is also assigned as the edge weight of edge e_{ij}.

$$W_{diff}(e_{ij}) = \frac{\sum_{u \in N(i) \cap N(j)} (c_{iu})^{\alpha_0} (c_{uj})^{\alpha_0} + (c_{ij})^{\alpha_0}}{\min\left(D_i(\alpha_0),\ D_j(\alpha_0)\right) + 1 - (c_{ij})^{\alpha_0}}$$

Calculation of impact scores for paired mutations

In this section, an impact score for each paired mutation will be calculated to determine the rank of the mutation pair in driving cancers, which was achieved by applying the mutation scores and topological properties from both the PPI and differential expression networks.

Given a mutation score $M(i)$ for each gene i, a PPI network G_{PPI}, and a differential expression network G_{diff}, two mutations i and j that are connected by an edge in both the PPI and differential expression networks are defined as a mutation pair $p = p(i, j)$. A gene k is defined as a neighbour of mutation pair p in G_{PPI} (or G_{diff}) if at least one of the two edges e_{ki} and e_{kj} exists. If only one of the two edges exists, the effect score $E(k, p)$ of gene k on mutation pair p is defined as the corresponding edge weight, while the maximum score is defined if both edges exist. Based on the above definitions, a sub-network *sub*-G_{PPI} (or *sub*-G_{diff}) centred on mutation pair p was constructed with nodes and edges denoting the mutation pair p and its neighbors and their connections, and the edge weights representing the effect scores of the neighbors on the mutation pair p. The association strength of the network *sub*-G_{PPI} and the differential level of the network *sub*-G_{diff} were then calculated and combined to generate an impact score of the mutation pair p.

(1) Calculation of the association strength of the network *sub*-G_{PPI}. Given the network *sub*-G_{PPI} centred on a mutation pair $p = p(i, j)$, the association strength $AS(p, k)$ between p and its neighbour k was calculated by the following formula.

$$AS(p,k) = \begin{cases} \dfrac{M(i)M(j)M(k)}{(d_{ij} \cdot d_{pk})^2}, if\ N_p^{PPI} \neq \emptyset \\ \dfrac{M(i)M(j)M_{min}}{(d_{ij} \cdot d_{max})^2},\ if\ N_p^{PPI} = \emptyset \end{cases}$$

where $M(i)$ represents the mutation score of gene i, N_p^{PPI} represents the set of neighbors of p in G_{PPI}, d_{ij} and d_{pk} are the reciprocals of the edge weights $W_{PPI}(e_{ij})$ and $E(k, p)$, M_{min} is the minimum mutation score among all the mutations, and d_{max} denotes the

reciprocal of the minimum edge weight in G_{PPI}.

Based on the above calculations, the association strength $AS(p)$ of the sub-network *sub-G_{PPI}* centred on p was defined as the maximum association strength between the mutation pair p and its neighbors as follows.

$$AS(p) = \begin{cases} \max_{k \in N_p^{PPI}} AS(p,k), if\ N_p^{PPI} \neq \emptyset \\ \frac{M(i)M(j)M_{min}}{(d_{ij} \cdot d_{max})^2}, if\ N_p^{PPI} = \emptyset \end{cases}$$

(2) Calculation of the differential level of the network *sub-G_{diff}*. Based on the network *sub-G_{diff}* centred on a mutation pair $p = p(i, j)$, the differential level $DL(p,k)$ between p and its neighbour k was calculated as follows.

$$DL(p,k) = \begin{cases} \frac{max[W_{diff}(v_i), W_{diff}(v_j)] \cdot W_{diff}(v_k)}{(q_{pk})^2}, if\ N_p^{diff} \neq \emptyset \\ \frac{max[W_{diff}(v_i), W_{diff}(v_j)] \cdot W_{diff}(v_{min})}{(q_{max})^2}, if\ N_p^{diff} = \emptyset \end{cases}$$

where N_p^{diff} is the set of neighbors of p in G_{diff}, $q_{pk} = 1 - E(k,p)$, $q_{max} = \max_{e_{ij} \in G_{diff}} [1 - W_{diff}(e_{ij})]$, and $W_{diff}(v_{min})$ represents the minimum node weight in G_{diff}.

Then, the differential level $DEL(p)$ of the sub-network *sub-G_{diff}* centred on p was defined as the maximum differential level between the mutation pair p and its neighbors as follows.

$$DL(p) = \begin{cases} \max_{k \in N_p^{diff}} DL(p,k), if\ N_p^{diff} \neq \emptyset \\ \frac{max[W_{diff}(v_i), W_{diff}(v_j)] \cdot W_{diff}(v_{min})}{(q_{max})^2}, if\ N_p^{diff} = \emptyset \end{cases}$$

Based on the association strength $AS(p)$ and $DEL(p)$ of the two sub-networks *sub-G_{PPI}* and *sub-G_{diff}* centred on p, the impact score $DCIS(p)$ of the mutation pair p in driving cancers was calculated by multiplying $AS(p)$ and $DEL(p)$.

$$DCIS(p) = AS(p) \cdot DL(p)$$

Prioritization of individual mutations by partitioning mutation pairs

To effectively prioritize individual mutations, the impact score of a mutation pair $p = p(i, j)$ needs to be partitioned into two impact scores $DCIS(i,p)$ and $DCIS(j,p)$ corresponding to the two individual mutations. The partitioning ratio was determined by the different influences

of the two mutations on the PPI network. In this study, the influence $s(i)$ of gene *i* on the PPI network is defined as follows.

$$s(i) = \sum_{j \in N_i^{PPI}} W_{PPI}(e_{ij})$$

where N_i^{PPI} represents the neighbors of gene *i* on the PPI network. Therefore, the impact score $DCIS(i,p)$ of gene *i* based on the mutation pair *p* = *p*(*i*, *j*) was calculated as follows.

$$DCIS(i,p) = \frac{s(i)}{s(i) + s(j)} DCIS(p)$$

In practice, a gene *i* may be included in multiple mutation pairs, and the maximum one was regarded as the final impact score of the mutation.

$$DCIS(i) = \max_{j \in N_i^P} \frac{s(i)}{s(i) + s(j)} DCIS(p)$$

where N_i^P denotes the set of mutation pairs that include gene i. Based on the impact scores, individual mutations were prioritized accordingly.

Availability of supporting source code and requirements

Project name: DriverMP

Project home page: https://github.com/LiuYangyangSDU/DriverMP

Operating system(s): Linux/Unix

Programming language: C++

Other requirements: g++ version 7.5.0

License: GNU GPL v3.0

RRID: SCR_023796

Data availability

The somatic non-silent mutation data, gene expression data, and PPI networks supporting the results of this article are available at https://sourceforge.net/projects/transcriptomeassembly/files/DriverMP/.

Additional Files

Supplementary Materials. This file contains Supplementary Results, Supplementary Figures, and Supplementary Tables. The Supplementary Results section displays the five-level assessment of the other eight cancers including PRAD, LUSC, HNSC, COADREAD, BLCA,

UCEC, KIRC and KIRP. In the Supplementary Figures section, Figures S1-S4 show the comparison between DriverMP and other methods based on the HumanNet PPI network. Figures S5-S12 show the results of five-level assessment of the other eight cancers including PRAD, LUSC, HNSC, COADREAD, BLCA, UCEC, KIRC and KIRP. In the Supplementary Tables section, Table S1 shows the summary of the ten somatic non-silent mutation and tumor/normal expression datasets of each cancer type from TCGA. Tables S2 lists the genes for the four BC-related diseases against GAD. Tables S3 lists the genes for the four LUAD-related diseases against GAD. Tables S4-S24 show the detailed results of five levels of the other eight cancers including PRAD, LUSC, HNSC, COADREAD, BLCA, UCEC, KIRC and KIRP.

Competing Interests

The authors declare that they have no competing interests.

Authors' Contributions

Conceived and designed the experiments: JL. Performed the experiments: YL JH NX JL. Analyzed the data: YL TK. Contributed reagents/materials/analysis tools: YL JH QM. Wrote the paper: JL YL QM. Designed the software used in analysis: YL JH. Oversaw the project: JL.

Funding

This work was supported by the National Key R&D Program of China with code 2020YFA0712400, and the National Natural Science Foundation of China with code 62272268. The funders had no role in study design, data collection and analysis, decision to publish, or preparation of the manuscript.

References

1. What Is Cancer? https://www.cancer.gov/about-cancer/understanding/what-is-cancer.

2. Stratton MR, Campbell PJ and Futreal PA. The cancer genome. Nature. 2009;458 7239:719-24. doi:10.1038/nature07943.

3. Tomczak K, Czerwinska P and Wiznerowicz M. The Cancer Genome Atlas (TCGA): an

immeasurable source of knowledge. Contemp Oncol (Pozn). 2015;19 1A:A68-77. doi:10.5114/wo.2014.47136.

4. International Cancer Genome C, Hudson TJ, Anderson W, Artez A, Barker AD, Bell C, et al. International network of cancer genome projects. Nature. 2010;464 7291:993-8. doi:10.1038/nature08987.

5. Therapeutically Applicable Research to Generate Effective Treatments (TARGET). U.S. 2007. https://ocg.cancer.gov/programs/target.

6. Garraway LA and Lander ES. Lessons from the cancer genome. Cell. 2013;153 1:17-37. doi:10.1016/j.cell.2013.03.002.

7. Vogelstein B, Papadopoulos N, Velculescu VE, Zhou S, Diaz LA, Jr. and Kinzler KW. Cancer genome landscapes. Science. 2013;339 6127:1546-58. doi:10.1126/science.1235122.

8. Martincorena I and Campbell PJ. Somatic mutation in cancer and normal cells. Science. 2015;349 6255:1483-9. doi:10.1126/science.aab4082.

9. Lawrence MS, Stojanov P, Mermel CH, Robinson JT, Garraway LA, Golub TR, et al. Discovery and saturation analysis of cancer genes across 21 tumour types. Nature. 2014;505 7484:495-501. doi:10.1038/nature12912.

10. Lawrence MS, Stojanov P, Polak P, Kryukov GV, Cibulskis K, Sivachenko A, et al. Mutational heterogeneity in cancer and the search for new cancer-associated genes. Nature. 2013;499 7457:214-8. doi:10.1038/nature12213.

11. Jiang L, Zheng J, Kwan JSH, Dai S, Li C, Li MJ, et al. WITER: a powerful method for estimation of cancer-driver genes using a weighted iterative regression modelling

background mutation counts. Nucleic Acids Res. 2019;47 16:e96. doi:10.1093/nar/gkz566.

12. Reva B, Antipin Y and Sander C. Predicting the functional impact of protein mutations: application to cancer genomics. Nucleic Acids Res. 2011;39 17:e118. doi:10.1093/nar/gkr407.

13. Carter H, Chen S, Isik L, Tyekucheva S, Velculescu VE, Kinzler KW, et al. Cancer-specific high-throughput annotation of somatic mutations: computational prediction of driver missense mutations. Cancer Res. 2009;69 16:6660-7. doi:10.1158/0008-5472.CAN-09-1133.

14. Gonzalez-Perez A, Deu-Pons J and Lopez-Bigas N. Improving the prediction of the functional impact of cancer mutations by baseline tolerance transformation. Genome Med. 2012;4 11:89. doi:10.1186/gm390.

15. Shihab HA, Gough J, Cooper DN, Stenson PD, Barker GL, Edwards KJ, et al. Predicting the functional, molecular, and phenotypic consequences of amino acid substitutions using hidden Markov models. Hum Mutat. 2013;34 1:57-65. doi:10.1002/humu.22225.

16. Cho A, Shim JE, Kim E, Supek F, Lehner B and Lee I. MUFFINN: cancer gene discovery via network analysis of somatic mutation data. Genome Biol. 2016;17 1:129. doi:10.1186/s13059-016-0989-x.

17. Leiserson MD, Vandin F, Wu HT, Dobson JR, Eldridge JV, Thomas JL, et al. Pan-cancer network analysis identifies combinations of rare somatic mutations across pathways and protein complexes. Nat Genet. 2015;47 2:106-14. doi:10.1038/ng.3168.

18. Wood LD, Parsons DW, Jones S, Lin J, Sjoblom T, Leary RJ, et al. The genomic landscapes of human breast and colorectal cancers. Science. 2007;318 5853:1108-13. doi:10.1126/science.1145720.

19. Jia P and Zhao Z. VarWalker: personalized mutation network analysis of putative cancer genes from next-generation sequencing data. PLoS Comput Biol. 2014;10 2:e1003460. doi:10.1371/journal.pcbi.1003460.

20. Hou Y, Gao B, Li G and Su Z. MaxMIF: A New Method for Identifying Cancer Driver Genes through Effective Data Integration. Adv Sci (Weinh). 2018;5 9:1800640. doi:10.1002/advs.201800640.

21. Bashashati A, Haffari G, Ding J, Ha G, Lui K, Rosner J, et al. DriverNet: uncovering the impact of somatic driver mutations on transcriptional networks in cancer. Genome Biol. 2012;13 12:R124. doi:10.1186/gb-2012-13-12-r124.

22. Zhang W and Wang SL. A Novel Method for Identifying the Potential Cancer Driver Genes Based on Molecular Data Integration. Biochem Genet. 2020;58 1:16-39. doi:10.1007/s10528-019-09924-2.

23. Hou JP and Ma J. DawnRank: discovering personalized driver genes in cancer. Genome Med. 2014;6 7:56. doi:10.1186/s13073-014-0056-8.

24. Lee I, Blom UM, Wang PI, Shim JE and Marcotte EM. Prioritizing candidate disease genes by network-based boosting of genome-wide association data. Genome Res. 2011;21 7:1109-21. doi:10.1101/gr.118992.110.

25. Szklarczyk D, Franceschini A, Wyder S, Forslund K, Heller D, Huerta-Cepas J, et al. STRING v10: protein-protein interaction networks, integrated over the tree of life.

Nucleic Acids Res. 2015;43 Database issue:D447-52. doi:10.1093/nar/gku1003.

26. Huang da W, Sherman BT and Lempicki RA. Systematic and integrative analysis of large gene lists using DAVID bioinformatics resources. Nat Protoc. 2009;4 1:44-57. doi:10.1038/nprot.2008.211.

27. Becker KG, Barnes KC, Bright TJ and Wang SA. The genetic association database. Nat Genet. 2004;36 5:431-2. doi:10.1038/ng0504-431.

28. Kanehisa M, Furumichi M, Tanabe M, Sato Y and Morishima K. KEGG: new perspectives on genomes, pathways, diseases and drugs. Nucleic Acids Res. 2017;45 D1:D353-D61. doi:10.1093/nar/gkw1092.

29. Fabregat A, Sidiropoulos K, Viteri G, Forner O, Marin-Garcia P, Arnau V, et al. Reactome pathway analysis: a high-performance in-memory approach. BMC Bioinformatics. 2017;18 1:142. doi:10.1186/s12859-017-1559-2.

30. His M, Zelek L, Deschasaux M, Pouchieu C, Kesse-Guyot E, Hercberg S, et al. Prospective associations between serum biomarkers of lipid metabolism and overall, breast and prostate cancer risk. Eur J Epidemiol. 2014;29 2:119-32. doi:10.1007/s10654-014-9884-5.

31. Sunters A, Madureira PA, Pomeranz KM, Aubert M, Brosens JJ, Cook SJ, et al. Paclitaxel-induced nuclear translocation of FOXO3a in breast cancer cells is mediated by c-Jun NH2-terminal kinase and Akt. Cancer Res. 2006;66 1:212-20. doi:10.1158/0008-5472.CAN-05-1997.

32. Eddy SF, Kane SE and Sonenshein GE. Trastuzumab-resistant HER2-driven breast cancer cells are sensitive to epigallocatechin-3 gallate. Cancer Res. 2007;67 19:9018-

23. doi:10.1158/0008-5472.CAN-07-1691.

33. Majidinia M and Yousefi B. DNA repair and damage pathways in breast cancer development and therapy. DNA Repair (Amst). 2017;54:22-9. doi:10.1016/j.dnarep.2017.03.009.

34. Liang Q, Li L, Zhang J, Lei Y, Wang L, Liu DX, et al. CDK5 is essential for TGF-beta1-induced epithelial-mesenchymal transition and breast cancer progression. Sci Rep. 2013;3:2932. doi:10.1038/srep02932.

35. Zhang Y, Yang WK, Wen GM, Tang H, Wu CA, Wu YX, et al. High expression of PRKDC promotes breast cancer cell growth via p38 MAPK signaling and is associated with poor survival. Mol Genet Genomic Med. 2019;7 11:e908. doi:10.1002/mgg3.908.

36. Farabaugh SM, Boone DN and Lee AV. Role of IGF1R in Breast Cancer Subtypes, Stemness, and Lineage Differentiation. Front Endocrinol (Lausanne). 2015;6:59. doi:10.3389/fendo.2015.00059.

37. Thorslund T, McIlwraith MJ, Compton SA, Lekomtsev S, Petronczki M, Griffith JD, et al. The breast cancer tumor suppressor BRCA2 promotes the specific targeting of RAD51 to single-stranded DNA. Nat Struct Mol Biol. 2010;17 10:1263-5. doi:10.1038/nsmb.1905.

38. De Gregoriis G, Ramos JA, Fernandes PV, Vignal GM, Brianese RC, Carraro DM, et al. DNA repair genes PAXIP1 and TP53BP1 expression is associated with breast cancer prognosis. Cancer Biol Ther. 2017;18 6:439-49. doi:10.1080/15384047.2017.1323590.

39. Moslehi R, Tsao HS, Zeinomar N, Stagnar C, Fitzpatrick S and Dzutsev A. Integrative

genomic analysis implicates ERCC6 and its interaction with ERCC8 in susceptibility to breast cancer. Sci Rep. 2020;10 1:21276. doi:10.1038/s41598-020-77037-7.

40. Ding K, Li W, Zou Z, Zou X and Wang C. CCNB1 is a prognostic biomarker for ER+ breast cancer. Med Hypotheses. 2014;83 3:359-64. doi:10.1016/j.mehy.2014.06.013.

41. Pichiorri F, Palmieri D, De Luca L, Consiglio J, You J, Rocci A, et al. In vivo NCL targeting affects breast cancer aggressiveness through miRNA regulation. J Exp Med. 2013;210 5:951-68. doi:10.1084/jem.20120950.

42. Xing Z, Wang X, Liu J, Zhang M, Feng K and Wang X. Expression and prognostic value of CDK1, CCNA2, and CCNB1 gene clusters in human breast cancer. J Int Med Res. 2021;49 4:300060520980647. doi:10.1177/0300060520980647.

43. Colavito SA. AXL as a Target in Breast Cancer Therapy. J Oncol. 2020;2020:5291952. doi:10.1155/2020/5291952.

44. Massah S, Foo J, Li N, Truong S, Nouri M, Xie L, et al. Gli activation by the estrogen receptor in breast cancer cells: Regulation of cancer cell growth by Gli3. Mol Cell Endocrinol. 2021;522:111136. doi:10.1016/j.mce.2020.111136.

45. Lone BA, Ahmad F, Karna SKL and Pokharel YR. SUPT5H Post-Transcriptional Silencing Modulates PIN1 Expression, Inhibits Tumorigenicity, and Induces Apoptosis of Human Breast Cancer Cells. Cell Physiol Biochem. 2020;54 5:928-46. doi:10.33594/000000279.

46. Wu D, Han B, Guo L and Fan Z. Molecular mechanisms associated with breast cancer based on integrated gene expression profiling by bioinformatics analysis. J Obstet Gynaecol. 2016;36 5:615-21. doi:10.3109/01443615.2015.1127902.

47. Liu H and Ye H. Screening of the prognostic targets for breast cancer based co-expression modules analysis. Mol Med Rep. 2017;16 4:4038-44. doi:10.3892/mmr.2017.7063.

48. Cancer Genome Atlas N. Comprehensive molecular portraits of human breast tumours. Nature. 2012;490 7418:61-70. doi:10.1038/nature11412.

49. Kruiswijk F, Labuschagne CF and Vousden KH. p53 in survival, death and metabolic health: a lifeguard with a licence to kill. Nat Rev Mol Cell Biol. 2015;16 7:393-405. doi:10.1038/nrm4007.

50. Templeton AJ, Diez-Gonzalez L, Ace O, Vera-Badillo F, Seruga B, Jordan J, et al. Prognostic relevance of receptor tyrosine kinase expression in breast cancer: a meta-analysis. Cancer Treat Rev. 2014;40 9:1048-55. doi:10.1016/j.ctrv.2014.08.003.

51. Qian BZ, Zhang H, Li J, He T, Yeo EJ, Soong DY, et al. FLT1 signaling in metastasis-associated macrophages activates an inflammatory signature that promotes breast cancer metastasis. J Exp Med. 2015;212 9:1433-48. doi:10.1084/jem.20141555.

52. Zhao D, Pan C, Sun J, Gilbert C, Drews-Elger K, Azzam DJ, et al. VEGF drives cancer-initiating stem cells through VEGFR-2/Stat3 signaling to upregulate Myc and Sox2. Oncogene. 2015;34 24:3107-19. doi:10.1038/onc.2014.257.

53. Park J, Kim S, Joh J, Remick SC, Miller DM, Yan J, et al. MLLT11/AF1q boosts oncogenic STAT3 activity through Src-PDGFR tyrosine kinase signaling. Oncotarget. 2016;7 28:43960-73. doi:10.18632/oncotarget.9759.

54. Ibrahim SA, Gadalla R, El-Ghonaimy EA, Samir O, Mohamed HT, Hassan H, et al. Syndecan-1 is a novel molecular marker for triple negative inflammatory breast cancer

and modulates the cancer stem cell phenotype via the IL-6/STAT3, Notch and EGFR signaling pathways. Mol Cancer. 2017;16 1:57. doi:10.1186/s12943-017-0621-z.

55. Wise R and Zolkiewska A. Metalloprotease-dependent activation of EGFR modulates CD44(+)/CD24(-) populations in triple negative breast cancer cells through the MEK/ERK pathway. Breast Cancer Res Treat. 2017;166 2:421-33. doi:10.1007/s10549-017-4440-0.

56. Wolf I, Sadetzki S, Catane R, Karasik A and Kaufman B. Diabetes mellitus and breast cancer. Lancet Oncol. 2005;6 2:103-11. doi:10.1016/S1470-2045(05)01736-5.

57. Khanh VC, Fukushige M, Moriguchi K, Yamashita T, Osaka M, Hiramatsu Y, et al. Type 2 Diabetes Mellitus Induced Paracrine Effects on Breast Cancer Metastasis Through Extracellular Vesicles Derived from Human Mesenchymal Stem Cells. Stem Cells Dev. 2020;29 21:1382-94. doi:10.1089/scd.2020.0126.

58. Thu KL, Soria-Bretones I, Mak TW and Cescon DW. Targeting the cell cycle in breast cancer: towards the next phase. Cell Cycle. 2018;17 15:1871-85. doi:10.1080/15384101.2018.1502567.

59. Ponnusamy L, Natarajan SR, Thangaraj K and Manoharan R. Therapeutic aspects of AMPK in breast cancer: Progress, challenges, and future directions. Biochim Biophys Acta Rev Cancer. 2020;1874 1:188379. doi:10.1016/j.bbcan.2020.188379.

60. Mazouni C, Fina F, Romain S, Ouafik L, Bonnier P, Brandone JM, et al. Epstein-Barr virus as a marker of biological aggressiveness in breast cancer. Br J Cancer. 2011;104 2:332-7. doi:10.1038/sj.bjc.6606048.

61. Miricescu D, Totan A, Stanescu S, II, Badoiu SC, Stefani C and Greabu M.

PI3K/AKT/mTOR Signaling Pathway in Breast Cancer: From Molecular Landscape to Clinical Aspects. Int J Mol Sci. 2020;22 1 doi:10.3390/ijms22010173.

62. Musgrove EA and Sutherland RL. Biological determinants of endocrine resistance in breast cancer. Nat Rev Cancer. 2009;9 9:631-43. doi:10.1038/nrc2713.

63. Amarante MK and Watanabe MA. The possible involvement of virus in breast cancer. J Cancer Res Clin Oncol. 2009;135 3:329-37. doi:10.1007/s00432-008-0511-2.

64. Pichard C, Plu-Bureau G, Neves ECM and Gompel A. Insulin resistance, obesity and breast cancer risk. Maturitas. 2008;60 1:19-30. doi:10.1016/j.maturitas.2008.03.002.

65. Luo M and Guan JL. Focal adhesion kinase: a prominent determinant in breast cancer initiation, progression and metastasis. Cancer Lett. 2010;289 2:127-39. doi:10.1016/j.canlet.2009.07.005.

66. Theocharis AD, Skandalis SS, Neill T, Multhaupt HA, Hubo M, Frey H, et al. Insights into the key roles of proteoglycans in breast cancer biology and translational medicine. Biochim Biophys Acta. 2015;1855 2:276-300. doi:10.1016/j.bbcan.2015.03.006.

67. Belfiore A and Frasca F. IGF and insulin receptor signaling in breast cancer. J Mammary Gland Biol Neoplasia. 2008;13 4:381-406. doi:10.1007/s10911-008-9099-z.

68. Hardy KM, Booth BW, Hendrix MJ, Salomon DS and Strizzi L. ErbB/EGF signaling and EMT in mammary development and breast cancer. J Mammary Gland Biol Neoplasia. 2010;15 2:191-9. doi:10.1007/s10911-010-9172-2.

69. Rabellino A, Andreani C and Scaglioni PP. The Role of PIAS SUMO E3-Ligases in Cancer. Cancer Res. 2017;77 7:1542-7. doi:10.1158/0008-5472.CAN-16-2958.

70. Darnell JE, Jr. Transcription factors as targets for cancer therapy. Nat Rev Cancer.

2002;2 10:740-9. doi:10.1038/nrc906.

71. Ralhan R, Kaur J, Kreienberg R and Wiesmuller L. Links between DNA double strand break repair and breast cancer: accumulating evidence from both familial and nonfamilial cases. Cancer Lett. 2007;248 1:1-17. doi:10.1016/j.canlet.2006.06.004.

72. Felty Q, Singh KP and Roy D. Estrogen-induced G1/S transition of G0-arrested estrogen-dependent breast cancer cells is regulated by mitochondrial oxidant signaling. Oncogene. 2005;24 31:4883-93. doi:10.1038/sj.onc.1208667.

73. Zhang Q, Lu S, Li T, Yu L, Zhang Y, Zeng H, et al. ACE2 inhibits breast cancer angiogenesis via suppressing the VEGFa/VEGFR2/ERK pathway. J Exp Clin Cancer Res. 2019;38 1:173. doi:10.1186/s13046-019-1156-5.

74. Wang L, Simons DL, Lu X, Tu TY, Avalos C, Chang AY, et al. Breast cancer induces systemic immune changes on cytokine signaling in peripheral blood monocytes and lymphocytes. EBioMedicine. 2020;52:102631. doi:10.1016/j.ebiom.2020.102631.

75. Harburg GC and Hinck L. Navigating breast cancer: axon guidance molecules as breast cancer tumor suppressors and oncogenes. J Mammary Gland Biol Neoplasia. 2011;16 3:257-70. doi:10.1007/s10911-011-9225-1.

76. Seiler A, Chen MA, Brown RL and Fagundes CP. Obesity, Dietary Factors, Nutrition, and Breast Cancer Risk. Curr Breast Cancer Rep. 2018;10 1:14-27. doi:10.1007/s12609-018-0264-0.

77. Abe R, Kumagai N, Kimura M, Hirosaki A and Nakamura T. Biological characteristics of breast cancer in obesity. Tohoku J Exp Med. 1976;120 4:351-9. doi:10.1620/tjem.120.351.

78. Ewertz M, Jensen MB, Gunnarsdottir KA, Hojris I, Jakobsen EH, Nielsen D, et al. Effect of obesity on prognosis after early-stage breast cancer. J Clin Oncol. 2011;29 1:25-31. doi:10.1200/JCO.2010.29.7614.

79. Osman MA and Hennessy BT. Obesity Correlation With Metastases Development and Response to First-Line Metastatic Chemotherapy in Breast Cancer. Clin Med Insights Oncol. 2015;9:105-12. doi:10.4137/CMO.S32812.

80. O'Flanagan CH, Rossi EL, McDonell SB, Chen X, Tsai YH, Parker JS, et al. Metabolic reprogramming underlies metastatic potential in an obesity-responsive murine model of metastatic triple negative breast cancer. NPJ Breast Cancer. 2017;3:26. doi:10.1038/s41523-017-0027-5.

81. Bousquenaud M, Fico F, Solinas G, Ruegg C and Santamaria-Martinez A. Obesity promotes the expansion of metastasis-initiating cells in breast cancer. Breast Cancer Res. 2018;20 1:104. doi:10.1186/s13058-018-1029-4.

82. Evangelista GCM, Salvador PA, Soares SMA, Barros LRC, Xavier F, Abdo LM, et al. 4T1 Mammary Carcinoma Colonization of Metastatic Niches Is Accelerated by Obesity. Front Oncol. 2019;9:685. doi:10.3389/fonc.2019.00685.

83. Furberg AS, Veierod MB, Wilsgaard T, Bernstein L and Thune I. Serum high-density lipoprotein cholesterol, metabolic profile, and breast cancer risk. J Natl Cancer Inst. 2004;96 15:1152-60. doi:10.1093/jnci/djh216.

84. Furberg AS, Jasienska G, Bjurstam N, Torjesen PA, Emaus A, Lipson SF, et al. Metabolic and hormonal profiles: HDL cholesterol as a plausible biomarker of breast cancer risk. The Norwegian EBBA Study. Cancer Epidemiol Biomarkers Prev. 2005;14

1:33-40.

85. Kucharska-Newton AM, Rosamond WD, Mink PJ, Alberg AJ, Shahar E and Folsom AR. HDL-cholesterol and incidence of breast cancer in the ARIC cohort study. Ann Epidemiol. 2008;18 9:671-7. doi:10.1016/j.annepidem.2008.06.006.

86. Kim Y, Park SK, Han W, Kim DH, Hong YC, Ha EH, et al. Serum high-density lipoprotein cholesterol and breast cancer risk by menopausal status, body mass index, and hormonal receptor in Korea. Cancer Epidemiol Biomarkers Prev. 2009;18 2:508-15. doi:10.1158/1055-9965.EPI-08-0133.

87. Touvier M, Fassier P, His M, Norat T, Chan DS, Blacher J, et al. Cholesterol and breast cancer risk: a systematic review and meta-analysis of prospective studies. Br J Nutr. 2015;114 3:347-57. doi:10.1017/S000711451500183X.

88. Li X, Tang H, Wang J, Xie X, Liu P, Kong Y, et al. The effect of preoperative serum triglycerides and high-density lipoprotein-cholesterol levels on the prognosis of breast cancer. Breast. 2017;32:1-6. doi:10.1016/j.breast.2016.11.024.

89. Schnarr B, Strunz K, Ohsam J, Benner A, Wacker J and Mayer D. Down-regulation of insulin-like growth factor-I receptor and insulin receptor substrate-1 expression in advanced human breast cancer. Int J Cancer. 2000;89 6:506-13. doi:10.1002/1097-0215(20001120)89:6<506::aid-ijc7>3.0.co;2-f.

90. Maor S, Yosepovich A, Papa MZ, Yarden RI, Mayer D, Friedman E, et al. Elevated insulin-like growth factor-I receptor (IGF-IR) levels in primary breast tumors associated with BRCA1 mutations. Cancer Lett. 2007;257 2:236-43. doi:10.1016/j.canlet.2007.07.019.

91. Aleem E, Nehrbass D, Klimek F, Mayer D and Bannasch P. Upregulation of the insulin receptor and type I insulin-like growth factor receptor are early events in hepatocarcinogenesis. Toxicol Pathol. 2011;39 3:524-43. doi:10.1177/0192623310396905.

92. Maor SB, Abramovitch S, Erdos MR, Brody LC and Werner H. BRCA1 suppresses insulin-like growth factor-I receptor promoter activity: potential interaction between BRCA1 and Sp1. Mol Genet Metab. 2000;69 2:130-6. doi:10.1006/mgme.1999.2958.

93. Abramovitch S, Glaser T, Ouchi T and Werner H. BRCA1-Sp1 interactions in transcriptional regulation of the IGF-IR gene. FEBS Lett. 2003;541 1-3:149-54. doi:10.1016/s0014-5793(03)00315-6.

94. Werner H and Bruchim I. IGF-1 and BRCA1 signalling pathways in familial cancer. Lancet Oncol. 2012;13 12:e537-44. doi:10.1016/S1470-2045(12)70362-5.

95. Inamura K. Lung Cancer: Understanding Its Molecular Pathology and the 2015 WHO Classification. Front Oncol. 2017;7:193. doi:10.3389/fonc.2017.00193.

96. Saad MI, Rose-John S and Jenkins BJ. ADAM17: An Emerging Therapeutic Target for Lung Cancer. Cancers (Basel). 2019;11 9 doi:10.3390/cancers11091218.

97. Eapen MS, Hansbro PM, Larsson-Callerfelt AK, Jolly MK, Myers S, Sharma P, et al. Chronic Obstructive Pulmonary Disease and Lung Cancer: Underlying Pathophysiology and New Therapeutic Modalities. Drugs. 2018;78 16:1717-40. doi:10.1007/s40265-018-1001-8.

98. Criner GJ, Agusti A, Borghaei H, Friedberg J, Martinez FJ, Miyamoto C, et al. Chronic Obstructive Pulmonary Disease and Lung Cancer: A Review for Clinicians. Chronic

Obstr Pulm Dis. 2022;9 3:454-76. doi:10.15326/jcopdf.2022.0296.

99. Liu Z, Sun Q and Wang X. PLK1, A Potential Target for Cancer Therapy. Transl Oncol. 2017;10 1:22-32. doi:10.1016/j.tranon.2016.10.003.

100. Reda M, Ngamcherdtrakul W, Nelson MA, Siriwon N, Wang R, Zaidan HY, et al. Development of a nanoparticle-based immunotherapy targeting PD-L1 and PLK1 for lung cancer treatment. Nat Commun. 2022;13 1:4261. doi:10.1038/s41467-022-31926-9.

101. Hsu TI, Wang MC, Chen SY, Yeh YM, Su WC, Chang WC, et al. Sp1 expression regulates lung tumor progression. Oncogene. 2012;31 35:3973-88. doi:10.1038/onc.2011.568.

102. Yu X, Zhang Y, Ma X and Pertsemlidis A. miR-195 potentiates the efficacy of microtubule-targeting agents in non-small cell lung cancer. Cancer Lett. 2018;427:85-93. doi:10.1016/j.canlet.2018.04.007.

103. Wang L, Qu J, Liang Y, Zhao D, Rehman FU, Qin K, et al. Identification and validation of key genes with prognostic value in non-small-cell lung cancer via integrated bioinformatics analysis. Thorac Cancer. 2020;11 4:851-66. doi:10.1111/1759-7714.13298.

104. Xia Z, Ou-Yang W, Hu T and Du K. Prognostic significance of CDC25C in lung adenocarcinoma: An analysis of TCGA data. Cancer Genet. 2019;233-234:67-74. doi:10.1016/j.cancergen.2019.04.001.

105. Gorgisen G, Hapil FZ, Yilmaz O, Cetin Z, Pehlivanoglu S, Ozbudak IH, et al. Identification of novel mutations of Insulin Receptor Substrate 1 (IRS1) in tumor

samples of non-small cell lung cancer (NSCLC): Implications for aberrant insulin signaling in development of cancer. Genet Mol Biol. 2019;42 1:15-25. doi:10.1590/1678-4685-gmb-2017-0307.

106. Ke C, Zhu K, Sun Y, Ni Y, Zhang Z and Li X. SUMO1 promotes the proliferation and invasion of non-small cell lung cancer cells by regulating NF-kappaB. Thorac Cancer. 2019;10 1:33-40. doi:10.1111/1759-7714.12895.

107. Zhang YQ, Bianco A, Malkinson AM, Leoni VP, Frau G, De Rosa N, et al. BARD1: an independent predictor of survival in non-small cell lung cancer. Int J Cancer. 2012;131 1:83-94. doi:10.1002/ijc.26346.

108. Zhou X, Wang N, Zhang Y, Yu H and Wu Q. KAT2B is an immune infiltration-associated biomarker predicting prognosis and response to immunotherapy in non-small cell lung cancer. Invest New Drugs. 2022;40 1:43-57. doi:10.1007/s10637-021-01159-6.

109. Zhang C, Lu J, Zhang QW, Zhao W, Guo JH, Liu SL, et al. USP7 promotes cell proliferation through the stabilization of Ki-67 protein in non-small cell lung cancer cells. Int J Biochem Cell Biol. 2016;79:209-21. doi:10.1016/j.biocel.2016.08.025.

110. Kou F, Sun H, Wu L, Li B, Zhang B, Wang X, et al. TOP2A Promotes Lung Adenocarcinoma Cells' Malignant Progression and Predicts Poor Prognosis in Lung Adenocarcinoma. J Cancer. 2020;11 9:2496-508. doi:10.7150/jca.41415.

111. He W, Liu Q, Wang L, Chen W, Li N and Cao X. TLR4 signaling promotes immune escape of human lung cancer cells by inducing immunosuppressive cytokines and apoptosis resistance. Mol Immunol. 2007;44 11:2850-9. doi:10.1016/j.molimm.2007.01.022.

112. Fu HY, Li C, Yang W, Gai XD, Jia T, Lei YM, et al. FOXP3 and TLR4 protein expression are correlated in non-small cell lung cancer: implications for tumor progression and escape. Acta Histochem. 2013;115 2:151-7. doi:10.1016/j.acthis.2012.06.002.

113. Sutton P, Borgia JA, Bonomi P and Plate JM. Lyn, a Src family kinase, regulates activation of epidermal growth factor receptors in lung adenocarcinoma cells. Mol Cancer. 2013;12:76. doi:10.1186/1476-4598-12-76.

114. Wang J, Shi M, Zhang H, Zhou H, Huang Z, Zhou Y, et al. PRKCB is relevant to prognosis of lung adenocarcinoma through methylation and immune infiltration. Thorac Cancer. 2022;13 12:1837-49. doi:10.1111/1759-7714.14466.

115. Cao LL, Song X, Pei L, Liu L, Wang H and Jia M. Histone deacetylase HDAC1 expression correlates with the progression and prognosis of lung cancer: A meta-analysis. Medicine (Baltimore). 2017;96 31:e7663. doi:10.1097/MD.0000000000007663.

116. Bartkova J, Horejsi Z, Sehested M, Nesland JM, Rajpert-De Meyts E, Skakkebaek NE, et al. DNA damage response mediators MDC1 and 53BP1: constitutive activation and aberrant loss in breast and lung cancer, but not in testicular germ cell tumours. Oncogene. 2007;26 53:7414-22. doi:10.1038/sj.onc.1210553.

117. Ruff SE, Logan SK, Garabedian MJ and Huang TT. Roles for MDC1 in cancer development and treatment. DNA Repair (Amst). 2020;95:102948. doi:10.1016/j.dnarep.2020.102948.

118. Carelli S, Zadra G, Vaira V, Falleni M, Bottiglieri L, Nosotti M, et al. Up-regulation of focal adhesion kinase in non-small cell lung cancer. Lung Cancer. 2006;53 3:263-71.

doi:10.1016/j.lungcan.2006.06.001.

119. Sulzmaier FJ, Jean C and Schlaepfer DD. FAK in cancer: mechanistic findings and clinical applications. Nat Rev Cancer. 2014;14 9:598-610. doi:10.1038/nrc3792.

120. Iksen, Pothongsrisit S and Pongrakhananon V. Targeting the PI3K/AKT/mTOR Signaling Pathway in Lung Cancer: An Update Regarding Potential Drugs and Natural Products. Molecules. 2021;26 13 doi:10.3390/molecules26134100.

121. Reinmuth N, Meister M, Muley T, Steins M, Kreuter M, Herth FJ, et al. Molecular determinants of response to RTK-targeting agents in nonsmall cell lung cancer. Int J Cancer. 2006;119 4:727-34. doi:10.1002/ijc.21750.

122. Hong Z, Hong C, Ma B, Wang Q, Zhang X, Li L, et al. MicroRNA‑126‑3p inhibits the proliferation, migration, invasion, and angiogenesis of triple‑negative breast cancer cells by targeting RGS3. Oncol Rep. 2019;42 4:1569-79. doi:10.3892/or.2019.7251.

123. Malumbres M and Barbacid M. Cell cycle, CDKs and cancer: a changing paradigm. Nat Rev Cancer. 2009;9 3:153-66. doi:10.1038/nrc2602.

124. Limonta P, Montagnani Marelli M, Mai S, Motta M, Martini L and Moretti RM. GnRH receptors in cancer: from cell biology to novel targeted therapeutic strategies. Endocr Rev. 2012;33 5:784-811. doi:10.1210/er.2012-1014.

125. Pradhan R, Singhvi G, Dubey SK, Gupta G and Dua K. MAPK pathway: a potential target for the treatment of non-small-cell lung carcinoma. Future Med Chem. 2019;11 8:793-5. doi:10.4155/fmc-2018-0468.

126. Chhabra Y, Wong HY, Nikolajsen LF, Steinocher H, Papadopulos A, Tunny KA, et al. A growth hormone receptor SNP promotes lung cancer by impairment of SOCS2-

mediated degradation. Oncogene. 2018;37 4:489-501. doi:10.1038/onc.2017.352.

127. Park EJ, Jun HW, Na IH, Lee HK, Yun J, Kim HS, et al. CD48-expressing non-small-cell lung cancer cells are susceptible to natural killer cell-mediated cytotoxicity. Arch Pharm Res. 2022;45 1:1-10. doi:10.1007/s12272-021-01365-z.

128. Li J, Wang L, Chen X, Li L, Li Y, Ping Y, et al. CD39/CD73 upregulation on myeloid-derived suppressor cells via TGF-beta-mTOR-HIF-1 signaling in patients with non-small cell lung cancer. Oncoimmunology. 2017;6 6:e1320011. doi:10.1080/2162402X.2017.1320011.

129. Tsukita Y, Fujino N, Miyauchi E, Saito R, Fujishima F, Itakura K, et al. Axl kinase drives immune checkpoint and chemokine signalling pathways in lung adenocarcinomas. Mol Cancer. 2019;18 1:24. doi:10.1186/s12943-019-0953-y.

130. Jacobsen K, Bertran-Alamillo J, Molina MA, Teixido C, Karachaliou N, Pedersen MH, et al. Convergent Akt activation drives acquired EGFR inhibitor resistance in lung cancer. Nat Commun. 2017;8 1:410. doi:10.1038/s41467-017-00450-6.

131. Aviel-Ronen S, Blackhall FH, Shepherd FA and Tsao MS. K-ras mutations in non-small-cell lung carcinoma: a review. Clin Lung Cancer. 2006;8 1:30-8. doi:10.3816/CLC.2006.n.030.

132. Engelman JA and Cantley LC. The role of the ErbB family members in non-small cell lung cancers sensitive to epidermal growth factor receptor kinase inhibitors. Clin Cancer Res. 2006;12 14 Pt 2:4372s-6s. doi:10.1158/1078-0432.CCR-06-0795.

133. Stewart DJ. Wnt signaling pathway in non-small cell lung cancer. J Natl Cancer Inst. 2014;106 1:djt356. doi:10.1093/jnci/djt356.

134. Bushweller JH. Targeting transcription factors in cancer - from undruggable to reality. Nat Rev Cancer. 2019;19 11:611-24. doi:10.1038/s41568-019-0196-7.

135. Baietti MF and Sewduth RN. Novel Therapeutic Approaches Targeting Post-Translational Modifications in Lung Cancer. Pharmaceutics. 2023;15 1 doi:10.3390/pharmaceutics15010206.

136. Helleday T, Petermann E, Lundin C, Hodgson B and Sharma RA. DNA repair pathways as targets for cancer therapy. Nat Rev Cancer. 2008;8 3:193-204. doi:10.1038/nrc2342.

137. Xia Y, Wei K, Yang FM, Hu LQ, Pan CF, Pan XL, et al. miR-1260b, mediated by YY1, activates KIT signaling by targeting SOCS6 to regulate cell proliferation and apoptosis in NSCLC. Cell Death Dis. 2019;10 2:112. doi:10.1038/s41419-019-1390-y.

138. Alevizakos M, Kaltsas S and Syrigos KN. The VEGF pathway in lung cancer. Cancer Chemother Pharmacol. 2013;72 6:1169-81. doi:10.1007/s00280-013-2298-3.

139. Casper J, Zweig AS, Villarreal C, Tyner C, Speir ML, Rosenbloom KR, et al. The UCSC Genome Browser database: 2018 update. Nucleic Acids Res. 2018;46 D1:D762-D9. doi:10.1093/nar/gkx1020.

Figure Legends

Fig. 1 Workflow of DriverMP. a) Normalization of the mutation matrix and calculation of mutation scores. b) Preprocessing of the differential expression data and construction of the differential expression network G_{diff}. c) Calculation of impact scores (DCIS by AS and DL from *sub-*G_{PPI} and *sub-*G_{diff}, respectively) for paired mutations. d) Prioritization of individual mutations by partitioning mutation pairs.

Fig. 2 ROC curves of DriverMP and the other compared methods in **a)** BRCA, **b)** PRAD, **c)** LUAD, **d)** LUSC, **e)** KIRC, **f)** KIRP, **g)** HNSC, **h)** COADREAD **i)** UCEC and **j)** BLCA, using the STRINGv10 network.

Fig. 3 The curves of the numbers of identified known driver mutations of the top ranked 500 genes in **a)** BRCA, **b)** PRAD, **c)** LUAD, **d)** LUSC, **e)** KIRC, **f)** KIRP, **g)** HNSC, **h)** COADREAD, **i)** BLCA and **j)** BLCA, using the STRINGv10 PPI network.

Fig. 4 Performance of F1-scores of DriverMP and other compared methods in ten cancer types—**a)** BRCA, **b)** PRAD, **c)** LUAD, **d)** LUSC, **e)** KIRC, **f)** KIRP, **g)** HNSC, **h)** COADREAD, **i)** BLCA and **j)** BLCA—using the STRINGv10 network.

Fig. 5 Performance of DriverMP and other compared methods in terms of AUC and AUFC in ten cancer types. **a)** Boxplot of AUC scores across ten cancer types. **b)** Boxplot of AUFC scores across ten cancer types.

Fig. 6 Results of the stability tests. This figure shows the box plots of the effects of the number of samples on the performance of DriverMP in terms of mutation data (M) and differential expression (DE) data, measured by average AUC **(a)** and AUFC **(b)** of 10 times of random selections of different numbers of samples using STRINGv10 network.

Fig. 7 Five-level assessment of 51 driver candidates of breast cancer. **a)** Subnetwork of the genes in the analysis at the "cancer-type level" from the STRINGv10 network. The five genes in the blue box are enriched in the "FoxO signalling pathway" (KEGG), and the six genes in the red box are enriched in "DNA repair" (Reactome). **b)** Relationship of genes enriched in two biological pathways against KEGG from the STRINGv10 network. **c)** Relationship of genes enriched in two biological pathways against Reactome from the STRINGv10 network. **d)** Venn diagram of the top four levels of breast cancer.

Fig. 8 Five-level assessment of 60 driver candidates of lung adenocarcinoma. **a)** Subnetwork of the genes of the "cancer-type level" from the STRINGv10 network. The five genes in the blue box are enriched in the "cell cycle" pathway (KEGG), and the six genes in the red box are enriched in "DNA double-strand break repair" pathway (Reactome). **b)** Relationship of the genes enriched in the two biological pathways in the KEGG analysis of the STRINGv10 network. **c)** Relationship of the genes enriched in the two biological pathways in the Reactome analysis of the STRINGv10 network. **d)** Venn diagram of driver candidates for the top four levels of analysis for lung adenocarcinoma.

Fig. 9 The online web server of DriverMP. **a)** The interface of the online service. Users only need to submit formatted somatic mutation data and tumour and normal gene expression data,

choose one of the two PPI networks (HumanNet or STRINGv10), and press the “submit” button to start DriverMP. **b)** Results page. The top 50 gene candidates ranked by DriverMP are displayed by default, and users can manually choose to show the top 10, 20, 50, 100 and 200 genes. To view the full output, users can download the result file.

Fig. 10 Searchable database page. We applied DriverMP to ten different cancer types and obtained 85 driver gene candidates not included in CGC but strongly supported by related literature, based on which we built a database of reliable novel drivers.

Fig. 1

Click here to access/download;Figure;Figure1.tif

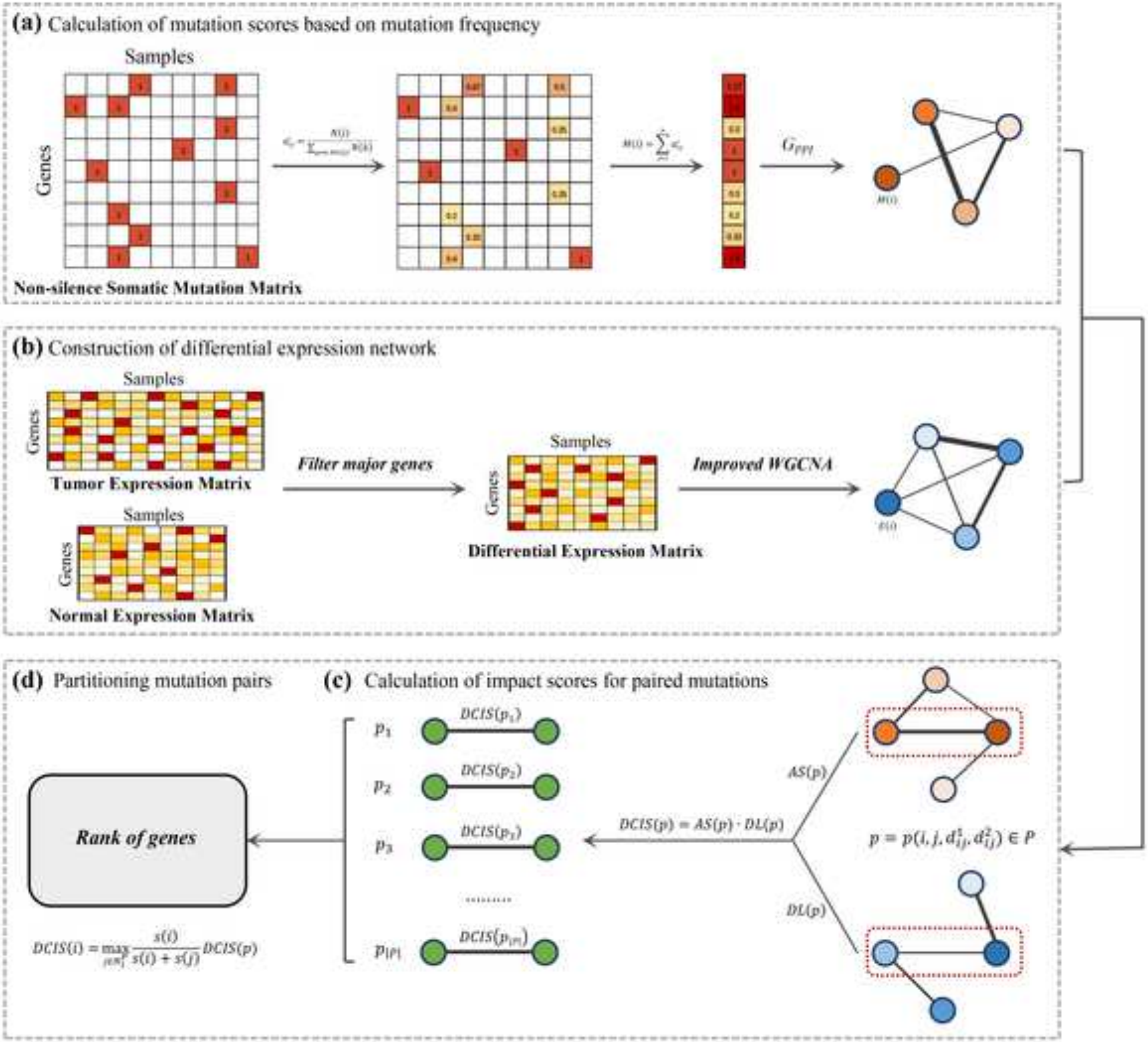

Fig. 2 Click here to access/download;Figure;Figure2.tif

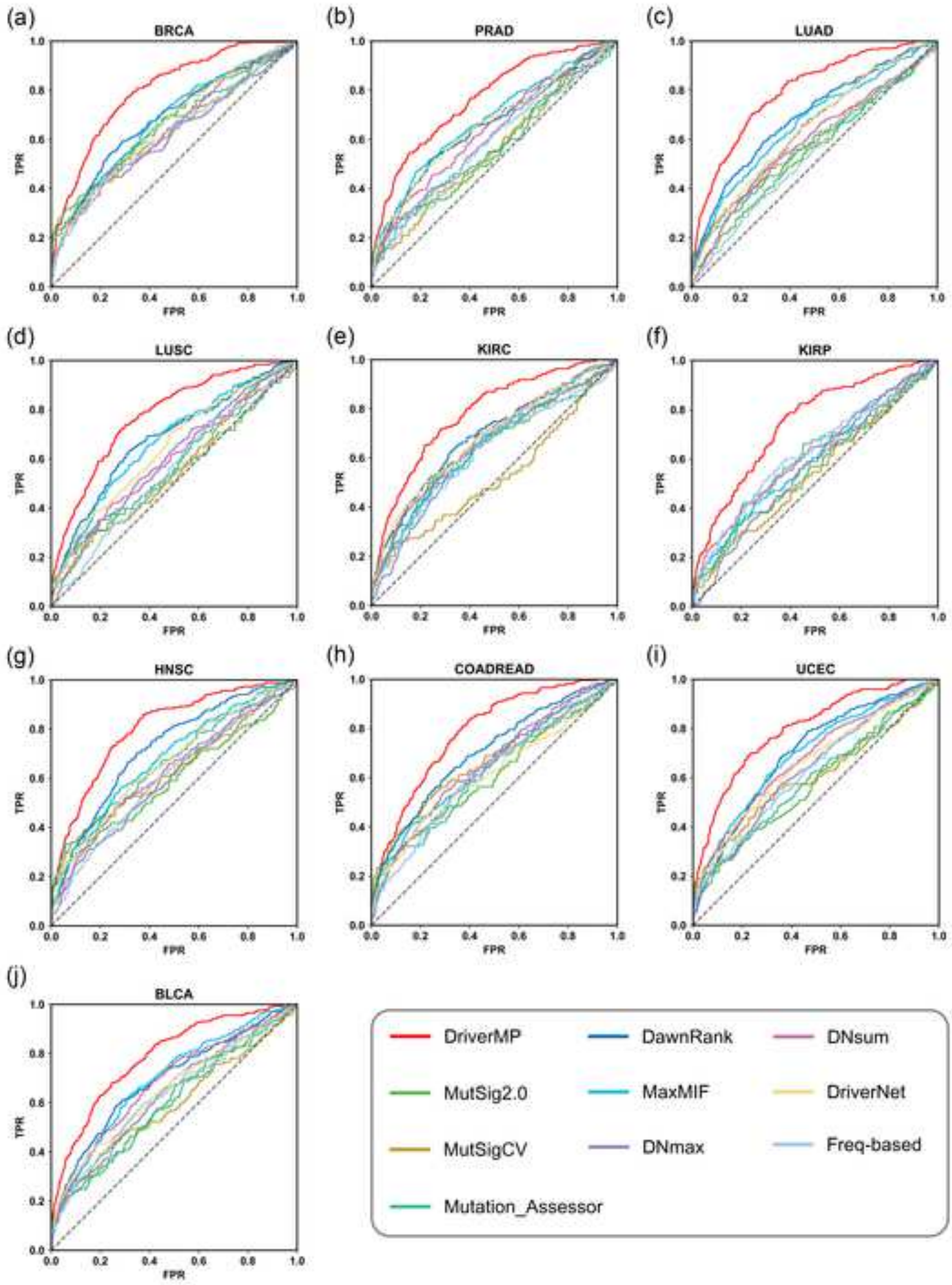

Fig. 3

Click here to access/download;Figure;Figure3.tif

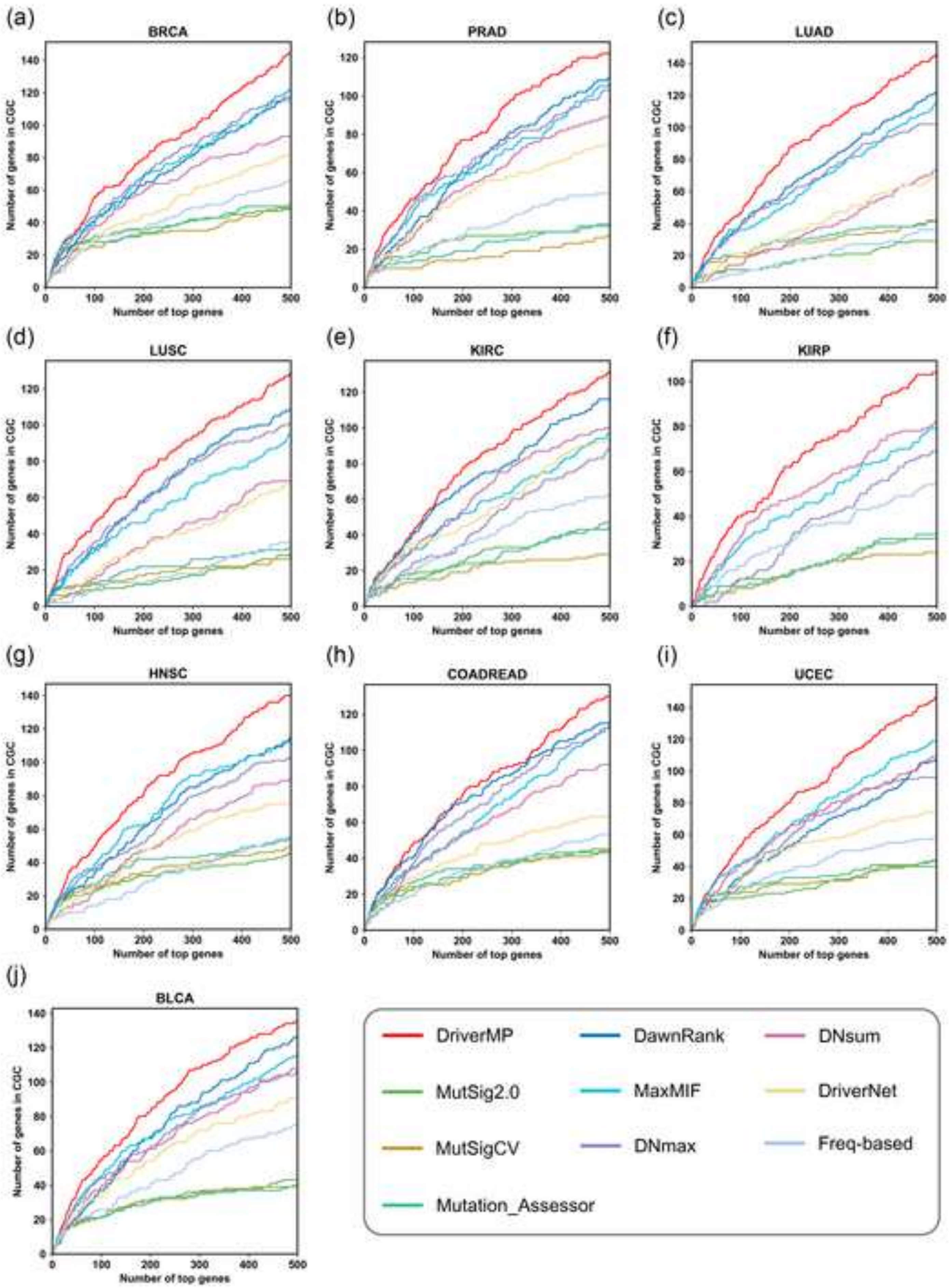

Fig. 4

Click here to access/download;Figure;Figure4.tif

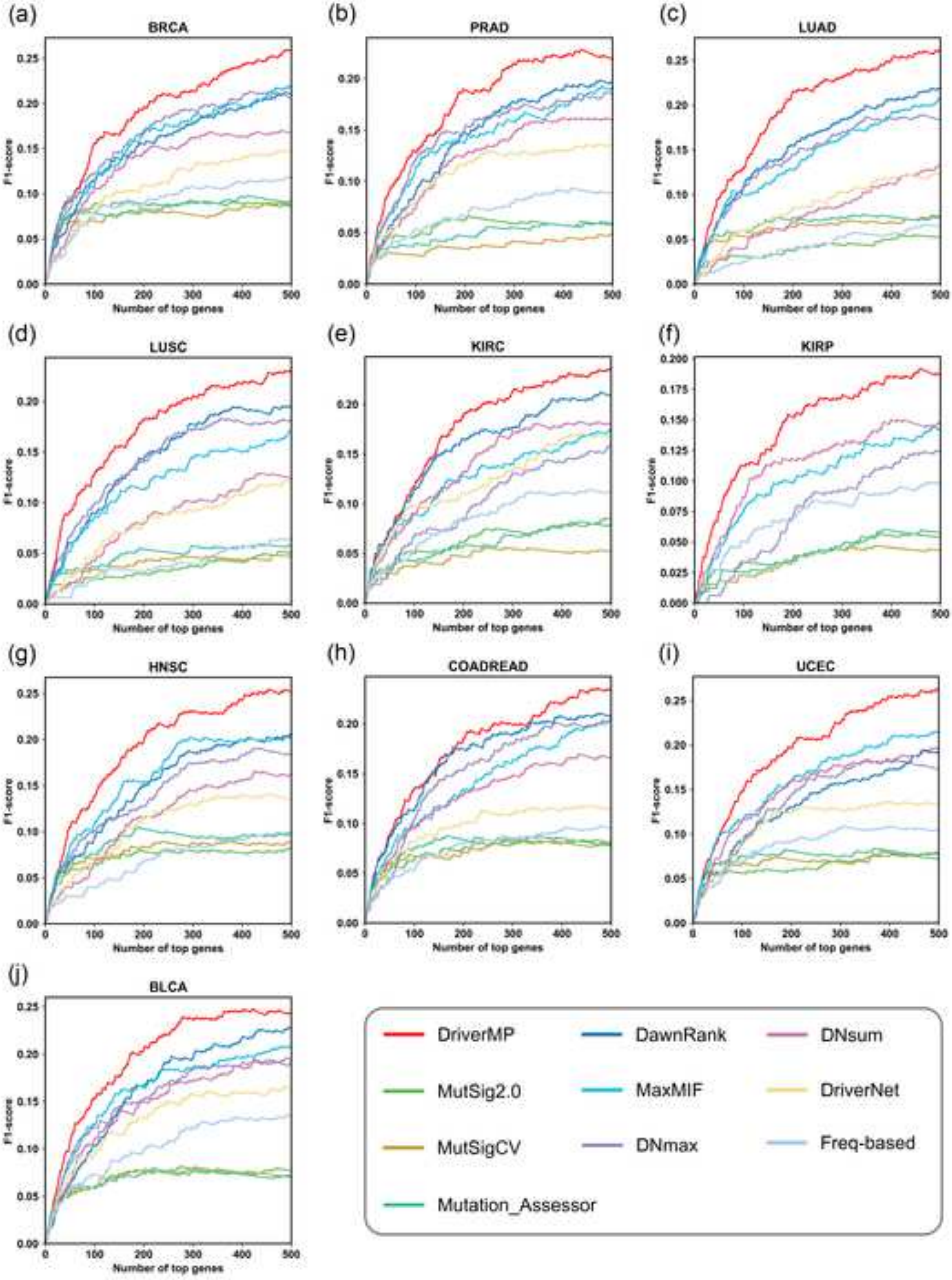

Fig. 5

Click here to access/download;Figure;Figure5.tif

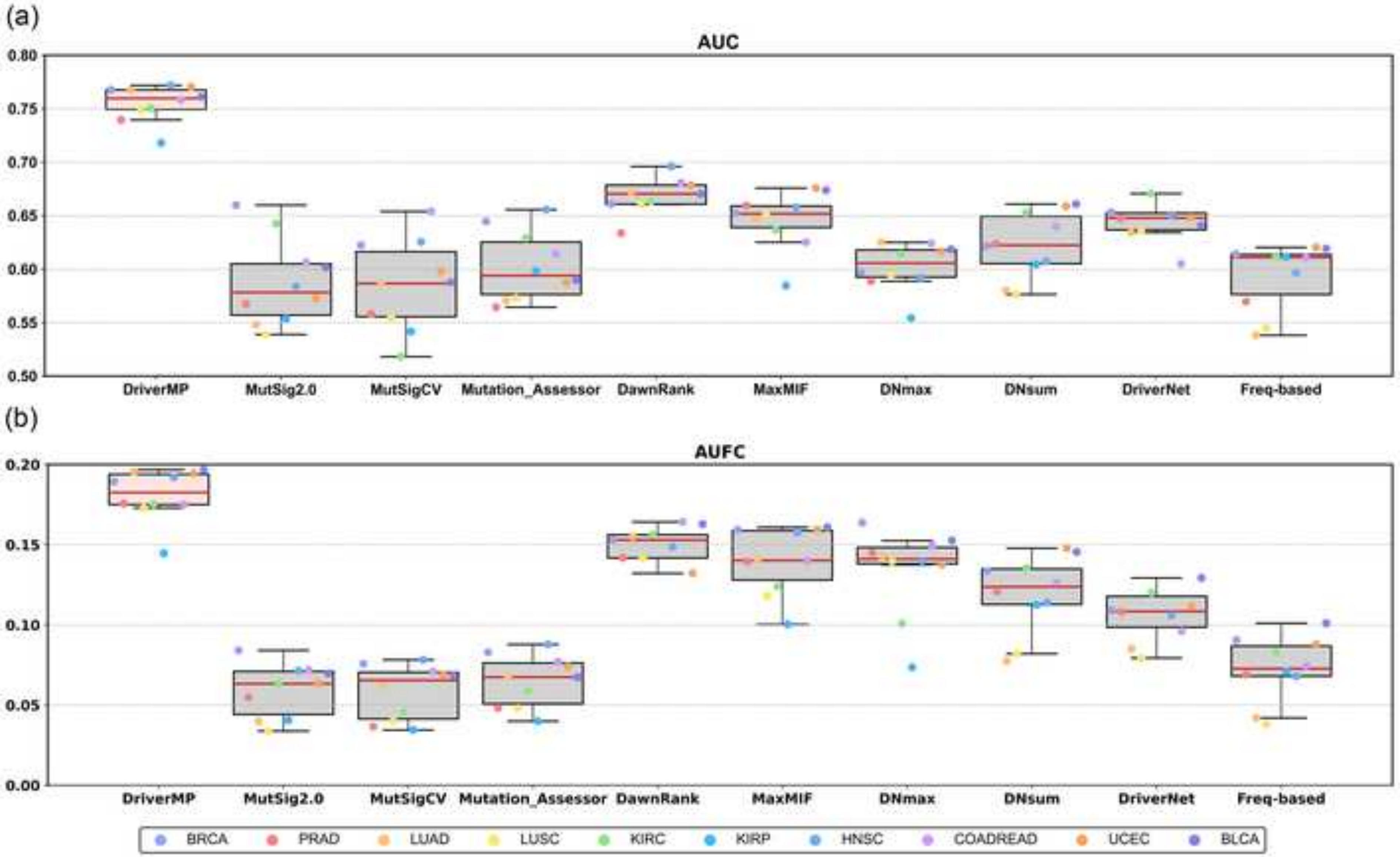

Fig. 6

Click here to access/download;Figure;Figure6.tif

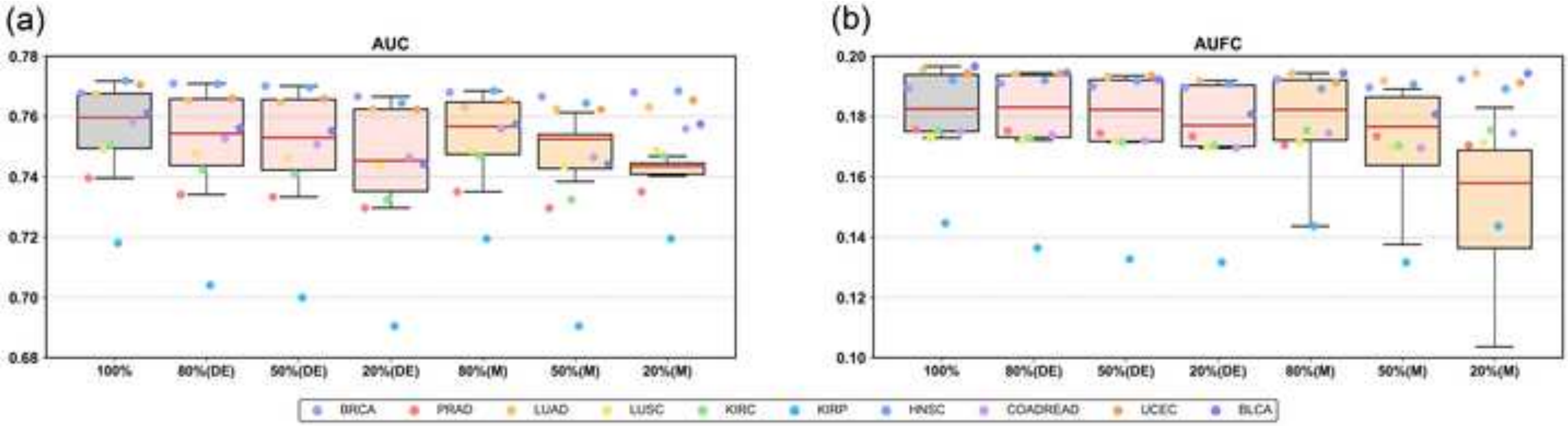

Fig. 7

Click here to access/download;Figure;Figure7.tif

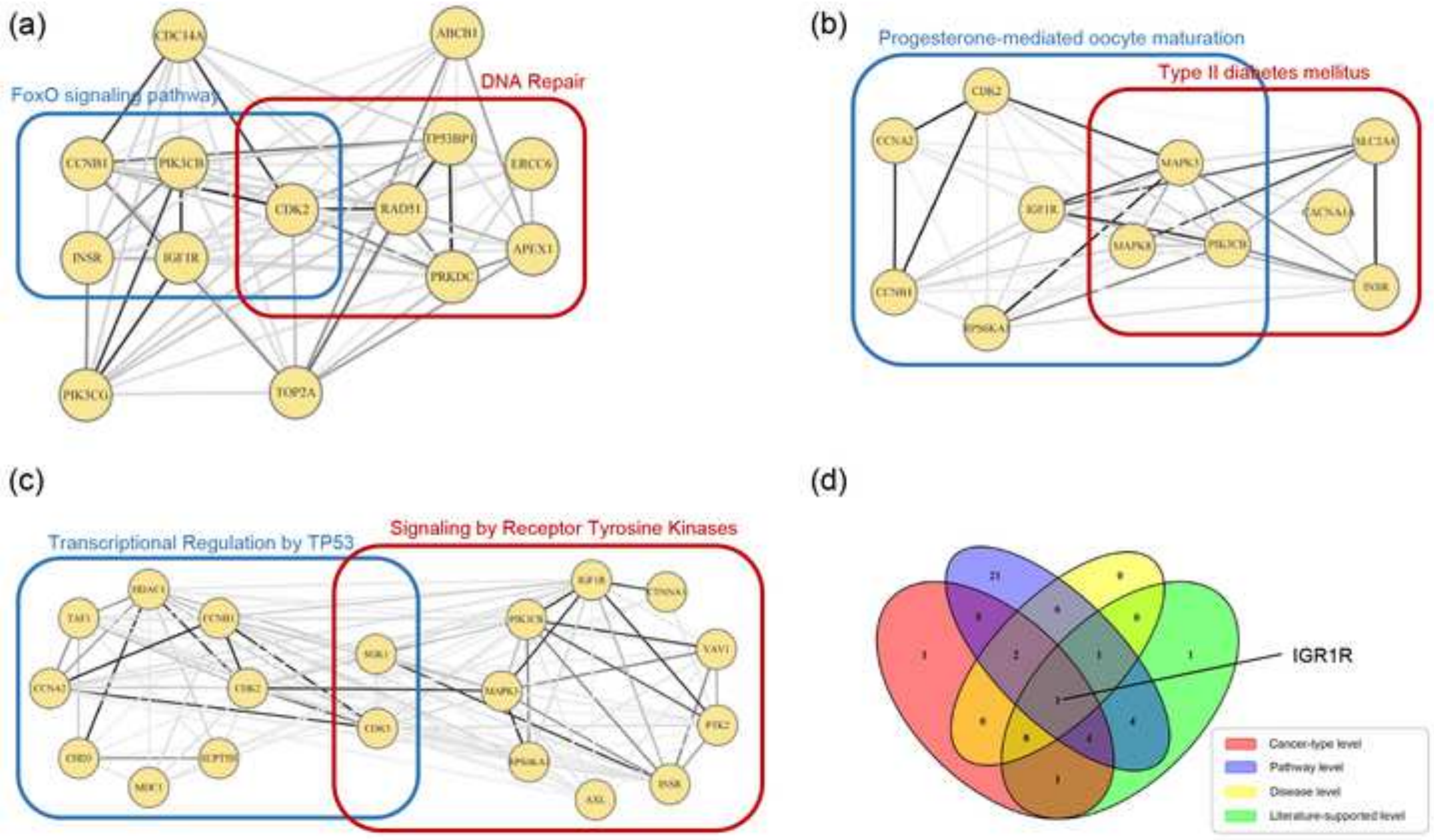

Fig. 8 Click here to access/download;Figure;Figure8.tif

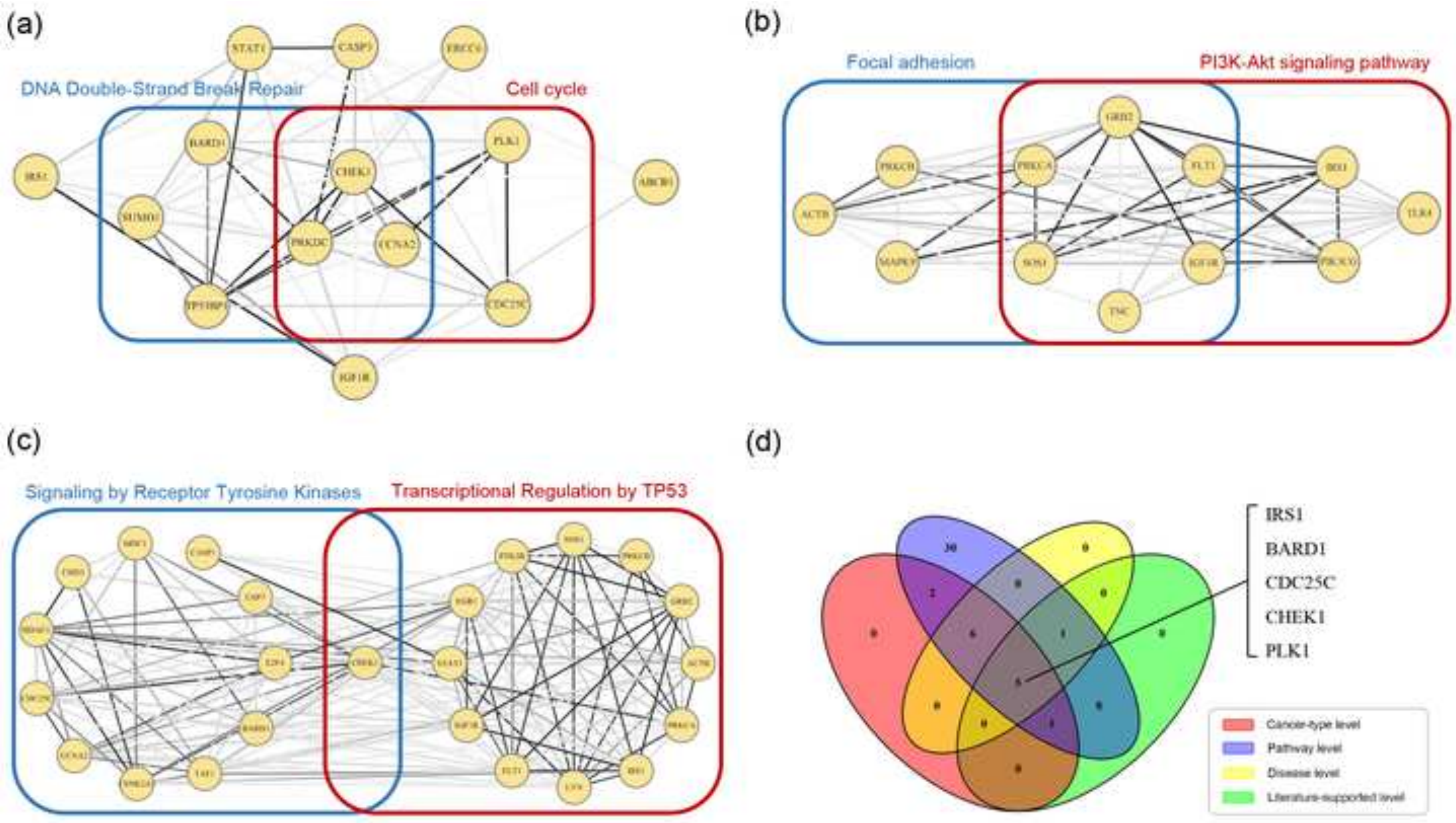

Fig. 9

Click here to access/download;Figure;Figure9.tif

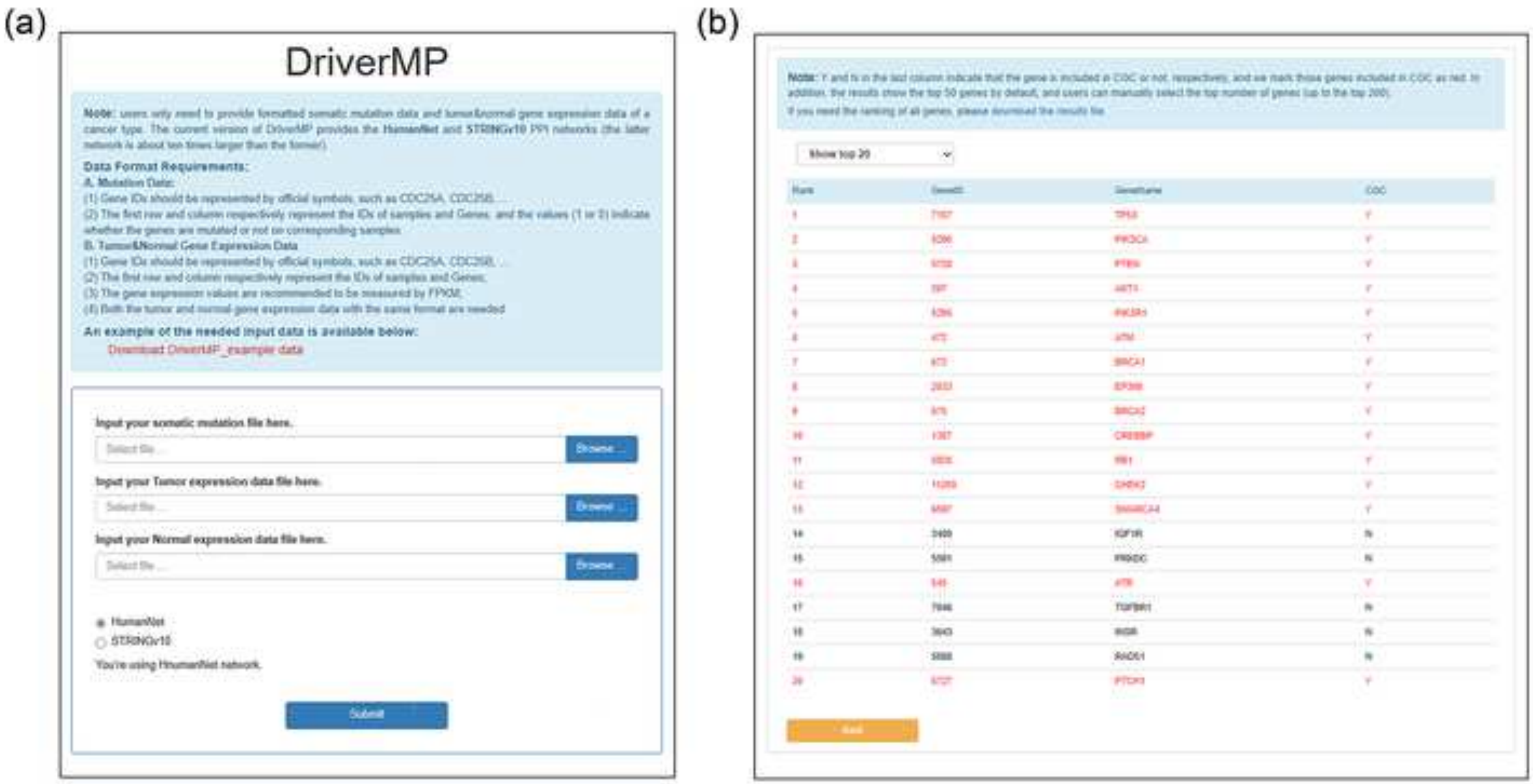

Fig. 10

Click here to access/download;Figure;Figure10.tif

LiuLab　　Home　Server　Contact

Download the database

Database of Evidence-supported Novel Drivers Predicted by DriverMP

BRCA

Gene	NCBI Entrez ID	Related Cancer	Function
AXL	558	BRCA	In breast cancer AXL expression has been observed in all of the main transcriptional subtypes, and AXL expression in primary breast tumors is strongly predictive of reduced patient survival and poor outcome.
CCNA2	890	BRCA	Kaplan–Meier survival analyses confirmed that elevated CCNA2, and CCNB1 expression levels were associated with overall and post-progression survival and recurrence-free probability rates in patients with BRCA.
CCNB1	891	BRCA	CCNB1 is a biomarker for the prognosis of ER+ breast cancer and monitoring of hormone therapy efficacy.
CDK5	1020	BRCA	CDK5 is commonly overexpressed and significantly correlated with several poor prognostic parameters of breast cancer. Its overexpression also exhibited a potential synergy in promoting TGF-β1-induced EMT.
ERCC6	2074	BRCA	Integrative genomics approach suggests that ERCC6 may be a previously unreported low- to moderate-risk breast cancer susceptibility gene, which may also interact with ERCC8.
GLI3	2737	BRCA	ERα+ BrCa cell growth is dependent on Gli3, which indicate that Gli might be a preferential target for the clinical management of ERα+ BrCa.
IGF1R	3480	BRCA	IGF1R, as part of insulin-like growth factor (IGF) signaling, is highly overexpressed in most malignant tissues where it functions as an anti-apoptotic agent by enhancing cell survival.
NCL	4691	BRCA	NCL is commonly overexpressed in human breast tumors and that its expression correlates with that of NCL-dependent miRNAs.
PRKDC	5591	BRCA	PRKDC are all involved with the growth and development of breast cancer cells.
RAD51	5888	BRCA	Breast cancer driver gene BRCA2 directed the binding of RAD51 recombinase to ssDNA, reduced the binding of RAD51 to duplex DNA and stimulated RAD51-mediated DNA strand exchange.
SUPT5H	6829	BRCA	SUPT5H plays an important role in BrCa tumorigenicity by regulating the expression levels of genes that control the proliferation, migration, cell cycle and apoptosis of breast cancer MDA-MB-231 cells.
TP53BP1	7158	BRCA	TP53BP1 may be associated with breast cancer staging and breast cancer prognosis.

Click here to access/download
Supplementary Material
Supplementary Materials.pdf

Personal Cover
Click here to access/download;Personal Cover;Cover Letter.doc

Shandong University
School of Mathematics and Statistics
Address: 180 Wenhuaxi Road,
Weihai, Shandong Province
Postal Code: 264209

July 19, 2023

Dear editor,

We are submitting the enclosed manuscript "**DriverMP enables improved identification of cancer driver mutations**" for your consideration.

It is widely accepted that cancer is mainly caused by genetic mutations. For this reason, substantial genetic and genomic efforts have been undertaken to identify causal cancer driver genes. However, the identification of driver mutations from a large amount of passenger mutations is a highly challenging computational task. In this study, we made three main contributions in the identification of cancer drivers as follows.

(1) According to our observation that most driver mutations have a driver neighbour in the PPI network, we developed a new computational method named DriverMP for effectively prioritizing altered genes based on ranking mutated gene pairs. In this study, a mutation pair is ranked highly if it, as well as the subnetwork centred on it, are highly differentially expressed, or it is strongly associated with other mutations in a biological network. In comparison with nine other state-of-the-art methods, DriverMP achieved the following improvements.

• DriverMP demonstrates much better overall performance than all the compared methods in identifying known driver mutations.

• DriverMP shows great improvement in identifying top-ranked mutations under multiple evaluation criteria.

• DriverMP keeps very stable performance when the sample number is reduced by 20%, 50%, and 80%.

(2) We proposed a cancer-specific five-level assessment by a comprehensive analysis to fully assess the quality of the novel driver candidates predicted by DriverMP. Results showed that DriverMP reliably predicts novel driver genes on all the ten cancer types. In addition, a database of those novel driver mutations was developed, and the relationships between the genes and the corresponding cancer types are detailed in the database (see **Figure 1** at the end of the letter).

(3) A user-friendly web server for use of the DriverMP tool was developed for the convenience of users. After entering the web server, users only need to upload their datasets and press the "submit" button to start the running of DriverMP. After running, the results will be directly displayed on the web page and can also be downloaded from our page (see **Figure 2** at the end of the letter).

The DriverMP method, the database of reliable novel drivers, and the user-friendly online server are expected to contribute to new diagnostic and therapeutic opportunities for cancers.

Thanks for your consideration. If you have any questions, please do not hesitate to contact me.

Yours sincerely,

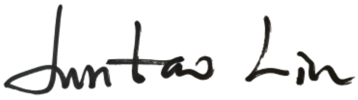

Juntao Liu, PhD
Associate Professor
E-mail: juntaosdu@126.com
Lab: https://www.liulab.top

==

LiuLab   Home   Server   Contact

Download the database

Database of Evidence-supported Novel Drivers Predicted by DriverMP

BRCA

Gene	NCBI Entrez ID	Related Cancer	Function
AXL	558	BRCA	In breast cancer AXL expression has been observed in all of the main transcriptional subtypes, and AXL expression in primary breast tumors is strongly predictive of reduced patient survival and poor outcome.
CCNA2	890	BRCA	Kaplan–Meier survival analyses confirmed that elevated CCNA2, and CCNB1 expression levels were associated with overall and post-progression survival and recurrence-free probability rates in patients with BRCA.
CCNB1	891	BRCA	CCNB1 is a biomarker for the prognosis of ER+ breast cancer and monitoring of hormone therapy efficacy.
CDK5	1020	BRCA	CDK5 is commonly overexpressed and significantly correlated with several poor prognostic parameters of breast cancer. Its overexpression also exhibited a potential synergy in promoting TGF-β1-induced EMT.
ERCC6	2074	BRCA	Integrative genomics approach suggests that ERCC6 may be a previously unreported low- to moderate-risk breast cancer susceptibility gene, which may also interact with ERCC8.
GLI3	2737	BRCA	ERα+ BrCa cell growth is dependent on Gli3, which indicate that Gli might be a preferential target for the clinical management of ERα+ BrCa.
IGF1R	3480	BRCA	IGF1R, as part of insulin-like growth factor (IGF) signaling, is highly overexpressed in most malignant tissues where it functions as an anti-apoptotic agent by enhancing cell survival.
NCL	4691	BRCA	NCL is commonly overexpressed in human breast tumors and that its expression correlates with that of NCL-dependent miRNAs.
PRKDC	5591	BRCA	PRKDC are all involved with the growth and development of breast cancer cells.
RAD51	5888	BRCA	Breast cancer driver gene BRCA2 directed the binding of RAD51 recombinase to ssDNA, reduced the binding of RAD51 to duplex DNA and stimulated RAD51-mediated DNA strand exchange.
SUPT5H	6829	BRCA	SUPT5H plays an important role in BrCa tumorigenicity by regulating the expression levels of genes that control the proliferation, migration, cell cycle and apoptosis of breast cancer MDA-MB-231 cells.
TP53BP1	7158	BRCA	TP53BP1 may be associated with breast cancer staging and breast cancer prognosis.

Figure 1. Searchable database page. We applied DriverMP to ten different cancer types and obtained 85 reliable driver gene candidates not included in CGC, based on which we built a database of the novel drivers.

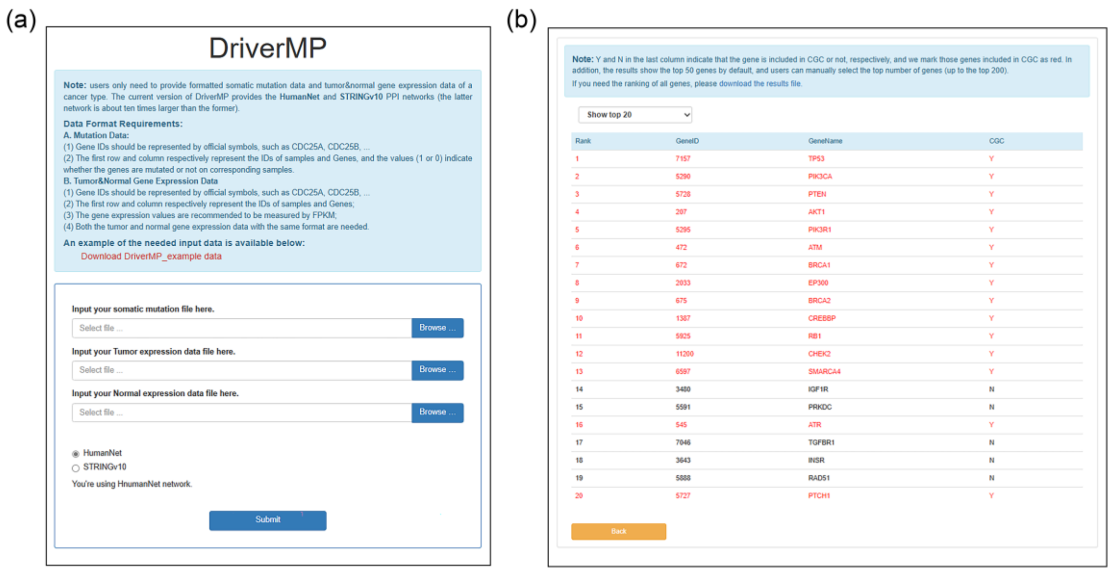

Figure 2. The online web server of DriverMP. **a)** The interface of the online service. Users only need to submit formatted somatic mutation data and tumour and normal gene expression data, choose one of the two PPI networks (HumanNet or STRINGv10), and press the "submit" button to start DriverMP. **b)** Results page. The top 50 gene candidates ranked by DriverMP are displayed by default, and users can manually choose to show the top 10, 20, 50, 100 and 200 genes. To view the full output, users can download the result file.
