# Supplementary material for: DriverMP enables improved identification of cancer driver genes
Source: Gigascience. 2023 Dec 13;12:giad106. doi: 10.1093/gigascience/giad106 (PMC10716827; doi:10.1093/gigascience/giad106)
Supplement: giad106_Supplemental_File [file giad106_supplemental_file.pdf]

Supplemental Material

DriverMP enables improved identification of cancer driver mutations

Yangyang Liu[1], Jiyun Han[1], Tongxin Kong[1], Nannan Xiao[1], Qinglin Mei[2], Juntao Liu[1,*]

[1]School of Mathematics and Statistics, Shandong University (Weihai), Weihai, 264209, China

[2]MOE Key Laboratory of Bioinformatics, BNRIST Bioinformatics Division, Department of Automation, Tsinghua University, Beijing, China

Table of Content

Supplemental Results

Six-level assessment of PRAD

Six-level assessment of LUSC

Six-level assessment of HNSC

Six-level assessment of COADREAD

Six-level assessment of BLCA

Six-level assessment of UCEC

Six-level assessment of KIRC

Six-level assessment of KIRP

Supplemental Figures

Supplemental Tables

References

Supplementary Discussion

This section demonstrates six-level assessments of the eight cancer types PRAD, LUSC, HNSC, COADREAD, BLCA, UCEC, KIRC and KIRP.

Six-level assessment of PRAD

Prostate cancer is the most common solid cancer in men (diagnosed in 12%) and is often fatal (9% of male cancer deaths) [1]. We identified a total of 71 novel driver candidates that ranked within the top 250 in both networks and were not previously included in CGC. These candidates underwent the six-level assessment, and were subsequently divided into six levels (Table S4).

Cancer-type level. We found that 51 (71.8%) of 71 driver candidates were enriched in "cancer" ($p = 2.9 \times 10^{-18}, FDR = 5.3 \times 10^{-17}$), and 14 (19.7%) candidates were included in "prostate cancer" ($p = 1.4 \times 10^{-6}, FDR = 1.1 \times 10^{-4}$). Therefore, the cancer-type level consists of 14 novel genes that are noted as being related to prostate cancer. And interestingly, five of them were enriched in the "FoxO signaling pathway" (KEGG, $FDR = 8.3 \times 10^{-6}$, Fig. S4a).

Literature-supported level. Based on the comprehensive survey of reliable literature, 18 novel genes supported by relevant studies were classified into this level (Table S5).

Pathway level. To investigate whether the enriched biological pathways are associated with prostate cancer, pathway enrichment was performed using the 71 driver candidates. A total of 9 and 15 prostate cancer-related biological pathways were identified using the KEGG and Reactome databases, respectively, covering 58 (81.69%) of the 71 driver candidates (Table S6). For example, 12 (16.9%) and 9 (15.0%) of the 71 genes are respectively enriched in "MicroRNAs in cancer" ($FDR = 3.8 \times 10^{-10}$) and "FoxO signaling pathway" ($FDR = 1.6 \times 10^{-7}$) against KEGG (Fig. S4b). 12 (16.9%) and 11 (15.5%) genes are respectively enriched in "Regulation of TP53 Activity" ($FDR = 4.6 \times 10^{-10}$) and "DNA Repair" ($FDR = 3.0 \times 10^{-6}$) against Reactome (Fig. S4c).

Non-cancer disease level. Disease analysis did not enrich for diseases related to prostate cancer.

Gene level. We analyzed the homology between the 71 driver candidates and the genes in CGC, and a total of 23 (32.4%) genes have homologues in CGC, which are shown in Table S7. In

particular, homologues AR [2], CDKN1B [3], NOTCH2 [4], PRDM1 [5], and ZBTB16 [6] in CGC are associated with prostate cancer, corresponding to the driver candidates NR3C1, CDKN1A, PTGS2, SP1, and SP3 predicted by DriverMP.

Validation-required level. There are seven genes belong to this level. They were ranked high in DriverMP, but their direct relationship with prostate cancer were unclear based on this study. Future investigations may discover the associations of these genes with prostate cancers.

Subsequently, to visualize the relationship among the four levels, we drew a Venn graph and found that CDKN1A appeared in these levels (Fig. S4d). Analysis of CDKN1A genotypes may prove useful in determining which patients are at risk for developing advanced prostate carcinoma [7].

In addition, genes of cancer-type level form a significantly dense and highly weighted sub-network in the PPI network. The number of edges and sum of edge weights of them in the sub-network in STRINGv10 are 50 and 19.89 compared to 2.19 and 0.65 at ramdom with *p-value* = 0.

Six-level assessment of LUSC

We identified a total of 68 novel driver candidates that ranked within the top 250 in both networks and were not previously included in CGC. These candidates underwent the six-level assessment, and were subsequently divided into six levels (Table S8).

Cancer-type level. We found that 45 (66.2%) of 68 driver candidates were enriched in "cancer" ($p = 2.7 \times 10^{-15}, FDR = 4.9 \times 10^{-14}$), and 15 (22.1%) candidates were included in "lung cancer" ($p = 7.0 \times 10^{-8}, FDR = 1.3 \times 10^{-5}$). Therefore, the cancer-type level consists of 15 novel genes that are noted as being related to lung cancer. And interestingly, six of them were enriched in the "Cell Cycle Checkpoints" (Reactome, $FDR = 7.1 \times 10^{-5}$, Fig. S5a).

Literature-supported level. Based on the comprehensive survey of reliable literature, 14 novel genes supported by relevant studies were classified into this level (Table S9).

Pathway level. To investigate whether the enriched biological pathways are associated with prostate cancer, pathway enrichment was performed using the 68 driver candidates. A total of 12 and 12 lung cancer-related biological pathways were identified using the KEGG and Reactome databases, respectively, covering 55 (80.88%) of the 68 driver candidates

(Table S10). For example, 9 (13.2%) and 12 (17.6%) of the 68 genes were respectively enriched in "Cell cycle" ($FDR = 2.1 \times 10^{-7}$) and "PI3K-Akt signaling pathway" ($FDR = 6.2 \times 10^{-7}$) against KEGG (Fig. S5b). 18 (26.5%) and 21 (30.9%) genes were respectively enriched in "Cell Cycle" ($FDR = 1.6 \times 10^{-8}$) and "Generic Transcription Pathway" ($FDR = 4.6 \times 10^{-7}$) against Reactome (Fig. S5c)

Non-cancer disease level. In addition to the "cancer-type level" discussed above, we also found that 11 of the 68 driver candidates were enriched in "chronic obstructive pulmonary disease", a lung cancer related disease, which constructed the "non-cancer disease level".

Gene level. We analyzed the homology between the 68 driver candidates and the genes in CGC, and a total of 21 (30.88%) genes have homologues EGFR [8], FGFR2 [9], FGFR3 [9], PDGFRA [10], and PIK3CA [11] in CGC, which are shown in Table S11. In particular, homologues in CGC are associated with LUSC, corresponding to the driver candidates FLT1, IGF1R, INSR and PRKDC predicted by DriverMP.

Validation-required level. There are eight genes belong to this level. They were ranked high in DriverMP, but their direct relationship with lung cancer were unclear based on this study. Future investigations may discover the associations of these genes with lung cancers.

Subsequently, to visualize the relationship among the five levels, we drew a Venn graph and found that two genes PARP1 and CDK5 appeared in these levels (Fig. S5d). The inhibition of PARP1 can provide a potential targeted therapy and prevention of inflammation-associated lung carcinogenesis [12]. Recent reports suggest that high CDK5 expression is associated with shorter overall survival (OS) in lung cancer and promotes proliferation and metastasis of lung cancer cells [13].

In addition, genes of cancer-type level form a significantly dense and highly weighted sub-network in the PPI network. The number of edges and average edge weights of them in the sub-network in STRINGv10 are 89 and 44.26, compared to 2.53 and 0.75 at ramdom with *p-value* = 0.

Six-level assessment of HNSC

We identified a total of 65 novel driver candidates that ranked within the top 250 in both networks and were not previously included in CGC. These candidates underwent the six-level assessment, and were subsequently divided into six levels (Table S12).

Cancer-type level. We found that 44 (67.7%) of 65 driver candidates were enriched in "cancer" ($p = 1.0 \times 10^{-15}, FDR = 1.8 \times 10^{-14}$), and 6 (9.2%) candidates were included in "head and neck cancer" ($p = 4.9 \times 10^{-5}, FDR = 2.2 \times 10^{-3}$). Therefore, the cancer-type level consists of 6 novel genes that are noted as being related to head and neck cancer. And interestingly, four of them were enriched in the "Pathway in cancer" (KEGG, $p = 2.5 \times 10^{-3}$, Fig. S6a).

Literature-supported level. Based on the comprehensive survey of reliable literature, 12 novel genes supported by relevant studies were classified into this level (Table S13).

Pathway level. To investigate whether the enriched biological pathways are associated with head and neck cancer, pathway enrichment was performed using the 65 driver candidates. A total of 8 and 14 head and neck cancer-related biological pathways were identified using the KEGG and Reactome databases, respectively, covering 52 (80.00%) of the 65 driver candidates (Table S14). For example, 10 (15.6%) and 17 (26.2%) of the 65 candidates were respectively enriched in "Endocrine resistance" ($FDR = 3.6 \times 10^{-10}$) and "Pathways in cancer" ($FDR = 3.6 \times 10^{-10}$) against KEGG (Fig. S6b). 18 (27.7%) and 14 (21.5%) genes were respectively enriched in "Transcriptional Regulation by TP53" ($FDR = 1.2 \times 10^{-13}$) and "DNA Repair" ($FDR = 1.0 \times 10^{-9}$) against Reactome (Fig. S6c).

Non-cancer disease level. Disease analysis did not enrich for diseases related to head and neck cancer.

Gene level. We analyzed the homology between the 65 driver candidates and the genes in CGC, and a total of 26 (40.00%) genes have homologues in CGC, which are shown in Table S15. In particular, homologues CTCF [14], FAT1 [15], FGFR3 [16], MAPK [17], NOTCH1 [18, 19], and PIK3CA [20] in CGC are associated with HNSC, corresponding to the driver candidates EGR1, NOTCH3, IGF1R, MAPK8, MAPK9, MAPK10, and PRKDC predicted by DriverMP.

Validation-required level. There are seven genes belong to this level. They were ranked high in DriverMP, but their direct relationship with head and neck cancer were unclear based on this study. Future investigations may discover the associations of these genes with head and neck cancers.

Subsequently, to visualize the relationship among the four levels, we drew a Venn graph and found that two genes IGF1R and PTGS2 appeared in these levels (Fig. S6d). IGF1R was upregulated in HNSCC compared to paired benign oropharyngeal epithelial cells, with 36%

of tumors having prominent IGF1R signals on the plasma membrane and 92% on the cytoplasm [21]. Recent studies have identified EGFR and PTGS2 as key nodes of the gene regulatory network in head and neck cancer through an integrative multi-omics approach, and several DEGs associated with the immune phenotype are affected by EGFR inhibition in tumor cell lines [22].

In addition, genes of cancer-type level form a significantly dense and highly weighted sub-network in the PPI network. The number of edges and average edge weights of them in the sub-network in STRINGv10 are 11 and 3.44, compared to 0.36 and 0.11 at ramdom with *p-value* = 0.

Six-level assessment of COADREAD

We identified a total of 60 novel driver candidates that ranked within the top 250 in both networks and were not previously included in CGC. These candidates underwent the six-level assessment, and were subsequently divided into six levels (Table S16).

Cancer-type level. We found that 42 (70.0%) of 60 driver candidates were enriched in "cancer" ($p = 7.3 \times 10^{-14}, FDR = 1.3 \times 10^{-12}$), and 13 (21.7%) candidates were included in "colorectal cancer" ($p = 2.6 \times 10^{-7}, FDR = 2.3 \times 10^{-5}$). Therefore, the cancer-type level consists of 13 novel genes that are noted as being related to colorectal cancer. And interestingly, five of them were enriched in the "DNA Repair" (KEGG, $FDR = 1.6 \times 10^{-3}$, Fig. S7a).

Literature-supported level. Based on the comprehensive survey of reliable literature, 16 novel genes supported by relevant studies were classified into this level (Table S17).

Pathway level. To investigate whether the enriched biological pathways are associated with colorectal cancer, pathway enrichment was performed using the 60 driver candidates. A total of 19 and 12 colorectal cancer -related biological pathways were identified using the KEGG and Reactome databases, respectively, covering 56 (93.33%) of the 60 driver candidates (Table S18). For example, 18 (30.0%) and 13 (21.7%) of the 60 candidates were respectively enriched in "Pathways in cancer" ($FDR = 5.6 \times 10^{-12}$) and "Focal adhesion" ($FDR = 9.8 \times 10^{-12}$) against KEGG (Fig. S7b). 14 (23.3%) and 23 (38.3%) genes were respectively enriched in "DNA Repair" ($FDR = 8.0 \times 10^{-10}$) and "Generic Transcription Pathway" ($FDR = 8.0 \times 10^{-10}$) against Reactome (Fig. S7c).

Non-cancer disease level. Disease analysis did not enrich for diseases related to colorectal

cancer.

Gene level. We analyzed the homology between the 65 driver candidates and the genes in CGC, and a total of 26 (40.00%) genes have homologues in CGC, which are shown in Table S19. In particular, homologues ERBB2 [23], ERBB4 [24], FAT4 [25], PIK3CA [26], and SMARCA4 [27] in CGC are associated with COADREAD, corresponding to the driver candidates FYN, PTK2, NOTCH3, PRKDC, and SMARCA2 predicted by DriverMP.

Validation-required level. There are two genes belong to this level. They were ranked high in DriverMP, but their direct relationship with colorectal cancer were unclear based on this study. Future investigations may discover the associations of these genes with colorectal cancers.

Subsequently, to visualize the relationship among the four levels, we drew a Venn graph and found that IGF1R, appeared in these levels (Fig. S7d). Overexpression of RTKs, including IGFIR, has been demonstrated in CRC. Several experimental studies have shown that IGF1R plays a role in resistance to anti-EGFR therapy in mCRC patients treated with cetuximab [28].

In addition, genes of cancer-type level form a significantly dense and highly weighted sub-network in the PPI network. The number of edges and average edge weights of them in the sub-network in STRINGv10 are 51 and 22.65, compared to 1.88 and 0.56 at ramdom with p-value = 0.

Six-level assessment of BLCA

We identified a total of 70 novel driver candidates that ranked within the top 250 in both networks and were not previously included in CGC. These candidates underwent the Six-level assessment, and were subsequently divided into six levels (Table S20).

Cancer-type level. We found that 48 (68.6%) of 70 driver candidates were enriched in "cancer" ($p = 7.8 \times 10^{-16}, FDR = 1.4 \times 10^{-14}$), and 17 (24.3%) candidates were included in "bladder cancer" ($p = 5.0 \times 10^{-9}, FDR = 7.0 \times 10^{-7}$). Therefore, the cancer-type level consists of 17 novel genes that are noted as being related to bladder cancer. And interestingly, seven of them were enriched in the "Cell cycle" (KEGG, $FDR = 2.5 \times 10^{-9}$, Fig. S8a).

Literature-supported level. Based on the comprehensive survey of reliable literature, 16

novel genes supported by relevant studies were classified into this level (Table S21).

Pathway level. To investigate whether the enriched biological pathways are associated with bladder cancer, pathway enrichment was performed using the 70 driver candidates. A total of 11 and 15 bladder cancer-related biological pathways were identified using the KEGG and Reactome databases, respectively, covering 57 (81.43%) of the 70 driver candidates (Table S22). For example, 12 (17.1%) and 11 (15.7%) candidates were respectively enriched in "Cell cycle" ($FDR = 1.3 \times 10^{-11}$) and "Thyroid hormone signaling pathway" ($FDR = 1.8 \times 10^{-10}$) against KEGG (Fig. S8b). 12 (17.1%) and 10 (14.3%) candidates were respectively enriched in "SUMO E3 ligases SUMOylate target proteins" ($FDR = 4.4 \times 10^{-10}$) and "Epigenetic regulation of gene expression" ($FDR = 5.7 \times 10^{-9}$) against Reactome (Fig. S8c).

Non-cancer disease level. Disease analysis did not enrich for diseases related to bladder cancer.

Gene level. We analyzed the homology between the 70 driver candidates and the genes in CGC, and a total of 25 (35.71%) genes have homologues in CGC, which are shown in Table S23. In particular, homologues FGFR3 [29], FLT4 [30], and PIK3CA [31] in CGC are associated with BLCA, corresponding to the driver candidates IGF1R, INSR, PTK2 and PRKDC predicted by DriverMP.

Validation-required level. There are seven genes belong to this level. They were ranked high in DriverMP, but their direct relationship with bladder cancer were unclear based on this study. Future investigations may discover the associations of these genes with bladder cancer.

Subsequently, to visualize the relationship among the four levels, we drew a Venn graph and found that three genes CDKN1A, INSR, and STAT1 appeared in these levels (Fig. S8d). A report of whole genome sequencing of different subtypes of bladder cancer demonstrated CDKN1A as a tumor suppressor gene in bladder cancer [32]. IGF-2/INSR mediated paracrine crosstalk between bladder cancer cells and endothelial cells is functionally involved in tumor angiogenesis and may thus represent a new therapeutic target [33]. One study provided an apparent prognostic prediction model for bladder cancer and identified STAT1 as a key gene in a gene regulatory network related to the immune phenotype of bladder cancer [34].

In addition, genes of cancer-type level form a significantly dense and highly weighted sub-network in the PPI network. The number of edges and average edge weights of them in the sub-

network in STRINGv10 are 110 and 51.70, compared to 3.28 and 0.98 at random with p-value = 0.

Six-level assessment of UCEC

We identified a total of 52 novel driver candidates that ranked within the top 250 in both networks and were not previously included in CGC. These candidates underwent the six-level assessment, and were subsequently divided into six levels (Table S24).

Cancer-type level. We found that 37 (71.2%) of these candidates were enriched in "cancer" ($p = 2.4 \times 10^{-12}, FDR = 4.2 \times 10^{-11}$) against GAD. Unfortunately, no gene was directly enriched in endometrial cancer and therefore the level is missing in this cancer type.

Literature-supported level. Based on the comprehensive survey of reliable literature, 8 novel genes supported by relevant studies were classified into this level (Table S25).

Pathway level. To investigate whether the enriched biological pathways are associated with UCEC, pathway enrichment was performed using the 52 driver candidates. A total of 12 and 9 UCEC-related biological pathways were identified using the KEGG and Reactome databases, respectively, covering 34 (65.38%) of the 52 driver candidates (Table S26). For example, 12 (23.0%) and 14 (26.9%) of them were respectively enriched in "Focal adhesion" ($FDR = 7.9 \times 10^{-11}$) and "PI3K-Akt signaling pathway" ($FDR = 7.9 \times 10^{-11}$) against KEGG (Fig. S9a). 12 (23.0%) and 18 (34.6%) candidates were respectively enriched in "Signaling by VEGF" ($FDR = 4.6 \times 10^{-13}$) and "Signaling by Receptor Tyrosine Kinases" ($FDR = 6.3 \times 10^{-13}$) against Reactome (Fig. S9b).

Non-cancer disease level. Disease analysis did not enrich for diseases related to UCEC.

Gene level. We analyzed the homology between the 52 driver candidates and the genes in CGC, and a total of 25 (48.08%) genes have homologues in CGC, which are shown in Table S27. In particular, homologues ABL1 [35], AKT1 [36], FGFR2 [37], JAK1 [38], and PIK3CA [39] in CGC are associated with UCEC, corresponding to the driver candidates FLT1, INSR, PTK2, AXL, AKT3, SGK1, PRKCA, IGF1R, TEK, TYK2, PRKDC, and PIK3CB predicted by DriverMP.

Validation-required level. There are eleven genes belong to this level. They were ranked high in DriverMP, but their direct relationship with UCEC were unclear based on this study. Future investigations may discover the associations of these genes with UCEC.

Subsequently, to visualize the relationship among the three levels, we drew a Venn graph

and found that three genes IGF1R, SGK1, and AKT3 appeared in these levels (Fig. S9d). A study validated IGF1R as a target for endometrial cancer treatment and clearly demonstrated that IGFIR inhibitors, including tyrosine kinase inhibitors and IGF1R antibodies, have potential therapeutic benefits in endometrial cancer treatment [40]. The increased expression of SGK1 in endometrial cancer tissues suggests a role for SGK1 in this type of cancer. Furthermore, the SGK1 inhibitor SI113 induced a significant decrease in endometrial cancer cell viability, suggesting that SGK1 may be an attractive molecular target for the treatment of endometrial cancer [41]. AKT3 has been observed to be reversely correlated with miR-582-5p and also validated as a direct target of miR-582-5p in endometrial cancer. Moreover, AKT3 is also involved in regulation of cell proliferation and apoptosis in ECC1 cells [42].

Six-level assessment of KIRC

We identified a total of 45 novel driver candidates that ranked within the top 250 in both networks and were not previously included in CGC. These candidates underwent the Six-level assessment, and were subsequently divided into six levels (Table S28).

Cancer-type level. We found that 24 (53.3%) of these candidates were enriched in "cancer" ($p = 5.2 \times 10^{-6}, FDR = 4.7 \times 10^{-5}$) against GAD. Unfortunately, no gene was directly enriched in KIRC and therefore the level is missing in this cancer type.

Literature-supported level. Based on the comprehensive survey of reliable literature, 12 novel genes supported by relevant studies were classified into this level (Table S29).

Pathway level. To investigate whether the enriched biological pathways are associated with KIRC, pathway enrichment was performed using the 45 driver candidates. A total of 2 and 6 KIRC-related biological pathways were identified using the KEGG and Reactome databases, respectively, covering 21 (41.67%) of the 45 driver candidates (Table S30). For example, 8 (17.8%) and 6 (13.3%) of the 45 candidates were respectively enriched in "Cell cycle" ($FDR = 1.6 \times 10^{-7}$) and "Thyroid hormone signaling pathway" ($FDR = 6.6 \times 10^{-5}$) against KEGG (Fig. S10a). 9 (20.0%) and 9 (20.0%) genes were respectively enriched in "Mitotic G1 phase and G1/S transition" ($FDR = 1.5 \times 10^{-7}$) and "Signaling by NOTCH" ($FDR = 5.8 \times 10^{-7}$) against Reactome (Fig. S10b).

Non-cancer disease level. Disease analysis did not enrich for diseases related to KIRC.

Gene level. We analyzed the homology between the 45 driver candidates and the genes in CGC, and a total of 13 (28.89%) genes have homologues in CGC, which are shown in Table S31.

Validation-required level. There are fourteen genes belong to this level. They were ranked high in DriverMP, but their direct relationship with KIRC were unclear based on this study. Future investigations may discover the associations of these genes with KIRC.

Subsequently, to visualize the relationship among the three levels, we drew a Venn graph and found that four genes CDK2, SMARCA2, EGR1, and STAT1 appeared in these levels (Fig. S10c). CDK1 and CDK2 activity is a strong predictor of renal cell carcinoma recurrence [43]. High SMARCA2 expression was associated with good prognosis and benign differentiated tumors in kidney renal clear cell carcinoma [44]. MAML1 may be a component of the transcriptional networks which regulate EGR1 target genes during nephrogenesis and could also have implications for the development of renal cell carcinoma [45]. STAT1 may play a key role in RCC radioresistance and manipulation of this pathway may enhance the efficacy of radiotherapy [46].

Six-level assessment of KIRP

We identified a total of 56 novel driver candidates that ranked within the top 250 in both networks and were not previously included in CGC.

Cancer-type level. We first found that 17 (30.4%) of 56 driver candidates were enriched in "cancer" ($p = 2.3 \times 10^{-2}, FDR = 8.3 \times 10^{-2}$) against GAD. Unfortunately, our KIRP candidates were not enriched in "kidney renal papillary cell carcinoma" in GAD.

Pathway level. Subsequently, we performed a pathway analysis for these candidates. We discovered that 4 (7.1%) and 6 (10.7%) of the 45 candidates are respectively enriched in "RNA polymerase" ($FDR = 9.8 \times 10^{-4}$) and "Hepatocellular carcinoma" ($FDR = 1.3 \times 10^{-3}$) against KEGG (Fig. S11a), 13 (23.2%) and 16 (28.6%) genes are respectively enriched in "Metabolism of RNA" ($FDR = 8.4 \times 10^{-5}$) and "Gene expression (Transcription)" ($FDR = 2.3 \times 10^{-3}$) against Reactome (Fig. S11b).

Non-cancer disease level. Disease analysis did not enrich for diseases related to KIRP.

Gene level. We analyzed the homology between the 56 driver candidates and the genes in CGC, and a total of 11 (19.64%) genes have homologues in CGC, which are shown in Table S32.

Validation-required level. There are twenty-two genes belong to this level. They were ranked high in DriverMP, but their direct relationship with KIRP were unclear based on this study. Future investigations may discover the associations of these genes with KIRP.

Supplementary Figures

Fig. S1-S3 shows comparison of DriverMP and other methods in HumanNet. Fig. S4-S11 illustrate the results of the six-level assessment for eight cancers PRAD, LUSC, HNSC, COADREAD, BLCA, UCEC, KIRC and KIRP.

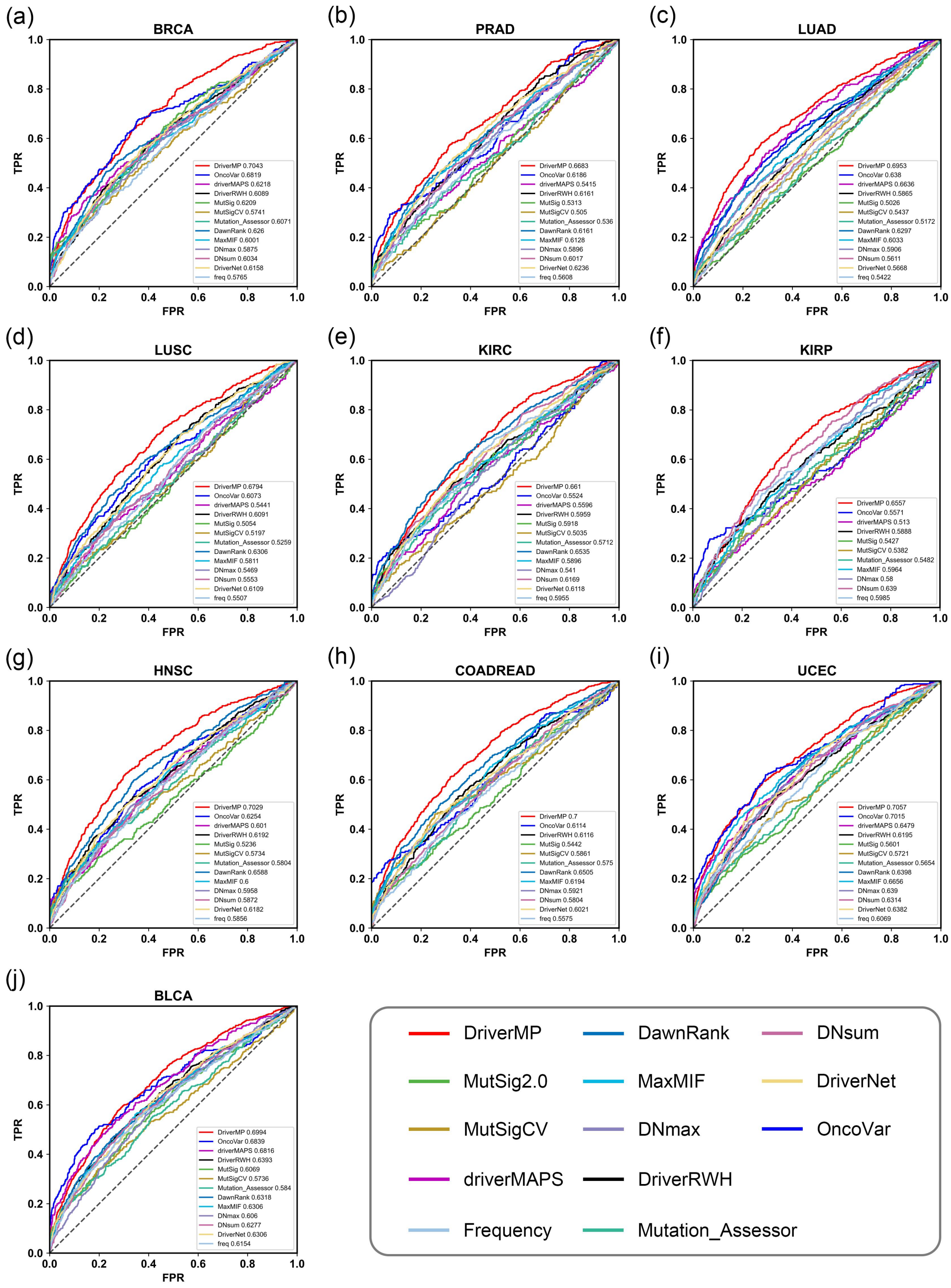

Figure S1. ROC curves of DriverMP and the other compared methods in **a)** BRCA, **b)** PRAD, **c)** LUAD, **d)** LUSC, **e)** KIRC, **f)** KIRP, **g)** HNSC, **h)** COADREAD **i)** UCEC and **j)** BLCA, using HumanNet network.

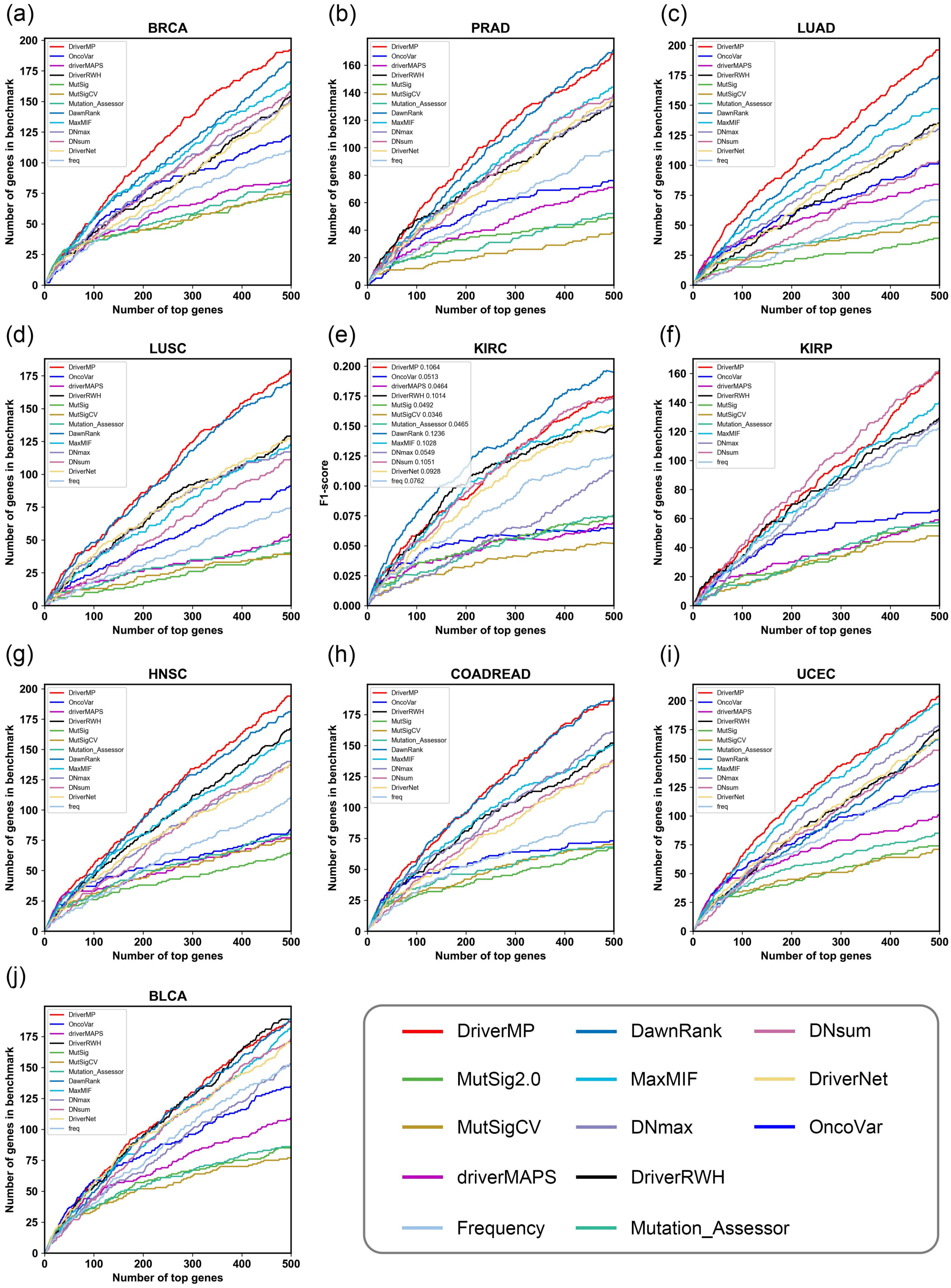

Figure S2. The curves of the numbers of identified known driver mutations of the top ranked 500 genes in **a)** BRCA, **b)** PRAD, **c)** LUAD, **d)** LUSC, **e)** KIRC, **f)** KIRP, **g)** HNSC, **h)** COADREAD, **i)** BLCA and **j)** BLCA, using HumanNet PPI network.

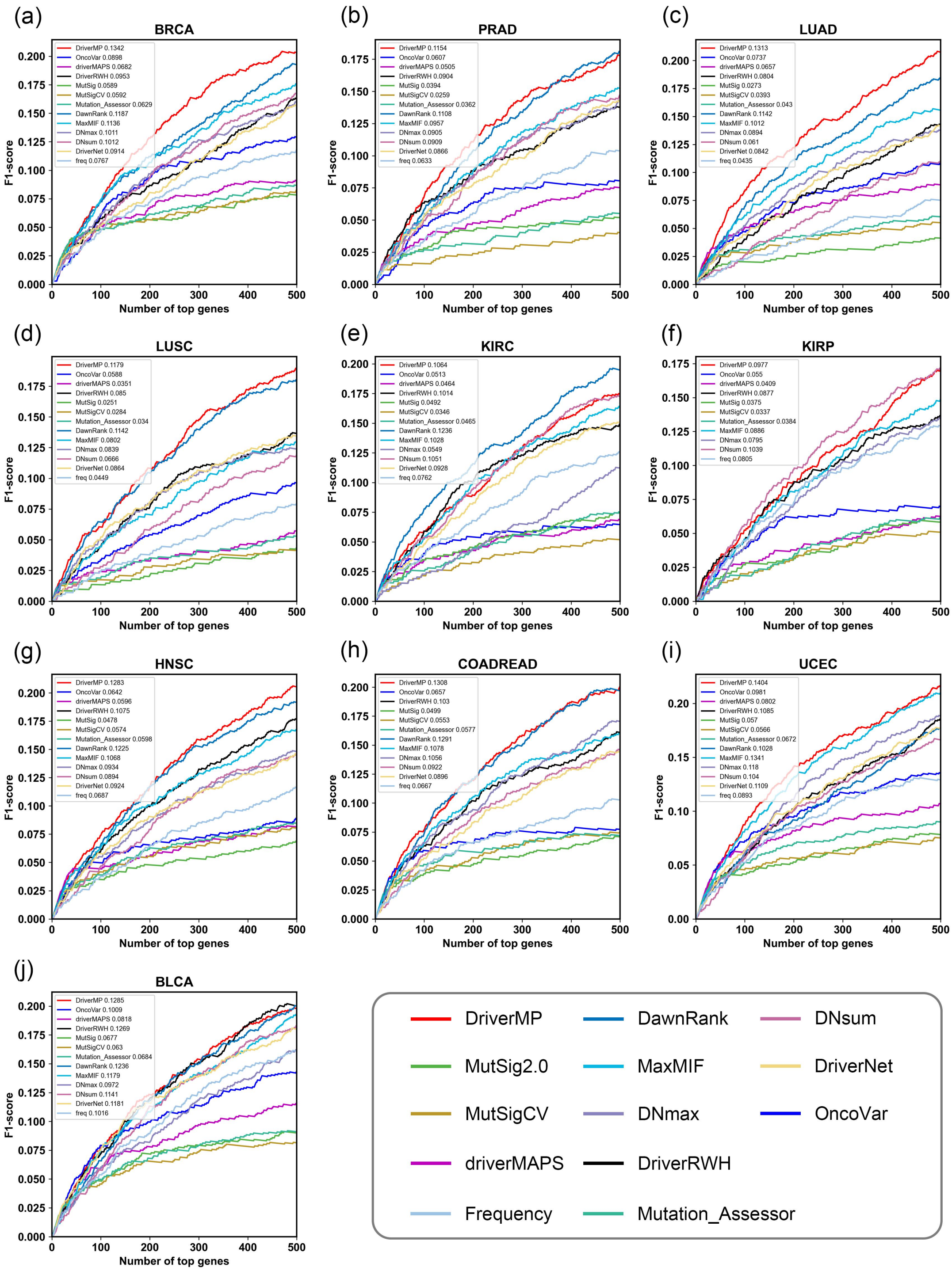

Figure S3. Performance of F1-scores of DriverMP and other compared methods in ten cancer types in **a)** BRCA, **b)** PRAD, **c)** LUAD, **d)** LUSC, **e)** KIRC, **f)** KIRP, **g)** HNSC, **h)** COADREAD, **i)** BLCA and **j)** BLCA, using HumanNet netwrok.

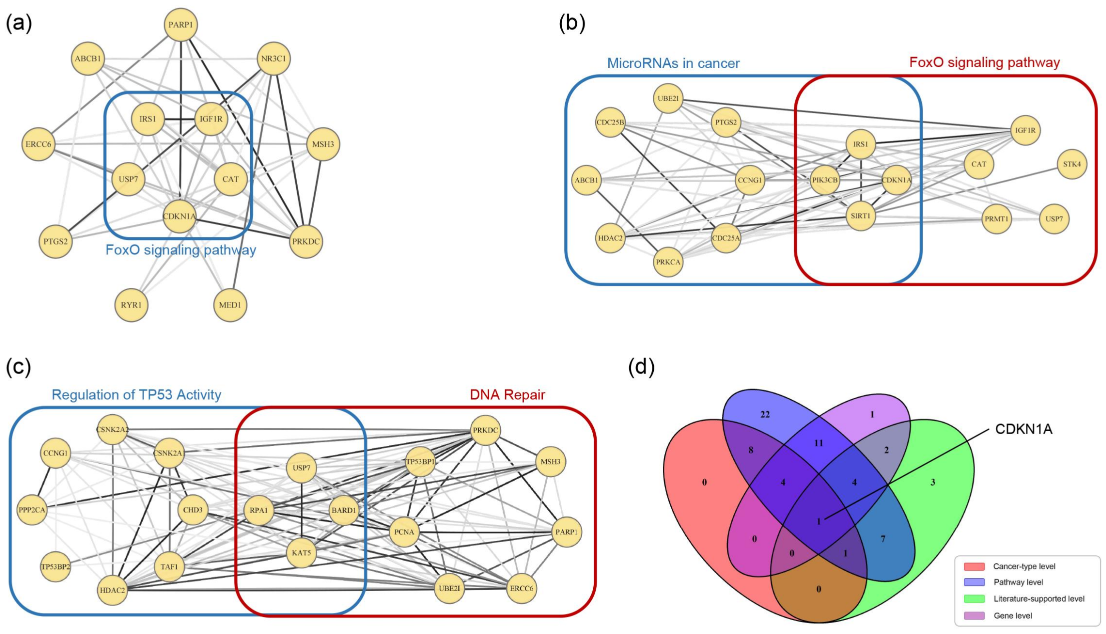

Figure S4. Six-level assessment of PRAD. **a)** Sub-network of the genes in "cancer-type level" from STRINGv10 network. The five genes in the blue box are enriched in "FoxO signaling pathway" (KEGG). **b)** Relationship of genes enriched in two biological pathways against KEGG from STRINGv10 network. **c)** Relationship of genes enriched in two biological pathways against Reactome from STRINGv10 network. **d)** Venn diagram of four levels of prostate cancer.

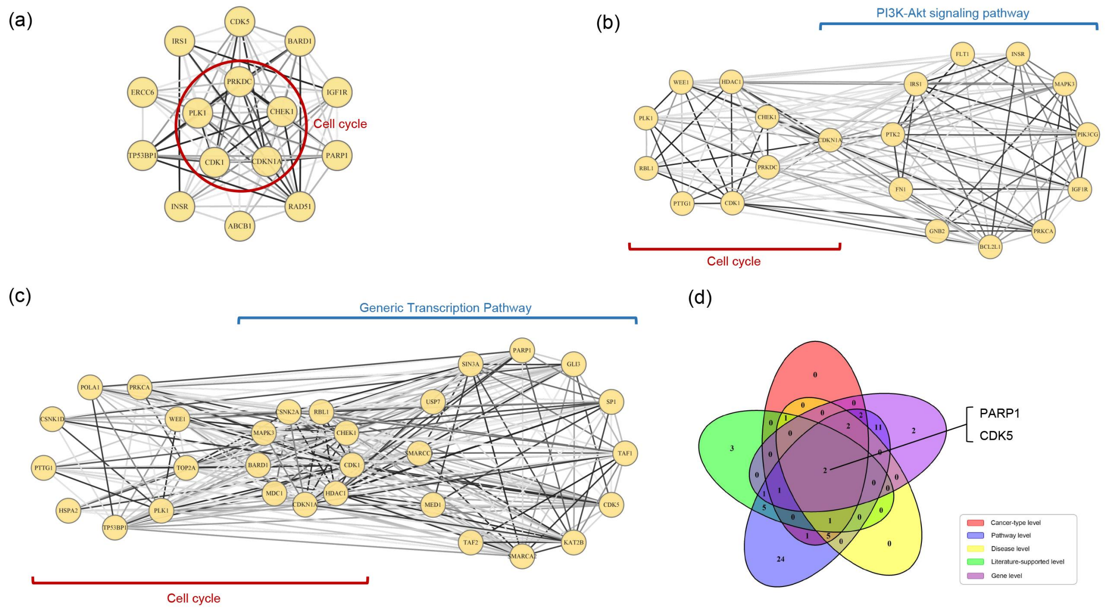

Figure S5. Six-level assessment of LUSC. a) Subgraph of cancer-type level in STRINGv10 network. b) Subgraph of candidates of pathway level (KEGG). c) Subgraph of candidates of pathway level (Reactome). d) Venn diagram of five levels.

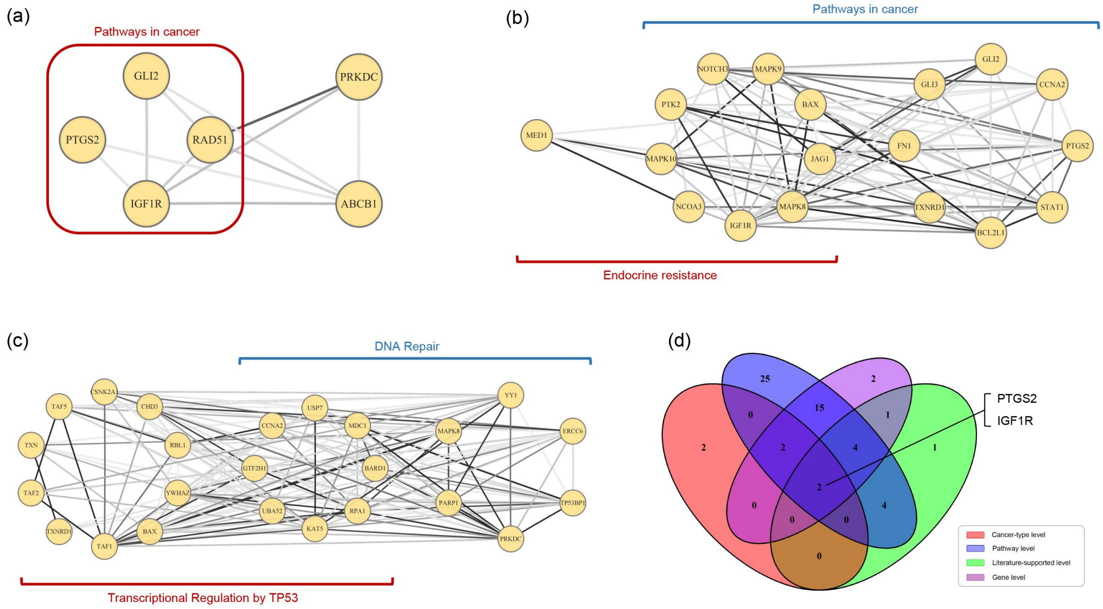

Figure S6. Six-level assessment of HNSC. a) Subgraph of cancer-type level in STRINGv10 network. b) Subgraph of candidates of pathway level (KEGG). c) Subgraph of candidates of pathway level (Reactome). d) Venn diagram of four levels.

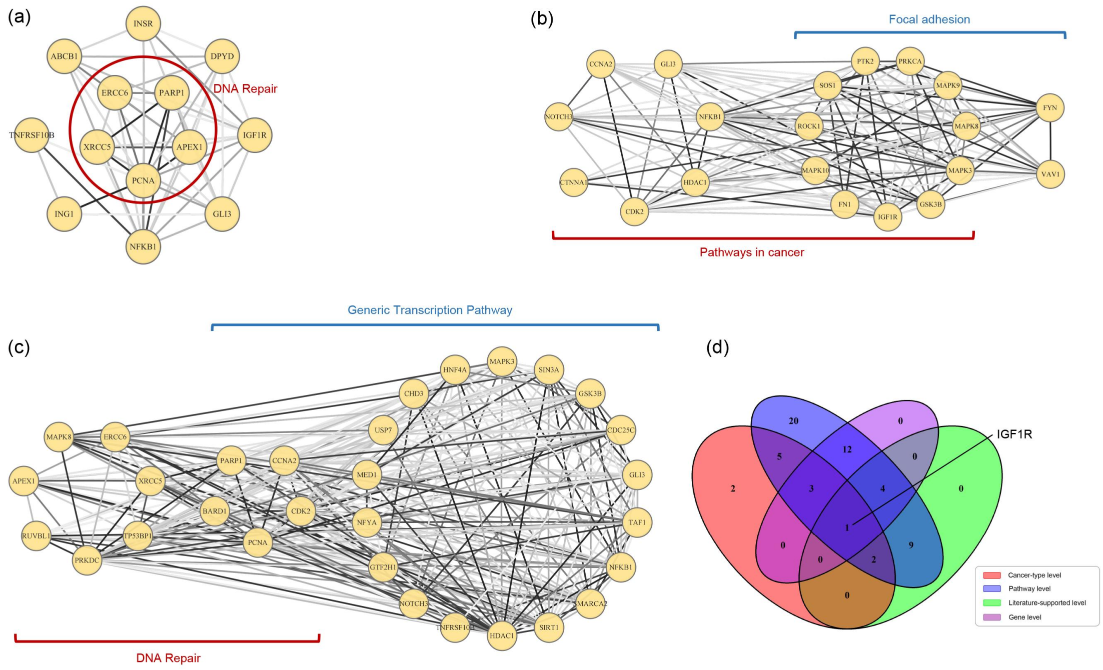

Figure S7. Six-level assessment of COADREAD. a) Subgraph of cancer-type level in STRINGv10 network. b) Subgraph of candidates of pathway level (KEGG). c) Subgraph of candidates of pathway level (Reactome). d) Venn diagram of four levels.

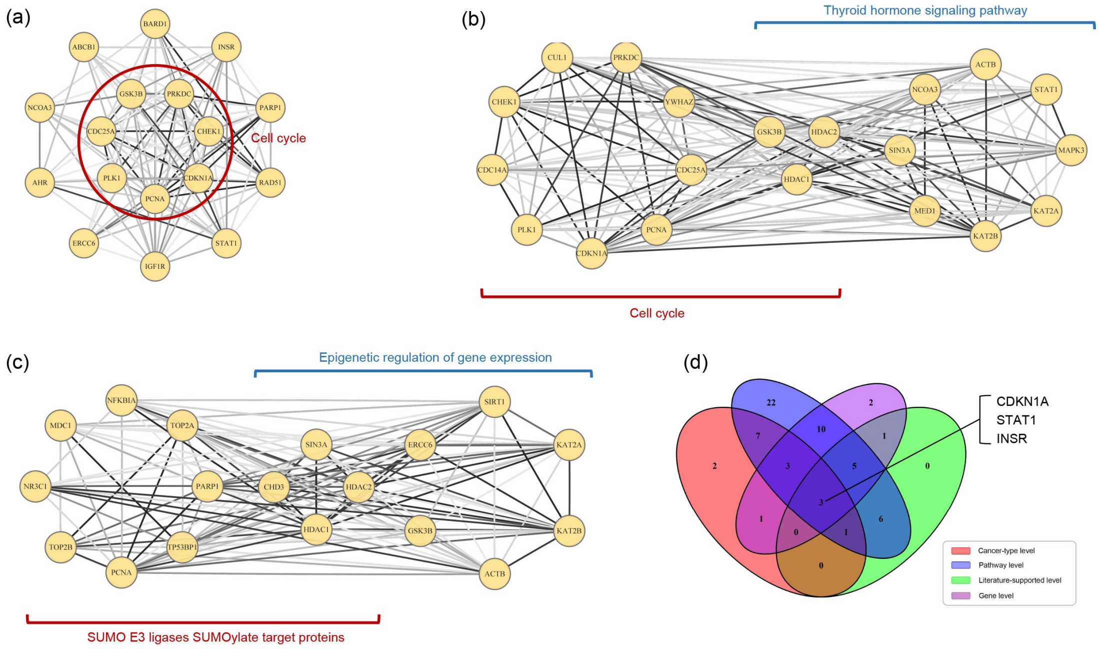

Figure S8. Six-level assessment of BLCA. a) Subgraph of cancer-type level in STRINGv10 network. b) Subgraph of candidates of pathway level (KEGG). c) Subgraph of candidates of pathway level (Reactome). d) Venn diagram of four levels.

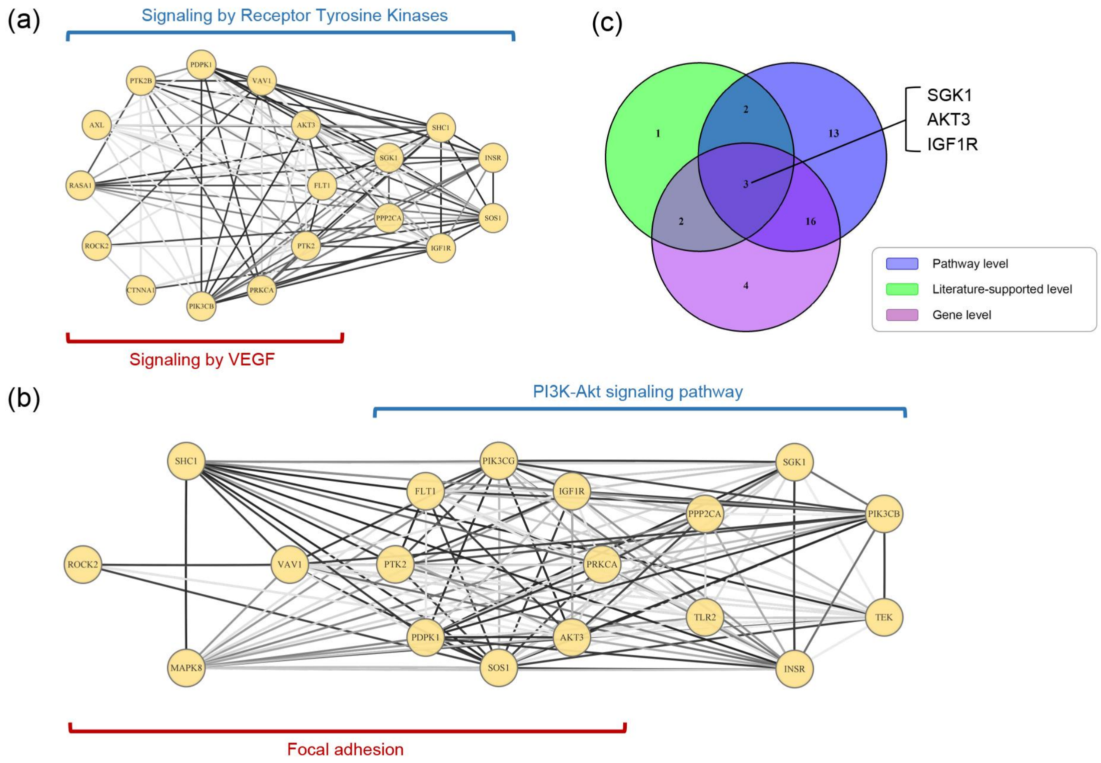

Figure S9. Six-level assessment of UCEC. a) Subgraph of candidates of pathway level (KEGG). b) Subgraph of candidates of pathway level (Reactome). c) Venn diagram of three level.

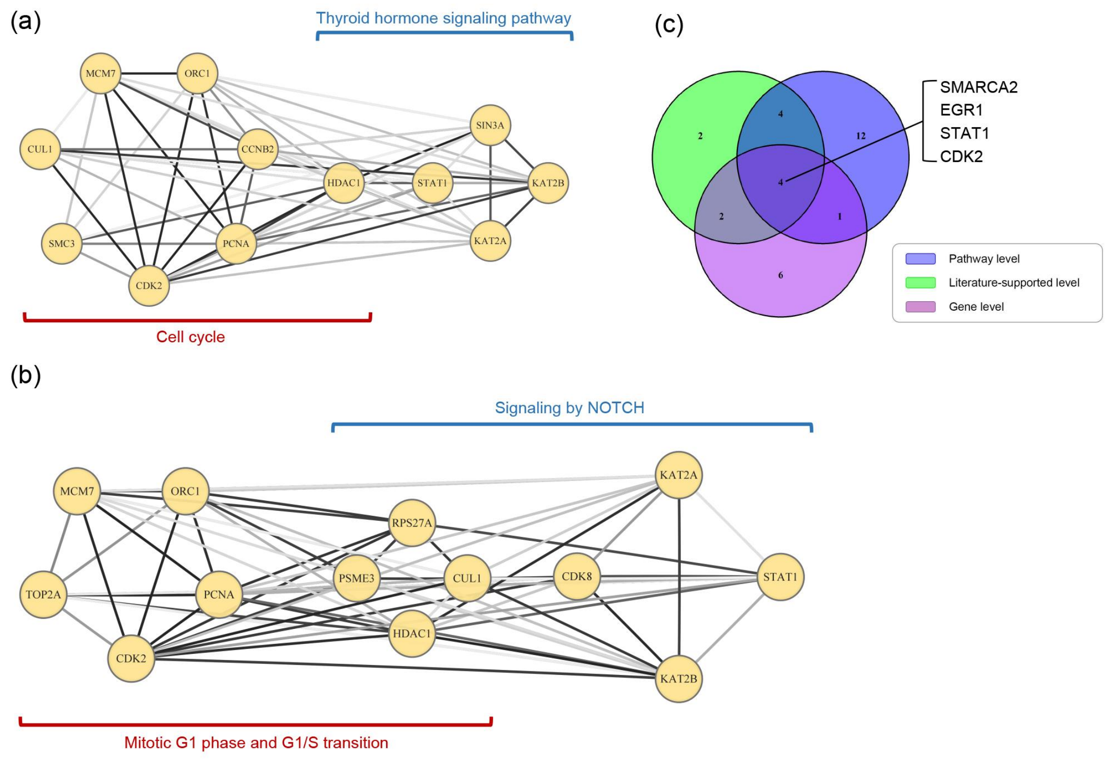

Figure S10. Six-level assessment of KIRC. a) Subgraph of candidates of pathway level (KEGG). b) Subgraph of candidates of pathway level (Reactome). c) Venn diagram of three levels.

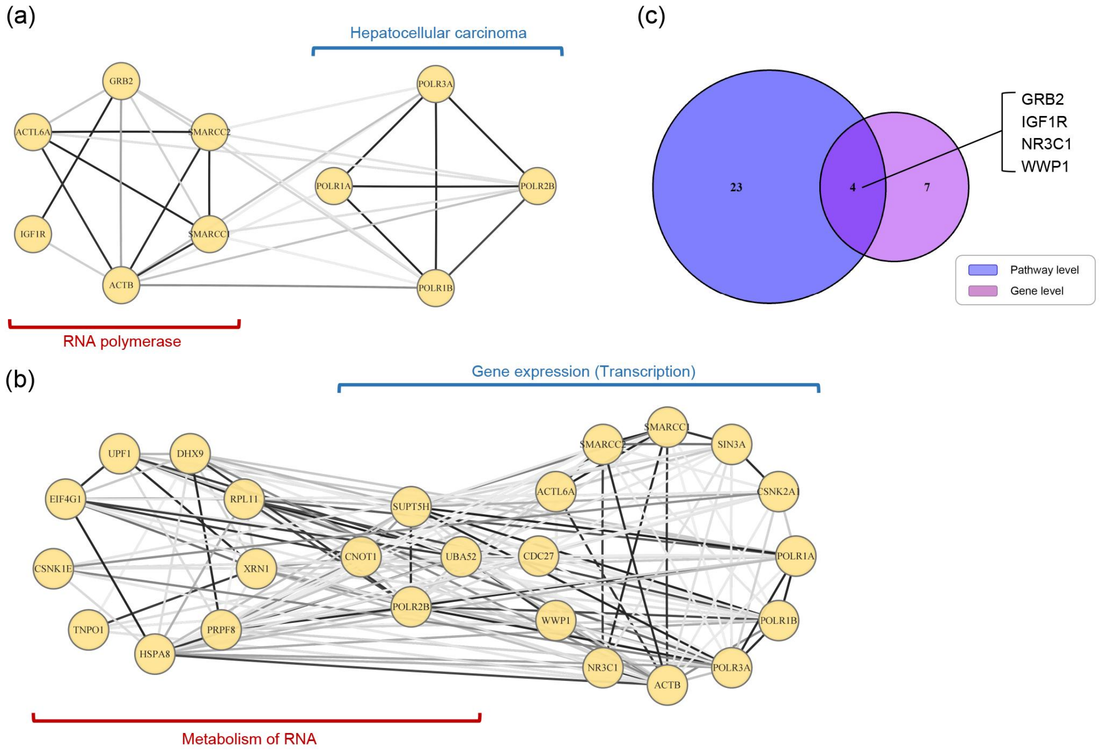

Figure S11. Six-level assessment of KIRP. a) Subgraph of candidates of pathway-related level (KEGG). b) Subgraph of candidates of pathway-related level (Reactome). c) Venn diagram of two levels.

Supplementary Tables

Table S1 Summary of the ten somatic non-silent mutation and tumor/normal expression datasets of each cancer type from TCGA.

Cancer type (TCGA)	Cancer type	Mutation size	Tumor size	Normal size
BRCA	breast invasive carcinoma	771	1091	113
PRAD	prostate adenocarcinoma	499	499	51
LUAD	lung adenocarcinoma	543	536	58
LUSC	lung squamous cell carcinoma	178	501	48
KIRC	kidney clear cell carcinoma	417	540	72
KIRP	kidney renal papillary cell carcinoma	282	539	71
HNSC	head & neck squamous cell carcinoma	306	501	43
COADREAD	colon & rectum adenocarcinoma	224	477	40
UCEC	uterine corpus endometrioid carcinoma	248	552	34
BLCA	bladder urothelial carcinoma	396	411	18

Table S2 List of genes for the four BC-related diseases against GAD.

Database	Disease type	Genes	Counts	Persentage(%)
GAD	breast cancer	ABCB1, TOP2A, ERCC6, RAD51, APEX1, CDC14A, CCNB1, CDK2, IGF1R, INSR, PIK3CB, PIK3CG, PRKDC, TP53BP1	14	27.45%
	plasma HDL cholesterol (HDL-C) levels	SMARCA2, IGF1R, INSR, MAPK3, NR3C1, SUMO1, SLC2A4	7	13.73%
	diabetes, type 2	CDK5, IGF1R, INSR, NR3C1, PIK3CB, SGK1, SLC2A4	7	13.73%
	obesity	IGF1R, INSR, NR3C1, SLC2A4	4	7.84%

Table S3 List of genes for the four LUAD-related diseases against GAD.

Database	Disease type	Genes	Counts	Persentage(%)
GAD	lung cancer	ABCB1, BARD1, ERCC6, CASP3, CDC25C, CHEK1, CCNA2, IGF1R, IRS1, PLK1, PRKDC, STAT1, SUMO1, TP53BP1	14	23.33%
	chronic obstructive pulmonary disease	ABCB1, BARD1, ERCC6, CASP3, CDC25C, CHEK1, CCNA2, IGF1R, IRS1, PLK1, STAT1, TLR4	12	20.00%

Table S4. Six levels of the 71 driver candidates of PRAD.

No.	Level	Driver candidates	Count	Percentage
1	Cancer-type level	ABCB1, CAT, CDKN1A, ERCC6, IGF1R, IRS1, MED1, MSH3, NR3C1, PARP1, PRKDC, PTGS2, RYR1, USP7	14	19.72%
2	Literature-supported level	BIRC5, BMX, CDC25A, CDC25B, CDKN1A, EEF2, EGR1, GSK3B, KAT5, MSH3, PIK3CG, PKD1, PRMT1, RPL11, SP1, SRCAP, YBX1, YWHAZ	18	25.35%
3	Pathway level	ABCB1, ANK1, BARD1, CASP1, CAT, CCNG1, CDC25A, CDC25B, CDC27, CDKN1A, CEBPB, CHD3, CSNK2A1, CSNK2A2, EEF2, EGR1, EPHA3, ERCC6, GLI3, GSK3B, HDAC2, HSPA5, HSPA8, IGF1R, IRS1, KAT5, MED1, MSH3, NR3C1, PARP1, PCNA, PIK3CB, PIK3CG, PPP2CA, PRKCA, PRKDC, PRMT1, PTGS2, PTK2, RPA1, RPL11, RYR1, RYR2, SIRT1, SP1, SP3, STK4, SYNE1, TAF1, TLE1, TNFRSF10B, TOP2A, TOP2B, TP53BP1, TP53BP2, UBE2I, USP7, YWHAZ	58	81.69%
4	Non-cancer disease level	▲	▲	▲
5	Gene level	BARD1, BMX, CDKN1A, CHD3, EGR1, EPHA3, GLI3, IGF1R, KAT5, NEB, NR3C1, PIK3CB, PPP2C, PRKCA, PRKDC, PTGS2, PTK2, SP1, SP3, SRCAP, TAF1, TP53BP2, YWHAZ	23	32.39%
6	Validation-required level	GNL3, HSPA4, HSPA9, CAD, TTN, ING1, MKI67	7	9.86%

Table S5 18 driver candidates of "Literature-supported level" of prostate cancer.

Gene	NCBI Entrez ID	Rank (HumanNet)	Rank (STRINGv10)	Function	Type
CDKN1A	1026	81	135	Analysis of CDKN1A genotypes may prove useful in determining which patients are at risk for developing advanced prostate carcinoma and therefore would gain the most from aggressive screening, prophylaxis, and/or treatment [7].	B
MSH3	4437	233	108	Related reports have experimentally shown that MSH3 polymorphisms may be a risk factor for prostate cancer [47].	E
EEF2	1938	26	113	Study indicates that expression of eEF2 protein is a potential biomarker for evaluating prostate cancer [48].	B
PRMT1	3276	28	235	Recent findings suggest that PRMT1 is a key regulator of AR output and provide a preclinical framework for the co-targeting of AR and PRMT1 in advanced prostate cancer [49].	E&B
SP1	6667	29	89	Studies have shown that Sp1 regulates important genes like androgen receptor, TGF-β, c-Met, fatty acid synthase, matrix metalloprotein (MT1-MMP), PSA, and α-integrin in prostate cancer [50].	O
CDC25A	993	46	111	It was shown that CDC25A is upregulated in human prostate cancer and interacts with androgen receptor and represses its transcriptional activity in human prostate cancer cells [51].	E&B
YWHAZ	7534	50	165	YWHAZ is an androgen-responsive gene that activates proliferation, cell survival, and androgen receptor transcriptional activity. Recent studies have shown that YWHAZ plays an independent role as a strong predictor of aggression in prostate cancer [52, 53].	E&B
EGR1	1958	62	72	Studies have shown that EGR1 regulates angiogenic and osteoclastogenic factors that inform the underlying signaling networks of autonomous and microenvironmental mechanisms that influence cancer metastasis and have a direct impact on prostate cancer metastasis [54].	B
GSK3B	2932	74	99	GSK3B, one of two isoforms of Glycogen synthase kinase (GSK-3), is associated with high expression of the androgen receptor [55].	E
SRCAP	10847	75	247	A report showed that SRCAP is expressed in normal prostate epithelium and prostate cancer cells and is associated with androgen receptor in the nucleus. Furthermore, inhibition of SRCAP expression significantly inhibited the growth of androgen dependent prostate cancer cells [56].	E
PIK3CG	5294	93	94	Recent reports suggest that PIK3CG plays an important role in the progression and metastasis of prostate cancer and may be a new therapeutic target for metastatic castration-resistant prostate cancer [57].	E
CDC25B	994	98	133	CDC25B is upregulated in human prostate cancer and its interaction with androgen receptor may contribute to prostate cancer development [58].	E
KAT5	10524	109	44	Recent studies suggested that elevation of androgen receptor expression and androgen receptor signaling in prostate tumors promotes prostate cancer metastasis by induction of epithelial-mesenchymal transition and reduction of KAT5 [59].	E
BMX	660	148	206	Some findings indicate that BMX contributes to activation of tyrosine kinase signaling in vivo in prostate cancer, with subsequent increases in MAPK and PI3K pathway activation [60, 61].	E
YBX1	4904	150	249	Recent studies have shown that CASC11 promotes the development and progression of prostate cancer by interacting with YBX1 and inhibiting p53 signaling [62].	E&B
RPL11	6135	154	42	RPL11 encodes a ribosomal protein and putative cancer suppressor upstream of the MDM2–TP53 pathway, and the enrichment of missense mutations in RPL11 suggests its oncogenic role in prostate cancer [63].	E&B
PKD1	5310	161	44	Osteoblastic PKD1 contributes to the prostate cancer cells dormancy via GAS6-circadian clock signaling [64].	E
BIRC5	332	196	48	Recent data obtained on a very large number of prostate cancer samples demonstrate that BIRC5 mRNA increased in prostate cancer and prostate cancer metastases compared to tissues from healthy donors or from adjacent normal prostate tissues combined [65].	E&B

Table S6. Biological pathways associated with prostate cancer.

Database	Pathway ID	Pathway	Genes	Count	Function	FDR
KEGG	hsa05206	MicroRNAs in cancer	SIRT1, CDC25B, PIK3CB, CDC25A, IRS1, CCNG1, UBE2I, PTGS2, CDKN1A, PRKCA, HDAC2, ABCB1	12	The abnormal expressions of miRNAs (MicroRNA) are now considered valuable biomarkers for diagnosis, prognosis and classification of PCa [66].	3.81E-10
	hsa04110	Cell cycle	CDC25B, CDC25A, PRKDC, GSK3B, PCNA, YWHAZ, CDKN1A, HDAC2, CDC27	9	Numerous proteins involved in cell cycle regulation are often mutated in PCa,Cell cycle proteins commonly mutated during PCa progression include the negative cell cycle regulators Rb , p14ARF , p16INK4a , p53 , and p27KIP1 [67].	1.53E-07
	hsa04068	FoxO signaling pathway	SIRT1, CAT, IGF1R, PIK3CB, IRS1, USP7, STK4, CDKN1A, PRMT1	9	Both FoxO1 and FoxO3 are deleted in 15% to 20% prostate cancer, indicating their tumor suppressive function in prostate cancer [68].	1.63E-07
	hsa04151	PI3K-Akt signaling pathway	IGF1R, PIK3CB, IRS1, GSK3B, PTK2, PIK3CG, YWHAZ, CDKN1A, PRKCA, PPP2CA	10	We found that activation of the PI3K/Akt/mTOR pathway proteins are associated with prostate cancer progression [69].	3.44E-05
	hsa05020	Prion disease	CSNK2A1, EGR1, CSNK2A2, PIK3CB, HSPA5, GSK3B, RYR1, RYR2, HSPA8	9	Prion protein may have a positive relationship with the malignant degree, aggressiveness, or progression of tumor and hold the potential to be one of the referenced markers of prostate and breast cancers [70].	3.44E-05
	hsa04921	Oxytocin signaling pathway	EEF2, PIK3CG, RYR1, RYR2, PTGS2, CDKN1A, PRKCA	7	Oxytocin may also be one of the key factors involved in the regulation of metabolic disorder-induced PCa [71].	6.36E-05
	hsa04152	AMPK signaling pathway	SIRT1, IGF1R, PIK3CB, IRS1, EEF2, PPP2CA	6	Additional preclinical studies using pharmacological, molecular, and genetic approaches have now identified an oncogenic role for AMPK in prostate cancer [72].	2.10E-04
	hsa04012	ErbB signaling pathway	PIK3CB, GSK3B, PTK2, CDKN1A, PRKCA	5	In addition, we show evidence that the ErbB/PI3K/Akt/ NF-κB signaling pathway is involved in prostate cancer progression and that a multiple marker approach identifying active signaling pathways may be prognostically more relevant than single markers [73].	4.70E-04
	hsa04064	NF-kappa B signaling pathway	CSNK2A1, CSNK2A2, UBE2I, PARP1, PTGS2	5	These results demonstrate that NF-κB/p65 is frequently activated in human prostate adenocarcinoma and expression may be related to progression [74].	8.60E-04
Reactome	HSA-212436	Generic Transcription Pathway	SIRT1, CSNK2A1, NR3C1, CAT, RPA1, BARD1, CSNK2A2, TNFRSF10B, MED1, CEBPB, GSK3B, SP1, KAT5, TP53BP2, USP7, CCNG1, UBE2I, PARP1, PCNA, CHD3, GLI3, YWHAZ, CDKN1A, PRMT1, TAF1, PPP2CA, HDAC2, CASP1, CDC27	29	Discussed before.	9.87E-14
	HSA-74160	Gene expression (Transcription)	SIRT1, CSNK2A1, NR3C1, CAT, RPA1, BARD1, CSNK2A2, TNFRSF10B, MED1, CEBPB, GSK3B, SP1, KAT5, TP53BP2, USP7, CCNG1, UBE2I, ERCC6, PARP1, PCNA, CHD3, GLI3, YWHAZ, CDKN1A, PRMT1, TAF1, PPP2CA, HDAC2, CASP1, CDC27	30		5.97E-13
	HSA-3700989	Transcriptional Regulation by TP53	CSNK2A1, RPA1, BARD1, CSNK2A2, TNFRSF10B, KAT5, TP53BP2, USP7, CCNG1, PCNA, CHD3, YWHAZ, CDKN1A, PRMT1, TAF1, PPP2CA, HDAC2, CASP1	18		6.73E-13
	HSA-5633007	Regulation of TP53 Activity	CSNK2A1, RPA1, BARD1, CSNK2A2, KAT5, TP53BP2, USP7, CCNG1, CHD3, TAF1, PPP2CA, HDAC2	12		4.59E-10
	HSA-1640170	Cell Cycle	CSNK2A1, CDC25B, RPA1, BARD1, CSNK2A2, CDC25A, GSK3B, KAT5, UBE2I, SYNE1, PCNA, TP53BP1, YWHAZ, CDKN1A, PRKCA, TOP2A, PPP2CA, CDC27	18		5.75E-09
	HSA-73894	DNA Repair	RPA1, BARD1, MSH3, PRKDC, KAT5, USP7, UBE2I, ERCC6, PARP1, PCNA, TP53BP1	11		3.02E-06
	HSA-69278	Cell Cycle, Mitotic	CSNK2A1, CDC25B, RPA1, CSNK2A2, CDC25A, GSK3B, UBE2I, PCNA, CDKN1A, PRKCA, TOP2A, PPP2CA, CDC27	13		7.16E-06
	HSA-5693532	DNA Double-Strand Break Repair	RPA1, BARD1, PRKDC, KAT5, UBE2I, PARP1, PCNA, TP53BP1	8		1.18E-05
	HSA-9614085	FOXO-mediated transcription	SIRT1, NR3C1, CAT, YWHAZ, CDKN1A, HDAC2	6	Whereas forced expression of FoxO1 induces apoptosis in certain cancer cell types, such as prostate cancer cells [75].	2.59E-05
	HSA-5693538	Homology Directed Repair	RPA1, BARD1, KAT5, UBE2I, PARP1, PCNA, TP53BP1	7	Germ-line and somatic mutations in genes that promote homology-directed repair (HDR), especially BRCA1 and BRCA2, are frequently observed in several cancers, in particular, breast and ovary but also prostate and other cancers [76].	3.89E-05
	HSA-597592	Post-translational protein modification	NR3C1, RPA1, BARD1, ANK1, CDC25A, EEF2, SP3, PRKDC, USP7, UBE2I, PARP1, PCNA, CHD3, TP53BP1, TOP2B, TOP2A, HDAC2, HSPA8	18	Many enzymes involved in PTMs are deregulated in prostate cancer and this directs prostate cancer cell behaviors. Since their driving role in prostate cancer, PTMs are widely explored in attempts to advance prostate cancer therapy [77].	1.60E-04
	HSA-5696398	Nucleotide Excision Repair	RPA1, USP7, UBE2I, ERCC6, PARP1, PCNA	6	Deficient nucleotide excision repair capacity enhances human prostate cancer risk [78].	3.00E-04
	HSA-9675108	Nervous system development	CSNK2A1, CSNK2A2, ANK1, PIK3CB, GSK3B, EPHA3, PTK2, RPL11, PRKCA, HDAC2, HSPA8	11	Autonomic nerve development contributes to prostate cancer progression [79].	5.50E-04
	HSA-6807070	PTEN Regulation	CSNK2A1, EGR1, CSNK2A2, USP7, CHD3, HDAC2	6	Genomic aberrations of the PTEN tumour suppressor gene are among the most common in prostate cancer [80].	8.80E-04
	HSA-195721	Signaling by WNT	CSNK2A1, CSNK2A2, GSK3B, KAT5, TLE1, YWHAZ, PRKCA, PPP2CA	8	Inhibition of WNT signaling has the potential to reduce the self-renewal of prostate cancer cells with stem cell characteristics and improve the therapeutic outcome [81].	9.00E-04

Table S7. 23 driver candidates of "Gene level" of PRAD.

Gene	NCBI Entrez ID	CGC [Gene (Sequence similarity/E_value)]
BARD1	580	BCOR (40.299/8.86E-18)
BMX	660	BTK (48.834/0), ITK (45.104/0), SRC (40.16/2.73E-90), PDGFRA (44.048/1.50E-38), PDGFRB (46.358/4.57E-36), KIT (44.079/2.42E-34), KDR (42.105/3.65E-34), FLT4 (42.763/1.32E-33), FLT3 (44.156/5.07E-33)
CDKN1A	1026	CDKN1B (42.857/9.18E-16)
CHD3	1107	CHD4 (71.161/0), TRIM24 (49.091/8.86E-12), KDM5C (56.522/4.58E-11), TRIM33 (40.678/4.34E-10), KDM5A (52.174/2.22E-10), NSD3 (54.348/3.76E-10), NSD2 (47.17/1.04E-06)
EGR1	1958	WT1 (61.053/1.47E-32), KLF6 (59.016/2.34E-19), BCL6 (41.379/5.24E-09), BCL5 (41.379/5.24E-09), ZNF331 (44.048/3.82E-17), PRDM16 (45.122/8.63E-18), ZNF521 (40.777/1.02E-17), MECOM (45.122/1.23E-17), CTCF (42.857/3.31E-13), SALL4 (49.057/7.86E-11)
EPHA3	2042	SRC (44.565/7.13E-66), LCK (41.958/7.56E-65), FES (40.672/5.47E-60), KIT (41.935/3.87E-34)
GLI3	2737	WT1 (40.164/2.43E-21), ZNF331 (40.94/3.47E-19), BCL6 (43.956/5.42E-11), BCL5 (43.956/5.42E-11), KLF6 (40.789/4.53E-14), BCL11B (47.059/7.70E-07), BCL11A (47.059/1.07E-06), SALL4 (41.176/1.19E-05)
IGF1R	3480	ROS1 (40.302/1.30E-73), NTRK1 (41.892/2.27E-71), FGFR3 (40.21/5.94E-65), FGFR2 (40.351/1.35E-64), DDR2 (40.127/1.01E-58), ABL2 (41.606/1.51E-57), PDGFRA (40.462/8.93E-36), PDGFRB (41.176/2.73E-34)
KAT5	10524	KAT6A (53.846/3.42E-94), KAT6B (51.471/7.79E-87)
NEB	4703	LASP1 (72.464/6.43E-27), ABI1 (43.396/8.24E-06)
NR3C1	2908	AR (51.752/5.44E-125)
PIK3CB	5291	PIK3CA (40.796/0)
PPP2CA	5515	PPP6C (58.14/6.69E-131)
PRKCA	5578	PRKACA (100/0), AKT2 (47.432/1.54E-104), AKT1 (47.436/1.49E-100)
PRKDC	5591	PIK3CA (42.424/2.61E-06)
PTGS2	5743	NOTCH2 (46.341/3.27E-06)
PTK2	5747	ABL1 (40.214/1.47E-63), ABL2 (40.58/1.32E-61), SRC (41.288/7.18E-59), ERBB2 (40.074/1.74E-55), FLT4 (40.331/8.14E-40), KDR (41.714/1.84E-38), FLT3 (40.909/7.65E-35), PDGFRB (40.385/6.40E-31)
SP1	6667	KLF6 (54.639/1.45E-31), KLF4 (52.525/1.64E-30), WT1 (47.423/2.61E-26), PRDM1 (42.222/2.96E-16), MECOM (46.296/3.81E-09), PRDM16 (46.296/2.43E-09), PATZ1 (43.333/3.33E-11), ZNF331 (44.737/1.28E-10), IKZF1 (46.97/6.42E-12), SALL4 (50/1.10E-10), CTCF (42.466/1.40E-10), BCL11B (42/3.03E-07), BCL11A (42/6.09E-07)
SP3	6670	KLF6 (53.608/6.60E-31), KLF4 (52.632/2.03E-29), WT1 (44.737/1.71E-27), PRDM1 (43.333/9.13E-17), PATZ1 (41.284/1.31E-16), MECOM (42.623/1.29E-09), ZNF521 (42.5/3.86E-15), ZBTB16 (42.105/4.40E-15), PRDM16 (42.623/1.03E-09), PLAG1 (41.25/3.31E-14), ZNF331 (45/6.61E-13), IKZF1 (43.75/2.43E-12), SALL4 (50/1.31E-08), BCL11B (42.308/7.90E-08), BCL11A (42.105/1.29E-07)
SRCAP	10847	SMARCA4 (47.097/1.54E-40), CHD4 (42.714/1.03E-41)
TAF1	6872	TRIM24 (40.625/4.08E-08), TRIM33 (40.678/1.37E-06)
TP53BP2	7159	SH3GL1 (40.426/2.18E-06)
YWHAZ	7534	YWHAE (69.492/6.94E-119)

Table S8. Six levels of the 68 driver candidates of LUSC.

No.	Level	Driver candidates	Count	Percentage
1	Cancer-type level	ABCB1, BARD1, CDK1, CDK5, CDKN1A, CHEK1, ERCC6, IGF1R, INSR, IRS1, PARP1, PLK1, PRKDC, RAD51, TP53BP1	15	22.06%
2	Literature-supported level	BARD1, CDK1, CDK5, CHEK, DMD, EIF4G1, KAT2B, PARP1, PTTG1, RAD51, RYR2, SIN3A, SMARCA2, USP7	14	20.59%
3	Pathway level	ABCB1, ANK2, BARD1, BCL2L1, CDK1, CDK5, CDKN1A, CHEK1, COPS5, CSNK1D, CSNK2A1, EEF2, EGR1, ERCC6, FLT1, FN1, GLI3, GNB2, HDAC1, HSPA2, HSPA5, HSPA8, IGF1R, INSR, IRS1, KAT2B, MAPK3, MAPK8, MDC1, MED1, PARP1, PIK3CG, PLK1, POLA1, PRKCA, PRKDC, PTK2, PTTG1, RBL1, RYR1, RYR2, SIN3A, SMARCA2, SMARCC1, SP1, TAF1, TAF2, TOP2A, TOP2B, TP53BP1, TTN, UBE2A, USP7, VCAN, WEE1	55	80.88%
4	Non-cancer disease level	ABCB1, BARD1, CDK5, CHEK1, ERCC6, IGF1R, INSR, IRS1, PARP1, PLK1, RAD51	11	16.18%
5	Gene level	BARD1, BCL2L1, CDK1, CDK5, CDKN1A, E4F1, EGR1, FLT1, GLI3, IGF1R, INSR, MAPK3, MAPK8, NEB, PRKCA, PRKDC, PTK2, SMARCA2, SP1, TAF1, VCAN	21	30.88%
6	Validation-required level	CAPN3, GTPBP4, NTN1, ACTA1, NDN, CSNK1A1, SYNCRIP, HSPA9	8	11.76%

Table S9 14 driver candidates of “Literature-supported level” of LUSC.

Gene	NCBI Entrez ID	Rank (HumanNet)	Rank (STRINGv10)	Function	Type
CHEK1	1111	10	48	Studies have shown that CHEK1 expression is increased in NSCLC compared to adjacent normal tissues and that high CHEK1 expression in NSCLC is associated with poor OS. These results suggest that CHEK1 may play a potential oncogenic role in NSCLC [82, 83].	E&B
CDK1	983	27	79	CDK1 expression was higher in squamous cell lung cancer tissues than in normal lung samples. In addition, survival analysis showed that upregulation of CDK1 was associated with lower overall survival, lower first progression and post-progression survival in lung cancer patients [84].	B
PARP1	142	42	145	The inhibition of PARP1 can provide a potential targeted therapy and prevention of inflammation-associated lung carcinogenesis [12].	E
RAD51	5888	64	211	The decreased survival of patients with NSCLC with high levels of Rad51 expression may be related to the enhanced propensity of tumor cells to survive, resist apoptosis and chemo/radio resistance [85].	B
BARD1	580	108	112	BARD1 isoforms might be involved in tumor initiation and invasive progression and might represent a novel prognostic marker for NSCLC [86].	E
CDK5	1020	136	214	Recent reports suggest that high CDK5 expression is associated with shorter overall survival (OS) in lung cancer and promotes proliferation and metastasis of lung cancer cells [13].	B
KAT2B	8850	20	50	KAT2B was synergistic with multiple immune cells infiltration and immune checkpoints in NSCLC, which will promote the progression of new immunotherapeutic strategies [87].	B
USP7	7874	30	96	Data strongly suggested that the overexpression of USP7 might promote cell proliferation by deubiquitinating Ki-67 protein, thereby maintaining its high levels in the non-small cell lung cancer [88].	E&B
RYR2	6262	36	40	Survival analysis revealed that RYR2 mutations may be protective against NSCLC and that NSCLC patients with RYR2 mutations may have a better prognosis by downregulating DKK1 and upregulating GS1-115G20.1 [89].	B
SIN3A	25942	41	64	It was shown that the chromosomal region near D15S984 at 15q23, where the SIN3A gene is located, exhibited a higher incidence of LOH in NSCLC tumors (69%). SIN3A has an important role in lung carcinogenesis through allelic deletions and subsequent epigenetic alterations [90].	E
PTTG1	9232	43	212	PTTG1 expression levels correlate with clinicopathological features and patient survival in NSCLC and promote NSCLC cell invasion under the regulation of miR-186. PTTG1 may be novel therapeutic targets for NSCLC invasion intervention [91].	E
DMD	1756	128	18	DMD is differentially expressed in lung cancer subtypes curated in the Cancer Genome Atlas database, further revealing that DMD expression and mutations may have an impact on immune function [92].	B
SMARCA2	6595	164	249	SMARCA2 was found to be a tumor suppressor gene that significantly inhibited the viability of lung cancer cells, and its expression was downregulated in lung cancer. Its inactivation was significantly associated with poor survival of lung cancer patients [93].	E&B
EIF4G1	1981	206	237	A recent study has demonstrated for the first time the immunomodulatory function, clinical relevance and therapeutic potential of the EIF4G1 network in NSCLC, which may be a promising new target for improving lung cancer treatment [94].	B

Table S10. Biological pathways associated with LUSC.

Database	Pathway ID	Pathway	Genes	Count	Function	FDR
KEGG	hsa04110	Cell cycle	CDK1, CDKN1A, CHEK1, HDAC1, PLK1, PRKDC, PTTG1, RBL1, WEE1	9	Discussed before.	2.05E-07
	hsa04151	PI3K-Akt signaling pathway	BCL2L1, CDKN1A, FLT1, FN1, GNB2, IGF1R, INSR, IRS1, MAPK3, PIK3CG, PRKCA, PTK2	12		6.23E-07
	hsa05203	Viral carcinogenesis	CDK1, CDKN1A, CHEK1, HDAC1, KAT2B, MAPK3, RBL1, USP7	8	Viral infections such as human papillomavirus (HPV), HIV and Epstein-Barr virus infections are thought to be linked to lung cancer [95].	1.12E-05
	hsa04014	Ras signaling pathway	BCL2L1, FLT1, GNB2, IGF1R, INSR, MAPK3, MAPK8, PRKCA	8	Discussed before.	3.73E-05
	hsa04066	HIF-1 signaling pathway	CDKN1A, FLT1, IGF1R, INSR, MAPK3, PRKCA	6	CD39/CD73 upregulation on myeloid-derived suppressor cells via TGF-β-mTOR-HIF-1 signaling in patients with non-small cell lung cancer [96].	5.25E-05
	hsa04919	Thyroid hormone signaling pathway	HDAC1, KAT2B, MAPK3, MED1, PRKCA, SIN3A	6	Hyperthyroidism is correlated with cancer prevalence in various tumor types, including breast, thyroid, lung, brain, liver and colorectal cancer [97].	9.25E-05
	hsa01524	Platinum drug resistance	BCL2L1, CDKN1A, MAPK3, TOP2A, TOP2B	5	Platinum is one of the most frequently used (neo) adjuvant treatment for lung cancer. However, platinum resistance limits its clinical application. Recently, there are multiple newer, more effective and less toxic agents to treat platinum resistance in lung cancer [98].	1.10E-04
	hsa04510	Focal adhesion	FLT1, FN1, IGF1R, MAPK3, MAPK8, PRKCA, PTK2	7		1.20E-04
	hsa04010	MAPK signaling pathway	FLT1, HSPA2, HSPA8, IGF1R, INSR, MAPK3, MAPK8, PRKCA	8	Discussed before.	1.30E-04
	hsa04012	ErbB signaling pathway	CDKN1A, MAPK3, MAPK8, PRKCA, PTK2	5		1.90E-04
	hsa04713	Circadian entrainment	GNB2, MAPK3, PRKCA, RYR1, RYR2	5	Chronic circadian disruption promotes tumor growth by altering the circadian rhythms of NK cell function [99].	2.90E-04
	hsa05206	MicroRNAs in cancer	ABCB1, CDKN1A, HDAC1, IRS1, MAPK3, PRKCA	6	Discussed before.	3.10E-04
Reactome	HSA-1640170	Cell Cycle	BARD1, CDK1, CDKN1A, CHEK1, CSNK1D, CSNK2A1, HDAC1, HSPA2, MAPK3, MDC1, PLK1, POLA1, PRKCA, PTTG1, RBL1, TOP2A, TP53BP1, WEE1	18	Discussed before.	
	HSA-212436	Generic Transcription Pathway	BARD1, CDK1, CDK5, CDKN1A, CHEK1, CSNK2A1, GLI3, HDAC1, KAT2B, MAPK3, MDC1, MED1, PARP1, RBL1, SIN3A, SMARCA2, SMARCC1, SP1, TAF1, TAF2, USP7	21		
	HSA-74160	Gene expression (Transcription)	BARD1, CDK1, CDK5, CDKN1A, CHEK1, CSNK2A1, ERCC6, GLI3, HDAC1, KAT2B, MAPK3, MDC1, MED1, PARP1, RBL1, SIN3A, SMARCA2, SMARCC1, SP1, TAF1, TAF2, USP7	22		
	HSA-3700989	Transcriptional Regulation by TP53	BARD1, CDK1, CDK5, CDKN1A, CHEK1, CSNK2A1, HDAC1, MDC1, RBL1, TAF1, TAF2, USP7	12		
	HSA-597592	Post-translational protein modification	ANK2, BARD1, CDK1, COPS5, CSNK1D, EEF2, FN1, HDAC1, HSPA8, KAT2B, MDC1, PARP1, PRKDC, SIN3A, TOP2A, TOP2B, TP53BP1, UBE2A, USP7, VCAN	20	SUMOylation has attracted increasing attention as a widely used post-translational protein modification. The disorder of SUMOylation can lead to the development of certain diseases and tumors. SUMO can thus be used as a potential therapeutic target for cancer [100].	7.39E-06
	HSA-3108232	SUMO E3 ligases SUMOylate target proteins	HDAC1, MDC1, PARP1, SIN3A, TOP2A, TOP2B, TP53BP1	7		2.30E-04
	HSA-73894	DNA Repair	BARD1, CHEK1, COPS5, ERCC6, MAPK8, MDC1, PARP1, PRKDC, TP53BP1, USP7	10	Discussed before.	2.78E-05
	HSA-5693532	DNA Double-Strand Break Repair	BARD1, CHEK1, MAPK8, MDC1, PARP1, PRKDC, TP53BP1	7	Suppression of a DNA double-strand break repair gene, Ku70, increases radio- and chemosensitivity in a human lung carcinoma cell line [101].	1.50E-04
	HSA-6804756	Regulation of TP53 Activity through Phosphorylation	BARD1, CDK5, CHEK1, CSNK2A1, TAF1, TAF2	6	Rapamycin prevents strong phosphorylation of p53 on serine 46 and attenuates activation of the p53 pathway in A549 lung cancer cells exposed to actinomycin D [102].	1.50E-04
	HSA-9006934	Signaling by Receptor Tyrosine Kinases	CDK5, CHEK1, EGR1, FLT1, FN1, IGF1R, INSR, IRS1, MAPK3, PRKCA, PTK2	11	Discussed before.	1.50E-04
	HSA-76002	Platelet activation, signaling and aggregation	FN1, GNB2, HSPA5, MAPK3, PIK3CG, PRKCA, PTK2, TTN	8	Platelets play a major role in the metastatic dissemination of tumor cells in vivo. Study demonstrates significant downregulation of platelet gene expression in metastatic lung cancer [103].	3.60E-04

Table S11. 23 driver candidates of "Gene level" of LUSC.

Gene	NCBI Entrez ID	CGC [Gene (Sequence similarity/E_value)]
BARD1	580	BCOR (40.299/8.86E-18)
CHD3	1107	CHD4 (71.161/0), TRIM24 (49.091/8.86E-12), KDM5C (56.522/4.58E-11), TRIM33 (40.678/4.34E-10), KDM5A (52.174/2.22E-10), NSD3 (54.348/3.76E-10), NSD2 (47.17/1.04E-06)
EGR1	1958	WT1 (61.053/1.47E-32), KLF6 (59.016/2.34E-19), BCL6 (41.379/5.24E-09), BCL5 (41.379/5.24E-09), ZNF331 (44.048/3.82E-17), PRDM16 (45.122/8.63E-18), ZNF521 (40.777/1.02E-17), MECOM (45.122/1.23E-17), CTCF (42.857/3.31E-13), SALL4 (49.057/7.86E-11)
FLT1	2321	KDR (44.933/0), FLT4 (40.603/0), FLT3 (40.812/2.65E-104), FGFR4 (44.857/7.29E-88), RET (42.135/3.70E-78), MET (45.604/3.68E-45), PTK6 (47.647/1.77E-40), ABL2 (44.654/3.47E-39), ROS1 (43.506/1.65E-37), ALK (42.683/1.92E-37), ABL1 (40.719/8.67E-37), EGFR (41.975/2.94E-36), FES (41.718/4.07E-36), ERBB2 (42.405/7.47E-35), SYK (40.881/3.99E-31), JAK3 (42.529/1.62E-30)
GLI2	2736	WT1 (40.164/6.23E-22), ZNF331 (40.741/1.86E-14), BCL6 (43.956/4.04E-11), BCL5 (43.956/4.04E-11), BCL11B (45.283/2.68E-07), BCL11A (45.283/3.92E-07), SALL4 (40.741/3.17E-06)
GLI3	2737	WT1 (40.164/2.43E-21), ZNF331 (40.94/3.47E-19), BCL6 (43.956/5.42E-11), BCL5 (43.956/5.42E-11), KLF6 (40.789/4.53E-14), BCL11B (47.059/7.70E-07), BCL11A (47.059/1.07E-06), SALL4 (41.176/1.19E-05)
GRB2	2885	SH3GL1 (50/2.47E-13), ABI1 (42/2.30E-09), SRGAP3 (43.396/1.43E-07), ARHGAP26 (41.509/3.79E-07)
HOXA5	3202	HOXA9 (68.333/2.48E-23), MNX1 (52.778/1.36E-21), CDX2 (53.012/5.89E-21), HOXA11 (47.945/4.71E-19), HOXD11 (51.667/1.58E-18), HOXC11 (47.541/7.85E-18), HOXA13 (49.254/2.89E-15), HOXD13 (48.438/1.35E-13)
IGF1R	3480	ROS1 (40.302/1.30E-73), NTRK1 (41.892/2.27E-71), FGFR3 (40.21/5.94E-65), FGFR2 (40.351/1.35E-64), DDR2 (40.127/1.01E-58), ABL2 (41.606/1.51E-57), PDGFRA (40.462/8.93E-36), PDGFRB (41.176/2.73E-34)
LYN	4067	LCK (67.292/0), SRC (60.271/0), ABL1 (41.648/4.59E-113), ABL2 (41.203/3.66E-112), PDGFRA (41.071/9.31E-13), PDGFRB (48.322/1.99E-39), KIT (45.161/1.99E-38), FLT3 (42.391/2.54E-38), KDR (42.012/6.19E-35)
MAPK9	5601	MAPK1 (41.274/1.00E-84)
NEB	4703	LASP1 (72.464/6.43E-27), ABI1 (43.396/8.24E-06)
NR3C1	2908	AR (51.752/5.44E-125)
PPP3CA	5530	PPP6C (41.219/1.27E-67)
PRKCA	5578	PRKACA (100/0), AKT2 (47.432/1.54E-104), AKT1 (47.436/1.49E-100)
PRKCB	5579	PRKACA (79.552/0), AKT2 (45.584/3.35E-110), AKT1 (42.785/3.16E-105)
PRKDC	5591	PIK3CA (42.424/2.61E-06)
PTK2B	2185	ROS1 (42.697/2.24E-52), FLT4 (45.342/2.22E-38), KDR (43.195/2.11E-37), KIT (40.667/3.18E-32)
SMARCA2	6595	SMARCA4 (79.037/0), CHD4 (40.262/6.65E-109)
SP1	6667	KLF6 (54.639/1.45E-31), KLF4 (52.525/1.64E-30), WT1 (47.423/2.61E-26), PRDM1 (42.222/2.96E-16), MECOM (46.296/3.81E-09), PRDM16 (46.296/2.43E-09), PATZ1 (43.333/3.33E-11), ZNF331 (44.737/1.28E-10), IKZF1 (46.97/6.42E-12), SALL4 (50/1.10E-10), CTCF (42.466/1.40E-10), BCL11B (42/3.03E-07), BCL11A (42/6.09E-07)
STAT1	6772	STAT3 (52.901/0)
TAF1	6872	TRIM24 (40.625/4.08E-08), TRIM33 (40.678/1.37E-06)
VCAN	1462	NOTCH2 (52.381/3.46E-15), NOTCH1 (49.315/1.16E-15), FAT1 (48.571/3.81E-05)

Table S12. Six levels of the 65 driver candidates of HNSC.

No.	Level	Driver candidates	Count	Percentage
1	Cancer-type level	ABCB1, GLI2, IGF1R, PRKDC, PTGS2, RAD51	6	9.23%
2	Literature-supported level	ACTA1, EGR1, FLT1, FN1, HMGB1, IGF1R, PTGS2, PTK2, RUNX3, SIRT1, TP53BP1, YY1	12	18.46%
3	Pathway level	APP, ARRB1, BARD1, BAX, BCL2L1, CCNA2, CDC14A, CHD3, CSNK1E, CSNK2A1, ERCC6, FLT1, FN1, GLI2, GLI3, GTF2H1, HMGB1, HSPA8, IGF1R, JAG1, KAT5, MAPK10, MAPK8, MAPK9, MDC1, MED1, MEF2C, NOTCH3, NR3C1, PARP1, PRKDC, PTGS2, PTK2, RBL1, RPA1, RUNX3, SIN3A, SIRT1, SMARCA2, STAT1, TAF1, TAF2, TAF5, TOP2A, TOP2B, TP53BP1, TXN, TXNRD1, UBA52, USP7, YWHAZ, YY1	52	80.00%
4	Non-cancer disease level	▲	▲	▲
5	Gene level	BARD1, BCL2L1, CHD3, EGR1, FLT1, GLI2, GLI3, IGF1R, JAG1, KAT5, MAPK10, MAPK8, MAPK9, NCOA3, NEB, NOTCH3, NR3C1, PRKDC, PTGS2, PTK2, RUNX3, SMARCA2, STAT1, TAF1, YWHAZ, YY1	26	40.00%
6	Validation-required level	EIF4G1, PIK3CG, SYNCRIP, NCL, KRT5, TTN, DMD	7	10.77%

Table S13 12 driver candidates of "Literature-supported level" of HNSC.

Gene	NCBI Entrez ID	Rank (HumanNet)	Rank (STRINGv10)	Function	Type
TP53BP1	7158	19	32	Data suggest that TP53BP1 variants may have protective effects on SCCHN risk but such effects were confined to TP53 variant allele/haplotype carriers [104].	B
IGF1R	3480	82	40	Data from an experiment showed that IGF-1R was upregulated in HNSCC compared to paired benign oropharyngeal epithelial cells, with 36% of tumors having prominent IGF-1R signals on the plasma membrane and 92% on the cytoplasm [21].	B
HMGB1	3164	35	203	HMGB1 is overexpressed in tumor cells of HNSCC, and serum levels are significantly elevated. Further, HMGB1 is a chemoattractant for regulatory T cells (Treg) and promotes their suppressive function [105].	E
PTK2	5747	48	62	Proteomic analysis identified PTK2/FAK overexpression is a biomarker of radioresistance in locally advanced HNSCC, and PTK2/FAK inhibition radiosensitized HNSCC cells [106].	E&B
EGR1	1958	58	81	Recent reports found that combined inhibition of GDF15 and EGR1 in an HNC mouse xenograft model resulted in a significant reduction in tumor volume compared to inhibition of EGR1 or GDF15 alone, suggesting that the GDF15-EGR1 signaling axis may be a good target for HNC patients [107].	E
SIRT1	23411	74	232	Recent studies have identified Sirt6 and Sirt6-mediated inhibition of Sirt1 upregulates reactive oxygen species, which further leads to HNSCC cell death. The molecular role of Sirt6 and Sirt1 in tumorigenesis may contribute to the development of new strategies for the treatment of HNSCC [108].	E&B
ACTA1	58	91	214	Recent bioinformatics-based reports suggest that ACTA1 may play an important role in regulating the initiation and progression of Head and neck squamous cell carcinoma (HNSCC) and could be identified as a key biomarker for accurate diagnosis and prognosis of HNSCC [109].	B
YY1	7528	108	66	Recent reports suggest that combined high expression of YY1 and CP2 mRNA may have prognostic relevance in HNSCC, which warrants further investigation [110].	B
PTGS2	5743	172	240	Recent studies have identified EGFR and PTGS2 as key nodes of the gene regulatory network in head and neck cancer through an integrative multi-omics approach, and several DEGs associated with the immune phenotype are affected by EGFR inhibition in tumor cell lines [22].	B
FLT1	2321	193	54	Studies have shown that the receptor tyrosine kinase FLT1 was identified as an important driver of cell survival and radioresistance in HNSCC. Specifically, FLT1 kinase is selectively upregulated in tumor tissue and can regulate radioresistance and cancer cell survival [111].	E&B
FN1	2335	198	136	The findings suggest that FN1 should be explored as a potential novel biomarker for radioresistance in squamous cell carcinoma of the head and neck [112].	B
RUNX3	864	208	118	RUNX3 overexpression was frequently observed and was well correlated with malignant behaviors in head and neck cancer. Moreover, it has been revealed that RUNX3 overexpression promoted cell growth and inhibited apoptosis in head and neck cancer cells [113].	E

Table S14. Biological pathways associated with HNSC.

Database	Pathway ID	Pathway	Genes	Count	Function	FDR
KEGG	hsa04068	FoxO signaling pathway	CSNK1E, IGF1R, MAPK10, MAPK8, MAPK9, SIRT1, USP7	7	Gain-of-function mutant p53 promotes the oncogenic potential of head and neck squamous cell carcinoma cells by targeting the transcription factors FOXO3a and FOXM1 [114].	8.27E-06
	hsa05165	Human papillomavirus infection	BAX, CCNA2, FN1, JAG1, NOTCH3, PTGS2, PTK2, RBL1, STAT1	9	Human papillomavirus (HPV) is now recognized to play a role in the pathogenesis of a subset of head and neck squamous cell carcinomas (HNSCCs), particularly those that arise from the lingual and palatine tonsils within the oropharynx [115].	3.12E-05
	hsa04621	NOD-like receptor signaling pathway	BCL2L1, MAPK10, MAPK8, MAPK9, STAT1, TP53BP1, TXN	7	In head-and-neck cancers, the upregulated level of Nod-like receptor protein 3 (NLRP3) promotes tumor progression [116].	4.18E-05
	hsa04510	Focal adhesion	FLT1, FN1, IGF1R, MAPK10, MAPK8, MAPK9, PTK2	7	Focal adhesion kinase (FAK) overexpression is a frequent and early event in HNSCC that is maintained during tumor progression. Overexpression of FAK in these carcinomas is associated with the presence of lymph node metastasis [117].	9.01E-05
	hsa04340	Hedgehog signaling pathway	ARRB1, CSNK1E, GLI2, GLI3	4	Hedgehog signaling alters reliance on EGF receptor signaling and mediates anti-EGFR therapeutic resistance in head and neck cancer [118].	3.00E-04
	hsa04668	TNF signaling pathway	JAG1, MAPK10, MAPK8, MAPK9, PTGS2	5	Constitutive activation of NF-κB is mediated through the TRADD-TRAF2-RIP-TAK1-IKK pathway, making TNF a novel target in the treatment of head and neck cancer [119].	4.60E-04
	hsa04010	MAPK signaling pathway	ARRB1, FLT1, HSPA8, IGF1R, MAPK10, MAPK8, MAPK9	7	Nearly 1/5 of head and neck squamous cell carcinoma (HNSCC) harbors MAPK pathway mutations, which are largely activating mutations [17].	5.60E-04
	hsa04110	Cell cycle	CCNA2, CDC14A, PRKDC, RBL1, YWHAZ	5	Cell cycle deregulation is a common feature of human cancer. Cancer cells frequently display unscheduled proliferation, genomic instability (increased DNA mutations and chromosomal aberrations) and chromosomal instability (changes in chromosome number) [120].	5.60E-04
Reactome	HSA-212436	Generic Transcription Pathway	BARD1, BAX, CCNA2, CHD3, CSNK2A1, GLI2, GLI3, GTF2H1, JAG1, KAT5, MDC1, MED1, MEF2C, NOTCH3, NR3C1, PARP1, RBL1, RPA1, RUNX3, SIN3A, SIRT1, SMARCA2, STAT1, TAF1, TAF2, TAF5, TXN, TXNRD1, UBA52, USP7, YWHAZ, YY1	32	Discussed before.	2.21E-18
	HSA-74160	Gene expression (Transcription)	BARD1, BAX, CCNA2, CHD3, CSNK2A1, ERCC6, GLI2, GLI3, GTF2H1, JAG1, KAT5, MDC1, MED1, MEF2C, NOTCH3, NR3C1, PARP1, RBL1, RPA1, RUNX3, SIN3A, SIRT1, SMARCA2, STAT1, TAF1, TAF2, TAF5, TXN, TXNRD1, UBA52, USP7, YWHAZ, YY1	33		1.88E-17
	HSA-3700989	Transcriptional Regulation by TP53	BARD1, BAX, CCNA2, CHD3, CSNK2A1, GTF2H1, KAT5, MDC1, RBL1, RPA1, TAF1, TAF2, TAF5, TXN, TXNRD1, UBA52, USP7, YWHAZ	18		1.21E-13
	HSA-73894	DNA Repair	BARD1, CCNA2, ERCC6, GTF2H1, KAT5, MAPK8, MDC1, PARP1, PRKDC, RPA1, TP53BP1, UBA52, USP7, YY1	14		1.00E-09
	HSA-5633007	Regulation of TP53 Activity	BARD1, CCNA2, CHD3, CSNK2A1, KAT5, RPA1, TAF1, TAF2, TAF5, UBA52, USP7	11		2.75E-09
	HSA-5693532	DNA Double-Strand Break Repair	BARD1, CCNA2, KAT5, MAPK8, MDC1, PARP1, PRKDC, RPA1, TP53BP1, UBA52	10	DNA-repair genes are, like detoxification enzymes, responsible for preventing cancer by protecting the integrity of the genome and are therefore considered as cancer-susceptibility genes [121].	2.53E-08
	HSA-449147	Signaling by Interleukins	APP, BCL2L1, FN1, HMGB1, HSPA8, MAPK10, MAPK8, MAPK9, MEF2C, PTGS2, STAT1, UBA52, YWHAZ	13	Signal transducer and activator of transcription 3 (STAT3) has been reported to be activated by interleukin-6 receptor (IL-6R) or epidermal growth factor receptor (EGFR) in head and neck squamous cell carcinomas (HNSCC), which may have important implications for responsiveness to therapeutics targeted at EGFR, IL-6R, or intermediary kinases [122].	5.31E-07
	HSA-168164	Toll Like Receptor 3 (TLR3) Cascade	APP, HMGB1, MAPK10, MAPK8, MAPK9, MEF2C, UBA52	7	Toll-like receptor 3 signaling induces apoptosis in human head and neck cancer via survivin associated pathway [123].	3.46E-06
	HSA-597592	Post-translational protein modification	APP, ARRB1, BARD1, CCNA2, CHD3, FN1, HSPA8, MDC1, NR3C1, PARP1, PRKDC, RPA1, SIN3A, TOP2A, TOP2B, TP53BP1, UBA52, USP7, YY1	19	Various lines of evidence have proven that protein post-translational modifications (PTMs) play an important role in the development of HNC, and that they are involved in the regulation of HNC proliferation, invasion and metastasis, chemo-resistance, radio-sensitivity, anti-apoptosis and so on [124].	5.67E-06
	HSA-5696398	Nucleotide Excision Repair	ERCC6, GTF2H1, PARP1, RPA1, UBA52, USP7, YY1	7	Reduced expression of the nucleotide excision repair genes ERCC1, XPB/ERCC3, XPG/ERCC5 and CSB/ERCC6 was associated with a more than 2-fold increase in the risk of squamous cell carcinoma of the head and neck [125].	7.02E-06
	HSA-1640170	Cell Cycle	BARD1, CCNA2, CDC14A, CSNK1E, CSNK2A1, KAT5, MDC1, RBL1, RPA1, TOP2A, TP53BP1, UBA52, YWHAZ	13	Discussed before.	1.20E-05
	HSA-5693571	Nonhomologous End-Joining (NHEJ)	BARD1, KAT5, MDC1, PRKDC, TP53BP1	5	TRIP13 promotes error-prone nonhomologous end joining and induces chemoresistance in head and neck cancer [126].	6.02E-05
	HSA-450294	MAP kinase activation	MAPK10, MAPK8, MAPK9, MEF2C, UBA52	5	Protein kinase C (PKC) zeta mediates EGF-stimulated MAPK activation in keratinocytes and SCCHN cell lines, and EGFR and MAPK-dependent proliferation in SCCHN cell lines [127].	1.30E-04
	HSA-8878166	Transcriptional regulation by RUNX2	BAX, GLI2, GLI3, NR3C1, STAT1, UBA52	6	Parathyroid Hormone-Like Hormone (PTHLH) expression is a poor prognosis marker in HNSCC patients, and RUNX2-PTHLH axis contributes to HNSCC tumor growth [128].	1.30E-04

Table S15. 26 driver candidates of "Gene level" of HNSC.

Gene	NCBI Entrez ID	CGC [Gene (Sequence similarity/E_value)]
BARD1	580	BCOR (40.299/8.86E-18)
BCL2L1	598	BCL2 (49.746/6.66E-56)
CHD3	1107	CHD4 (71.161/0), TRIM24 (49.091/8.86E-12), KDM5C (56.522/4.58E-11), TRIM33 (40.678/4.34E-10), KDM5A (52.174/2.22E-10), NSD3 (54.348/3.76E-10), NSD2 (47.17/1.04E-06)
EGR1	1958	WT1 (61.053/1.47E-32), KLF6 (59.016/2.34E-19), BCL6 (41.379/5.24E-09), BCL5 (41.379/5.24E-09), ZNF331 (44.048/3.82E-17), PRDM16 (45.122/8.63E-18), ZNF521 (40.777/1.02E-17), MECOM (45.122/1.23E-17), CTCF (42.857/3.31E-13), SALL4 (49.057/7.86E-11)
FLT1	2321	KDR (44.933/0), FLT4 (40.603/0), FLT3 (40.812/2.65E-104), FGFR4 (44.857/7.29E-88), RET (42.135/3.70E-78), MET (45.604/3.68E-45), PTK6 (47.647/1.77E-40), ABL2 (44.654/3.47E-39), ROS1 (43.506/1.65E-37), ALK (42.683/1.92E-37), ABL1 (40.719/8.67E-37), EGFR (41.975/2.94E-36), FES (41.718/4.07E-36), ERBB2 (42.405/7.47E-35), SYK (40.881/3.99E-31), JAK3 (42.529/1.62E-30)
GLI2	2736	WT1 (40.164/6.23E-22), ZNF331 (40.741/1.86E-14), BCL6 (43.956/4.04E-11), BCL5 (43.956/4.04E-11), BCL11B (45.283/2.68E-07), BCL11A (45.283/3.92E-07), SALL4 (40.741/3.17E-06)
GLI3	2737	WT1 (40.164/2.43E-21), ZNF331 (40.94/3.47E-19), BCL6 (43.956/5.42E-11), BCL5 (43.956/5.42E-11), KLF6 (40.789/4.53E-14), BCL11B (47.059/7.70E-07), BCL11A (47.059/1.07E-06), SALL4 (41.176/1.19E-05)
IGF1R	3480	ROS1 (40.302/1.30E-73), NTRK1 (41.892/2.27E-71), FGFR3 (40.21/5.94E-65), FGFR2 (40.351/1.35E-64), DDR2 (40.127/1.01E-58), ABL2 (41.606/1.51E-57), PDGFRA (40.462/8.93E-36), PDGFRB (41.176/2.73E-34)
JAG1	182	FAT4 (41.667/4.67E-05)
KAT5	10524	KAT6A (53.846/3.42E-94), KAT6B (51.471/7.79E-87)
MAPK10	5602	MAPK1 (42.535/3.80E-87)
MAPK8	5599	MAPK1 (42.735/4.93E-86)
MAPK9	5601	MAPK1 (41.274/1.00E-84)
NCOA3	8202	NCOA2 (42.394/0)
NEB	4703	LASP1 (72.464/6.43E-27), ABI1 (43.396/8.24E-06)
NOTCH3	4854	NOTCH2 (49.855/0), NOTCH1 (52.222/0), FAT4 (43.939/4.14E-27), FAT1 (40.523/6.39E-22)
NR3C1	2908	AR (51.752/5.44E-125)
PRKDC	5591	PIK3CA (42.424/2.61E-06)
PTGS2	5743	NOTCH2 (46.341/3.27E-06)
PTK2	5747	ABL1 (40.214/1.47E-63), ABL2 (40.58/1.32E-61), SRC (41.288/7.18E-59), ERBB2 (40.074/1.74E-55), FLT4 (40.331/8.14E-40), KDR (41.714/1.84E-38), FLT3 (40.909/7.65E-35), PDGFRB (40.385/6.40E-31)
RUNX3	864	RUNX1 (58.051/8.07E-171)
SMARCA2	6595	SMARCA4 (79.037/0), CHD4 (40.262/6.65E-109)
STAT1	6772	STAT3 (52.901/0)
TAF1	6872	TRIM24 (40.625/4.08E-08), TRIM33 (40.678/1.37E-06)
YWHAZ	7534	YWHAE (69.492/6.94E-119)
YY1	7528	ZNF384 (41.739/9.48E-22), ZBTB16 (40.909/1.96E-21), PRDM1 (42.727/8.18E-21), BCL6 (42.056/2.36E-16), BCL5 (42.056/2.36E-16), ZNF331 (41.818/2.84E-17), KLF6 (43.678/3.07E-16), KLF4 (43.023/4.45E-16), PRDM16 (42.857/4.49E-16), SALL4 (43.396/3.45E-07), BCL11B (48.148/1.23E-07), BCL11A (47.273/1.11E-07)

Table S16. Six levels of the 60 driver candidates of COADREAD.

No.	Level	Driver candidates	Count	Percentage
1	Cancer-type level	ABCB1, APEX1, DPYD, ERCC6, GLI3, IGF1R, ING1, INSR, NFKB1, PARP1, PCNA, TNFRSF10B, XRCC5	13	21.67%
2	Literature-supported level	APEX1, CCNA2, CDC25C, CDK2, CTNNA1, ERCC6, FN1, IGF1R, LYN, MAPK8, NFYA, NOTCH3, PIK3CG, SIRT1, TP53BP1, USP7	16	26.67%
3	Pathway level	ABCB1, APEX1, BARD1, BUB1, CCNA2, CDC25C, CDK2, CHD3, CSNK1D, CTNNA1, EGR1, ERCC6, FN1, FYN, GLI3, GSK3B, GTF2H1, HDAC1, HNF4A, HSPA8, HSPA9, IGF1R, INSR, LYN, MAPK10, MAPK3, MAPK8, MAPK9, MED1, NFKB1, NFYA, NOTCH3, PARP1, PCNA, PIK3CG, PLK1, POLA1, PRKCA, PRKDC, PRKG1, PTK2, ROCK1, RUVBL1, SIN3A, SIRT1, SMARCA2, SOS1, TAF1, TNFRSF10B, TOP2A, TOP2B, TP53BP1, TTN, USP7, VAV1, XRCC5	56	93.33%
4	Non-cancer disease level	▲	▲	▲
5	Gene level	BARD1, CDK2, CHD3, EGR1, FYN, GLI3, IGF1R, INSR, LYN, MAPK10, MAPK3, MAPK8, MAPK9, NFKB1, NOTCH3, PRKCA, PRKDC, PTK2, SMARCA2, TAF1	20	33.33%
6	Validation-required level	DMD, GNL3	2	3.33%

Table S17 16 driver candidates of "Literature-supported level" of COADREAD.

Gene	NCBI Entrez ID	Rank (HumanNet)	Rank (STRINGv10)	Function	Type
APEX1	328	35	210	A study suggests that APEX1 contributes to the aggressive behavior of colon cancer and functions as an upstream activator in the Jagged1/Notch signaling pathway and could be a potential therapeutic target for colon cancers that exhibit high levels of Jagged1/Notch signaling [129].	E
ERCC6	2074	63	55	Elevated expression of ERCC6 contributes to chemoresistance in CRC cells, and low expression of ERCC6 is associated with chemoresponse and survival in CRC patients. This protein represents a novel therapeutic target for improving chemotherapy efficacy and a biomarker for predicting patient survival [130].	E
IGF1R	3480	72	30	Overexpression of RTKs, including IGF-IR, has been demonstrated in CRC. Several experimental studies have shown that IGF1R plays a role in resistance to anti-EGFR therapy in mCRC patients treated with cetuximab [28].	O
PIK3CG	5294	38	67	It has been shown that silencing of the PIK3CG gene plays an important role in suppressing the PI3K-Akt/PKB signaling system, which is responsible for tumorigenesis and colorectal cancer progression [131].	E
MAPK8	5599	45	134	Recent experimental studies have shown that the PVT1/miR-152-3p/E2F3/MAPK8 axis promotes CRC progression [132].	E&B
TP53BP1	7158	48	75	A report showed that loss of 53BP1 was associated with poor survival in colorectal cancer through clinical data analysis. In addition, loss of 53BP1 inhibited apoptosis and induced proliferation in colorectal cancer cells [133].	E&B
USP7	7874	52	147	Preclinical study indicated that USP7 could be a potential drug target and its inhibitor P5091 deserves further development as anticancer agent for Wnt hyper-activated CRC therapy [134].	E
SIRT1	23411	71	207	SIRT1 plays a key role in maintaining the cellular properties of CSCs. SIRT1 is a potential independent prognostic factor for CRC patients after therapeutic tumor resection and will help provide a promising new approach to targeting CSCs in CRC therapy [135].	E
CDK2	1017	77	183	Combined PI3K and CDK2 inhibition induces cell death and enhances in vivo antitumour activity in colorectal cancer [136].	E
CDC25C	995	79	229	Recent study showed a novel synthetic lethality interaction between ARID1A and AURKA and indicated that pharmacologically inhibiting the AURKA–CDC25C axis represents a novel strategy for treating CRC with ARID1A loss-of-function mutations [137].	E
CCNA2	890	84	137	CCNA2, a novel oncogene, plays a role in regulating cancer cell growth and apoptosis. It can be used as a new biomarker for the diagnosis and treatment of CRC [138].	E&B
FN1	2335	98	50	FN1 suppressed apoptosis and promoted viability, invasion, and migration in CRC through interacting with ITGA5. FN1 may be a prognostic factor and potential target for CRC treatment [139].	E&B
CTNNA1	1495	128	82	It was shown that an allele of the αE-catenin (CTNNA1) gene is mutated in the human colon cancer cell family HCT-8, which is identical to HCT-15, DLD-1 and HRT-18. CTNNA1 gene is an invasion suppressor gene according to Knudsen's two-hit model for tumor suppressor genes [140].	E
LYN	4067	134	142	It has been shown that Lyn is involved in CD24-induced activation of ERK1/2 in colorectal cancer (CRC) and that CD24 expression is associated with activation of Lyn and ERK1/2, which may be a novel mechanism related to CD24-mediated regulation of CRC development [141].	E&B
NFYA	4800	199	211	Recent studies have shown that through the S100A2/KNPA2 complex, NFYA is transported to the nucleus and inhibits the transcriptional activity of E-cadherin, which in turn promotes CRC metastasis [142].	E
NOTCH3	4854	243	28	Notch3 can modulate the tumourigenic properties of CRC cells and contributes to sustained Notch activity in DLL4-expressing tumors [143].	E

Table S18. Biological pathways associated with COADREAD.

Database	Pathway ID	Pathway	Genes	Count	Function	FDR
	hsa04510	Focal adhesion	FN1, FYN, GSK3B, IGF1R, MAPK10, MAPK3, MAPK8, MAPK9, PRKCA, PTK2, ROCK1, SOS1, VAV1	13	In cancer, FAK has been shown to be involved in cell motility, invasion, survival, gene expression and self-renewal of cancer stem cells. Recently, it has been shown that the anti-invasive effect of resveratrol through inhibition of FAK activity has a potential beneficial effect on disease prevention and treatment of CRC [144].	9.84E-12
	hsa05206	MicroRNAs in cancer	ABCB1, CDC25C, HDAC1, MAPK3, NFKB1, NOTCH3, PRKCA, ROCK1, SIRT1, SOS1	10	MicroRNAs have been linked to CRC development, and these molecules have been recently studied as new potential biomarkers in diagnosis and treatment of CRC [145].	3.33E-09
	hsa04110	Cell cycle	BUB1, CCNA2, CDC25C, CDK2, GSK3B, HDAC1, PCNA, PLK1, PRKDC	9	Discussed before.	5.88E-09
	hsa04071	Sphingolipid signaling pathway	FYN, MAPK10, MAPK3, MAPK8, MAPK9, NFKB1, PRKCA, ROCK1	8	Sphingolipid metabolism could be a possible target for CRC chemoprevention and chemotherapy [146].	8.25E-08
	hsa04062	Chemokine signaling pathway	GSK3B, LYN, MAPK3, NFKB1, PIK3CG, PTK2, ROCK1, SOS1, VAV1	9	The chemokines associated with CRC are instrumental in shaping the immune microenvironment and clinical outcomes.The chemokines CXCR4 and CXCR7 are examples of tumour growth regulators as their expression in CRC increases with tumour stages. They enhance tumour growth and thus lead to a poor prognosis and decreased overall survival rates within CRC patients [147].	1.40E-07
	hsa04151	PI3K-Akt signaling pathway	CDK2, FN1, GSK3B, IGF1R, INSR, MAPK3, NFKB1, PIK3CG, PRKCA, PTK2, SOS1	11	PI3K/AKT signaling leads to reduced apoptosis, stimulates cell growth and increases proliferation. In colorectal cancer the most commonly observed pathway changes are IGF2 overexpression, PIK3CA mutations and PTEN mutations and deletions. Combined, these alterations are found in about 40% of large bowel tumors [148].	1.90E-07
	hsa04010	MAPK signaling pathway	HSPA8, IGF1R, INSR, MAPK10, MAPK3, MAPK8, MAPK9, NFKB1, PRKCA, SOS1	10	Activation of this signalling pathway is important in intestinal epithelial differentiation. There is growing evidence that activation of the ERK MAPK pathway is involved in the pathogenesis, progression, and oncogenic behaviour of human colorectal cancer [149].	3.46E-07
	hsa05165	Human papillomavirus infection	CCNA2, CDK2, FN1, GSK3B, HDAC1, MAPK3, NFKB1, NOTCH3, PTK2, SOS1	10	HPV is present in the colon and rectum of most patients with colorectal adenocarcinoma, suggesting that this virus may be related to the pathogenesis of colorectal cancer [150].	9.10E-07
	hsa04919	Thyroid hormone signaling pathway	GSK3B, HDAC1, MAPK3, MED1, NOTCH3, PRKCA, SIN3A	7	Thyroid hormone promotes β-Catenin activation and cell proliferation in colorectal cancer [151].	1.43E-06
KEGG	hsa05130	Pathogenic Escherichia coli infection	FYN, MAPK10, MAPK3, MAPK8, MAPK9, NFKB1, ROCK1, TNFRSF10B	8	Pathogenic E. coli could be a cofactor in pathogenesis of colorectal cancer [152].	1.62E-06
	hsa05205	Proteoglycans in cancer	FN1, IGF1R, MAPK3, PRKCA, PTK2, ROCK1, SOS1, VAV1	8	Heparan sulfate proteoglycans are candidate molecules to clarify colorectal cancer tumorigenesis, as well as important targets to therapy and diagnosis [153].	2.07E-06
	hsa05210	Colorectal cancer	GSK3B, MAPK10, MAPK3, MAPK8, MAPK9, SOS1	6	Colorectal cancer.	2.63E-06
	hsa04657	IL-17 signaling pathway	GSK3B, MAPK10, MAPK3, MAPK8, MAPK9, NFKB1	6	Chronic inflammation is known to promote colorectal tumorigenesis. The pro-inflammatory cytokines TNFα and IL-17 may contribute to this effect by stimulating glycolysis and growth factor production in colorectal cancer cells [154].	4.18E-06
	hsa04611	Platelet activation	FYN, LYN, MAPK3, PIK3CG, PRKG1, ROCK1	6	Enhanced platelet activation occurs in colorectal cancer patients. Permanent inactivation of platelet COX-1 by low-dose aspirin might restore anti-tumor reactivity [155].	1.63E-05
	hsa04520	Adherens junction	CTNNA1, FYN, IGF1R, INSR, MAPK3	5	Carcinoembryonic antigen promotes colorectal cancer progression by targeting adherens junction complexes [156].	1.65E-05
	hsa04072	Phospholipase D signaling pathway	FYN, INSR, MAPK3, PIK3CG, PRKCA, SOS1	6	Phopholipase D (PLD) isozyme acts as a novel transcriptional target and positive feedback regulator of Wnt signaling, and then promotes Wnt-driven anchorage-independent growth of colorectal cancer cells. Therapeutic interventions targeting PLD may confer a clinical benefit in Wnt/β-catenin-driven malignancies [157].	3.84E-05
	hsa04150	mTOR signaling pathway	GSK3B, IGF1R, INSR, MAPK3, PRKCA, SOS1	6	mTOR signaling is associated with the clinical pathological parameters of human CRC. siRNA-mediated gene silencing of mTOR may be a novel therapeutic strategy for CRC [158].	4.31E-05
	hsa04310	Wnt signaling pathway	GSK3B, MAPK10, MAPK8, MAPK9, PRKCA, RUVBL1	6	In CRC, WNT pathway hyperactivation is arguably the most critical cancer driver and represents an exciting avenue for targeted therapy [159].	4.66E-05
	hsa04620	Toll-like receptor signaling pathway	MAPK10, MAPK3, MAPK8, MAPK9, NFKB1	5	Persistent TLR-specific activation of NF-κB in CRC and particularly in tumor-initiating cells may sustain further tumor growth and progression through perpetuation of signaling from inflammatory and tissue repair mechanisms, with consequent self-renewal of pluripotent tumor cells [160].	8.53E-05
	HSA-212436	Generic Transcription Pathway	BARD1, CCNA2, CDC25C, CDK2, CHD3, GLI3, GSK3B, GTF2H1, HDAC1, HNF4A, MAPK3, MED1, NFKB1, NFYA, NOTCH3, PARP1, PCNA, SIN3A, SIRT1, SMARCA2, TAF1, TNFRSF10B, USP7	23		8.02E-10
	HSA-73894	DNA Repair	APEX1, BARD1, CCNA2, CDK2, ERCC6, GTF2H1, MAPK8, PARP1, PCNA, PRKDC, RUVBL1, TP53BP1, USP7, XRCC5	14		8.02E-10
	HSA-74160	Gene expression (Transcription)	BARD1, CCNA2, CDC25C, CDK2, CHD3, ERCC6, GLI3, GSK3B, GTF2H1, HDAC1, HNF4A, MAPK3, MED1, NFKB1, NFYA, NOTCH3, PARP1, PCNA, SIN3A, SIRT1, SMARCA2, TAF1, TNFRSF10B, USP7	24	Discussed before.	1.73E-09
	HSA-162582	Signal Transduction	BUB1, CDC25C, CDK2, CHD3, CTNNA1, EGR1, FN1, FYN, GLI3, GSK3B, HDAC1, IGF1R, INSR, LYN, MAPK3, MAPK8, MED1, NFKB1, NOTCH3, PARP1, PIK3CG, PLK1, PRKCA, PRKG1, PTK2, ROCK1, RUVBL1, SOS1, TNFRSF10B, USP7, VAV1	31		2.83E-09
	HSA-1640170	Cell Cycle	BARD1, BUB1, CCNA2, CDC25C, CDK2, CSNK1D, GSK3B, HDAC1, LYN, MAPK3, PCNA, PLK1, POLA1, PRKCA, RUVBL1, TOP2A, TP53BP1	17		2.94E-09
Reactome	HSA-9006934	Signaling by Receptor Tyrosine Kinases	CTNNA1, EGR1, FN1, FYN, IGF1R, INSR, LYN, MAPK3, PRKCA, PTK2, ROCK1, SOS1, VAV1	13	Combination of RTK and MEK inhibitors led to concomitant inhibition of PI3K and MEK signaling, marked growth suppression, and robust apoptosis of human KRAS mutant colorectal cancer cell lines in vitro and upon xenografting in mice [161].	6.73E-07
	HSA-76002	Platelet activation, signaling and aggregation	FN1, FYN, LYN, MAPK3, PIK3CG, PRKCA, PTK2, SOS1, TTN, VAV1	10	Discussed in "Platelet activation" pathway of KEGG.	1.31E-06
	HSA-449147	Signaling by Interleukins	FN1, FYN, HSPA8, HSPA9, LYN, MAPK10, MAPK3, MAPK8, MAPK9, NFKB1, SOS1, VAV1	12	In general, ILs facilitate CRC by promoting tumorigenesis, tumour growth, angiogenesis, and cancer cell invasion and metastasis and inhibit CRC via complex pathways. In addition, some clinical trials in progress are expected to lead to a breakthrough in the treatment of CRC [162].	1.43E-06
	HSA-3700989	Transcriptional Regulation by TP53	BARD1, CCNA2, CDC25C, CDK2, CHD3, GTF2H1, HDAC1, PCNA, TAF1, TNFRSF10B, USP7	11	Discussed before.	2.10E-06
	HSA-194315	Signaling by Rho GTPases	BUB1, CDC25C, CTNNA1, MAPK3, PLK1, PRKCA, PTK2, ROCK1, SOS1, VAV1	10	It has now been well established that increased expression of Rho GTPases correlates with tumor progression in colorectal cancer [163].	5.25E-05
	HSA-450294	MAP kinase activation	MAPK10, MAPK3, MAPK8, MAPK9, NFKB1	5	Mitogen-activated protein kinase activity drives cell trajectories in colorectal cancer [164].	1.00E-04
	HSA-597592	Post-translational protein modification	BARD1, CCNA2, CHD3, CSNK1D, FN1, HDAC1, HSPA8, PARP1, PCNA, PRKDC, RUVBL1, SIN3A, TOP2A, TOP2B, TP53BP1, USP7	`6	Accumulating evidence suggests that abnormal PTM events are associated with a variety of human diseases, such as CRC, thus highlighting the need for studying PTMs to discover both the molecular mechanisms and therapeutic targets of CRC [165].	1.50E-04

Table S19. 20 driver candidates of "Gene level" of COADREAD.

Gene	NCBI Entrez ID	CGC [Gene (Sequence similarity/E_value)]
BARD1	580	BCOR (40.299/8.86E-18)
CDK2	1017	CDK6 (48.495/4.54E-93), CDK4 (45.424/8.14E-82), CDK12 (43.934/7.16E-74)
CHD3	1107	CHD4 (71.161/0), TRIM24 (49.091/8.86E-12), KDM5C (56.522/4.58E-11), TRIM33 (40.678/4.34E-10), KDM5A (52.174/2.22E-10), NSD3 (54.348/3.76E-10), NSD2 (47.17/1.04E-06)
EGR1	1958	WT1 (61.053/1.47E-32), KLF6 (59.016/2.34E-19), BCL6 (41.379/5.24E-09), BCL5 (41.379/5.24E-09), ZNF331 (44.048/3.82E-17), PRDM16 (45.122/8.63E-18), ZNF521 (40.777/1.02E-17), MECOM (45.122/1.23E-17), CTCF (42.857/3.31E-13), SALL4 (49.057/7.86E-11)
FYN	2534	SRC (75.732/0), LCK (62.806/0), PTK6 (43.304/4.23E-131), ABL2 (45.372/8.47E-120), ABL1 (44.244/1.17E-118), ROS1 (40.86/2.29E-56), ERBB4 (41.379/9.24E-50), ERBB2 (40.37/3.53E-49), PDGFRA (40.594/3.52E-11), KIT (44.371/2.47E-37), FLT4 (40.606/3.25E-34), PDGFRB (40.881/1.20E-32)
GLI3	2737	WT1 (40.164/2.43E-21), ZNF331 (40.94/3.47E-19), BCL6 (43.956/5.42E-11), BCL5 (43.956/5.42E-11), KLF6 (40.789/4.53E-14), BCL11B (47.059/7.70E-07), BCL11A (47.059/1.07E-06), SALL4 (41.176/1.19E-05)
IGF1R	3480	ROS1 (40.302/1.30E-73), NTRK1 (41.892/2.27E-71), FGFR3 (40.21/5.94E-65), FGFR2 (40.351/1.35E-64), DDR2 (40.127/1.01E-58), ABL2 (41.606/1.51E-57), PDGFRA (40.462/8.93E-36), PDGFRB (41.176/2.73E-34)
INSR	3643	ROS1 (49.64/2.50E-73), NTRK1 (42.568/7.32E-69), FGFR3 (40.702/1.05E-63), MET (42.105/2.40E-57), ABL1 (40.293/4.29E-57), KIT (42.038/1.84E-36), PDGFRA (41.714/8.88E-36), PDGFRB (40.667/1.35E-32)
LYN	4067	LCK (67.292/0), SRC (60.271/0), ABL1 (41.648/4.59E-113), ABL2 (41.203/3.66E-112), PDGFRA (41.071/9.31E-13), PDGFRB (48.322/1.99E-39), KIT (45.161/1.99E-38), FLT3 (42.391/2.54E-38), KDR (42.012/6.19E-35)
MAPK10	5602	MAPK1 (42.535/3.80E-87)
MAPK3	5595	MAPK1 (88.15/0)
MAPK8	5599	MAPK1 (42.735/4.93E-86)
MAPK9	5601	MAPK1 (41.274/1.00E-84)
NFKB1	4790	NFKB2 (42.193/0), REL (44.713/4.02E-77)
NOTCH3	4854	NOTCH2 (49.855/0), NOTCH1 (52.222/0), FAT4 (43.939/4.14E-27), FAT1 (40.523/6.39E-22)
PRKCA	5578	PRKACA (100/0), AKT2 (47.432/1.54E-104), AKT1 (47.436/1.49E-100)
PRKDC	5591	PIK3CA (42.424/2.61E-06)
PTK2	5747	ABL1 (40.214/1.47E-63), ABL2 (40.58/1.32E-61), SRC (41.288/7.18E-59), ERBB2 (40.074/1.74E-55), FLT4 (40.331/8.14E-40), KDR (41.714/1.84E-38), FLT3 (40.909/7.65E-35), PDGFRB (40.385/6.40E-31)
SMARCA2	6595	SMARCA4 (79.037/0), CHD4 (40.262/6.65E-109)
TAF1	6872	TRIM24 (40.625/4.08E-08), TRIM33 (40.678/1.37E-06)

Table S20. Six levels of the 70 driver candidates of BLCA.

No.	Level	Driver candidates	Count	Percentage
1	Cancer-type level	ABCB1, AHR, BARD1, CDC25A, CDKN1A, CHEK1, ERCC6, GSK3B, IGF1R, INSR, NCOA3, PARP1, PCNA, PLK1, PRKDC, RAD51, STAT1	17	24.29%
2	Literature-supported level	CDKN1A, EGR1, FOXM1, GLI3, HDAC1, INSR, KLF5, MDC1, MED1, NR3C1, PLK1, SIRT1, SMARCC1, STAT1, TOP2A, YWHAZ	16	22.86%
3	Pathway level	ABCB1, ACTA2, ACTB, BARD1, CASP1, CDC14A, CDC25A, CDKN1A, CHD3, CHEK1, CSNK2A1, CUL1, EEF2, EGR1, ERCC6, FOXM1, GLI3, GSK3B, HDAC1, HDAC2, HSPA8, IGF1R, INSR, KAT2A, KAT2B, KAT5, MAPK10, MAPK3, MAPK8, MAPK9, MDC1, MED1, NFKBIA, NR3C1, PARP1, PCNA, PLK1, PRKDC, PTK2, RYR1, RYR2, SIN3A, SIRT1, SMARCA2, SMARCC1, SOS1, STAT1, SYNE1, TAF1, TAF2, TOP2A, TOP2B, TP53BP1, TP53BP2, UBA52, USP7, YWHAZ	57	81.43%
4	Non-cancer disease level	▲	▲	▲
5	Gene level	BARD1, CDKN1A, CHD3, EGR1, FOXM1, GLI3, IGF1R, INSR, KAT5, KLF5, MAPK10, MAPK3, MAPK8, MAPK9, NCOA3, NEB, NR3C1, PRKDC, PTK2, SMARCA2, SRCAP, STAT1, TAF1, TP53BP2, YWHAZ	25	35.71%
6	Validation-required level	HSPA9, TTN, GNL3, DMD, MAP1B, CAD, HSPA5	7	10.00%

Table S21 16 driver candidates of “Literature-supported level” of BLCA.

Gene	NCBI Entrez ID	Rank (HumanNet)	Rank (STRINGv10)	Function	Type
CDKN1A	1026	9	8	A report of whole genome sequencing of different subtypes of bladder cancer demonstrated CDKN1A as a tumor suppressor gene in bladder cancer [32].	E
INSR	3643	38	246	Data suggest that IGF-2/INSR mediated paracrine crosstalk between bladder cancer cells and endothelial cells is functionally involved in tumour angiogenesis and may thus represent a new therapeutic target [33].	E
PLK1	5347	119	197	Five downstream genes of PLK1 were associated with the regulation of cell proliferation, invasion and migration in bladder cancer. Furthermore, these genes may play important roles in bladder cancer and become important biomarkers and targets for cancer treatment [166].	B
STAT1	6772	180	68	One study provided an apparent prognostic prediction model for bladder cancer and identified STAT1 as a key gene in a gene regulatory network related to the immune phenotype of bladder cancer [34].	B
SIRT1	23411	29	69	SIRT1 overexpression significantly promoted the transcriptional activity and expression of GLUT1, indicating that SIRT1 increases the transcription activity and expression of GLUT1, therefore, promoting the cell proliferation and glycolysis in BC cells [167].	E&B
YWHAZ	7534	30	92	YWHAZ plays an essential role in sustaining cell proliferation during chemo/radiotherapy. Treatments based on anti-YWHAZ strategies may thus be beneficial for UCUB patients overexpressing YWHAZ [168].	E&B
TOP2A	7153	33	77	TOP2A was overexpressed in BLCA and could serve as a prognostic biomarker for BLCA. Moreover, TOP2A is functionally important for the proliferation, invasion and survival of BLCA cells [169].	B
GLI3	2737	48	161	miR-7-5p, a mircoRNA, acts as a tumor suppressor in BCa by downregulating Gli3 [170].	B
EGR1	1958	80	66	Promoter CpG hypomethylation and transcription factor EGR1 hyperactivate heparanase expression in bladder cancer, where heparanase plays a critical role in the degradation of extracellular matrix and cell membrane and is frequently upregulated in malignant tumors [171].	E&B
MED1	5469	84	83	The downregulation of MED1 is associated with muscle invasion, metastatic spread, and shorter overall survival in BCa [172].	B
NR3C1	2908	87	220	circNR3C1 is significantly downregulated in bladder cancer tissues and cell lines, and it suppresses cell cycle progression by directly sponging miR-27a-3p to block its interaction with cyclin D1 mRNA 5′UTR, and subsequently inhibits cyclin D1 expression to impair proliferation of bladder cancer cells [173].	B
HDAC1	3065	93	89	The maspin/HDAC1 signaling axis may represent the antitumor characteristics in human bladder carcinoma cells [174].	E
SMARCC1	6599	167	179	Recent study demonstrated KPNA2, Nup50 and Nup153 regulate the process of SMARCC1 nuclear translocation in BC and SMARCC1 may be a competent candidate as a diagnostic and therapeutic target for BC [175].	E&B
MDC1	9656	197	114	One study identified a novel antisense lncRNA, MDC1-AS, which may participate in bladder cancer through up-regulation of its antisense tumor-suppressing gene MDC1 [176].	E
FOXM1	2305	23	84	A recent study uncovered a novel FOXM1/RNF26/p57 axis that modulates the cell cycle process and enhances the progression of bladder cancer. Thus, the FOXM1/RNF26/p57 signaling axis could be a candidate target for the treatment of bladder cancer [177].	B
KLF5	688	238	43	Studies suggest that the KLF5 transcription factor plays an oncogenic role in the TSU-Pr1 bladder cancer cell line through the regulation of a subset of genes [178].	E

Table S22. Biological pathways associated with BLCA.

Database	Pathway ID	Pathway	Genes	Count	Function	FDR
KEGG	hsa04110	Cell cycle	CDC14A, CDC25A, CDKN1A, CHEK1, CUL1, GSK3B, HDAC1, HDAC2, PCNA, PLK1, PRKDC, YWHAZ	12	Discussed before.	1.31E-11
	hsa04012	ErbB signaling pathway	CDKN1A, GSK3B, MAPK10, MAPK3, MAPK8, MAPK9, PTK2, SOS1	8	Functional in vitro findings provide evidence that the viability of SCC-derived cells strongly depends on ERBB signaling suggesting anti-EGFR TKI therapy as a valid target, in particular when combined with standard chemotherapy [179].	4.18E-08
	hsa04510	Focal adhesion	ACTB, GSK3B, IGF1R, MAPK10, MAPK3, MAPK8, MAPK9, PTK2, SOS1	9	Focal adhesion kinases crucially regulate TGFβ-induced migration and invasion of bladder cancer cells via Src kinase and E-cadherin [180].	9.20E-07
	hsa05206	MicroRNAs in cancer	ABCB1, CDC25A, CDKN1A, HDAC1, HDAC2, MAPK3, SIRT1, SOS1	8	A growing body of evidence suggests that miRNAs contribute to bladder cancer development, progression and metastasis. Genome-wide miRNA expression signatures have been used to rapidly and precisely identify aberrant miRNA expression in bladder cancer [181].	1.95E-06
	hsa04620	Toll-like receptor signaling pathway	MAPK10, MAPK3, MAPK8, MAPK9, NFKBIA, STAT1	6	Decreased TLRs expression was found in bladder tumours, particularly in non-muscle-invasive ones. Bacillus Calmette-Guerin (BCG) (agonist of TLR2 and TLR4) is approved by US FDA for immunotherapy of bladder cancer [182].	1.75E-05
	hsa04010	MAPK signaling pathway	HSPA8, IGF1R, INSR, MAPK10, MAPK3, MAPK8, MAPK9, SOS1	8	The Ras-MAPK pathway is important to orchestrating a cell's response to external and internal stimuli. This pathway is commonly dysregulated in cancer, including bladder cancer [183].	6.71E-05
	hsa04014	Ras signaling pathway	IGF1R, INSR, MAPK10, MAPK3, MAPK8, MAPK9, SOS1	7		1.20E-04
	hsa04310	Wnt signaling pathway	CSNK2A1, CUL1, GSK3B, MAPK10, MAPK8, MAPK9	6	A review highlights the importance of Wnt signalling associated components as possible targets that could be exploited for the development of novel and more effective preventive, diagnostic and therapeutic approaches for bladder cancer [184].	1.30E-04
	hsa04330	Notch signaling pathway	HDAC1, HDAC2, KAT2A, KAT2B	4	Loss of Notch activity is a driving event in urothelial cancer [185].	2.40E-04
	hsa04371	Apelin signaling pathway	ACTA2, EGR1, MAPK3, RYR1, RYR2	5	Apelin might be a therapeutic potential biomarker in muscle-invasive bladder cancer patients [186].	5.60E-04
	hsa04150	mTOR signaling pathway	GSK3B, IGF1R, INSR, MAPK3, SOS1	5	A subset of mammalian target of rapamycin (mTOR) pathway alterations have been shown to occur in bladder cancer9 and appear to influence tumor behavior [187].	9.00E-04
Reactome	HSA-212436	Generic Transcription Pathway	BARD1, CASP1, CDKN1A, CHD3, CHEK1, CSNK2A1, CUL1, GLI3, GSK3B, HDAC1, HDAC2, KAT2A, KAT2B, KAT5, MAPK3, MDC1, MED1, NR3C1, PARP1, PCNA, SIN3A, SIRT1, SMARCA2, SMARCC1, STAT1, TAF1, TAF2, TP53BP2, UBA52, USP7, YWHAZ	31	Discussed before.	4.82E-16
	HSA-74160	Gene expression (Transcription)	ACTB, BARD1, CASP1, CDKN1A, CHD3, CHEK1, CSNK2A1, CUL1, ERCC6, GLI3, GSK3B, HDAC1, HDAC2, KAT2A, KAT2B, KAT5, MAPK3, MDC1, MED1, NR3C1, PARP1, PCNA, SIN3A, SIRT1, SMARCA2, SMARCC1, STAT1, TAF1, TAF2, TP53BP2, UBA52, USP7, YWHAZ	33		5.22E-16
	HSA-3700989	Transcriptional Regulation by TP53	BARD1, CASP1, CDKN1A, CHD3, CHEK1, CSNK2A1, HDAC1, HDAC2, KAT5, MDC1, PCNA, TAF1, TAF2, TP53BP2, UBA52, USP7, YWHAZ	17		8.82E-12
	HSA-1640170	Cell Cycle	BARD1, CDC14A, CDC25A, CDKN1A, CHEK1, CSNK2A1, CUL1, FOXM1, GSK3B, HDAC1, KAT5, MAPK3, MDC1, PCNA, PLK1, SYNE1, TOP2A, TP53BP1, UBA52, YWHAZ	20		4.52E-11
	HSA-5633007	Regulation of TP53 Activity	BARD1, CHD3, CHEK1, CSNK2A1, HDAC1, HDAC2, KAT5, TAF1, TAF2, TP53BP2, UBA52, USP7	12		3.20E-10
	HSA-73894	DNA Repair	ACTB, BARD1, CHEK1, ERCC6, KAT5, MAPK8, MDC1, PARP1, PCNA, PRKDC, TP53BP1, UBA52, USP7	13		1.97E-08
	HSA-9614085	FOXO-mediated transcription	CDKN1A, HDAC1, HDAC2, KAT2B, NR3C1, SIN3A, SIRT1, YWHAZ	8	Over-expression of miR-96 targets and down-regulates the expression of FOXO1 which is the leading cause of accelerated tumour cell proliferation and inhibits apoptosis in enumerated cancers like bladder cancer, cervical cancer, colorectal cancer and prostate cancer [188].	3.37E-08
	HSA-597592	Post-translational protein modification	ACTB, BARD1, CDC25A, CHD3, CUL1, EEF2, HDAC1, HDAC2, HSPA8, KAT2A, KAT2B, MDC1, NFKBIA, NR3C1, PARP1, PCNA, PRKDC, SIN3A, TOP2A, TOP2B, TP53BP1, UBA52, USP7	23	Post-translational modifications (PTMs) constitute an untapped repertoire of tumor targets in bladder cancer and PTM-targeted therapy could potentially overcome tumor heterogeneity [189].	4.19E-08
	HSA-8878166	Transcriptional regulation by RUNX2	CDKN1A, CUL1, GLI3, GSK3B, MAPK3, NR3C1, STAT1, UBA52	8	High infiltration of Cancer-associated fibroblasts (CAFs) is associated with tumor progression and poor prognosis in BLCA. RUNX2 is a transcription factor related to CAFs, which is overexpressed in bladder cancer and affects the prognosis. RUNX2 is a potential marker relating CAFs and therapy target in BLCA [190].	1.61E-06
	HSA-6807070	PTEN Regulation	CHD3, CSNK2A1, EGR1, HDAC1, HDAC2, MAPK3, UBA52, USP7	8	A present review demonstrated that PTEN functions as an onco-suppressor that diminishes growth and proliferation of bladder cancer cells. In vitro, in vivo and clinical trials have examined the onco-suppressor role of PTEN in bladder cancer [191].	2.98E-06
	HSA-157118	Signaling by NOTCH	ACTA2, CUL1, HDAC1, HDAC2, KAT2A, KAT2B, STAT1, UBA52, YWHAZ	9	Studies indicating that NOTCH1 and NOTCH2 have opposite effects on the progression of bladder cancer could give rise to potential therapeutic approaches aimed at blocking or restoring the Notch pathway [192].	3.31E-06
	HSA-3247509	Chromatin modifying enzymes	ACTB, CHD3, HDAC1, HDAC2, KAT2A, KAT2B, KAT5, SMARCA2, SMARCC1	9	Mutations in chromatin modifying enzymes are wide-spread in bladder cancer and several promising therapeutic targets for modulating activity of these genes are currently in clinical trials [193].	7.57E-06
	HSA-5696398	Nucleotide Excision Repair	ACTB, ERCC6, PARP1, PCNA, UBA52, USP7	6	Common genetic variation in nucleotide excision repair (NER) might influence the risk of smoking-related cancers, such as bladder cancer [194].	8.84E-05
	HSA-9006934	Signaling by Receptor Tyrosine Kinases	ACTB, CHEK1, EGR1, IGF1R, INSR, MAPK3, PTK2, SOS1, STAT1, UBA52	10	Receptor tyrosine kinase EphB4 is thus a potential candidate as a predictor of disease outcome in bladder cancer and as target for novel therapy [195].	2.80E-04
	HSA-156711	Polo-like kinase mediated events	CDC25A, FOXM1, PLK1	3	Targeted inhibition of Polo-like kinase 1 by a novel small-molecule inhibitor induces mitotic catastrophe and apoptosis in human bladder cancer cells [196].	8.10E-04

Table S23. 25 driver candidates of "Gene level" of BLCA.

Gene	NCBI Entrez ID	CGC [Gene (Sequence similarity/E_value)]
BARD1	580	BCOR (40.299/8.86E-18)
CDKN1A	1026	CDKN1B (42.857/9.18E-16)
CHD3	1107	CHD4 (71.161/0), TRIM24 (49.091/8.86E-12), KDM5C (56.522/4.58E-11), TRIM33 (40.678/4.34E-10), KDM5A (52.174/2.22E-10), NSD3 (54.348/3.76E-10), NSD2 (47.17/1.04E-06)
EGR1	1958	WT1 (61.053/1.47E-32), KLF6 (59.016/2.34E-19), BCL6 (41.379/5.24E-09), BCL5 (41.379/5.24E-09), ZNF331 (44.048/3.82E-17), PRDM16 (45.122/8.63E-18), ZNF521 (40.777/1.02E-17), MECOM (45.122/1.23E-17), CTCF (42.857/3.31E-13), SALL4 (49.057/7.86E-11)
FOXM1	2305	FOXA1 (42.424/5.45E-18), FOXL2 (44.048/2.32E-17), FOXO4 (45.122/3.84E-16), FOXO3 (46.341/5.39E-16), FOXO1 (45.122/6.36E-16), FOXP1 (45.333/1.18E-15)
GLI3	2737	WT1 (40.164/2.43E-21), ZNF331 (40.94/3.47E-19), BCL6 (43.956/5.42E-11), BCL5 (43.956/5.42E-11), KLF6 (40.789/4.53E-14), BCL11B (47.059/7.70E-07), BCL11A (47.059/1.07E-06), SALL4 (41.176/1.19E-05)
IGF1R	3480	ROS1 (40.302/1.30E-73), NTRK1 (41.892/2.27E-71), FGFR3 (40.21/5.94E-65), FGFR2 (40.351/1.35E-64), DDR2 (40.127/1.01E-58), ABL2 (41.606/1.51E-57), PDGFRA (40.462/8.93E-36), PDGFRB (41.176/2.73E-34)
INSR	3643	ROS1 (49.64/2.50E-73), NTRK1 (42.568/7.32E-69), FGFR3 (40.702/1.05E-63), MET (42.105/2.40E-57), ABL1 (40.293/4.29E-57), KIT (42.038/1.84E-36), PDGFRA (41.714/8.88E-36), PDGFRB (40.667/1.35E-32)
KAT5	10524	KAT6A (53.846/3.42E-94), KAT6B (51.471/7.79E-87)
KLF5	688	KLF6 (76.042/2.49E-50), KLF4 (73.469/4.34E-46), WT1 (56.383/5.56E-31), PRDM16 (44.33/2.99E-19), MECOM (40.909/1.22E-18), PRDM1 (42.105/8.12E-16), BCL11B (42.697/1.71E-15), BCL11A (42.697/5.23E-15), PATZ1 (42.857/1.14E-14), ZNF331 (46.053/4.29E-13), SALL4 (40.426/1.30E-07)
MAPK10	5602	MAPK1 (42.535/3.80E-87)
MAPK3	5595	MAPK1 (88.15/0)
MAPK8	5599	MAPK1 (42.735/4.93E-86)
MAPK9	5601	MAPK1 (41.274/1.00E-84)
NCOA3	8202	NCOA2 (42.394/0)
NEB	4703	LASP1 (72.464/6.43E-27), ABI1 (43.396/8.24E-06)
NR3C1	2908	AR (51.752/5.44E-125)
PRKDC	5591	PIK3CA (42.424/2.61E-06)
PTK2	5747	ABL1 (40.214/1.47E-63), ABL2 (40.58/1.32E-61), SRC (41.288/7.18E-59), ERBB2 (40.074/1.74E-55), FLT4 (40.331/8.14E-40), KDR (41.714/1.84E-38), FLT3 (40.909/7.65E-35), PDGFRB (40.385/6.40E-31)
SMARCA2	6595	SMARCA4 (79.037/0), CHD4 (40.262/6.65E-109)
SRCAP	10847	SMARCA4 (47.097/1.54E-40), CHD4 (42.714/1.03E-41)
STAT1	6772	STAT3 (52.901/0)
TAF1	6872	TRIM24 (40.625/4.08E-08), TRIM33 (40.678/1.37E-06)
TP53BP2	7159	SH3GL1 (40.426/2.18E-06)
YWHAZ	7534	YWHAE (69.492/6.94E-119)

Table S24. Six levels of the 52 driver candidates of UCEC.

No.	Level	Driver candidates	Count	Percentage
1	Cancer-type level	▲	▲	▲
2	Literature-supported level	AKT3, CDH2, CSNK2A1, IGF1R, SGK1, SKP2, TOP2A, YY1	8	15.38%
3	Pathway level	ABCB1, AKT3, AXL, CASP1, CD4, CHD3, CSNK2A1, CTNNA1, FLT1, GNA13, IGF1R, INSR, KIF23, MAPK8, PDPK1, PIK3CB, PIK3CG, PPP2CA, PRKCA, PTK2, PTK2B, RASA1, ROCK2, SGK1, SHC1, SKP2, SOS1, TAF1, TEK, TLR2, TTN, TYK2, USP7, VAV1	34	65.38%
4	Non-cancer disease level	▲	▲	▲
5	Gene level	AKT3, AXL, CDH2, CHD3, FLT1, GLI3, GNA13, IGF1R. INSR, KIF23, MAPK8, NR3C1, PIK3CB, PPP2CA, PRKCA, PRKDC, PTK2, PTK2B, SGK1, SHC1, SMARCA2, TAF1, TEK, TYK2, YY1	25	48.08%
6	Validation-required level	HSPA4, EEF2, TP53BP1, SIN3A, POLA1, TOP2B, GNL3, ERCC6, BIRC5, KAT2B, ING1	11	21.15%

Table S25 8 driver candidates of "Literature-supported level" of UCEC.

Gene	NCBI Entrez ID	Rank (HumanNet)	Rank (STRINGv10)	Function	Type
IGF1R	3480	11	84	The study validated IGF-1R as a target for endometrial cancer treatment and clearly demonstrated that IGFIR inhibitors, including tyrosine kinase inhibitors and IGF1R antibodies, have potential therapeutic benefits in endometrial cancer treatment [40].	E&B
CSNK2A1	1357	13	81	Putative endometrial cancer biomarkers, including CSNK2A1, were identified by a novel graph convolutional sample network approach, which may be helpful in future investigations of the molecular mechanisms and therapeutic targets of endometrial cancers [197].	B
SGK1	6446	27	19	The increased expression of SGK1 in endometrial cancer tissues suggests a role for SGK1 in this type of cancer. Furthermore, the SGK1 inhibitor SI113 induced a significant decrease in endometrial cancer cell viability, suggesting that SGK1 may be an attractive molecular target for the treatment of endometrial cancer [41].	E
AKT3	10000	37	68	In recent study, AKT3 has been observed to be reversely correlated with miR-582-5p and also validated as a direct target of miR-582-5p in endometrial cancer. Moreover, AKT3 is also involved in regulation of cell proliferation and apoptosis in ECC1 cells [42].	E
TOP2A	7153	64	135	Patients with TOP2A overexpression have a worse prognosis compared with those with TOP2A nonexpression, and TOP2A may be a useful biomarker in patients receiving adjuvant taxane-platinum regimens with moderate- to high-risk endometrial cancer [198].	B
SKP2	6502	139	202	High expression of skp2 was linked to poor prognostic factors of the clinicopathological parameters and poor prognosis. Therefore, skp2 expression is a good indicator for the analysis of the poor prognosis of patients with endometrial adenocarcinoma [199].	B
CDH2	1000	201	173	In vitro study showed that in Ishikawa endometrial carcinoma cells, downregulation of PTEN was associated with the expression of the CDH1 and CDH2 genes and upregulated expression of the cell membrane glycoprotein, CD133, which may be associated with epithelial-mesenchymal transition (EMT) in malignancy [200].	E
YY1	7528	212	160	Study demonstrated that YY1 is upregulated in EEC cell lines and primary tumors; and its expression is associated with tumor stages. Depletion of YY1 inhibits EEC cell proliferation and migration both in vitro and in vivo, whereas overexpression of YY1 promotes EEC cell growth [201].	E&B

Table S26. Biological pathways associated with UCEC.

Database	Pathway ID	Pathway	Genes	Count	Function	FDR
KEGG	hsa04151	PI3K-Akt signaling pathway	AKT3, FLT1, IGF1R, INSR, PDPK1, PIK3CB, PIK3CG, PPP2CA, PRKCA, PTK2, SGK1, SOS1, TEK, TLR2	14	Overactivation of the PI3K/AKT/mTOR pathway, a signaling pathway that plays an important role in cellular growth and survival, has recently been implicated in endometrial cancer pathogenesis, and as such, inhibition of the PI3K/AKT/mTOR pathway is of therapeutic interest [202].	7.86E-11
	hsa04150	mTOR signaling pathway	AKT3, IGF1R, INSR, PDPK1, PIK3CB, PRKCA, SGK1, SKP2, SOS1	9		1.46E-08
	hsa04068	FoxO signaling pathway	AKT3, IGF1R, INSR, MAPK8, PDPK1, PIK3CB, SGK1, SKP2, SOS1, USP7	10	Loss of FOXO1 perturbs endometrial homeostasis, promotes uncontrolled cell proliferation and increases susceptibility to genotoxic insults [203].	2.73E-10
	hsa04071	Sphingolipid signaling pathway	AKT3, GNA13, MAPK8, PDPK1, PIK3CB, PPP2CA, PRKCA, ROCK2	8	Endometrial carcinoma is characterized by profound changes in sphingolipid metabolism that likely contribute to its progression and chemoresistance [204].	4.06E-08
	hsa04010	MAPK signaling pathway	AKT3, FLT1, IGF1R, INSR, MAPK8, PRKCA, RASA1, SOS1, TEK	9	Estrogen receptor alpha activates MAPK signaling pathway to promote the development of endometrial cancer [205].	1.06E-06
	hsa04910	Insulin signaling pathway	AKT3, INSR, MAPK8, PDPK1, PIK3CB, SHC1, SOS1	7	Insulin resistance plays a central role in endometrial cancer development. Understanding the relationship between insulin resistance and endometrial cancer may supply new ideas to fight this malignancy [206].	1.06E-06
	hsa04931	Insulin resistance	AKT3, INSR, MAPK8, PDPK1, PIK3CB	5		7.98E-05
	hsa05213	Endometrial cancer	AKT3, CTNNA1, PDPK1, PIK3CB, SOS1	5	Cancer itself.	6.31E-06
	hsa04926	Relaxin signaling pathway	AKT3, MAPK8, PIK3CB, PRKCA, SHC1, SOS1	6	Relaxin 2/RXFP1 signaling induces cell invasion via the β-catenin pathway in endometrial cancer [207].	1.28E-05
	hsa04917	Prolactin signaling pathway	AKT3, MAPK8, PIK3CB, SHC1, SOS1	5	Tumor expression of human growth hormone and human prolactin predict a worse survival outcome in patients with mammary or endometrial carcinoma [208].	1.30E-05
	hsa05235	PD-L1 expression and PD-1 checkpoint pathway in cancer	AKT3, CD4, CSNK2A1, PIK3CB, TLR2	5	There was significant association of PD-L1 expression in both tumor cells and immune cells with advanced stage endometrial cancer [209].	3.50E-05
	hsa05206	MicroRNAs in cancer	ABCB1, KIF23, PIK3CB, PRKCA, SHC1, SOS1	6	In endometrial cancer, miRNAs are associated with regulation of gene expression, epigenetic dysfunction and carcinogenesis. Thus, miRNAs are likely to have key roles in diagnosis, prognostic prediction, and therapy in endometrial cancer [210].	3.61E-05
Reactome	HSA-194138	Signaling by VEGF	AKT3, AXL, CTNNA1, FLT1, PDPK1, PIK3CB, PRKCA, PTK2, PTK2B, RASA1, ROCK2, VAV1	12	VEGF-mTOR signaling drives endometrial cell growth leading to hyperplasia and cancer [211].	4.63E-13
	HSA-76002	Platelet activation, signaling and aggregation	GNA13, PDPK1, PIK3CB, PIK3CG, PRKCA, PTK2, SHC1, SOS1, TTN, VAV1	10	Mean platelet volume (MPV) was found to be a marker for predicting advanced-stage endometrial cancers [212].	6.09E-07
	HSA-5633007	Regulation of TP53 Activity	AKT3, CHD3, CSNK2A1, PDPK1, PPP2CA, SGK1, TAF1, USP7	8	Discussed before.	3.08E-06
	HSA-6804757	Regulation of TP53 Degradation	AKT3, PDPK1, PPP2CA, SGK1, USP7	5		1.10E-05
	HSA-3700989	Transcriptional Regulation by TP53	AKT3, CASP1, CHD3, CSNK2A1, PDPK1, PPP2CA, SGK1, TAF1, USP7	9		4.99E-05
	HSA-2428924	IGF1R signaling cascade	IGF1R, PDPK1, PIK3CB, SHC1, SOS1	5	ALKBH5 promoted proliferation and invasion of endometrial cancer via erasing IGF1R m6A-modifications, which suggests a potential therapeutic target for endometrial cancer [213].	4.81E-05
	HSA-74751	Insulin receptor signalling cascade	INSR, PDPK1, PIK3CB, SHC1, SOS1	5	Estrogen and insulin synergistically promote endometrial cancer progression via crosstalk between their receptor signaling pathways [214].	4.99E-05
	HSA-354192	Integrin signaling	PDPK1, PTK2, SHC1, SOS1	4	CTHRC1 promotes M2-like macrophage recruitment and myometrial invasion in endometrial carcinoma by integrin-Akt signaling pathway [215].	1.00E-04
	HSA-1280215	Cytokine Signaling in Immune system	AKT3, CASP1, CD4, MAPK8, PIK3CB, PPP2CA, PTK2B, SHC1, SOS1, TYK2, VAV1	11	Cytokines play a pivotal role in malignant endometrium and carcinogenesis [216].	1.10E-04

Table S27. 25 driver candidates of "Gene level" of UCEC.

Gene	NCBI Entrez ID	CGC [Gene (Sequence similarity/E_value)]
AKT3	10000	AKT1 (82.708/0), AKT2 (77.273/0), PRKACA (41.645/6.12E-103)
AXL	558	MET (41.195/9.58E-68), ABL1 (44.086/1.57E-62), FGFR1 (40.122/6.05E-61), ABL2 (42.806/1.52E-60), FGFR3 (40.136/1.35E-59), KDR (40.187/1.30E-41), PDGFRB (41.718/1.17E-35)
CDH2	1000	CDH1 (46.614/0)
CHD3	1107	CHD4 (71.161/0), TRIM24 (49.091/8.86E-12), KDM5C (56.522/4.58E-11), TRIM33 (40.678/4.34E-10), KDM5A (52.174/2.22E-10), NSD3 (54.348/3.76E-10), NSD2 (47.17/1.04E-06)
FLT1	2321	KDR (44.933/0), FLT4 (40.603/0), FLT3 (40.812/2.65E-104), FGFR4 (44.857/7.29E-88), RET (42.135/3.70E-78), MET (45.604/3.68E-45), PTK6 (47.647/1.77E-40), ABL2 (44.654/3.47E-39), ROS1 (43.506/1.65E-37), ALK (42.683/1.92E-37), ABL1 (40.719/8.67E-37), EGFR (41.975/2.94E-36), FES (41.718/4.07E-36), ERBB2 (42.405/7.47E-35), SYK (40.881/3.99E-31), JAK3 (42.529/1.62E-30)
GLI3	2737	WT1 (40.164/2.43E-21), ZNF331 (40.94/3.47E-19), BCL6 (43.956/5.42E-11), BCL5 (43.956/5.42E-11), KLF6 (40.789/4.53E-14), BCL11B (47.059/7.70E-07), BCL11A (47.059/1.07E-06), SALL4 (41.176/1.19E-05)
GNA13	10672	GNAQ (45.556/2.93E-110), GNA11 (45/2.08E-106)
IGF1R	3480	ROS1 (40.302/1.30E-73), NTRK1 (41.892/2.27E-71), FGFR3 (40.21/5.94E-65), FGFR2 (40.351/1.35E-64), DDR2 (40.127/1.01E-58), ABL2 (41.606/1.51E-57), PDGFRA (40.462/8.93E-36), PDGFRB (41.176/2.73E-34)
INSR	3643	ROS1 (49.64/2.50E-73), NTRK1 (42.568/7.32E-69), FGFR3 (40.702/1.05E-63), MET (42.105/2.40E-57), ABL1 (40.293/4.29E-57), KIT (42.038/1.84E-36), PDGFRA (41.714/8.88E-36), PDGFRB (40.667/1.35E-32)
KIF23	9493	KIF5B (42.857/1.04E-14)
MAPK8	5599	MAPK1 (42.735/4.93E-86)
NR3C1	2908	AR (51.752/5.44E-125)
PIK3CB	5291	PIK3CA (40.796/0)
PPP2CA	5515	PPP6C (58.14/6.69E-131)
PRKCA	5578	PRKACA (100/0), AKT2 (47.432/1.54E-104), AKT1 (47.436/1.49E-100)
PRKDC	5591	PIK3CA (42.424/2.61E-06)
PTK2	5747	ABL1 (40.214/1.47E-63), ABL2 (40.58/1.32E-61), SRC (41.288/7.18E-59), ERBB2 (40.074/1.74E-55), FLT4 (40.331/8.14E-40), KDR (41.714/1.84E-38), FLT3 (40.909/7.65E-35), PDGFRB (40.385/6.40E-31)
PTK2B	2185	ROS1 (42.697/2.24E-52), FLT4 (45.342/2.22E-38), KDR (43.195/2.11E-37), KIT (40.667/3.18E-32)
SGK1	6446	AKT2 (48.837/6.11E-114), AKT1 (46.011/2.94E-112)
SHC1	6464	LCK (41.818/2.77E-05)
SMARCA2	6595	SMARCA4 (79.037/0), CHD4 (40.262/6.65E-109)
TAF1	6872	TRIM24 (40.625/4.08E-08), TRIM33 (40.678/1.37E-06)
TEK	7010	FGFR3 (45.819/4.72E-74), FGFR2 (46.545/1.87E-72), FGFR4 (47.636/2.08E-70), RET (44.863/1.21E-69), PDGFRA (43.814/7.99E-44), KIT (41.919/4.96E-41), PDGFRB (47.17/9.89E-40)
TYK2	7297	JAK1 (46.814/0)
YY1	7528	ZNF384 (41.739/9.48E-22), ZBTB16 (40.909/1.96E-21), PRDM1 (42.727/8.18E-21), BCL6 (42.056/2.36E-16), BCL5 (42.056/2.36E-16), ZNF331 (41.818/2.84E-17), KLF6 (43.678/3.07E-16), KLF4 (43.023/4.45E-16), PRDM16 (42.857/4.49E-16), SALL4 (43.396/3.45E-07), BCL11B (48.148/1.23E-07), BCL11A (47.273/1.11E-07)

Table S28. Six levels of the 45 driver candidates of KIRC.

No.	Level	Driver candidates	Count	Percentage
1	Cancer-type level	▲	▲	▲
2	Literature-supported level	AHR, CDK2, CSF1R, CUL1, EGR1, HDAC1, KAT2A, PRKCE, SMARCA2, STAT1, TOP2A, VCP	12	26.67%
3	Pathway level	ATP2A2, CCNB2, CDK2, CDK8, CUL1, EGR1, GLI3, HDAC1, KAT2A, KAT2B, MCM7, ORC1, PCNA, PSME3, RPS27A, RPTOR, SIN3A, SMARCA2, SMC3, STAT1, TOP2A	21	46.67%
4	Non-cancer disease level	▲	▲	▲
5	Gene level	CDK2, CSF1R, EGR1, GLI3, INO80, INSR, NEB, PABPC1, PRKCE, SMARCA2, STAT1, TAF1, VCAN	13	28.89%
6	Validation-required level	FN1, RPS3A, VWF, EIF5A, RPL11, DST, CAD, TTN, HSPA8, ERCC6, CUL2, HSPA9, ACLY, HSPA5	14	31.11%

Table S29 12 driver candidates of "Literature-supported level" of KIRC.

Gene	NCBI Entrez ID	Rank (HumanNet)	Rank (STRINGv10)	Function	Type
CDK2	1017	2	117	CDK1 and CDK2 activity is a strong predictor of renal cell carcinoma recurrence [43].	B
SMARCA2	6595	29	122	High SMARCA2 expression was associated with good prognosis and benign differentiated tumors in kidney renal clear cell carcinoma [44].	B
KAT2A	2648	100	169	Recent study indicated that KAT2A was an oncogenic chromatin modifier that promotes RCC progression by inducing MCT1 expression [217].	B
PRKCE	5581	114	182	Recent studies have shown that PRKCE may serve as a novel prognostic biomarker reflecting the level of immune infiltration and a novel therapeutic target for KIRC [218].	B
HDAC1	3065	133	12	HDAC1 and HDAC6 may play a role in ccRCC biology and could represent rational therapeutic targets [219].	E
TOP2A	7153	153	21	High mRNA levels of TOP2A is an independent predictors of poor outcome in RCC patients and may be used for individual risk-adapted therapy in the future [220].	E&B
EGR1	1958	154	144	MAML1 may be a component of the transcriptional networks which regulate EGR1 target genes during nephrogenesis and could also have implications for the development of renal cell carcinoma [45].	E
VCP	7415	180	194	Recent studies have identified five aging-related genes (DUSP22, MAPK14, MAPKAPK3, STAT1 and VCP) from patients with kidney cancer that are significantly associated with patient survival [221].	B
CUL1	8454	182	55	Knockdown of Cul1 inhibits RCC cell migration and invasion abilities by up-regulating the expression of TIMP-1 and Cul1 knockdown significantly reduced the tumor growth in vivo. Cul1 may constitute a potential therapeutic target in RCC [222].	E
AHR	196	192	121	Recent studies have demonstrated that AhR is linked to renal cell carcinoma (RCC) [223].	O
CSF1R	1436	240	151	CSF1R plays a relevant role in clear cell renal cell carcinoma carcinogenesis and raise the possibility that CSF1R may represent a future valuable therapeutic target in these patients [224].	E&B
STAT1	6772	245	35	STAT1 may play a key role in RCC radioresistance and manipulation of this pathway may enhance the efficacy of radiotherapy [46].	E&B

Table S30. Biological pathways associated with KIRC.

Database	Pathway ID	Pathway	Genes	Count	Function	FDR
KEGG	hsa04110	Cell cycle	CCNB2, CDK2, CUL1, HDAC1, MCM7, ORC1, PCNA, SMC3	8	Discussed before.	1.56E-07
	hsa04919	Thyroid hormone signaling pathway	ATP2A2, HDAC1, KAT2A, KAT2B, SIN3A, STAT1	6	It was found that TRα (both α1 and α2) mRNA amount was significantly decreased in tumors while compared with healthy kidney tissue, and this decrease was deepest in G1 (well differentiated) renal clear cell carcinoma [225].	6.64E-05
Reactome	HSA-453279	Mitotic G1 phase and G1/S transition	CDK2, CUL1, HDAC1, MCM7, ORC1, PCNA, PSME3, RPS27A, TOP2A	9	Transcription factor NFYA promotes G1/S cell cycle transition and cell proliferation by transactivating cyclin D1 and CDK4 in clear cell renal cell carcinoma [226].	1.52E-07
	HSA-69206	G1/S Transition	CDK2, CUL1, HDAC1, MCM7, ORC1, PCNA, PSME3, RPS27A	8		5.79E-07
	HSA-157118	Signaling by NOTCH	ATP2A2, CDK8, CUL1, HDAC1, KAT2A, KAT2B, PSME3, RPS27A, STAT1	9	Notch cascade may represent a novel and therapeutically accessible pathway in clear cell renal cell carcinoma [227].	5.79E-07
	HSA-8878171	Transcriptional regulation by RUNX1	HDAC1, KAT2B, PSME3, RPS27A, SIN3A, SMARCA2	6	RUNX1 is a driver of renal cell carcinoma correlating with clinical outcome [228].	4.60E-04
	HSA-8878166	Transcriptional regulation by RUNX2	CUL1, GLI3, PSME3, RPS27A, STAT1	5	Long noncoding RNA SNHG4 promotes renal cell carcinoma tumorigenesis and invasion by acting as ceRNA to sponge miR-204-5p and upregulate RUNX2 [229].	4.70E-04
	HSA-6807070	PTEN Regulation	EGR1, HDAC1, PSME3, RPS27A, RPTOR	5	NOTCH1 functions as an oncogene by regulating the PTEN/PI3K/AKT pathway in clear cell renal cell carcinoma [230].	8.10E-04

Table S31. 13 driver candidates of "Gene level" of KIRC.

Gene	NCBI Entrez ID	CGC [Gene (Sequence similarity/E_value)]
CDK2	1017	CDK6 (48.495/4.54E-93), CDK4 (45.424/8.14E-82), CDK12 (43.934/7.16E-74)
CSF1R	1436	KIT (42.049/0), FGFR4 (43.353/2.02E-80), ABL2 (45.033/6.82E-42), ABL1 (40.698/1.27E-41), LCK (41.52/1.24E-39), SRC (40.936/2.67E-39), FES (44.97/2.95E-39), NTRK1 (43.713/3.22E-39), PTK6 (42.424/2.98E-36), MET (43.396/1.47E-35)
EGR1	1958	WT1 (61.053/1.47E-32), KLF6 (59.016/2.34E-19), BCL6 (41.379/5.24E-09), BCL5 (41.379/5.24E-09), ZNF331 (44.048/3.82E-17), PRDM16 (45.122/8.63E-18), ZNF521 (40.777/1.02E-17), MECOM (45.122/1.23E-17), CTCF (42.857/3.31E-13), SALL4 (49.057/7.86E-11)
GLI3	2737	WT1 (40.164/2.43E-21), ZNF331 (40.94/3.47E-19), BCL6 (43.956/5.42E-11), BCL5 (43.956/5.42E-11), KLF6 (40.789/4.53E-14), BCL11B (47.059/7.70E-07), BCL11A (47.059/1.07E-06), SALL4 (41.176/1.19E-05)
INO80	54617	SMARCA4 (48.077/1.77E-40), CHD4 (50.943/7.07E-38)
INSR	3643	ROS1 (49.64/2.50E-73), NTRK1 (42.568/7.32E-69), FGFR3 (40.702/1.05E-63), MET (42.105/2.40E-57), ABL1 (40.293/4.29E-57), KIT (42.038/1.84E-36), PDGFRA (41.714/8.88E-36), PDGFRB (40.667/1.35E-32)
NEB	4703	LASP1 (72.464/6.43E-27), ABI1 (43.396/8.24E-06)
PABPC1	26986	UBR5 (47.887/4.80E-12)
PRKCE	5581	PRKACA (47.239/0), AKT2 (46.264/7.50E-105), AKT1 (46.961/1.16E-104)
SMARCA2	6595	SMARCA4 (79.037/0), CHD4 (40.262/6.65E-109)
STAT1	6772	STAT3 (52.901/0)
TAF1	6872	TRIM24 (40.625/4.08E-08), TRIM33 (40.678/1.37E-06)
VCAN	1462	NOTCH2 (52.381/3.46E-15), NOTCH1 (49.315/1.16E-15), FAT1 (48.571/3.81E-05)

Table S32. Six levels of the 56 driver candidates of KIRP.

No.	Level	Driver candidates	Count	Percentage
1	Cancer-type level	▲	▲	▲
2	Literature-supported level	▲	▲	▲
3	Pathway level	ACTB, ACTL6A, CDC27, CNOT1, CSNK1E, CSNK2A1, DHX9, EIF4G1, GRB2, HSPA8, IGF1R, NR3C1, POLR1A, POLR1B, POLR2B, POLR3A. PRPF8, RPL11, SIN3A, SMARCC1. SMARCC2, SUPT5H, TNPO1, UBA52, UPF1, WWP1, XRN1	27	48.21%
4	Non-cancer disease level	▲	▲	▲
5	Gene level	CACNA1A, CUBN, GRB2, IGF1R, LRP2, MYH10, NEB, NR3C1, SMARCA1, SRCAP, WWP1	11	19.64%
6	Validation-required level	ANK2, HSPH1, EEF1A1, RRP12, CCT4, DST, HSPA1A, EEF2, GBF1, HSPA2, VAV1, TTN, ANK1, OGDH, HSPA9, ATXN2, MAT2A, ITGB4, CAD, IL32, GAPDH, SMC6	22	39.29%

Table S33. 11 driver candidates of "Gene level" of KIRP.

Gene	NCBI Entrez ID	CGC [Gene (Sequence similarity/E_value)]
CACNA1A	773	CACNA1D (51.124/0)
CUBN	8029	FAT4 (41.935/3.76E-08), FAT1 (43.836/1.82E-12), LRP1B (44/4.98E-06)
GRB2	2885	SH3GL1 (50/2.47E-13), ABI1 (42/2.30E-09), SRGAP3 (43.396/1.43E-07), ARHGAP26 (41.509/3.79E-07)
IGF1R	3480	ROS1 (40.302/1.30E-73), NTRK1 (41.892/2.27E-71), FGFR3 (40.21/5.94E-65), FGFR2 (40.351/1.35E-64), DDR2 (40.127/1.01E-58), ABL2 (41.606/1.51E-57), PDGFRA (40.462/8.93E-36), PDGFRB (41.176/2.73E-34)
LRP2	4036	LRP1B (40.336/1.99E-06)
MYH10	4628	MYH9 (77.772/0), MYH11 (76.152/0)
NEB	4703	LASP1 (72.464/6.43E-27), ABI1 (43.396/8.24E-06)
NR3C1	2908	AR (51.752/5.44E-125)
SMARCA1	6594	SMARCA4 (41.679/7.78E-146)
SRCAP	10847	SMARCA4 (47.097/1.54E-40), CHD4 (42.714/1.03E-41)
WWP1	11059	BAP1 (62.5/3.10E-07), WWTR1 (56.25/3.71E-06)

References

1. Wedge DC, Gundem G, Mitchell T, Woodcock DJ, Martincorena I, Ghori M, et al. Sequencing of prostate cancers identifies new cancer genes, routes of progression and drug targets. Nature Genetics. 2018;50 5:682-92. doi:10.1038/s41588-018-0086-z.
2. Balk SP and Knudsen KE. AR, the cell cycle, and prostate cancer. Nucl Recept Signal. 2008;6:e001. doi:10.1621/nrs.06001.
3. Faisal FA, Murali S, Kaur H, Vidotto T, Guedes LB, Salles DC, et al. CDKN1B Deletions are Associated with Metastasis in African American Men with Clinically Localized, Surgically Treated Prostate Cancer. Clin Cancer Res. 2020;26 11:2595-602. doi:10.1158/1078-0432.CCR-19-1669.
4. Wang XD, Leow CC, Zha J, Tang Z, Modrusan Z, Radtke F, et al. Notch signaling is required for normal prostatic epithelial cell proliferation and differentiation. Dev Biol. 2006;290 1:66-80. doi:10.1016/j.ydbio.2005.11.009.
5. Zhou B, Zhang J, Zhu H and Wu S. A Potential Prognostic Marker PRDM1 in Pancreatic Adenocarcinoma. J Oncol. 2022;2022:1934381. doi:10.1155/2022/1934381.
6. Han D, Chen S, Han W, Gao S, Owiredu JN, Li M, et al. ZBTB7A Mediates the Transcriptional Repression Activity of the Androgen Receptor in Prostate Cancer. Cancer Res. 2019;79 20:5260-71. doi:10.1158/0008-5472.CAN-19-0815.
7. Kibel AS, Suarez BK, Belani J, Oh J, Webster R, Brophy-Ebbers M, et al. CDKN1A and CDKN1B polymorphisms and risk of advanced prostate carcinoma. Cancer Res. 2003;63 9:2033-6.
8. Cheung AH, Tong JH, Chung LY, Chau SL, Ng CS, Wan IYP, et al. EGFR mutation exists in squamous cell lung carcinoma. Pathology. 2020;52 3:323-8. doi:10.1016/j.pathol.2019.12.003.
9. Liao RG, Jung J, Tchaicha J, Wilkerson MD, Sivachenko A, Beauchamp EM, et al. Inhibitor-sensitive FGFR2 and FGFR3 mutations in lung squamous cell carcinoma. Cancer Res. 2013;73 16:5195-205. doi:10.1158/0008-5472.CAN-12-3950.
10. Kim HS, Mitsudomi T, Soo RA and Cho BC. Personalized therapy on the horizon for squamous cell carcinoma of the lung. Lung Cancer. 2013;80 3:249-55. doi:10.1016/j.lungcan.2013.02.015.
11. McGowan M, Hoven AS, Lund-Iversen M, Solberg S, Helland A, Hirsch FR, et al. PIK3CA mutations as prognostic factor in squamous cell lung carcinoma. Lung Cancer. 2017;103:52-7. doi:10.1016/j.lungcan.2016.11.018.
12. Apopa PL, Alley L, Penney RB, Arnaoutakis K, Steliga MA, Jeffus S, et al. PARP1 Is Up-Regulated in Non-small Cell Lung Cancer Tissues in the Presence of the Cyanobacterial Toxin Microcystin. Front Microbiol. 2018;9:1757. doi:10.3389/fmicb.2018.01757.
13. Zeng J, Xie S, Liu Y, Shen C, Song X, Zhou GL, et al. CDK5 Functions as a Tumor Promoter in Human Lung Cancer. J Cancer. 2018;9 21:3950-61. doi:10.7150/jca.25967.
14. Sun Q, Zhang SY, Zhao JF, Han XG, Wang HB and Sun ML. HIF-1alpha or HOTTIP/CTCF Promotes Head and Neck Squamous Cell Carcinoma Progression and Drug Resistance by Targeting HOXA9. Mol Ther Nucleic Acids. 2020;20:164-75. doi:10.1016/j.omtn.2019.12.045.
15. Chen Z, Zhang C, Chen J, Wang D, Tu J, Van Waes C, et al. The Proteomic Landscape of Growth Factor Signaling Networks Associated with FAT1 Mutations in Head and Neck

Cancers. Cancer Res. 2021;81 17:4402-16. doi:10.1158/0008-5472.CAN-20-3659.
16. von Massenhausen A, Deng M, Billig H, Queisser A, Vogel W, Kristiansen G, et al. Evaluation of FGFR3 as a Therapeutic Target in Head and Neck Squamous Cell Carcinoma. Target Oncol. 2016;11 5:631-42. doi:10.1007/s11523-016-0431-z.
17. Ngan HL, Law CH, Choi YCY, Chan JY and Lui VWY. Precision drugging of the MAPK pathway in head and neck cancer. NPJ Genom Med. 2022;7 1:20. doi:10.1038/s41525-022-00293-1.
18. Grilli G, Hermida-Prado F, Alvarez-Fernandez M, Allonca E, Alvarez-Gonzalez M, Astudillo A, et al. Impact of notch signaling on the prognosis of patients with head and neck squamous cell carcinoma. Oral Oncol. 2020;110:105003. doi:10.1016/j.oraloncology.2020.105003.
19. Shah PA, Huang C, Li Q, Kazi SA, Byers LA, Wang J, et al. NOTCH1 Signaling in Head and Neck Squamous Cell Carcinoma. Cells. 2020;9 12 doi:10.3390/cells9122677.
20. Qiu W, Schonleben F, Li X, Ho DJ, Close LG, Manolidis S, et al. PIK3CA mutations in head and neck squamous cell carcinoma. Clin Cancer Res. 2006;12 5:1441-6. doi:10.1158/1078-0432.CCR-05-2173.
21. Dale OT, Aleksic T, Shah KA, Han C, Mehanna H, Rapozo DC, et al. IGF-1R expression is associated with HPV-negative status and adverse survival in head and neck squamous cell cancer. Carcinogenesis. 2015;36 6:648-55. doi:10.1093/carcin/bgv053.
22. Feng B, Shen Y, Pastor Hostench X, Bieg M, Plath M, Ishaque N, et al. Integrative Analysis of Multi-omics Data Identified EGFR and PTGS2 as Key Nodes in a Gene Regulatory Network Related to Immune Phenotypes in Head and Neck Cancer. Clin Cancer Res. 2020;26 14:3616-28. doi:10.1158/1078-0432.CCR-19-3997.
23. Maurer CA, Friess H, Kretschmann B, Zimmermann A, Stauffer A, Baer HU, et al. Increased expression of erbB3 in colorectal cancer is associated with concomitant increase in the level of erbB2. Hum Pathol. 1998;29 8:771-7. doi:10.1016/s0046-8177(98)90444-0.
24. Williams CS, Bernard JK, Demory Beckler M, Almohazey D, Washington MK, Smith JJ, et al. ERBB4 is over-expressed in human colon cancer and enhances cellular transformation. Carcinogenesis. 2015;36 7:710-8. doi:10.1093/carcin/bgv049.
25. Ibrahim HM, Abdelrahman AE, Elwan A, Bakry A, Fahmy MM, Abdelhamid MI, et al. Prognostic Impact of FSTL3, ADAM12, and FAT4 in Patients of Colon Cancer: Clinicopathologic Study. Appl Immunohistochem Mol Morphol. 2023; doi:10.1097/PAI.0000000000001157.
26. Liao X, Morikawa T, Lochhead P, Imamura Y, Kuchiba A, Yamauchi M, et al. Prognostic role of PIK3CA mutation in colorectal cancer: cohort study and literature review. Clin Cancer Res. 2012;18 8:2257-68. doi:10.1158/1078-0432.CCR-11-2410.
27. Yao B, Gui T, Zeng X, Deng Y, Wang Z, Wang Y, et al. PRMT1-mediated H4R3me2a recruits SMARCA4 to promote colorectal cancer progression by enhancing EGFR signaling. Genome Med. 2021;13 1:58. doi:10.1186/s13073-021-00871-5.
28. Shali H, Ahmadi M, Kafil HS, Dorosti A and Yousefi M. IGF1R and c-met as therapeutic targets for colorectal cancer. Biomed Pharmacother. 2016;82:528-36. doi:10.1016/j.biopha.2016.05.034.
29. Williams SV, Hurst CD and Knowles MA. Oncogenic FGFR3 gene fusions in bladder

cancer. Hum Mol Genet. 2013;22 4:795-803. doi:10.1093/hmg/dds486.

30. Luo W, Zhu Y, Zhang H, Qu Y and Ye D. Pd12-09 Elevated Semaphorin-6a Facilitates Flt4-Meditated Tumor Migration and Associates with Immune Suppression in Bladder Cancer. Journal of Urology. 2022;207 Supplement 5 doi:10.1097/ju.0000000000002538.09.

31. Kompier LC, Lurkin I, van der Aa MN, van Rhijn BW, van der Kwast TH and Zwarthoff EC. FGFR3, HRAS, KRAS, NRAS and PIK3CA mutations in bladder cancer and their potential as biomarkers for surveillance and therapy. PLoS One. 2010;5 11:e13821. doi:10.1371/journal.pone.0013821.

32. Cazier JB, Rao SR, McLean CM, Walker AK, Wright BJ, Jaeger EE, et al. Whole-genome sequencing of bladder cancers reveals somatic CDKN1A mutations and clinicopathological associations with mutation burden. Nat Commun. 2014;5:3756. doi:10.1038/ncomms4756.

33. Roudnicky F, Dieterich LC, Poyet C, Buser L, Wild P, Tang D, et al. High expression of insulin receptor on tumour-associated blood vessels in invasive bladder cancer predicts poor overall and progression-free survival. J Pathol. 2017;242 2:193-205. doi:10.1002/path.4892.

34. Weng H, Yuan S, Huang Q, Zeng XT and Wang XH. STAT1 is a key gene in a gene regulatory network related to immune phenotypes in bladder cancer: An integrative analysis of multi-omics data. J Cell Mol Med. 2021;25 7:3258-71. doi:10.1111/jcmm.16395.

35. Brooks R, Darcy K, Tritchler D, Gold D, Birrer M, Rader J, et al. Single nucleotide polymorphisms in IL8RB, EGFR, ABL1, and SPAG9 Are associated with lymph node metastasis in endometrial cancer: A Gynecologic Oncology Group and Washington University School of Medicine study. Gynecologic Oncology. 2012;125 doi:10.1016/j.ygyno.2011.12.040.

36. Cohen Y, Shalmon B, Korach J, Barshack I, Fridman E and Rechavi G. AKT1 pleckstrin homology domain E17K activating mutation in endometrial carcinoma. Gynecol Oncol. 2010;116 1:88-91. doi:10.1016/j.ygyno.2009.09.038.

37. Jeske YW, Ali S, Byron SA, Gao F, Mannel RS, Ghebre RG, et al. FGFR2 mutations are associated with poor outcomes in endometrioid endometrial cancer: An NRG Oncology/Gynecologic Oncology Group study. Gynecol Oncol. 2017;145 2:366-73. doi:10.1016/j.ygyno.2017.02.031.

38. van der Zee M, Sacchetti A, Cansoy M, Joosten R, Teeuwssen M, Heijmans-Antonissen C, et al. IL6/JAK1/STAT3 Signaling Blockade in Endometrial Cancer Affects the ALDHhi/CD126+ Stem-like Component and Reduces Tumor Burden. Cancer Res. 2015;75 17:3608-22. doi:10.1158/0008-5472.CAN-14-2498.

39. Hayes MP, Wang H, Espinal-Witter R, Douglas W, Solomon GJ, Baker SJ, et al. PIK3CA and PTEN mutations in uterine endometrioid carcinoma and complex atypical hyperplasia. Clin Cancer Res. 2006;12 20 Pt 1:5932-5. doi:10.1158/1078-0432.CCR-06-1375.

40. Bitelman C, Sarfstein R, Sarig M, Attias-Geva Z, Fishman A, Werner H, et al. IGF1R-directed targeted therapy enhances the cytotoxic effect of chemotherapy in endometrial cancer. Cancer Lett. 2013;335 1:153-9. doi:10.1016/j.canlet.2013.02.009.

41. Conza D, Mirra P, Cali G, Tortora T, Insabato L, Fiory F, et al. The SGK1 inhibitor SI113 induces autophagy, apoptosis, and endoplasmic reticulum stress in endometrial cancer cells. J Cell Physiol. 2017;232 12:3735-43. doi:10.1002/jcp.25850.
42. Li L and Ma L. Upregulation of miR-582-5p regulates cell proliferation and apoptosis by targeting AKT3 in human endometrial carcinoma. Saudi J Biol Sci. 2018;25 5:965-70. doi:10.1016/j.sjbs.2018.03.007.
43. Hongo F, Takaha N, Oishi M, Ueda T, Nakamura T, Naitoh Y, et al. CDK1 and CDK2 activity is a strong predictor of renal cell carcinoma recurrence. Urol Oncol. 2014;32 8:1240-6. doi:10.1016/j.urolonc.2014.05.006.
44. Guerrero-Martinez JA and Reyes JC. High expression of SMARCA4 or SMARCA2 is frequently associated with an opposite prognosis in cancer. Sci Rep. 2018;8 1:2043. doi:10.1038/s41598-018-20217-3.
45. Hansson ML, Behmer S, Ceder R, Mohammadi S, Preta G, Grafstrom RC, et al. MAML1 acts cooperatively with EGR1 to activate EGR1-regulated promoters: implications for nephrogenesis and the development of renal cancer. PLoS One. 2012;7 9:e46001. doi:10.1371/journal.pone.0046001.
46. Hui Z, Tretiakova M, Zhang Z, Li Y, Wang X, Zhu JX, et al. Radiosensitization by inhibiting STAT1 in renal cell carcinoma. Int J Radiat Oncol Biol Phys. 2009;73 1:288-95. doi:10.1016/j.ijrobp.2008.08.043.
47. Hirata H, Hinoda Y, Kawamoto K, Kikuno N, Suehiro Y, Okayama N, et al. Mismatch repair gene MSH3 polymorphism is associated with the risk of sporadic prostate cancer. J Urol. 2008;179 5:2020-4. doi:10.1016/j.juro.2008.01.009.
48. Zhang X, Hu L, Du M, Wei X, Zhang J, Hui Y, et al. Eukaryotic Elongation Factor 2 (eEF2) is a Potential Biomarker of Prostate Cancer. Pathol Oncol Res. 2018;24 4:885-90. doi:10.1007/s12253-017-0302-7.
49. Tang S, Sethunath V, Metaferia NY, Nogueira MF, Gallant DS, Garner ER, et al. A genome-scale CRISPR screen reveals PRMT1 as a critical regulator of androgen receptor signaling in prostate cancer. Cell Rep. 2022;38 8:110417. doi:10.1016/j.celrep.2022.110417.
50. Sankpal UT, Goodison S, Abdelrahim M and Basha R. Targeting Sp1 transcription factors in prostate cancer therapy. Med Chem. 2011;7 5:518-25. doi:10.2174/157340611796799203.
51. Chiu YT, Han HY, Leung SC, Yuen HF, Chau CW, Guo Z, et al. CDC25A functions as a novel Ar corepressor in prostate cancer cells. J Mol Biol. 2009;385 2:446-56. doi:10.1016/j.jmb.2008.10.070.
52. Murata T, Takayama K, Urano T, Fujimura T, Ashikari D, Obinata D, et al. 14-3-3zeta, a novel androgen-responsive gene, is upregulated in prostate cancer and promotes prostate cancer cell proliferation and survival. Clin Cancer Res. 2012;18 20:5617-27. doi:10.1158/1078-0432.CCR-12-0281.
53. Lage-Vickers S, Bizzotto J, Valacco MP, Sanchis P, Nemirovsky S, Labanca E, et al. The expression of YWHAZ and NDRG1 predicts aggressive outcome in human prostate cancer. Commun Biol. 2021;4 1:103. doi:10.1038/s42003-020-01645-2.
54. Li L, Ameri AH, Wang S, Jansson KH, Casey OM, Yang Q, et al. EGR1 regulates angiogenic and osteoclastogenic factors in prostate cancer and promotes metastasis.

Oncogene. 2019;38 35:6241-55. doi:10.1038/s41388-019-0873-8.
55. Darrington RS, Campa VM, Walker MM, Bengoa-Vergniory N, Gorrono-Etxebarria I, Uysal-Onganer P, et al. Distinct expression and activity of GSK-3alpha and GSK-3beta in prostate cancer. Int J Cancer. 2012;131 6:E872-83. doi:10.1002/ijc.27620.
56. Slupianek A, Yerrum S, Safadi FF and Monroy MA. The chromatin remodeling factor SRCAP modulates expression of prostate specific antigen and cellular proliferation in prostate cancer cells. J Cell Physiol. 2010;224 2:369-75. doi:10.1002/jcp.22132.
57. Chung WC, Zhou X, Atfi A and Xu K. PIK3CG Is a Potential Therapeutic Target in Androgen Receptor-Indifferent Metastatic Prostate Cancer. Am J Pathol. 2020;190 11:2194-202. doi:10.1016/j.ajpath.2020.07.013.
58. Ngan ESW, Hashimoto Y, Ma Z-Q, Tsai M-J and Tsai SY. Overexpression of Cdc25B, an androgen receptor coactivator, in prostate cancer. Oncogene. 2003;22 5:734-9. doi:10.1038/sj.onc.1206121.
59. Lin CY, Jan YJ, Kuo LK, Wang BJ, Huo C, Jiang SS, et al. Elevation of androgen receptor promotes prostate cancer metastasis by induction of epithelial-mesenchymal transition and reduction of KAT5. Cancer Sci. 2018;109 11:3564-74. doi:10.1111/cas.13776.
60. Chen S, Jiang X, Gewinner CA, Asara JM, Simon NI, Cai C, et al. Tyrosine kinase BMX phosphorylates phosphotyrosine-primed motif mediating the activation of multiple receptor tyrosine kinases. Sci Signal. 2013;6 277:ra40. doi:10.1126/scisignal.2003936.
61. Chen S, Cai C, Sowalsky AG, Ye H, Ma F, Yuan X, et al. BMX-Mediated Regulation of Multiple Tyrosine Kinases Contributes to Castration Resistance in Prostate Cancer. Cancer Res. 2018;78 18:5203-15. doi:10.1158/0008-5472.CAN-17-3615.
62. Sun X, Xin S, Zhang Y, Jin L, Liu X, Zhang J, et al. Long non‑coding RNA CASC11 interacts with YBX1 to promote prostate cancer progression by suppressing the p53 pathway. Int J Oncol. 2022;61 3 doi:10.3892/ijo.2022.5400.
63. Wedge DC, Gundem G, Mitchell T, Woodcock DJ, Martincorena I, Ghori M, et al. Sequencing of prostate cancers identifies new cancer genes, routes of progression and drug targets. Nat Genet. 2018;50 5:682-92. doi:10.1038/s41588-018-0086-z.
64. Li G, Fan M, Zheng Z, Zhang Y, Zhang Z, Huang Z, et al. Osteoblastic protein kinase D1 contributes to the prostate cancer cells dormancy via GAS6-circadian clock signaling. Biochim Biophys Acta Mol Cell Res. 2022;1869 9:119296. doi:10.1016/j.bbamcr.2022.119296.
65. Hennigs JK, Minner S, Tennstedt P, Loser R, Huland H, Klose H, et al. Subcellular Compartmentalization of Survivin is Associated with Biological Aggressiveness and Prognosis in Prostate Cancer. Sci Rep. 2020;10 1:3250. doi:10.1038/s41598-020-60064-9.
66. Khanmi K, Ignacimuthu S and Paulraj MG. MicroRNA in prostate cancer. Clin Chim Acta. 2015;451 Pt B:154-60. doi:10.1016/j.cca.2015.09.022.
67. de Brot S and Mongan NP. The Cell Cycle and Androgen Signaling Interactions in Prostate Cancer. In: Robinson BD, and Mosquera JM, and Ro JY and and Divatia M, editors. Precision Molecular Pathology of Prostate Cancer. Springer International Publishing; 2018. p. 381-404.
68. Yadav RK, Chauhan AS, Zhuang L and Gan B. FoxO transcription factors in cancer metabolism. Semin Cancer Biol. 2018;50:65-76. doi:10.1016/j.semcancer.2018.01.004.

69. Chang L, Graham PH, Ni J, Hao J, Bucci J, Cozzi PJ, et al. Targeting PI3K/Akt/mTOR signaling pathway in the treatment of prostate cancer radioresistance. Crit Rev Oncol Hematol. 2015;96 3:507-17. doi:10.1016/j.critrevonc.2015.07.005.

70. Bing T, Wang J, Shen L, Liu X and Shangguan D. Prion Protein Targeted by a Prostate Cancer Cell Binding Aptamer, a Potential Tumor Marker? ACS Appl Bio Mater. 2020;3 5:2658-65. doi:10.1021/acsabm.0c00024.

71. Xu H, Fu S, Chen Q, Gu M, Zhou J, Liu C, et al. The function of oxytocin: a potential biomarker for prostate cancer diagnosis and promoter of prostate cancer. Oncotarget. 2017;8 19:31215-26. doi:10.18632/oncotarget.16107.

72. Khan AS and Frigo DE. A spatiotemporal hypothesis for the regulation, role, and targeting of AMPK in prostate cancer. Nat Rev Urol. 2017;14 3:164-80. doi:10.1038/nrurol.2016.272.

73. Koumakpayi IH, Le Page C, Mes-Masson AM and Saad F. Hierarchical clustering of immunohistochemical analysis of the activated ErbB/PI3K/Akt/NF-kappaB signalling pathway and prognostic significance in prostate cancer. Br J Cancer. 2010;102 7:1163-73. doi:10.1038/sj.bjc.6605571.

74. Zhang L, Altuwaijri S, Deng F, Chen L, Lal P, Bhanot UK, et al. NF-kappaB regulates androgen receptor expression and prostate cancer growth. Am J Pathol. 2009;175 2:489-99. doi:10.2353/ajpath.2009.080727.

75. Huang H and Tindall DJ. Dynamic FoxO transcription factors. J Cell Sci. 2007;120 Pt 15:2479-87. doi:10.1242/jcs.001222.

76. Chen CC, Feng W, Lim PX, Kass EM and Jasin M. Homology-Directed Repair and the Role of BRCA1, BRCA2, and Related Proteins in Genome Integrity and Cancer. Annu Rev Cancer Biol. 2018;2:313-36. doi:10.1146/annurev-cancerbio-030617-050502.

77. Samarzija I. Post-Translational Modifications That Drive Prostate Cancer Progression. Biomolecules. 2021;11 2 doi:10.3390/biom11020247.

78. Hu JJ, Hall MC, Grossman L, Hedayati M, McCullough DL, Lohman K, et al. Deficient nucleotide excision repair capacity enhances human prostate cancer risk. Cancer Res. 2004;64 3:1197-201. doi:10.1158/0008-5472.can-03-2670.

79. Magnon C, Hall SJ, Lin J, Xue X, Gerber L, Freedland SJ, et al. Autonomic nerve development contributes to prostate cancer progression. Science. 2013;341 6142:1236361. doi:10.1126/science.1236361.

80. Jamaspishvili T, Berman DM, Ross AE, Scher HI, De Marzo AM, Squire JA, et al. Clinical implications of PTEN loss in prostate cancer. Nat Rev Urol. 2018;15 4:222-34. doi:10.1038/nrurol.2018.9.

81. Bisson I and Prowse DM. WNT signaling regulates self-renewal and differentiation of prostate cancer cells with stem cell characteristics. Cell Res. 2009;19 6:683-97. doi:10.1038/cr.2009.43.

82. Liu B, Qu J, Xu F, Guo Y, Wang Y, Yu H, et al. MiR-195 suppresses non-small cell lung cancer by targeting CHEK1. Oncotarget. 2015;6 11:9445-56. doi:10.18632/oncotarget.3255.

83. Yu X, Zhang Y, Ma X and Pertsemlidis A. miR-195 potentiates the efficacy of microtubule-targeting agents in non-small cell lung cancer. Cancer Lett. 2018;427:85-93. doi:10.1016/j.canlet.2018.04.007.

84. Li M, He F, Zhang Z, Xiang Z and Hu D. CDK1 serves as a potential prognostic biomarker and target for lung cancer. J Int Med Res. 2020;48 2:300060519897508. doi:10.1177/0300060519897508.
85. Qiao GB, Wu YL, Yang XN, Zhong WZ, Xie D, Guan XY, et al. High-level expression of Rad51 is an independent prognostic marker of survival in non-small-cell lung cancer patients. Br J Cancer. 2005;93 1:137-43. doi:10.1038/sj.bjc.6602665.
86. Zhang YQ, Bianco A, Malkinson AM, Leoni VP, Frau G, De Rosa N, et al. BARD1: an independent predictor of survival in non-small cell lung cancer. Int J Cancer. 2012;131 1:83-94. doi:10.1002/ijc.26346.
87. Zhou X, Wang N, Zhang Y, Yu H and Wu Q. KAT2B is an immune infiltration-associated biomarker predicting prognosis and response to immunotherapy in non-small cell lung cancer. Invest New Drugs. 2022;40 1:43-57. doi:10.1007/s10637-021-01159-6.
88. Zhang C, Lu J, Zhang QW, Zhao W, Guo JH, Liu SL, et al. USP7 promotes cell proliferation through the stabilization of Ki-67 protein in non-small cell lung cancer cells. Int J Biochem Cell Biol. 2016;79:209-21. doi:10.1016/j.biocel.2016.08.025.
89. Ren W, Li Y, Chen X, Hu S, Cheng W, Cao Y, et al. RYR2 mutation in non-small cell lung cancer prolongs survival via down-regulation of DKK1 and up-regulation of GS1-115G20.1: A weighted gene Co-expression network analysis and risk prognostic models. IET Syst Biol. 2022;16 2:43-58. doi:10.1049/syb2.12038.
90. Suzuki H, Ouchida M, Yamamoto H, Yano M, Toyooka S, Aoe M, et al. Decreased expression of the SIN3A gene, a candidate tumor suppressor located at the prevalent allelic loss region 15q23 in non-small cell lung cancer. Lung Cancer. 2008;59 1:24-31. doi:10.1016/j.lungcan.2007.08.002.
91. Li H, Yin C, Zhang B, Sun Y, Shi L, Liu N, et al. PTTG1 promotes migration and invasion of human non-small cell lung cancer cells and is modulated by miR-186. Carcinogenesis. 2013;34 9:2145-55. doi:10.1093/carcin/bgt158.
92. Shi X, Young S and Morahan G. Identification of Genetic Variants Associated with Sex-Specific Lung-Cancer Risk. Cancers (Basel). 2021;13 24 doi:10.3390/cancers13246379.
93. Wu J, He K, Zhang Y, Song J, Shi Z, Chen W, et al. Inactivation of SMARCA2 by promoter hypermethylation drives lung cancer development. Gene. 2019;687:193-9. doi:10.1016/j.gene.2018.11.032.
94. Lu Y, Yu S, Wang G, Ma Z, Fu X, Cao Y, et al. Elevation of EIF4G1 promotes non-small cell lung cancer progression by activating mTOR signalling. J Cell Mol Med. 2021;25 6:2994-3005. doi:10.1111/jcmm.16340.
95. Akhtar N and Bansal JG. Risk factors of Lung Cancer in nonsmoker. Curr Probl Cancer. 2017;41 5:328-39. doi:10.1016/j.currproblcancer.2017.07.002.
96. Li J, Wang L, Chen X, Li L, Li Y, Ping Y, et al. CD39/CD73 upregulation on myeloid-derived suppressor cells via TGF-beta-mTOR-HIF-1 signaling in patients with non-small cell lung cancer. Oncoimmunology. 2017;6 6:e1320011. doi:10.1080/2162402X.2017.1320011.
97. Liu YC, Yeh CT and Lin KH. Molecular Functions of Thyroid Hormone Signaling in Regulation of Cancer Progression and Anti-Apoptosis. Int J Mol Sci. 2019;20 20 doi:10.3390/ijms20204986.
98. Lv P, Man S, Xie L, Ma L and Gao W. Pathogenesis and therapeutic strategy in platinum

resistance lung cancer. Biochim Biophys Acta Rev Cancer. 2021;1876 1:188577. doi:10.1016/j.bbcan.2021.188577.

99. Logan RW, Zhang C, Murugan S, O'Connell S, Levitt D, Rosenwasser AM, et al. Chronic shift-lag alters the circadian clock of NK cells and promotes lung cancer growth in rats. J Immunol. 2012;188 6:2583-91. doi:10.4049/jimmunol.1102715.

100. Han ZJ, Feng YH, Gu BH, Li YM and Chen H. The post-translational modification, SUMOylation, and cancer (Review). Int J Oncol. 2018;52 4:1081-94. doi:10.3892/ijo.2018.4280.

101. Omori S, Takiguchi Y, Suda A, Sugimoto T, Miyazawa H, Takiguchi Y, et al. Suppression of a DNA double-strand break repair gene, Ku70, increases radio- and chemosensitivity in a human lung carcinoma cell line. DNA Repair (Amst). 2002;1 4:299-310. doi:10.1016/s1568-7864(02)00006-x.

102. Krzesniak M, Zajkowicz A, Matuszczyk I and Rusin M. Rapamycin prevents strong phosphorylation of p53 on serine 46 and attenuates activation of the p53 pathway in A549 lung cancer cells exposed to actinomycin D. Mech Ageing Dev. 2014;139:11-21. doi:10.1016/j.mad.2014.06.002.

103. Calverley DC, Phang TL, Choudhury QG, Gao B, Oton AB, Weyant MJ, et al. Significant downregulation of platelet gene expression in metastatic lung cancer. Clin Transl Sci. 2010;3 5:227-32. doi:10.1111/j.1752-8062.2010.00226.x.

104. Chen K, Hu Z, Wang LE, Zhang W, El-Naggar AK, Sturgis EM, et al. Polymorphic TP53BP1 and TP53 gene interactions associated with risk of squamous cell carcinoma of the head and neck. Clin Cancer Res. 2007;13 14:4300-5. doi:10.1158/1078-0432.CCR-07-0469.

105. Wild CA, Brandau S, Lotfi R, Mattheis S, Gu X, Lang S, et al. HMGB1 is overexpressed in tumor cells and promotes activity of regulatory T cells in patients with head and neck cancer. Oral Oncol. 2012;48 5:409-16. doi:10.1016/j.oraloncology.2011.12.009.

106. Skinner HD, Giri U, Yang L, Woo SH, Story MD, Pickering CR, et al. Proteomic Profiling Identifies PTK2/FAK as a Driver of Radioresistance in HPV-negative Head and Neck Cancer. Clin Cancer Res. 2016;22 18:4643-50. doi:10.1158/1078-0432.CCR-15-2785.

107. Jin Y, Jung SN, Lim MA, Oh C, Piao Y, Kim HJ, et al. Transcriptional Regulation of GDF15 by EGR1 Promotes Head and Neck Cancer Progression through a Positive Feedback Loop. Int J Mol Sci. 2021;22 20 doi:10.3390/ijms222011151.

108. Park JJ, Hah YS, Ryu S, Cheon SY, Won SJ, Lee JS, et al. MDM2-dependent Sirt1 degradation is a prerequisite for Sirt6-mediated cell death in head and neck cancers. Exp Mol Med. 2021;53 3:422-31. doi:10.1038/s12276-021-00578-y.

109. Yang K, Zhang S, Zhang D, Tao Q, Zhang T, Liu G, et al. Identification of SERPINE1, PLAU and ACTA1 as biomarkers of head and neck squamous cell carcinoma based on integrated bioinformatics analysis. Int J Clin Oncol. 2019;24 9:1030-41. doi:10.1007/s10147-019-01435-9.

110. Schnoell J, Jank BJ, Kadletz-Wanke L, Stoiber S, Spielvogel CP, Gurnhofer E, et al. Transcription factors CP2 and YY1 as prognostic markers in head and neck squamous cell carcinoma: analysis of The Cancer Genome Atlas and a second independent cohort. J Cancer Res Clin Oncol. 2021;147 3:755-65. doi:10.1007/s00432-020-03482-6.

111. Van Limbergen EJ, Zabrocki P, Porcu M, Hauben E, Cools J and Nuyts S. FLT1 kinase is a

mediator of radioresistance and survival in head and neck squamous cell carcinoma. Acta Oncol. 2014;53 5:637-45. doi:10.3109/0284186X.2013.835493.
112. Jerhammar F, Ceder R, Garvin S, Grenman R, Grafstrom RC and Roberg K. Fibronectin 1 is a potential biomarker for radioresistance in head and neck squamous cell carcinoma. Cancer Biol Ther. 2010;10 12:1244-51. doi:10.4161/cbt.10.12.13432.
113. Selvarajan V, Osato M, Nah GSS, Yan J, Chung TH, Voon DC, et al. RUNX3 is oncogenic in natural killer/T-cell lymphoma and is transcriptionally regulated by MYC. Leukemia. 2017;31 10:2219-27. doi:10.1038/leu.2017.40.
114. Tanaka N, Zhao M, Tang L, Patel AA, Xi Q, Van HT, et al. Gain-of-function mutant p53 promotes the oncogenic potential of head and neck squamous cell carcinoma cells by targeting the transcription factors FOXO3a and FOXM1. Oncogene. 2018;37 10:1279-92. doi:10.1038/s41388-017-0032-z.
115. Fakhry C and Gillison ML. Clinical implications of human papillomavirus in head and neck cancers. J Clin Oncol. 2006;24 17:2606-11. doi:10.1200/JCO.2006.06.1291.
116. Sheeja K and Lakshmi S. Nod-like receptor protein 3 inflammasome in head-and-neck cancer. J Cancer Res Ther. 2020;16 3:405-9. doi:10.4103/jcrt.JCRT_849_18.
117. Canel M, Secades P, Rodrigo JP, Cabanillas R, Herrero A, Suarez C, et al. Overexpression of focal adhesion kinase in head and neck squamous cell carcinoma is independent of fak gene copy number. Clin Cancer Res. 2006;12 11 Pt 1:3272-9. doi:10.1158/1078-0432.CCR-05-1583.
118. Keysar SB, Le PN, Anderson RT, Morton JJ, Bowles DW, Paylor JJ, et al. Hedgehog signaling alters reliance on EGF receptor signaling and mediates anti-EGFR therapeutic resistance in head and neck cancer. Cancer Res. 2013;73 11:3381-92. doi:10.1158/0008-5472.CAN-12-4047.
119. Jackson-Bernitsas DG, Ichikawa H, Takada Y, Myers JN, Lin XL, Darnay BG, et al. Evidence that TNF-TNFR1-TRADD-TRAF2-RIP-TAK1-IKK pathway mediates constitutive NF-kappaB activation and proliferation in human head and neck squamous cell carcinoma. Oncogene. 2007;26 10:1385-97. doi:10.1038/sj.onc.1209945.
120. Malumbres M and Barbacid M. Cell cycle, CDKs and cancer: a changing paradigm. Nat Rev Cancer. 2009;9 3:153-66. doi:10.1038/nrc2602.
121. Werbrouck J, De Ruyck K, Duprez F, Van Eijkeren M, Rietzschel E, Bekaert S, et al. Single-nucleotide polymorphisms in DNA double-strand break repair genes: association with head and neck cancer and interaction with tobacco use and alcohol consumption. Mutat Res. 2008;656 1-2:74-81. doi:10.1016/j.mrgentox.2008.07.013.
122. Lee TL, Yeh J, Van Waes C and Chen Z. Epigenetic modification of SOCS-1 differentially regulates STAT3 activation in response to interleukin-6 receptor and epidermal growth factor receptor signaling through JAK and/or MEK in head and neck squamous cell carcinomas. Mol Cancer Ther. 2006;5 1:8-19. doi:10.1158/1535-7163.MCT-05-0069.
123. Kodama. Toll-like receptor 3 signaling induces apoptosis in human head and neck cancer via survivin associated pathway. Oncology Reports. 2010;24 1 doi:10.3892/or_00000850.
124. Zhang H and Han W. Protein Post-translational Modifications in Head and Neck Cancer. Front Oncol. 2020;10:571944. doi:10.3389/fonc.2020.571944.
125. Cheng L, Sturgis EM, Eicher SA, Spitz MR and Wei Q. Expression of nucleotide excision

repair genes and the risk for squamous cell carcinoma of the head and neck. Cancer. 2002;94 2:393-7. doi:10.1002/cncr.10231.

126. Banerjee R, Russo N, Liu M, Basrur V, Bellile E, Palanisamy N, et al. TRIP13 promotes error-prone nonhomologous end joining and induces chemoresistance in head and neck cancer. Nat Commun. 2014;5:4527. doi:10.1038/ncomms5527.

127. Cohen EE, Lingen MW, Zhu B, Zhu H, Straza MW, Pierce C, et al. Protein kinase C zeta mediates epidermal growth factor-induced growth of head and neck tumor cells by regulating mitogen-activated protein kinase. Cancer Res. 2006;66 12:6296-303. doi:10.1158/0008-5472.CAN-05-3139.

128. Chang WM, Lin YF, Su CY, Peng HY, Chang YC, Hsiao JR, et al. Parathyroid Hormone-Like Hormone is a Poor Prognosis Marker of Head and Neck Cancer and Promotes Cell Growth via RUNX2 Regulation. Sci Rep. 2017;7:41131. doi:10.1038/srep41131.

129. Kim MH, Kim HB, Yoon SP, Lim SC, Cha MJ, Jeon YJ, et al. Colon cancer progression is driven by APEX1-mediated upregulation of Jagged. J Clin Invest. 2013;123 8:3211-30. doi:10.1172/JCI65521.

130. Zhao Z, Zhang G and Li W. Elevated Expression of ERCC6 Confers Resistance to 5-Fluorouracil and Is Associated with Poor Patient Survival in Colorectal Cancer. DNA Cell Biol. 2017;36 9:781-6. doi:10.1089/dna.2017.3768.

131. Semba S, Itoh N, Ito M, Youssef EM, Harada M, Moriya T, et al. Down-regulation of PIK3CG, a catalytic subunit of phosphatidylinositol 3-OH kinase, by CpG hypermethylation in human colorectal carcinoma. Clin Cancer Res. 2002;8 12:3824-31.

132. Liu X, Li L, Bai J, Li L, Fan J, Fu Z, et al. Long noncoding RNA plasmacytoma variant translocation 1 promotes progression of colorectal cancer by sponging microRNA-152-3p and regulating E2F3/MAPK8 signaling. Cancer Sci. 2022;113 1:109-19. doi:10.1111/cas.15113.

133. Jianping Bi, Ai Huang, Tao Liu, Tao Zhang and Ma H. Expression of DNA damage checkpoint 53BP1 is correlated with prognosis, cell proliferation and apoptosis in colorectal cancer. International Journal of Clinical and Experimental Pathology. 2015;8 6:6070-82.

134. An T, Gong Y, Li X, Kong L, Ma P, Gong L, et al. USP7 inhibitor P5091 inhibits Wnt signaling and colorectal tumor growth. Biochem Pharmacol. 2017;131:29-39. doi:10.1016/j.bcp.2017.02.011.

135. Chen X, Sun K, Jiao S, Cai N, Zhao X, Zou H, et al. High levels of SIRT1 expression enhance tumorigenesis and associate with a poor prognosis of colorectal carcinoma patients. Sci Rep. 2014;4:7481. doi:10.1038/srep07481.

136. Beale G, Haagensen EJ, Thomas HD, Wang LZ, Revill CH, Payne SL, et al. Combined PI3K and CDK2 inhibition induces cell death and enhances in vivo antitumour activity in colorectal cancer. Br J Cancer. 2016;115 6:682-90. doi:10.1038/bjc.2016.238.

137. Wu C, Lyu J, Yang EJ, Liu Y, Zhang B and Shim JS. Targeting AURKA-CDC25C axis to induce synthetic lethality in ARID1A-deficient colorectal cancer cells. Nat Commun. 2018;9 1:3212. doi:10.1038/s41467-018-05694-4.

138. Gan Y, Li Y, Li T, Shu G and Yin G. CCNA2 acts as a novel biomarker in regulating the growth and apoptosis of colorectal cancer. Cancer Manag Res. 2018;10:5113-24. doi:10.2147/CMAR.S176833.

139. Cai X, Liu C, Zhang TN, Zhu YW, Dong X and Xue P. Down-regulation of FN1 inhibits colorectal carcinogenesis by suppressing proliferation, migration, and invasion. J Cell Biochem. 2018;119 6:4717-28. doi:10.1002/jcb.26651.
140. Vermeulen SJ, Nollet F, Teugels E, Vennekens KM, Malfait F, Philippe J, et al. The alphaE-catenin gene (CTNNA1) acts as an invasion-suppressor gene in human colon cancer cells. Oncogene. 1999;18 4:905-15. doi:10.1038/sj.onc.1202348.
141. Su N, Peng L, Xia B, Zhao Y, Xu A, Wang J, et al. Lyn is involved in CD24-induced ERK1/2 activation in colorectal cancer. Mol Cancer. 2012;11:43. doi:10.1186/1476-4598-11-43.
142. Han F, Zhang L, Liao S, Zhang Y, Qian L, Hou F, et al. The interaction between S100A2 and KPNA2 mediates NFYA nuclear import and is a novel therapeutic target for colorectal cancer metastasis. Oncogene. 2022;41 5:657-70. doi:10.1038/s41388-021-02116-6.
143. Serafin V, Persano L, Moserle L, Esposito G, Ghisi M, Curtarello M, et al. Notch3 signalling promotes tumour growth in colorectal cancer. J Pathol. 2011;224 4:448-60. doi:10.1002/path.2895.
144. Buhrmann C, Shayan P, Goel A and Shakibaei M. Resveratrol Regulates Colorectal Cancer Cell Invasion by Modulation of Focal Adhesion Molecules. Nutrients. 2017;9 10 doi:10.3390/nu9101073.
145. Mohammadi A, Mansoori B and Baradaran B. The role of microRNAs in colorectal cancer. Biomed Pharmacother. 2016;84:705-13. doi:10.1016/j.biopha.2016.09.099.
146. Garcia-Barros M, Coant N, Truman JP, Snider AJ and Hannun YA. Sphingolipids in colon cancer. Biochim Biophys Acta. 2014;1841 5:773-82. doi:10.1016/j.bbalip.2013.09.007.
147. Braoudaki M, Ahmad MS, Mustafov D, Seriah S, Siddiqui MN and Siddiqui SS. Chemokines and chemokine receptors in colorectal cancer; multifarious roles and clinical impact. Semin Cancer Biol. 2022;86 Pt 2:436-49. doi:10.1016/j.semcancer.2022.06.002.
148. Danielsen SA, Eide PW, Nesbakken A, Guren T, Leithe E and Lothe RA. Portrait of the PI3K/AKT pathway in colorectal cancer. Biochim Biophys Acta. 2015;1855 1:104-21. doi:10.1016/j.bbcan.2014.09.008.
149. Fang JY and Richardson BC. The MAPK signalling pathways and colorectal cancer. Lancet Oncol. 2005;6 5:322-7. doi:10.1016/S1470-2045(05)70168-6.
150. Damin DC, Caetano MB, Rosito MA, Schwartsmann G, Damin AS, Frazzon AP, et al. Evidence for an association of human papillomavirus infection and colorectal cancer. Eur J Surg Oncol. 2007;33 5:569-74. doi:10.1016/j.ejso.2007.01.014.
151. Lee YS, Chin YT, Shih YJ, Nana AW, Chen YR, Wu HC, et al. Thyroid Hormone Promotes beta-Catenin Activation and Cell Proliferation in Colorectal Cancer. Horm Cancer. 2018;9 3:156-65. doi:10.1007/s12672-018-0324-y.
152. Bonnet M, Buc E, Sauvanet P, Darcha C, Dubois D, Pereira B, et al. Colonization of the human gut by E. coli and colorectal cancer risk. Clin Cancer Res. 2014;20 4:859-67. doi:10.1158/1078-0432.CCR-13-1343.
153. Vicente CM, da Silva DA, Sartorio PV, Silva TD, Saad SS, Nader HB, et al. Heparan Sulfate Proteoglycans in Human Colorectal Cancer. Anal Cell Pathol (Amst). 2018;2018:8389595. doi:10.1155/2018/8389595.
154. Straus DS. TNFalpha and IL-17 cooperatively stimulate glucose metabolism and growth factor production in human colorectal cancer cells. Mol Cancer. 2013;12:78.

doi:10.1186/1476-4598-12-78.
155. Sciulli MG, Filabozzi P, Tacconelli S, Padovano R, Ricciotti E, Capone ML, et al. Platelet activation in patients with colorectal cancer. Prostaglandins Leukot Essent Fatty Acids. 2005;72 2:79-83. doi:10.1016/j.plefa.2004.10.006.
156. Bajenova O, Chaika N, Tolkunova E, Davydov-Sinitsyn A, Gapon S, Thomas P, et al. Carcinoembryonic antigen promotes colorectal cancer progression by targeting adherens junction complexes. Exp Cell Res. 2014;324 2:115-23. doi:10.1016/j.yexcr.2014.04.007.
157. Kang DW and Min do S. Positive feedback regulation between phospholipase D and Wnt signaling promotes Wnt-driven anchorage-independent growth of colorectal cancer cells. PLoS One. 2010;5 8:e12109. doi:10.1371/journal.pone.0012109.
158. Zhang YJ, Dai Q, Sun DF, Xiong H, Tian XQ, Gao FH, et al. mTOR signaling pathway is a target for the treatment of colorectal cancer. Ann Surg Oncol. 2009;16 9:2617-28. doi:10.1245/s10434-009-0555-9.
159. Schatoff EM, Leach BI and Dow LE. Wnt Signaling and Colorectal Cancer. Curr Colorectal Cancer Rep. 2017;13 2:101-10. doi:10.1007/s11888-017-0354-9.
160. Li TT, Ogino S and Qian ZR. Toll-like receptor signaling in colorectal cancer: carcinogenesis to cancer therapy. World J Gastroenterol. 2014;20 47:17699-708. doi:10.3748/wjg.v20.i47.17699.
161. Ebi H, Corcoran RB, Singh A, Chen Z, Song Y, Lifshits E, et al. Receptor tyrosine kinases exert dominant control over PI3K signaling in human KRAS mutant colorectal cancers. J Clin Invest. 2011;121 11:4311-21. doi:10.1172/JCI57909.
162. Li J, Huang L, Zhao H, Yan Y and Lu J. The Role of Interleukins in Colorectal Cancer. Int J Biol Sci. 2020;16 13:2323-39. doi:10.7150/ijbs.46651.
163. Leve F and Morgado-Diaz JA. Rho GTPase signaling in the development of colorectal cancer. J Cell Biochem. 2012;113 8:2549-59. doi:10.1002/jcb.24153.
164. Uhlitz F, Bischoff P, Peidli S, Sieber A, Trinks A, Luthen M, et al. Mitogen-activated protein kinase activity drives cell trajectories in colorectal cancer. EMBO Mol Med. 2021;13 10:e14123. doi:10.15252/emmm.202114123.
165. Zhu G, Jin L, Sun W, Wang S and Liu N. Proteomics of post-translational modifications in colorectal cancer: Discovery of new biomarkers. Biochim Biophys Acta Rev Cancer. 2022;1877 4:188735. doi:10.1016/j.bbcan.2022.188735.
166. Zhang Z, Zhang G, Gao Z, Li S, Li Z, Bi J, et al. Comprehensive analysis of differentially expressed genes associated with PLK1 in bladder cancer. BMC Cancer. 2017;17 1:861. doi:10.1186/s12885-017-3884-2.
167. Chen J, Cao L, Li Z and Li Y. SIRT1 promotes GLUT1 expression and bladder cancer progression via regulation of glucose uptake. Hum Cell. 2019;32 2:193-201. doi:10.1007/s13577-019-00237-5.
168. Yu CC, Li CF, Chen IH, Lai MT, Lin ZJ, Korla PK, et al. YWHAZ amplification/overexpression defines aggressive bladder cancer and contributes to chemo-/radio-resistance by suppressing caspase-mediated apoptosis. J Pathol. 2019;248 4:476-87. doi:10.1002/path.5274.
169. Zeng S, Liu A, Dai L, Yu X, Zhang Z, Xiong Q, et al. Prognostic value of TOP2A in bladder urothelial carcinoma and potential molecular mechanisms. BMC Cancer. 2019;19 1:604.

doi:10.1186/s12885-019-5814-y.
170. Li J, Qiu M, An Y, Huang J and Gong C. miR-7-5p acts as a tumor suppressor in bladder cancer by regulating the hedgehog pathway factor Gli3. Biochem Biophys Res Commun. 2018;503 3:2101-7. doi:10.1016/j.bbrc.2018.07.166.
171. Ogishima T, Shiina H, Breault JE, Terashima M, Honda S, Enokida H, et al. Promoter CpG hypomethylation and transcription factor EGR1 hyperactivate heparanase expression in bladder cancer. Oncogene. 2005;24 45:6765-72. doi:10.1038/sj.onc.1208811.
172. Klumper N, Syring I, Vogel W, Schmidt D, Muller SC, Ellinger J, et al. Mediator Complex Subunit MED1 Protein Expression Is Decreased during Bladder Cancer Progression. Front Med (Lausanne). 2017;4:30. doi:10.3389/fmed.2017.00030.
173. Zheng F, Wang M, Li Y, Huang C, Tao D, Xie F, et al. CircNR3C1 inhibits proliferation of bladder cancer cells by sponging miR-27a-3p and downregulating cyclin D1 expression. Cancer Lett. 2019;460:139-51. doi:10.1016/j.canlet.2019.06.018.
174. Lin YH, Tsui KH, Chang KS, Hou CP, Feng TH and Juang HH. Maspin is a PTEN-Upregulated and p53-Upregulated Tumor Suppressor Gene and Acts as an HDAC1 Inhibitor in Human Bladder Cancer. Cancers (Basel). 2019;12 1 doi:10.3390/cancers12010010.
175. Wei Z, Xu J, Li W, Ou L, Zhou Y, Wang Y, et al. SMARCC1 Enters the Nucleus via KPNA2 and Plays an Oncogenic Role in Bladder Cancer. Front Mol Biosci. 2022;9:902220. doi:10.3389/fmolb.2022.902220.
176. Xue Y, Ma G, Zhang Z, Hua Q, Chu H, Tong N, et al. A novel antisense long noncoding RNA regulates the expression of MDC1 in bladder cancer. Oncotarget. 2015;6 1:484-93. doi:10.18632/oncotarget.2861.
177. Yi L, Wang H, Li W, Ye K, Xiong W, Yu H, et al. The FOXM1/RNF26/p57 axis regulates the cell cycle to promote the aggressiveness of bladder cancer. Cell Death Dis. 2021;12 10:944. doi:10.1038/s41419-021-04260-z.
178. Chen C, Benjamin MS, Sun X, Otto KB, Guo P, Dong XY, et al. KLF5 promotes cell proliferation and tumorigenesis through gene regulation and the TSU-Pr1 human bladder cancer cell line. Int J Cancer. 2006;118 6:1346-55. doi:10.1002/ijc.21533.
179. Rose M, Maurer A, Wirtz J, Bleilevens A, Waldmann T, Wenz M, et al. EGFR activity addiction facilitates anti-ERBB based combination treatment of squamous bladder cancer. Oncogene. 2020;39 44:6856-70. doi:10.1038/s41388-020-01465-y.
180. Kong DB, Chen F and Sima N. Focal adhesion kinases crucially regulate TGFbeta-induced migration and invasion of bladder cancer cells via Src kinase and E-cadherin. Onco Targets Ther. 2017;10:1783-92. doi:10.2147/OTT.S122463.
181. Yoshino H, Seki N, Itesako T, Chiyomaru T, Nakagawa M and Enokida H. Aberrant expression of microRNAs in bladder cancer. Nat Rev Urol. 2013;10 7:396-404. doi:10.1038/nrurol.2013.113.
182. Ohadian Moghadam S and Nowroozi MR. Toll-like receptors: The role in bladder cancer development, progression and immunotherapy. Scand J Immunol. 2019;90 6:e12818. doi:10.1111/sji.12818.
183. Dangle PP, Zaharieva B, Jia H and Pohar KS. Ras-MAPK pathway as a therapeutic target in cancer--emphasis on bladder cancer. Recent Pat Anticancer Drug Discov. 2009;4 2:125-36. doi:10.2174/157489209788452812.

184. Wu G, Weng W, Xia P, Yan S, Zhong C, Xie L, et al. Wnt signalling pathway in bladder cancer. Cell Signal. 2021;79:109886. doi:10.1016/j.cellsig.2020.109886.

185. Rampias T, Vgenopoulou P, Avgeris M, Polyzos A, Stravodimos K, Valavanis C, et al. A new tumor suppressor role for the Notch pathway in bladder cancer. Nat Med. 2014;20 10:1199-205. doi:10.1038/nm.3678.

186. Yang L, Li YL, Li XQ and Zhang Z. High Apelin Level Indicates a Poor Prognostic Factor in Muscle-Invasive Bladder Cancer. Dis Markers. 2019;2019:4586405. doi:10.1155/2019/4586405.

187. Ching CB and Hansel DE. Expanding therapeutic targets in bladder cancer: the PI3K/Akt/mTOR pathway. Lab Invest. 2010;90 10:1406-14. doi:10.1038/labinvest.2010.133.

188. Rani M, Kumari R, Singh SP, Devi A, Bansal P, Siddiqi A, et al. MicroRNAs as master regulators of FOXO transcription factors in cancer management. Life Sci. 2023;321:121535. doi:10.1016/j.lfs.2023.121535.

189. Oo HZ, Seiler R, Black PC and Daugaard M. Post-translational modifications in bladder cancer: Expanding the tumor target repertoire. Urol Oncol. 2020;38 12:858-66. doi:10.1016/j.urolonc.2018.09.001.

190. Liu B, Pan S, Liu J and Kong C. Cancer-associated fibroblasts and the related Runt-related transcription factor 2 (RUNX2) promote bladder cancer progression. Gene. 2021;775:145451. doi:10.1016/j.gene.2021.145451.

191. Ashrafizadeh M, Zarrabi A, Samarghandian S and Najafi M. PTEN: What we know of the function and regulation of this onco-suppressor factor in bladder cancer? Eur J Pharmacol. 2020;881:173226. doi:10.1016/j.ejphar.2020.173226.

192. Goriki A, Seiler R, Wyatt AW, Contreras-Sanz A, Bhat A, Matsubara A, et al. Unravelling disparate roles of NOTCH in bladder cancer. Nat Rev Urol. 2018;15 6:345-57. doi:10.1038/s41585-018-0005-1.

193. Meghani K, Folgosa Cooley L, Piunti A and Meeks JJ. Role of Chromatin Modifying Complexes and Therapeutic Opportunities in Bladder Cancer. Bladder Cancer. 2022;8 2:101-12. doi:10.3233/blc-211609.

194. Garcia-Closas M, Malats N, Real FX, Welch R, Kogevinas M, Chatterjee N, et al. Genetic variation in the nucleotide excision repair pathway and bladder cancer risk. Cancer Epidemiol Biomarkers Prev. 2006;15 3:536-42. doi:10.1158/1055-9965.EPI-05-0749.

195. Xia G, Kumar SR, Stein JP, Singh J, Krasnoperov V, Zhu S, et al. EphB4 receptor tyrosine kinase is expressed in bladder cancer and provides signals for cell survival. Oncogene. 2006;25 5:769-80. doi:10.1038/sj.onc.1209108.

196. Zhang Z, Zhang G and Kong C. Targeted inhibition of Polo-like kinase 1 by a novel small-molecule inhibitor induces mitotic catastrophe and apoptosis in human bladder cancer cells. J Cell Mol Med. 2017;21 4:758-67. doi:10.1111/jcmm.13018.

197. Wu E, Fan X, Tang T, Li J, Wang J, Liu X, et al. Biomarkers discovery for endometrial cancer: A graph convolutional sample network method. Comput Biol Med. 2022;150:106200. doi:10.1016/j.compbiomed.2022.106200.

198. Ito F, Furukawa N and Nakai T. Evaluation of TOP2A as a Predictive Marker for Endometrial Cancer With Taxane-Containing Adjuvant Chemotherapy. Int J Gynecol Cancer. 2016;26 2:325-30. doi:10.1097/IGC.0000000000000607.

199. Kamata Y, Watanabe J, Nishimura Y, Arai T, Kawaguchi M, Hattori M, et al. High expression of skp2 correlates with poor prognosis in endometrial endometrioid adenocarcinoma. J Cancer Res Clin Oncol. 2005;131 9:591-6. doi:10.1007/s00432-005-0671-2.
200. Lee P and Li X. Expression Profiles of the Phosphatase and Tensin Homolog (PTEN), CDH1, and CDH2 Genes, and the Cell Membrane Protein, CD133, in the Ishikawa Human Endometrial Adenocarcinoma Cell Line. Med Sci Monit. 2019;25:9829-35. doi:10.12659/MSM.918787.
201. Yang Y, Zhou L, Lu L, Wang L, Li X, Jiang P, et al. A novel miR-193a-5p-YY1-APC regulatory axis in human endometrioid endometrial adenocarcinoma. Oncogene. 2013;32 29:3432-42. doi:10.1038/onc.2012.360.
202. Slomovitz BM and Coleman RL. The PI3K/AKT/mTOR pathway as a therapeutic target in endometrial cancer. Clin Cancer Res. 2012;18 21:5856-64. doi:10.1158/1078-0432.CCR-12-0662.
203. Goto T, Takano M, Albergaria A, Briese J, Pomeranz KM, Cloke B, et al. Mechanism and functional consequences of loss of FOXO1 expression in endometrioid endometrial cancer cells. Oncogene. 2008;27 1:9-19. doi:10.1038/sj.onc.1210626.
204. Knapp P, Baranowski M, Knapp M, Zabielski P, Blachnio-Zabielska AU and Gorski J. Altered sphingolipid metabolism in human endometrial cancer. Prostaglandins Other Lipid Mediat. 2010;92 1-4:62-6. doi:10.1016/j.prostaglandins.2010.03.002.
205. Liu A, Zhang D, Yang X and Song Y. Estrogen receptor alpha activates MAPK signaling pathway to promote the development of endometrial cancer. J Cell Biochem. 2019;120 10:17593-601. doi:10.1002/jcb.29027.
206. Mu N, Zhu Y, Wang Y, Zhang H and Xue F. Insulin resistance: a significant risk factor of endometrial cancer. Gynecol Oncol. 2012;125 3:751-7. doi:10.1016/j.ygyno.2012.03.032.
207. Fue M, Miki Y, Takagi K, Hashimoto C, Yaegashi N, Suzuki T, et al. Relaxin 2/RXFP1 Signaling Induces Cell Invasion via the beta-Catenin Pathway in Endometrial Cancer. Int J Mol Sci. 2018;19 8 doi:10.3390/ijms19082438.
208. Wu ZS, Yang K, Wan Y, Qian PX, Perry JK, Chiesa J, et al. Tumor expression of human growth hormone and human prolactin predict a worse survival outcome in patients with mammary or endometrial carcinoma. J Clin Endocrinol Metab. 2011;96 10:E1619-29. doi:10.1210/jc.2011-1245.
209. Mamat Yusof MN, Chew KT, Kampan N, Abd Aziz NH, Md Zin RR, Tan GC, et al. PD-L1 Expression in Endometrial Cancer and Its Association with Clinicopathological Features: A Systematic Review and Meta-Analysis. Cancers (Basel). 2022;14 16 doi:10.3390/cancers14163911.
210. Banno K, Yanokura M, Kisu I, Yamagami W, Susumu N and Aoki D. MicroRNAs in endometrial cancer. Int J Clin Oncol. 2013;18 2:186-92. doi:10.1007/s10147-013-0526-9.
211. Sahoo SS, Lombard JM, Ius Y, O'Sullivan R, Wood LG, Nahar P, et al. Adipose-Derived VEGF-mTOR Signaling Promotes Endometrial Hyperplasia and Cancer: Implications for Obese Women. Mol Cancer Res. 2018;16 2:309-21. doi:10.1158/1541-7786.MCR-17-0466.
212. Oge T, Yalcin OT, Ozalp SS and Isikci T. Platelet volume as a parameter for platelet activation in patients with endometrial cancer. J Obstet Gynaecol. 2013;33 3:301-4.

doi:10.3109/01443615.2012.758089.
213. Pu X, Gu Z and Gu Z. ALKBH5 regulates IGF1R expression to promote the Proliferation and Tumorigenicity of Endometrial Cancer. J Cancer. 2020;11 19:5612-22. doi:10.7150/jca.46097.
214. Tian W, Teng F, Gao J, Gao C, Liu G, Zhang Y, et al. Estrogen and insulin synergistically promote endometrial cancer progression via crosstalk between their receptor signaling pathways. Cancer Biol Med. 2019;16 1:55-70. doi:10.20892/j.issn.2095-3941.2018.0157.
215. Li LY, Yin KM, Bai YH, Zhang ZG, Di W and Zhang S. CTHRC1 promotes M2-like macrophage recruitment and myometrial invasion in endometrial carcinoma by integrin-Akt signaling pathway. Clin Exp Metastasis. 2019;36 4:351-63. doi:10.1007/s10585-019-09971-4.
216. Azadehrah M, Vosoogh S and Azadehrah M. The roles and therapeutic applications of cytokines in endometrial cancer. J Reprod Immunol. 2022;152:103652. doi:10.1016/j.jri.2022.103652.
217. Guo Y, Liu B, Liu Y, Sun W, Gao W, Mao S, et al. Oncogenic Chromatin Modifier KAT2A Activates MCT1 to Drive the Glycolytic Process and Tumor Progression in Renal Cell Carcinoma. Frontiers in Cell and Developmental Biology. 2021;9 doi:10.3389/fcell.2021.690796.
218. Wang J, Jin J, Liang Y, Zhang Y, Wu N, Fan M, et al. miR-21-5p/PRKCE axis implicated in immune infiltration and poor prognosis of kidney renal clear cell carcinoma. Front Genet. 2022;13:978840. doi:10.3389/fgene.2022.978840.
219. Ramakrishnan S, Ku S, Ciamporcero E, Miles KM, Attwood K, Chintala S, et al. HDAC 1 and 6 modulate cell invasion and migration in clear cell renal cell carcinoma. BMC Cancer. 2016;16:617. doi:10.1186/s12885-016-2604-7.
220. Chen D, Maruschke M, Hakenberg O, Zimmermann W, Stief CG and Buchner A. TOP2A, HELLS, ATAD2, and TET3 Are Novel Prognostic Markers in Renal Cell Carcinoma. Urology. 2017;102:265 e1- e7. doi:10.1016/j.urology.2016.12.050.
221. Oh E, Kim JH, Um J, Jung DW, Williams DR and Lee H. Genome-Wide Transcriptomic Analysis of Non-Tumorigenic Tissues Reveals Aging-Related Prognostic Markers and Drug Targets in Renal Cell Carcinoma. Cancers (Basel). 2021;13 12 doi:10.3390/cancers13123045.
222. Ping JG, Wang F, Pu JX, Hou PF, Chen YS, Bai J, et al. The expression of Cullin1 is increased in renal cell carcinoma and promotes cancer cell proliferation, migration, and invasion. Tumour Biol. 2016;37 9:12823-31. doi:10.1007/s13277-016-5151-6.
223. Zhao H, Chen L, Yang T, Feng YL, Vaziri ND, Liu BL, et al. Aryl hydrocarbon receptor activation mediates kidney disease and renal cell carcinoma. J Transl Med. 2019;17 1:302. doi:10.1186/s12967-019-2054-5.
224. Soares MJ, Pinto M, Henrique R, Vieira J, Cerveira N, Peixoto A, et al. CSF1R copy number changes, point mutations, and RNA and protein overexpression in renal cell carcinomas. Mod Pathol. 2009;22 6:744-52. doi:10.1038/modpathol.2009.43.
225. Puzianowska-Kuznicka M, Nauman A, Madej A, Tanski Z, Cheng S and Nauman J. Expression of thyroid hormone receptors is disturbed in human renal clear cell carcinoma. Cancer Lett. 2000;155 2:145-52. doi:10.1016/s0304-3835(00)00416-x.
226. Li Y, Xiao X, Chen H, Chen Z, Hu K and Yin D. Transcription factor NFYA promotes G1/S

cell cycle transition and cell proliferation by transactivating cyclin D1 and CDK4 in clear cell renal cell carcinoma. Am J Cancer Res. 2020;10 8:2446-63.

227. Sjolund J, Johansson M, Manna S, Norin C, Pietras A, Beckman S, et al. Suppression of renal cell carcinoma growth by inhibition of Notch signaling in vitro and in vivo. J Clin Invest. 2008;118 1:217-28. doi:10.1172/JCI32086.

228. Rooney N, Mason SM, McDonald L, Dabritz JHM, Campbell KJ, Hedley A, et al. RUNX1 Is a Driver of Renal Cell Carcinoma Correlating with Clinical Outcome. Cancer Res. 2020;80 11:2325-39. doi:10.1158/0008-5472.CAN-19-3870.

229. Wu J, Liu T, Sun L, Zhang S and Dong G. Long noncoding RNA SNHG4 promotes renal cell carcinoma tumorigenesis and invasion by acting as ceRNA to sponge miR-204-5p and upregulate RUNX2. Cancer Cell Int. 2020;20:514. doi:10.1186/s12935-020-01606-z.

230. Liu S, Ma X, Ai Q, Huang Q, Shi T, Zhu M, et al. NOTCH1 functions as an oncogene by regulating the PTEN/PI3K/AKT pathway in clear cell renal cell carcinoma. Urol Oncol. 2013;31 6:938-48. doi:10.1016/j.urolonc.2011.07.006.
