# Supplementary material for: DriverMP enables improved identification of cancer driver genes
Source: Gigascience. 2023 Dec 13;12:giad106. doi: 10.1093/gigascience/giad106 (PMC10716827; doi:10.1093/gigascience/giad106)
Supplement: giad106_Response_to_Reviewer_Comments_Original_Submission [file giad106_response_to_reviewer_comments_original_submission.pdf]

Author's Response To Reviewer Comments

Reviewer #1:
1. The authors pointed that new algorithms need to consider more meaningful biological information, however in this method, the authors used somatic mutation data, protein–protein interaction networks, and differential expression data which are widely used in this field. What is the new information here? In my opinion, this method is lack of innovation. The authors should point it well.
Response: Thank you for the valuable comment and we apologize for the confusion. In this study, we mainly focused on designing a new driver gene prediction method by deeply mining more meaningful biological information from widely used data, rather than developing new data. We summarize the innovations as follows.
(1) According to our observation that most driver genes in our benchmark reference have a driver neighbour in PPI networks, we propose a theory that assessment of gene pairs would capture cancer driver characteristics in a more realistic pattern than direct assessment of individual genes. Based on the above consideration, we developed a new computational method named DriverMP for effectively prioritizing genes by first ranking gene pairs.
(2) Considering that driver genes are expected to exhibit different expression patterns and simultaneously influence the expression of their interacting genes in a biological sub-network, a gene pair is ranked highly if it, as well as the sub-network centred on it, are highly differentially expressed. The new DriverMP approach quantifies the differential expression of the sub-network centred on a gene pair by defining a new differential expression network.
(3) According to another observation that driver genes tend to converge to a limited number of biological pathways or protein complexes, a gene pair is ranked highly by DriverMP if it is strongly associated with other mutated genes in a PPI network. The DriverMP framework quantifies the association strength between each gene pair and its mutated neighbors in the PPI network.
Combining the topological properties from both the PPI network and differential expression network, DriverMP generates an impact score for each gene pair and then prioritize individual genes based on their contributions in the gene pairs. We briefly point the innovations of DriverMP in the fifth paragraph of the Introduction section.

2. The authors compare DriverMP with eight state-of-the-art approaches, however, these approaches are a little old. There are many new related methods, it will be better if the authors introduce more new methods and compare with them.
Response: Thank you for the comment. We carefully reviewed related new methods published in recent years, such as driverMAPS (Zhao et al. 2019), MinNetRank (Wei et al. 2020), Driver_IRW (Wei et al. 2020), DriverSubNet (Zhang et al. 2021), OncoVar (Wang et al. 2021), MENC (Tang et al. 2021), HA-RAS (He et al. 2022) and DriverRWH (Wang et al. 2022), and tried to compare them with DriverMP. Some of them were added in the revised manuscript for performance comparison with DriverMP such as driverMAPS, OncoVar, DriverRWH, while the others were not due to the unavailability of their codes or the running errors of their codes. For example, MinNetRank can be successfully installed but it reported an error when running it. The authors did not provide the source code of DriverSubNet in their paper, but we found it on the GitHub page of the first author. However, the author did not provide installation steps of DriverSubNet. We tried a lot to make it run but it still failed. For the three methods Driver_IRW, MENC and HA-RAS, their source codes were not provided in their paper. With the addition of driverMAPS, OncoVar, and DriverRWH, we compared with a total of eleven methods, and the comparison results showed that DriverMP consistently performs better than all of them.

3. In many studies, the driver mutations are not same as driver genes. Genes harbor driver mutations are usually called driver genes and passenger mutations are not equal to passenger genes in most related studies. I suggest the authors should distinguish the definitions of these terminologies clearly.
Response: Thank you for the comment and we apologize for the confusion. We carefully distinguished the differences of these terminologies and revised them accordingly in the revised manuscript.

4. The definition of the passenger genes in this paper is that genes are not included in the CGC, however genes not in CGC may have been demonstrated as cancer genes in other database or studies, the definition

seems not reasonable.
Response: Thank you for the comment. We definitely agree with you about the issue. However, it is almost impossible for us to pick up those true passenger genes since there is no completely reliable gold standard. In order to make the definition of passenger genes more reasonable, we collected another four commonly used databases, CGCpointMut, HCD, MouseMut, and Rule2020. And then, a gene is defined as a passenger gene if it is not included in any the five databases (CGC, CGCpointMut, HCD, MouseMut, and Rule2020).

5. In the stability experiment, the authors use two conditions by random selecting samples in (1) the constructed differential expression matrix and (2) the mutation dataset, is the selection separate? If so, are the samples same in differential expression data and mutation data?
Response: Thank you for the comment. The selection is separate in this study to separately test the stability of DriverMP in terms of differential expression and mutation data. For example, if we randomly select 50% samples from the differential expression matrix to test the stability of DriverMP in terms of differential expression data, then all the samples of mutation data remain and vice versa. In fact, the samples are generally different in differential expression data and mutation data downloaded from TCGA, and DriverMP is designed to separately extract biological information from the two kinds of data and then combine them together to prioritize mutated genes. Therefore, the computation process of DriverMP is independent of whether the samples of these two datasets are the same or not.

6. There are many methods to calculate differential genes and there will be different differential genes using different methods, why do the authors select this one in this paper?
Response: Thank you for the comment. When we designed the DriverMP method, we tried multiple approaches for calculating differential genes, such as edgeR (Chen et al.) and DESeq2 (Love, Michael I et al.), which all made DriverMP perform worse than the current version. Just as you mentioned, different methods output different differential genes. Therefore, the reason why these methods made DriverMP perform worse may be that their predicted differential genes are not compatible with the framework or other modules of DriverMP. In contrast, the one used in this paper is very compatible with the framework of DriverMP, which makes it perform better.

Reviewer #2:
1. One significant observation in the paper is that neighbors of driver genes may also be driver genes, which I personally agree with. However, there might be a logical flaw in defining genes not included in CGC as passenger genes, as current driver gene research is not comprehensive, and the absence of genes in CGC does not necessarily mean they are not driver genes. Additionally, many databases define driver gene sets, and I believe it may be more reasonable to define genes not included in any driver gene database as passenger genes.
Response: Thank you for the comment. We definitely agree with you about the issue. In order to make the definition of passenger genes more reasonable, we collected another four commonly used databases, CGCpointMut, HCD, MouseMut, and Rule2020. And then, a gene is defined as a passenger gene if it is not included in any the five databases (CGC, CGCpointMut, HCD, MouseMut, and Rule2020).

2. Similarly, when evaluating the results of each model, the authors only use CGC as the driver gene set. Although CGC is a widely recognized dataset, most of the genes in it are pan-cancer driver genes, and the authors evaluate these methods on ten different types of cancer. Did the authors use the same gold standard for these ten cancer types? If so, there may be some bias in this evaluation, and the authors might consider validating their approach on more driver gene datasets, especially those specific to certain types of cancer.
Response: Thank you for the comment. We still totally agree with you about this issue. However, to the best of our knowledge, the widely recognized driver gene datasets (like CGC) for certain cancer types are currently lacking, and therefore, driver gene prediction methods usually evaluate their performance based on those commonly used driver gene databases, such as CGC. Just as the paper (Briefings in Bioinformatics, 2022, 23(2), 1-14) you mentioned, the authors evaluate the performance of driver gene predictors on 36 specific cancer types by also using driver gene databases like CGC, CGCpointMut, etc. In order to make the evaluation process more reasonable, as mentioned above, we selected more driver gene databases in the revised manuscript.

3. In the paper, the AUC values for the comparison methods are mostly below 0.7, and even below 0.6.

This result is inconsistent with some previous papers, such as the one in Briefings in Bioinformatics, 2022, 23(2), 1-14, which systematically evaluated the predictive performance of various methods. In their paper, methods like DNmax and DNsum had AUCs above 0.7, which differs from the results in Liu et al. Furthermore, some methods like DORGE performed well, so the authors should carefully review their evaluation process to ensure its reliability and compare it with methods that perform even better.
Response: Thank you for the comment. The calculated AUC values in this paper is indeed different from the ones in the paper you mentioned because we made a normalization for each method before plotting ROC curves. In this paper, we plotted the ROC curves and calculated corresponding AUC values by first normalizing the number of genes output by each of the compared methods. In fact, the numbers of genes output by the methods are different, e.g., on the BRCA data, the number of genes output by MaxMIF is 33855, DNmax and DNsum output 17684 genes, and DawnRank output 5647 genes, etc. In addition, the number of genes output by a method largely affects its ROC curve and AUC value, and a larger number of genes generally corresponds to a higher AUC value for a method with a possible reason that most driver genes distribute among top ranked genes. Taking MaxMIF as an example, when we plot an ROC curve by using all the 33855 output genes, its AUC value is 0.8649, while it is 0.7287 when we select top 17684 genes (the same with DNmax and DNsum), and it is only 0.6745 if we select top 5647 genes (the same with DawnRank). Therefore, in order to make a fair comparison among the methods, we in this study normalize the number of genes by selecting top N genes for each method, where N is the smallest number of genes output by all the compared methods (we described the calculation details in the "Evaluation criteria" subsection of the Methods section).
For the DORGE method, it is an excellent data-driven machine learning framework for identifying tumor suppressor genes (TSGs) and oncogene genes (OGs) by integrating comprehensive genetic and epigenetic data. DORGE constructed 75 features that might predict cancer driver genes based on the literature and trained two binary classification algorithms by using 242 TSGs and 240 OGs from the CGC database. As a machine learning algorithm, DORGE uses a large amount of genes from the CGC as its training set that are also used for evaluating the performance of all the compared methods. Therefore, it is not reasonable to compare it with the other methods. Despite all this, we still made a comparison between DORGE and DriverMP based on the provided results of DORGE on the four cancer types including Breast, Endometrium, Prostate, and Lung that are most similar with the BRCA, UCEC, PRAD, and LUAD datasets. Since DORGE has two modes, OG and TSG, we obtained two AUC values for it on each cancer type. After comparison, it shows that AUC-OG and AUC-TSG values of DORGE are respectively 0.7213 and 0.7156 on BRCA, 0.7381 and 0.7352 on UCEC, 0.7188 and 0.7107 on PRAD, and 0.7366 and 0.7352 on LUAD. The AUC values of DriverMP on the four cancer types are respectively 0.7239, 0.7452, 0.6921, and 0.7306. From the above comparison, even though DORGE has the advantage of "knowing the answer before the test", DriverMP still demonstrates very comparable performance.

4. As the authors mentioned, there have been numerous works using biological networks, mutation information, and omics data, including gene expression, for cancer driver gene prediction. It is recommended that the authors provide a more explicit description of the innovation of their method in the paper.
Response: Thank you for the comment. In this study, we mainly focused on designing a new driver gene prediction method by deeply mining more meaningful biological information from widely used data. We summarize the innovations as follows.
(1) According to our observation that most driver genes in our benchmark reference have a driver neighbour in PPI networks, we propose a theory that assessment of gene pairs would capture cancer driver characteristics in a more realistic pattern than direct assessment of individual genes. Based on the above consideration, we developed a new computational method named DriverMP for effectively prioritizing genes by first ranking gene pairs.
(2) Considering that driver genes are expected to exhibit different expression patterns and simultaneously influence the expression of their interacting genes in a biological sub-network, a gene pair is ranked highly if it, as well as the sub-network centred on it, are highly differentially expressed. The new DriverMP approach quantifies the differential expression of the sub-network centred on a gene pair by defining a new differential expression network.
(3) According to another observation that driver genes tend to converge to a limited number of biological pathways or protein complexes, a gene pair is ranked highly by DriverMP if it is strongly associated with other mutated genes in a PPI network. The DriverMP framework quantifies the association strength between each gene pair and its mutated neighbors in the PPI network.

Combining the topological properties from both the PPI network and differential expression network, DriverMP generates an impact score for each gene pair and then prioritize individual genes based on their contributions in the gene pairs. We explicitly described the innovations of DriverMP in the fifth paragraph of the Introduction section.

5. The clarity of the figures in the paper is insufficient. Although the figures show that the performance of the proposed method in this paper is relatively good, it is suggested to include specific AUC values on the plots or present the exact AUC data in a table format in the paper.
Response: Thank you for the valuable comment. In the revised version, we added legends with AUC and AUFC values for each method to the ROC and F1-score figures.

Reviewer #3:
1. Perhaps the authors mean "driver genes" instead of "driver mutations" in the full title of this manuscript since the purpose of this manuscript is to detect driver genes, not driver mutations.
Response: Thank you for the comment and we apologize for the confusion. We carefully distinguished the differences of these terminologies and revised them accordingly in the revised manuscript.

2. The Introduction/Methods Section should clarify that non-silent somatic mutation data used in this study pertain only to coding regions. Studies have made marked advances in studying point mutations, gene fusion events and somatic rearrangements in intergenic regions with effects on cancer. In addition, silent mutations have also been identified to play important roles in cancer development. These findings should either be mentioned or the introduction should clarify that only non-silent somatic mutations affecting protein-coding genes are at the focus.
Response: Thank you for the comment. In this study, DriverMP focuses on the role of non-silent somatic mutational behaviors involved in protein coding on the identification of cancer driver genes, which was added in the Introduction section revised version.

3. It is not clear to me why the authors compared DriverMP with the state-of-the-art approaches published before 2018. It would be better for authors to justify why not compare with the recent representative methods, since this is the norm in the field.
Response: Thank you for the comment. We carefully reviewed related new methods published in recent years, such as driverMAPS (Zhao et al. 2019), MinNetRank (Wei et al. 2020), Driver_IRW (Wei et al. 2020), DriverSubNet (Zhang et al. 2021), OncoVar (Wang et al. 2021), MENC (Tang et al. 2021), HA-RAS (He et al. 2022) and DriverRWH (Wang et al. 2022), and tried to compare them with DriverMP. Some of them were added in the revised manuscript for performance comparison with DriverMP such as driverMAPS, OncoVar, DriverRWH, while the others were not added due to the unavailability of their codes or the running errors of their codes. For example, MinNetRank can be successfully installed but it reported an error when running it. The authors did not provide the source code of DriverSubNet in their paper, but we found it on the GitHub page of the first author. However, the author did not provide installation steps of DriverSubNet. We tried a lot to make it run but it still failed. For the three methods Driver_IRW, MENC and HA-RAS, their source codes were not provided in their paper. With the addition of driverMAPS, OncoVar, and DriverRWH, we compared with a total of eleven methods, and the comparison results showed that DriverMP consistently performs better than all of them.

4.The authors proposed a cancer-specific five-level assessment method to comprehensively evaluate the reliability of the novel driver candidates by applying various analytical approaches, which is of critical importance. I have a suggestion that the authors may add a gene level assessment. In this level, the authors can analyze whether the candidate gene has a homologue in the Cancer Gene Census. In addition, I would recommend using "Non-cancer disease level" instead of "Disease level".
Response: Thank you for the valuable comment. We added the gene-level assessment for all 10 cancers in the revised version. In detail, we used the BLAST tool to analyze the sequence similarity between the novel driver genes predicted by DriverMP and the genes in CGC, based on which homologues can be defined for predicted novel driver genes. In the revised version, we defined that a novel candidate has a homologue in CGC if the sequence similarity between the novel candidate and a CGC gene is higher than 40% with e-value lower than 10-4. In addition, we revised the “Disease level” into “Non-cancer disease level”.
