# Supplementary material for: DriverMP enables improved identification of cancer driver genes
Source: Gigascience. 2023 Dec 13;12:giad106. doi: 10.1093/gigascience/giad106 (PMC10716827; doi:10.1093/gigascience/giad106)
Supplement: giad106_Reviewer_1_Report_Original_Submission — Chunhou Zheng -- 9/1/2023 Reviewed [file giad106_reviewer_1_report_original_submission.pdf]

Reviewer Report

Title: DriverMP enables improved identification of cancer driver genes

Version: Original Submission **Date:** 9/1/2023

Reviewer name: Chunhou Zheng

Reviewer Comments to Author:

In this paper, the authors proposed a method called DriverMP for effectively prioritizing altered genes on a cancer-type level by considering mutated gene pairs. The experiments demonstrated that the identified driver genes are strongly supported by clinical experiments, disease enrichment, or biological pathway analysis. However, However, there are some questions I care about:1. The authors pointed that new algorithms need to consider more meaningful biological information, however in this method, the authors used somatic mutation data, proteinâ€'protein interaction networks, and differential expression data which are widely used in this field. What is the new information here? In my opinion, this method is lack of innovation. The authors should point it well.2. The authors compare DriverMP with eight state-of-the-art approaches, however, these approaches are a little old. There are many new related methods, it will be better if the authors introduce more new methods and compare with them.3. In many studies, the driver mutations are not same as driver genes. Genes harbor driver mutations are usually called driver genes and passenger mutations are not equal to passenger genes in most related studies. I suggest the authors should distinguish the definitions of these terminologies clearly.4. The definition of the passenger genes in this paper is that genes are not included in the CGC, however genes not in CGC may have been demonstrated as cancer genes in other database or studies, the definition seems not reasonable.5. In the stability experiment, the authors use two conditions by random selecting samples in (1) the constructed differential expression matrix and (2) the mutation dataset, is the selection separate? If so, are the samples same in differential expression data and mutation data?6. There are many methods to calculate differential genes and there will be different differential genes using different methods, why do the authors select this one in this paper?

Level of Interest

Please indicate how interesting you found the manuscript: Choose an item.

Quality of Written English

Please indicate the quality of language in the manuscript: Choose an item.

Declaration of Competing Interests

Please complete a declaration of competing interests, considering the following questions:

- Have you in the past five years received reimbursements, fees, funding, or salary from an organisation that may in any way gain or lose financially from the publication of this manuscript, either now or in the future?
- Do you hold any stocks or shares in an organisation that may in any way gain or lose financially from the publication of this manuscript, either now or in the future?
- Do you hold or are you currently applying for any patents relating to the content of the manuscript?
- Have you received reimbursements, fees, funding, or salary from an organization that holds or has applied for patents relating to the content of the manuscript?
- Do you have any other financial competing interests?
- Do you have any non-financial competing interests in relation to this paper?

If you can answer no to all of the above, write 'I declare that I have no competing interests' below. If your reply is yes to any, please give details below.

I declare that I have no competing interests.

I agree to the open peer review policy of the journal. I understand that my name will be included on my report to the authors and, if the manuscript is accepted for publication, my named report including any attachments I upload will be posted on the website along with the authors' responses. I agree for my report to be made available under an Open Access Creative Commons CC-BY license (http://creativecommons.org/licenses/by/4.0/). I understand that any comments which I do not wish to be included in my named report can be included as confidential comments to the editors, which will not be published.

Choose an item.

To further support our reviewers, we have joined with Publons, where you can gain additional credit to further highlight your hard work (see: https://publons.com/journal/530/gigascience). On publication of this paper, your review will be automatically added to Publons, you can then choose whether or not to claim your Publons credit. I understand this statement.

Yes Choose an item.
