# Supplementary material for: DriverMP enables improved identification of cancer driver genes
Source: Gigascience. 2023 Dec 13;12:giad106. doi: 10.1093/gigascience/giad106 (PMC10716827; doi:10.1093/gigascience/giad106)
Supplement: giad106_Reviewer_2_Report_Original_Submission — Xionghui Zhou -- 9/17/2023 Reviewed [file giad106_reviewer_2_report_original_submission.pdf]

Reviewer Report

Title: DriverMP enables improved identification of cancer driver genes

Version: Original Submission **Date:** 9/17/2023

Reviewer name: Xionghui Zhou

Reviewer Comments to Author:

Identification of cancer driver genes is crucial for cancer research. In this paper, based on the observation that genes neighboring driver genes in protein-protein interaction networks are more likely to be driver genes, Liu et al. proposed a new method called DriverMP that prioritizes cancer genes on a cancer-type level by utilizing somatic mutation data, protein-protein interaction networks, and differential expression data. Results on 10 cancer datasets confirm the superiority of their approach compared to nine other methods. Literature review and biological function analysis suggest that their method may identify new driver genes. Overall, the paper is well-organized, and the results are as expected. I have several major concerns:One significant observation in the paper is that neighbors of driver genes may also be driver genes, which I personally agree with. However, there might be a logical flaw in defining genes not included in CGC as passenger genes, as current driver gene research is not comprehensive, and the absence of genes in CGC does not necessarily mean they are not driver genes. Additionally, many databases define driver gene sets, and I believe it may be more reasonable to define genes not included in any driver gene database as passenger genes.Similarly, when evaluating the results of each model, the authors only use CGC as the driver gene set. Although CGC is a widely recognized dataset, most of the genes in it are pan-cancer driver genes, and the authors evaluate these methods on ten different types of cancer. Did the authors use the same gold standard for these ten cancer types? If so, there may be some bias in this evaluation, and the authors might consider validating their approach on more driver gene datasets, especially those specific to certain types of cancer.In the paper, the AUC values for the comparison methods are mostly below 0.7, and even below 0.6. This result is inconsistent with some previous papers, such as the one in Briefings in Bioinformatics, 2022, 23(2), 1-14, which systematically evaluated the predictive performance of various methods. In their paper, methods like DNmax and DNsum had AUCs above 0.7, which differs from the results in Liu et al. Furthermore, some methods like DORGE performed well, so the authors should carefully review their evaluation process to ensure its reliability and compare it with methods that perform even better.As the authors mentioned, there have been numerous works using biological networks, mutation information, and omics data, including gene expression, for cancer driver gene prediction. It is recommended that the authors provide a more explicit description of the innovation of their method in the paper.Minor issues:The clarity of the figures in the paper is insufficient.Although the figures show that the performance of the proposed method in this paper is relatively good, it is suggested to include specific AUC values on the plots or present the exact AUC data in a table format in the paper.

Level of Interest

Please indicate how interesting you found the manuscript: Choose an item.

Quality of Written English

Please indicate the quality of language in the manuscript: Choose an item.

Declaration of Competing Interests

Please complete a declaration of competing interests, considering the following questions:

- Have you in the past five years received reimbursements, fees, funding, or salary from an organisation that may in any way gain or lose financially from the publication of this manuscript, either now or in the future?
- Do you hold any stocks or shares in an organisation that may in any way gain or lose financially from the publication of this manuscript, either now or in the future?
- Do you hold or are you currently applying for any patents relating to the content of the manuscript?
- Have you received reimbursements, fees, funding, or salary from an organization that holds or has applied for patents relating to the content of the manuscript?
- Do you have any other financial competing interests?
- Do you have any non-financial competing interests in relation to this paper?

If you can answer no to all of the above, write 'I declare that I have no competing interests' below. If your reply is yes to any, please give details below.

I declare that I have no competing interests.

I agree to the open peer review policy of the journal. I understand that my name will be included on my report to the authors and, if the manuscript is accepted for publication, my named report including any attachments I upload will be posted on the website along with the authors' responses. I agree for my report to be made available under an Open Access Creative Commons CC-BY license (http://creativecommons.org/licenses/by/4.0/). I understand that any comments which I do not wish to be included in my named report can be included as confidential comments to the editors, which will not be published.

Choose an item.

To further support our reviewers, we have joined with Publons, where you can gain additional credit to further highlight your hard work (see: https://publons.com/journal/530/gigascience). On publication of this paper, your review will be automatically added to Publons, you can then choose whether or not to claim your Publons credit. I understand this statement.

Yes Choose an item.
