# Supplementary material for: DriverMP enables improved identification of cancer driver genes
Source: Gigascience. 2023 Dec 13;12:giad106. doi: 10.1093/gigascience/giad106 (PMC10716827; doi:10.1093/gigascience/giad106)
Supplement: giad106_Reviewer_3_Report_Original_Submission — Junfeng Xia -- 10/5/2023 Reviewed [file giad106_reviewer_3_report_original_submission.pdf]

Reviewer Report

Title: DriverMP enables improved identification of cancer driver genes

Version: Original Submission **Date:** 10/5/2023

Reviewer name: Junfeng Xia

Reviewer Comments to Author:

In this manuscript, the authors proposed a computational method called DriverMP for effectively prioritizing driver genes on a cancer-type level by considering mutated gene pairs. The results show that DriverMP obtained the highest AUC values compared with various representative methods. In addition, they also developed a user-friendly web server and a driver gene database for ten cancer types. The topic is interesting and important because cancer driver genes confer a selective advantage to cancers. I found the manuscript to be overall well-written and logically structured. However, some points should be addressed before acceptance.1. Perhaps the authors mean "driver genes" instead of "driver mutations" in the full title of this manuscript since the purpose of this manuscript is to detect driver genes, not driver mutations.2. The Introduction/Methods Section should clarify that non-silent somatic mutation data used in this study pertain only to coding regions. Studies have made marked advances in studying point mutations, gene fusion events and somatic rearrangements in intergenic regions with effects on cancer. In addition, silent mutations have also been identified to play important roles in cancer development. These findings should either be mentioned or the introduction should clarify that only non-silent somatic mutations affecting protein-coding genes are at the focus.3. It is not clear to me why the authors compared DriverMP with the state-of-the-art approaches published before 2018. It would be better for authors to justify why not compare with the recent representative methods, since this is the norm in the field.4.The authors proposed a cancer-specific five-level assessment method to comprehensively evaluate the reliability of the novel driver candidates by applying various analytical approaches, which is of critical importance. I have a suggestion that the authors may add a gene level assessment. In this level, the authors can analyze whether the candidate gene has a homologue in the Cancer Gene Census. In addition, I would recommend using "Non-cancer disease level" instead of "Disease level".

Level of Interest

Please indicate how interesting you found the manuscript: Choose an item.

Quality of Written English

Please indicate the quality of language in the manuscript: Choose an item.

Declaration of Competing Interests

Please complete a declaration of competing interests, considering the following questions:

- Have you in the past five years received reimbursements, fees, funding, or salary from an organisation that may in any way gain or lose financially from the publication of this manuscript, either now or in the future?
- Do you hold any stocks or shares in an organisation that may in any way gain or lose financially from the publication of this manuscript, either now or in the future?
- Do you hold or are you currently applying for any patents relating to the content of the manuscript?
- Have you received reimbursements, fees, funding, or salary from an organization that holds or has applied for patents relating to the content of the manuscript?
- Do you have any other financial competing interests?
- Do you have any non-financial competing interests in relation to this paper?

If you can answer no to all of the above, write 'I declare that I have no competing interests' below. If your reply is yes to any, please give details below.

I declare that I have no competing interests

I agree to the open peer review policy of the journal. I understand that my name will be included on my report to the authors and, if the manuscript is accepted for publication, my named report including any attachments I upload will be posted on the website along with the authors' responses. I agree for my report to be made available under an Open Access Creative Commons CC-BY license (http://creativecommons.org/licenses/by/4.0/). I understand that any comments which I do not wish to be included in my named report can be included as confidential comments to the editors, which will not be published.

Choose an item.

To further support our reviewers, we have joined with Publons, where you can gain additional credit to further highlight your hard work (see: https://publons.com/journal/530/gigascience). On publication of this paper, your review will be automatically added to Publons, you can then choose whether or not to claim your Publons credit. I understand this statement.

Yes Choose an item.
